# Supplementary material for: Immersive virtual reality-based intervention for psychological wellbeing among older adults: A systematic review and meta-analysis
Source: PLOS Digit Health. 2026 Jan 15;5(1):e0001110. doi: 10.1371/journal.pdig.0001110 (PMC12806852; doi:10.1371/journal.pdig.0001110)
Supplement: S1 File — (DOCX) [file pdig.0001110.s001.docx]

Supplementary 1: The MeSH terms and search strategies for each database

MeSH terms

(older OR senior* OR elder* OR aging OR ageing) AND (mental or "mental health" OR "mental disorder*" OR "mental illness*" OR "mental disease*" OR "mood disorder*" OR depressi* OR anxiety OR "mental wellbeing" OR "mental well-being" OR "psychological wellbeing" OR "psychological well-being" OR stress OR distress OR loneliness) AND ("virtual reality" OR virtual-reality OR immerse OR immersive* OR VR OR head mounted OR face mounted OR cave).

Search strategy for PubMed:

((older OR senior* OR elder* OR aging OR ageing) AND (mental or mental health OR mental disorder* OR mental illness* OR mental disease* OR mood disorder* OR depressi* OR anxiety OR mental wellbeing OR mental well-being OR psychological wellbeing OR psychological well-being OR stress OR distress OR loneliness)) AND (virtual reality OR virtual-reality OR immerse OR immersive* OR VR OR head mounted OR face mounted OR cave)

("older"[All Fields] OR "olders"[All Fields] OR "senior*"[All Fields] OR "elder*"[All Fields] OR ("aging"[MeSH Terms] OR "aging"[All Fields] OR "ageing"[All Fields]) OR ("aging"[MeSH Terms] OR "aging"[All Fields] OR "ageing"[All Fields])) AND ("mental"[All Fields] OR "mentalities"[All Fields] OR "mentality"[All Fields] OR "mentalization"[MeSH Terms] OR "mentalization"[All Fields] OR "mentalizing"[All Fields] OR "mentalize"[All Fields] OR "mentalized"[All Fields] OR "mentally"[All Fields] OR ("mental health"[MeSH Terms] OR ("mental"[All Fields] AND "health"[All Fields]) OR "mental health"[All Fields]) OR (("mental"[All Fields] OR "mentalities"[All Fields] OR "mentality"[All Fields] OR "mentalization"[MeSH Terms] OR "mentalization"[All Fields] OR "mentalizing"[All Fields] OR "mentalize"[All Fields] OR "mentalized"[All Fields] OR "mentally"[All Fields]) AND "disorder*"[All Fields]) OR (("mental"[All Fields] OR "mentalities"[All Fields] OR "mentality"[All Fields] OR "mentalization"[MeSH Terms] OR "mentalization"[All Fields] OR "mentalizing"[All Fields] OR "mentalize"[All Fields] OR "mentalized"[All Fields] OR "mentally"[All Fields]) AND "illness*"[All Fields]) OR (("mental"[All Fields] OR "mentalities"[All Fields] OR "mentality"[All Fields] OR "mentalization"[MeSH Terms] OR "mentalization"[All Fields] OR "mentalizing"[All Fields] OR "mentalize"[All Fields] OR "mentalized"[All Fields] OR "mentally"[All Fields]) AND "disease*"[All Fields]) OR (("affect"[MeSH Terms] OR "affect"[All Fields] OR "mood"[All Fields]) AND "disorder*"[All Fields]) OR "depressi*"[All Fields] OR ("anxiety"[MeSH Terms] OR "anxiety"[All Fields] OR "anxieties"[All Fields] OR "anxiety s"[All Fields]) OR (("mental"[All Fields] OR "mentalities"[All Fields] OR "mentality"[All Fields] OR "mentalization"[MeSH Terms] OR "mentalization"[All Fields] OR "mentalizing"[All Fields] OR "mentalize"[All Fields] OR "mentalized"[All Fields] OR "mentally"[All Fields]) AND "wellbeing"[All Fields]) OR ("mental health"[MeSH Terms] OR ("mental"[All Fields] AND "health"[All Fields]) OR "mental health"[All Fields] OR ("mental"[All Fields] AND "well"[All Fields]) OR "mental well being"[All Fields]) OR (("psychologic"[All Fields] OR "psychological"[All Fields] OR "psychologically"[All Fields] OR "psychologization"[All Fields] OR "psychologized"[All Fields] OR "psychologizing"[All Fields]) AND "wellbeing"[All Fields]) OR ("psychological well being"[MeSH Terms] OR ("psychological"[All Fields] AND "well being"[All Fields]) OR "psychological well being"[All Fields] OR ("psychological"[All Fields] AND "well"[All Fields]) OR "psychological well being"[All Fields]) OR ("stress"[All Fields] OR "stressed"[All Fields] OR "stresses"[All Fields] OR "stressful"[All Fields] OR "stressfulness"[All Fields] OR "stressing"[All Fields]) OR ("distress"[All Fields] OR "distressed"[All Fields] OR "distresses"[All Fields] OR "distressful"[All Fields] OR "distressing"[All Fields]) OR ("lonelier"[All Fields] OR "loneliness"[MeSH Terms] OR "loneliness"[All Fields])) AND ("virtual reality"[MeSH Terms] OR ("virtual"[All Fields] AND "reality"[All Fields]) OR "virtual reality"[All Fields] OR ("virtual reality"[MeSH Terms] OR ("virtual"[All Fields] AND "reality"[All Fields]) OR "virtual reality"[All Fields]) OR ("immerse"[All Fields] OR "immersed"[All Fields] OR "immerses"[All Fields] OR "immersing"[All Fields] OR "immersion"[MeSH Terms] OR "immersion"[All Fields] OR "immersions"[All Fields] OR "immersive"[All Fields] OR "immersiveness"[All Fields]) OR "immersive*"[All Fields] OR ("vis resour"[Journal] OR "proc ieee virtual real conf"[Journal] OR "vr"[All Fields]) OR (("head"[MeSH Terms] OR "head"[All Fields]) AND ("mount"[All Fields] OR "mounted"[All Fields] OR "mounts"[All Fields])) OR (("face"[MeSH Terms] OR "face"[All Fields]) AND ("mount"[All Fields] OR "mounted"[All Fields] OR "mounts"[All Fields])) OR ("caves"[MeSH Terms] OR "caves"[All Fields] OR "cave"[All Fields]))

Search Strategy for CINAHL

( ALL ( older OR senior* OR elder* OR aging OR ageing ) AND ALL ( mental or mental health OR mental disorder* OR mental illness* OR mental disease* OR mood disorder* OR depressi* OR anxiety OR mental wellbeing OR mental well-being OR psychological wellbeing OR psychological well-being OR stress OR distress OR loneliness ) AND ALL ( virtual reality OR virtual-reality OR immerse OR immersive* OR VR OR head mounted OR face mounted OR cave ) )

Search Strategy for Scopus

( ALL ( older OR senior* OR elder* OR aging OR ageing ) AND ALL ( mental or mental health OR mental disorder* OR mental illness* OR mental disease* OR mood disorder* OR depressi* OR anxiety OR mental wellbeing OR mental well-being OR psychological wellbeing OR psychological well-being OR stress OR distress OR loneliness ) AND ALL ( virtual reality OR virtual-reality OR immerse OR immersive* OR VR OR head mounted OR face mounted OR cave ) )

Search strategy for Web of Science

older OR senior* OR elder* OR aging OR ageing (Topic) and mental or mental health OR mental disorder* OR mental illness* OR mental disease* OR mood disorder* OR depressi* OR anxiety OR mental wellbeing OR mental well-being OR psychological wellbeing OR psychological well-being OR stress OR distress OR loneliness (Topic) and virtual reality OR virtual-reality OR immerse OR immersive* OR VR OR head mounted OR face mounted OR cave (Topic)

Search strategy for PsycINFO

abstract(older OR senior* OR elder* OR aging OR ageing ) AND abstract(mental or mental health OR mental disorder* OR mental illness* OR mental disease* OR mood disorder* OR depressi* OR anxiety OR mental wellbeing OR mental well-being OR psychological wellbeing OR psychological well-being OR stress OR distress OR loneliness) AND abstract(virtual reality OR virtual-reality OR immerse OR immersive* OR VR OR head mounted OR face mounted OR cave )

Search strategy for Embase

(older OR senior* OR elder* OR 'aging'/exp OR aging OR 'ageing'/exp OR ageing) AND (mental:ti,ab,kw OR 'mental health':ti,ab,kw OR 'mental disorder*':ti,ab,kw OR 'mental illness*':ti,ab,kw OR 'mental disease*':ti,ab,kw OR 'mood disorder':ti,ab,kw) AND ('virtual reality':ti,ab,kw OR immerse:ti,ab,kw OR immersive*:ti,ab,kw OR vr:ti,ab,kw OR 'head mounted':ti,ab,kw OR 'face mounted':ti,ab,kw OR cave:ti,ab,kw)

Screening results (n=3300)

| 1 | (2013). "Front Matter...Medicine Meets Virtual Reality 20. NextMed/MMVR20 Proceedings." Studies in Health Technology & Informatics 184: i-xxix. | wrong outcomes | |
| --- | --- | --- | --- |
| 2 | (2017). "Erratum: Computer classes and games in virtual reality environment to reduce loneliness among students of an elderly reference center: Study protocol for a randomised cross-over design: Erratum." Medicine (Baltimore) 96(20): e6991. | wrong outcomes | |
| 3 | (2017). Erratum: Computer classes and games in virtual reality environment to reduce loneliness among students of an elderly reference center: Study protocol for a randomised cross-over design: Erratum. Medicine. Baltimore, Maryland, Lippincott Williams & Wilkins. 96: 1-1. | wrong outcomes | |
| 4 | (2017). "Global, regional, and national under-5 mortality, adult mortality, age-specific mortality, and life expectancy, 1970-2016: a systematic analysis for the Global Burden of Disease Study 2016." Lancet 390(10100): 1084-1150. | wrong intervention | |
| 5 | (2018). "Global, regional, and national age-sex-specific mortality for 282 causes of death in 195 countries and territories, 1980-2017: a systematic analysis for the Global Burden of Disease Study 2017." Lancet 392(10159): 1736-1788. | wrong intervention | |
| 6 | (2018). "Prognostic model to predict postoperative acute kidney injury in patients undergoing major gastrointestinal surgery based on a national prospective observational cohort study." BJS Open 2(6): 400-410. | wrong intervention | |
| 7 | (2019). "The Main Predictors of Length of Stay After Total Knee Arthroplasty: Patient-Related or Procedure-Related Risk Factors." J Bone Joint Surg Am 101(12): 1093-1101. | wrong intervention | |
| 8 | (2020). "General Practice and the Community: Research on health service, quality improvements and training. Selected abstracts from the EGPRN Meeting in Vigo, Spain, 17-20 October 2019 Abstracts." EUROPEAN JOURNAL OF GENERAL PRACTICE 26(1): 42-50. | wrong outcomes | |
| 9 | (2020). "Predictors of Successful Treatment 1 Year After Arthroscopic Partial Meniscectomy: Data from the OME Cohort." JB JS Open Access 5(4). | wrong intervention | |
| 10 | (2020). "Supporting people with dementia and their caregivers' everyday occupations through home hazard identification and virtual reality-based training." ALZHEIMERS & DEMENTIA 16. | wrong outcomes | |
| 11 | (2020). "Swerve." ONE EARTH 3(1): 98-99. | wrong intervention | |
| 12 | (2022). "Mapping development and health effects of cooking with solid fuels in low-income and middle-income countries, 2000-18: a geospatial modelling study." Lancet Glob Health 10(10): e1395-e1411. | wrong intervention | |
| 13 | Aakre, J. A., et al. (2023). "Self-reported sleep fragmentation and sleep duration and their association with cognitive function in PROTECT, a large digital community-based cohort of people over 50." INTERNATIONAL JOURNAL OF GERIATRIC PSYCHIATRY 38(11). | wrong intervention | |
| 14 | Aakre, J. A., et al. (2023). "Self‐reported sleep fragmentation and sleep duration and their association with cognitive function in PROTECT, a large digital community‐based cohort of people over 50." International Journal of Geriatric Psychiatry 38(11): 1-10. | wrong intervention | |
| 15 | Aardoom, J. J., et al. (2022). "A Preoperative Virtual Reality App for Patients Scheduled for Cardiac Catheterization: Pre-Post Questionnaire Study Examining Feasibility, Usability, and Acceptability." JMIR Cardio 6(1): e29473. | wrong design | |
| 16 | Abbas, R. L., et al. (2024). "Effect of Adding Virtual Reality Training to Traditional Exercise Program on Pain, Mental Status and Psychological Status in Unilateral Traumatic Lower Limb Amputees: A Randomized Controlled Trial." GAMES FOR HEALTH JOURNAL 13(4): 245-251. | wrong population | |
| 17 | Abdalla, A. I. (2010). "Effect of long-term water aging on microtensile bond strength of self-etch adhesives to dentin." American Journal of Dentistry 23(1): 29-33. | wrong intervention | |
| 18 | Abdalrahim, A., et al. (2024). "Enhancing apathy treatment in Jordanian people living with dementia residing in care homes using virtual reality reminiscence therapy." Working with Older People 28(3): 293-304. | wrong design | |
| 19 | Abdalrahim, A., et al. (2024). "Enhancing apathy treatment in Jordanian people living with dementia residing in care homes using virtual reality reminiscence therapy." Working with Older People: Community Care Policy & Practice 28(3): 293-304. | wrong design | |
| 20 | Abdelaal, F. B., et al. (2014). "Effect of leachate composition on the long-term performance of a HDPE geomembrane." GEOTEXTILES AND GEOMEMBRANES 42(4): 348-362. | wrong intervention | |
| 21 | Abdolhoseinpour, H., et al. (2018). "Isolated Oculomotor and Abducens Nerve Palsies as Initial Presentation of Cavernous Sinus Tuberculoma: Case Report and Literature Review." WORLD NEUROSURGERY 117: 413-418. | wrong outcomes | |
| 22 | Abdul Sani, N. F., et al. (2018). "DNA damage and protein oxidation associated with ageing correlate with cognitive dysfunction in a Malaysian population." Free Radic Res 52(9): 1000-1009. | wrong intervention | |
| 23 | Abe, T., et al. (2021). "Assessing the Impact of a Hilly Environment on Depressive Symptoms among Community-Dwelling Older Adults in Japan: A Cross-Sectional Study." Int J Environ Res Public Health 18(9). | wrong design | |
| 24 | Abichou, K., et al. (2022). "How rich are false memories in a naturalistic context in healthy aging?" Memory 30(3): 262-278. | wrong intervention | |
| 25 | Abichou, K., et al. (2019). "Young and older adults benefit from sleep, but not from active wakefulness for memory consolidation of what-where-when naturalistic events." Frontiers in Aging Neuroscience 11: 16. | wrong intervention | |
| 26 | Abichou, K., et al. (2020). "False memory in normal ageing: empirical data from the DRM paradigm and theoretical perspectives." Geriatr Psychol Neuropsychiatr Vieil 18(1): 65-75. | wrong intervention | |
| 27 | Abichou, K., et al. (2021). "The production of false recognition and the associated state of consciousness following encoding in a naturalistic context in aging." Consciousness and Cognition: An International Journal 90: 17. | wrong intervention | |
| 28 | Abou Ghayda, R., et al. (2022). "The global case fatality rate of coronavirus disease 2019 by continents and national income: A meta-analysis." Journal of Medical Virology 94(6): 2402-2413. | wrong design | |
| 29 | Abou Ghayda, R., et al. (2022). "The global case fatality rate of coronavirus disease 2019 by continents and national income: A meta-analysis." J Med Virol 94(6): 2402-2413. | wrong design | |
| 30 | Abouzeid, C. A., et al. (2022). "Are burns a chronic condition? Examining patient reported outcomes up to 20 years after burn injury-A Burn Model System National Database investigation." J Trauma Acute Care Surg 92(6): 1066-1074. | wrong intervention | |
| 31 | Abouzeid, C. A., et al. (2022). "Are burns a chronic condition? Examining patient reported outcomes up to 20 years after burn injury-A Burn Model System National Database investigation." JOURNAL OF TRAUMA AND ACUTE CARE SURGERY 92(6): 1066-1074. | wrong intervention | |
| 32 | Abozaid, A. and R. Gerlai (2022). "Behavioral Effects of Buspirone in Juvenile Zebrafish of Two Different Genetic Backgrounds." TOXICS 10(1). | wrong design | |
| 33 | Abraham, N. (2020). "Wonder VR: Interactive Storytelling through VR 360 Video with NHS Patients Living with Dementia." CONTEMPORARY THEATRE REVIEW 30(4): 474-+. | wrong outcomes | |
| 34 | Abraham, S., et al. (2022). "Implementing 360-Degree Simulation Training During Psychiatry Placement Inductions: A Mixed Methods Training Evaluation." BJPsych Open 8: S6. | wrong design | |
| 35 | Abu-Rayya, H. M., et al. (2019). "The Psychometric Properties of the Arabic 16-Item Maladaptive Daydreaming Scale (MDS-16-AR) in a Multicountry Arab Sample." PSYCHOLOGY OF CONSCIOUSNESS-THEORY RESEARCH AND PRACTICE 6(2): 171-183. | wrong intervention | |
| 36 | Acharya, V., et al. (2023). "A noninferiority trial on information-based video versus self-selected video distraction technique for preoperative anxiety reduction in school children: Prepare trial." PEDIATRIC ANESTHESIA 33(11): 955-961. | wrong intervention | |
| 37 | Adali, U., et al. (2024). "Influence of sandblasting and bonding on the shear bond strength between differently pigmented polyetheretherketone (PEEK) and veneering composite after artificial aging." Dent Mater 40(8): 1123-1127. | wrong intervention | |
| 38 | Adamo, D. E., et al. (2012). "Age differences in virtual environment and real world path integration." Frontiers in Aging Neuroscience 4: 9. | wrong intervention | |
| 39 | Adams, C. E., et al. (2018). "Monitoring oral health of people in Early Intervention for Psychosis (EIP) teams: The extended Three Shires randomised trial." Int J Nurs Stud 77: 106-114. | wrong intervention | |
| 40 | Adams, H. H., et al. (2016). "Novel genetic loci underlying human intracranial volume identified through genome-wide association." Nat Neurosci 19(12): 1569-1582. | wrong intervention | |
| 41 | Addab, S., et al. (2022). "Use of virtual reality in managing paediatric procedural pain and anxiety: An integrative literature review." JOURNAL OF CLINICAL NURSING 31(21-22): 3032-3059. | wrong outcomes | |
| 42 | Aditya, P. V. A., et al. (2021). "Comparison of effectiveness of three distraction techniques to allay dental anxiety during inferior alveolar nerve block in children: A randomized controlled clinical trial." HELIYON 7(9). | wrong population | |
| 43 | Adjei-Banuah, N. Y., et al. (2024). "Factors Influencing Primary Care Access for Common Mental Health Conditions Among Adults in West Africa: Protocol for a Scoping Review." JMIR Res Protoc 13: e58890. | wrong outcomes | |
| 44 | Admiraal, R., et al. (2017). "Viral reactivations and associated outcomes in the context of immune reconstitution after pediatric hematopoietic cell transplantation." JOURNAL OF ALLERGY AND CLINICAL IMMUNOLOGY 140(6): 1643-+. | wrong population | |
| 45 | Afifi, T., et al. (2022). "Using virtual reality to improve the quality of life of older adults with cognitive impairments and their family members who live at a distance." Health Communication. | wrong design | |
| 46 | Afifi, T., et al. (2023). "Using Virtual Reality to Improve the Quality of Life of Older Adults with Cognitive Impairments and their Family Members who Live at a Distance." HEALTH COMMUNICATION 38(9): 1904-1915. | wrong design | |
| 47 | Agam, A., et al. (2020). "Flint Type Analysis of Bifaces From Acheulo-Yabrudian Qesem Cave (Israel) Suggests an Older Acheulian Origin." JOURNAL OF PALEOLITHIC ARCHAEOLOGY 3(4): 719-754. | wrong intervention | |
| 48 | Aganov, S., et al. (2022). "Pure purr virtual reality technology: measuring heart rate variability and anxiety levels in healthy volunteers affected by moderate stress." ARCHIVES OF MEDICAL SCIENCE 18(2): 336-343. | wrong population | |
| 49 | Agbangla, N. F., et al. (2022). "Snacktivity™ , Giant Games and Immersive Virtual Reality Exercises: A Rapid Narrative Review of These New Physical Activity Practices among Older People Living in Nursing Homes and Long-Term Care Facilities." HEALTHCARE 10(10). | wrong outcomes | |
| 50 | Aggarwal, N., et al. (2022). "Impact of COVID-19 on Structure and Function of Program of All-Inclusive Care for the Elderly (PACE) Sites in North Carolina." JOURNAL OF THE AMERICAN MEDICAL DIRECTORS ASSOCIATION 23(7): 1109-+. | wrong intervention | |
| 51 | Agli, O., et al. (2024). "La réalité virtuelle au service du bien-être: Étude pilote." NPG Neurologie - Psychiatrie - Gériatrie 24(140): 89-95. | wrong design | |
| 52 | Ahmad, K., et al. (2020). "Using Cognitive Task Analysis to train Orthopaedic Surgeons - Is it time to think differently? A systematic review." ANNALS OF MEDICINE AND SURGERY 59: 131-137. | wrong outcomes | |
| 53 | Ahmad, M., et al. (2020). "Virtual reality technology for pain and anxiety management among patients with cancer: A systematic review." Pain Management Nursing 21(6): 601-607. | wrong outcomes | |
| 54 | Ahmad, M., et al. (2020). "Virtual Reality Technology for Pain and Anxiety Management among Patients with Cancer: A Systematic Review." PAIN MANAGEMENT NURSING 21(6): 601-607. | wrong population | |
| 55 | Ahmadpour, N., et al. (2024). "Building enriching realities with children: Creating makerspaces that intertwine virtual and physical worlds in pediatric hospitals." INTERNATIONAL JOURNAL OF HUMAN-COMPUTER STUDIES 183. | wrong design | |
| 56 | Ahmadpour, N., et al. (2020). "Synthesizing Multiple Stakeholder Perspectives on Using Virtual Reality to Improve the Periprocedural Experience in Children and Adolescents: Survey Study." JOURNAL OF MEDICAL INTERNET RESEARCH 22(7). | wrong outcomes | |
| 57 | Ahmed, B., et al. (2018). Treatment of Alzheimer's, Cognitive, Chronic Pain Rehabilitation, Depression and Anxiety disorders in One System for Elderly Using VR. 2018 15TH INTERNATIONAL CONFERENCE ON UBIQUITOUS ROBOTS (UR): 483-488. | wrong intervention | |
| 58 | Ahn, B., et al. (2019). "Mitochondrial oxidative stress impairs contractile function but paradoxically increases muscle mass via fibre branching." J Cachexia Sarcopenia Muscle 10(2): 411-428. | wrong intervention | |
| 59 | Ahonle, Z. J., et al. (2020). "State-Federal Vocational Rehabilitation in Traumatic Brain Injury: What Predictors Are Associated With Employment Outcomes?" REHABILITATION COUNSELING BULLETIN 63(3): 143-155. | wrong population | |
| 60 | Ahrens, A. P., et al. (2021). "A Six-Day, Lifestyle-Based Immersion Program Mitigates Cardiovascular Risk Factors and Induces Shifts in Gut Microbiota, Specifically Lachnospiraceae, Ruminococcaceae, Faecalibacterium prausnitzii: A Pilot Study." Nutrients 13(10). | wrong population | |
| 61 | Ahuja, A., et al. (2023). "Effect of bubble CPAP (continuous positive airway pressure) on a newborn presenting with respiratory distress." RAWAL MEDICAL JOURNAL 48(2): 382-385. | wrong population | |
| 62 | Akan, I., et al. (2024). "Animated video reduces pain and anxiety during pin removal in children with supracondylar humerus fractures: a randomized controlled trial." ARCHIVES OF ORTHOPAEDIC AND TRAUMA SURGERY. | wrong population | |
| 63 | Akarsu, Ö., et al. (2023). "The Effect of 2 Different Distraction Methods on Pain, Fear, and Anxiety Levels During Venous Blood Draw in Children in a Pediatric Emergency Unit." JOURNAL OF NURSING CARE QUALITY 38(4): E51-E58. | wrong population | |
| 64 | Akderya, T., et al. (2024). "Effect of acidic environment on absorptive, morphological, mechanical, structural, and thermal properties of glass-carbon/epoxy-based hybrid composites." JOURNAL OF POLYMER RESEARCH 31(2). | wrong population | |
| 65 | Akin, H. (2015). "Magnetic Flux Density of Different Types of New Generation Magnetic Attachment Systems." J Prosthodont 24(5): 414-418. | wrong population | |
| 66 | Akinola, O. A. and C. T. Doabler (2022). "Determinants of employment outcomes of transition-age youth with depressive disorders." JOURNAL OF VOCATIONAL REHABILITATION 56(1): 55-68. | wrong population | |
| 67 | Akkara, F. J., et al. (2022). "The Effect of Micro-Alloying and Surface Finishes on the Thermal Cycling Reliability of Doped SAC Solder Alloys." Materials (Basel) 15(19). | wrong population | |
| 68 | Aksnes, M., et al. (2021). "Associations of cerebrospinal fluid amyloidogenic nanoplaques with cytokines in Alzheimer's disease." Transl Neurodegener 10(1): 18. | wrong intervention | |
| 69 | Al Dweik, R., et al. (2024). "Opportunities and challenges in leveraging digital technology for mental health system strengthening: a systematic review to inform interventions in the United Arab Emirates." BMC Public Health 24(1): 2592. | wrong outcomes | |
| 70 | Alabduljabbar, R., et al. (2023). "An Interactive Augmented and Virtual Reality System for Managing Dental Anxiety among Young Patients: A Pilot Study." APPLIED SCIENCES-BASEL 13(9). | wrong population | |
| 71 | Alahmari, K., et al. (2023). "Outcomes of virtual reality technology in the management of generalised anxiety disorder: a systematic review and meta-analysis." BEHAVIOUR & INFORMATION TECHNOLOGY 42(14): 2353-2365. | wrong outcomes | |
| 72 | Alam, R., et al. (2022). "Differential changes in self-reported quality of life in elderly populations after diagnosis of a genitourinary malignancy." UROLOGIC ONCOLOGY-SEMINARS AND ORIGINAL INVESTIGATIONS 40(10). | wrong intervention | |
| 73 | Alanazi, M. O., et al. (2023). "Nature-Based Virtual Reality Feasibility and Acceptability Pilot for Caregiver Respite." CURRENT ONCOLOGY 30(7): 5995-6005. | wrong design | |
| 74 | Alashram, A. R., et al. (2022). "Rehabilitation interventions for cognitive deficits in stroke survivors: A systematic review of randomized controlled trials." APPLIED NEUROPSYCHOLOGY-ADULT. | wrong outcomes | |
| 75 | Albano, A. M., et al. (2018). "VIRTUAL REALITY AND SOCIAL ANXIETY IN COLLEGE-AGED YOUNG ADULTS: IMPLICATIONS FOR ENHANCING DIAGNOSTIC PRECISION AND CBT." JOURNAL OF THE AMERICAN ACADEMY OF CHILD AND ADOLESCENT PSYCHIATRY 57(10): S297-S298. | wrong population | |
| 76 | Aldouby, H. (2020). "Fragile Traces, Treacherous Sands: Ronen Sharabani and Micha Ullman's Intergenerational Encounter." ARTS 9(2). | wrong population | |
| 77 | Aleo, A., et al. (2024). "A multi-analytical approach reveals flexible compound adhesive technology at Steenbokfontein Cave, Western Cape." JOURNAL OF ARCHAEOLOGICAL SCIENCE 167. | wrong population | |
| 78 | Alexander, K., et al. (2024). "Caregiving While Black: A Novel, Online Culturally Tailored Psychoeducation Course for Black Dementia Caregivers." The Gerontologist 64(6). | wrong intervention | |
| 79 | Alexander, Y. P., et al. (2016). Influence of the modern Web communication on the psychological characteristics of the rising generation (12-13 year old) from the view of the Information Images Theory. 7TH ANNUAL INTERNATIONAL CONFERENCE ON BIOLOGICALLY INSPIRED COGNITIVE ARCHITECTURES, (BICA 2016). 88: 423-428. | wrong outcomes | |
| 80 | Alexandraki, K. I., et al. (2000). Adrenal Insufficiency. Endotext. K. R. Feingold, B. Anawalt, M. R. Blackman et al. South Dartmouth (MA), MDText.com, Inc. | wrong outcomes | |
| 81 | Alfaifi, A. A. and A. U. Althemery (2022). "Sociodemographic characteristics and health-related quality of life of individuals undergoing antidepressant therapy." SCIENTIFIC REPORTS 12(1). | wrong intervention | |
| 82 | Alhirsan, S. M., et al. (2023). "The Immediate Effects of Different Types of Augmented Feedback on Fast Walking Speed Performance and Intrinsic Motivation After Stroke." ARCHIVES OF REHABILITATION RESEARCH AND CLINICAL TRANSLATION 5(2). | wrong population | |
| 83 | Ali Ismail, A. M. (2022). "Virtual-reality rehabilitation for inpatient elderly leukemic patients: psychological and physical roles during the fourth COVID-19 wave." Family Medicine & Primary Care Review 24(2): 185-186. | wrong design | |
| 84 | Ali, M. and R. K. Rowe (2024). "Long-term performance of HDPE extrusion Welds aged at 85°C in synthetic leachate." GEOSYNTHETICS INTERNATIONAL. | wrong population | |
| 85 | Ali, S., et al. (2022). "Virtual reality-based distraction for intravenous insertion-related distress in children: a study protocol for a randomised controlled trial." BMJ OPEN 12(3). | wrong outcomes | |
| 86 | Allahham, A., et al. (2024). "The impact of burn injury on the central nervous system." Burns Trauma 12: tkad037. | wrong intervention | |
| 87 | Allain, P., et al. (2014). "Detecting everyday action deficits in Alzheimer's disease using a nonimmersive virtual reality kitchen." Journal of the International Neuropsychological Society 20(5): 468-477. | wrong outcomes | |
| 88 | Allen, J., et al. (2018). "The sensitivity of the MOS SF-12 and PROMISA® global summary scores to adverse health events in an older cohort." QUALITY OF LIFE RESEARCH 27(8): 2207-2215. | wrong intervention | |
| 89 | Allison, S. and D. Head (2017). "Route repetition and route reversal: Effects of age and encoding method." Psychol Aging 32(3): 220-231. | wrong intervention | |
| 90 | Almajed, O. S., et al. (2023). "The Effectiveness of Virtual Reality in Controlling Pain and Anxiety Levels in Four-to-Six-Year-Old Children During Dental Treatment." CUREUS JOURNAL OF MEDICAL SCIENCE 15(12). | wrong population | |
| 91 | Almajid, R., et al. (2021). "Effects of wearing a head-mounted display during a standard clinical test of dynamic balance." Gait and Posture 85: 78-83. | wrong intervention | |
| 92 | Almajid, R., et al. (2020). "Visual dependence affects the motor behavior of older adults during the Timed Up and Go (TUG) test." Archives of Gerontology and Geriatrics 87: 7. | wrong design | |
| 93 | Almarzouq, S., et al. (2024). "Effectiveness of Nonpharmacological Behavioural Interventions in Managing Dental Fear and Anxiety among Children: A Systematic Review and Meta-Analysis." HEALTHCARE 12(5). | wrong population | |
| 94 | Almudhi, A. (2022). "Evaluating adaptation effect in real versus virtual reality environments with people who stutter." EXPERT REVIEW OF MEDICAL DEVICES 19(1): 75-81. | wrong population | |
| 95 | Alonso-Pérez, R., et al. (2021). "Original versus nonoriginal cast-to-gold abutment-implant connection: Analysis of the internal fit and long-term fatigue performance." J Prosthet Dent 126(1): 94.e91-94.e99. | wrong intervention | |
| 96 | Alqarni, M. A., et al. (2019). "Microleakage of Aesthetic Restorations Following Functional Simulation and Immersion in Saudi-Traditional Mouth Rinses." Open Access Maced J Med Sci 7(21): 3630-3633. | wrong intervention | |
| 97 | Alrimy, T., et al. (2023). "Desktop Virtual Reality Offers a Novel Approach to Minimize Pain and Anxiety during Burn Wound Cleaning/Debridement in Infants and Young Children: A Randomized Crossover Pilot Study." JOURNAL OF CLINICAL MEDICINE 12(15). | wrong population | |
| 98 | Alrimy, T., et al. (2022). "Virtual Reality Animal Rescue World: Pediatric virtual reality analgesia during just noticeable pressure pain in children aged 2-10 years old (crossover design)." FRONTIERS IN PSYCHOLOGY 13. | wrong population | |
| 99 | Alshamrani, K. A., et al. (2024). "Assistive technology services for adults with disabilities in state-federal vocational rehabilitation programs." DISABILITY AND REHABILITATION-ASSISTIVE TECHNOLOGY 19(4): 1382-1391. | wrong design | |
| 100 | Al-Sharqi, L., et al. (2015). GENDER COMPARISON ON PERCEPTIONS OF SOCIAL MEDIA IMPACT ON STUDENTS' SOCIAL BEHAVIOR. EDULEARN15: 7TH INTERNATIONAL CONFERENCE ON EDUCATION AND NEW LEARNING TECHNOLOGIES: 1428-1437. | wrong outcomes | |
| 101 | Alshatrat, S. M., et al. (2022). "Effect of immersive virtual reality on pain in different dental procedures in children: A pilot study." INTERNATIONAL JOURNAL OF PAEDIATRIC DENTISTRY 32(2): 264-272. | wrong population | |
| 102 | Altena, E., et al. (2023). "Vestibular symptoms are related to the proportion of REM sleep in people with sleep complaints: A preliminary report." Journal of Vestibular Research: Equilibrium & Orientation 33(3): 165-172. | wrong design | |
| 103 | Altena, E., et al. (2019). "How sleep problems contribute to simulator sickness: Preliminary results from a realistic driving scenario." JOURNAL OF SLEEP RESEARCH 28(2). | wrong design | |
| 104 | Althumairi, A., et al. (2021). "Virtual Reality: Is It Helping Children Cope with Fear and Pain During Vaccination?" JOURNAL OF MULTIDISCIPLINARY HEALTHCARE 14: 2625-2632. | wrong population | |
| 105 | Altman, D. E., et al. (2018). "Developmental differences in stress responding after repeated underwater trauma exposures in rats." Stress 21(3): 267-273. | wrong population | |
| 106 | Alvarado, J. C., et al. (2020). "Antioxidants and Vasodilators for the Treatment of Noise-Induced Hearing Loss: Are They Really Effective?" FRONTIERS IN CELLULAR NEUROSCIENCE 14. | wrong intervention | |
| 107 | Alves, R., et al. (2016). Serious Games for the Cognitive Stimulation of Adults: A Proposal of a Pilot Project. SERIOUS GAMES, INTERACTION, AND SIMULATION, SGAMES 2015. 161: 36-41. | wrong design | |
| 108 | Amaefule, C. O., et al. (2020). "Effect of spatial disorientation in a virtual environment on gait and vital features in patients with dementia: Pilot single-blind randomized control trial." JMIR Serious Games 8(4): 13. | wrong outcomes | |
| 109 | Amaefule, C. O., et al. (2020). "Effect of Spatial Disorientation in a Virtual Environment on Gait and Vital Features in Patients with Dementia: Pilot Single-Blind Randomized Control Trial." JMIR SERIOUS GAMES 8(3). | wrong outcomes | |
| 110 | Amali, R. J. and S. S. Chavan (2023). "Effectiveness of Virtual Reality Distraction on Pain Perception and Fear among Children with Cancer Undergoing IV Cannulation." INDIAN JOURNAL OF COMMUNITY MEDICINE 48(6): 909-914. | wrong population | |
| 111 | Amaral, C., et al. (2018). "A Feasibility Clinical Trial to Improve Social Attention in Autistic Spectrum Disorder (ASD) Using a Brain Computer Interface." FRONTIERS IN NEUROSCIENCE 12. | wrong design | |
| 112 | Ames, H., et al. (2017). "Does functional motor incomplete (AIS D) spinal cord injury confer unanticipated challenges?" Rehabilitation Psychology 62(3): 401-406. | wrong intervention | |
| 113 | Amick, M. M., et al. (2013). "Driving simulator performance of Veterans from the Iraq and Afghanistan wars." JOURNAL OF REHABILITATION RESEARCH AND DEVELOPMENT 50(4): 463-470. | wrong intervention | |
| 114 | Amsalem, D., et al. (2023). "Anxiety and Depression Symptoms Among Young U.S. Essential Workers During the COVID-19 Pandemic." Psychiatr Serv 74(10): 1010-1018. | wrong population | |
| 115 | Amziane, S. and A. Perrot (2017). "The plate test carried out on fresh cement-based materials: How and why?" CEMENT AND CONCRETE RESEARCH 93: 1-7. | wrong population | |
| 116 | Anand, P. and T. C. Honeycutt (2020). "Long-Term Outcomes for Transition-Age Youth With Mental Health Conditions Who Receive Postsecondary Education Support." JOURNAL OF DISABILITY POLICY STUDIES 30(4): 223-232. | wrong population | |
| 117 | Anand, S. (2016). "Movies in Mind:Star Wars The consciousness ‘awakens’." Australian and New Zealand Journal of Psychiatry 50(8): 812. | wrong population | |
| 118 | Anandan, D., et al. (2018). "Development of mechanically compliant 3D composite scaffolds for bone tissue engineering applications." J Biomed Mater Res A 106(12): 3267-3274. | wrong population | |
| 119 | Anandhanarayanan, A., et al. (2000). Diabetic Neuropathies. Endotext. K. R. Feingold, B. Anawalt, M. R. Blackman et al. South Dartmouth (MA), MDText.com, Inc. | wrong outcomes | |
| 120 | Anastasiadou, Z., et al. (2024). "Design and Evaluation of a Memory-Recalling Virtual Reality Application for Elderly Users." MULTIMODAL TECHNOLOGIES AND INTERACTION 8(3). | wrong design | |
| 121 | Anchala, K., et al. (2024). "Efficacy of kaleidoscope, virtual reality, and video games to alleviate dental anxiety during local anesthesia in children: a randomized clinical trial." JOURNAL OF DENTAL ANESTHESIA AND PAIN MEDICINE 24(3): 195-204. | wrong population | |
| 122 | Anderson, A. P., et al. (2017). "Relaxation with Immersive Natural Scenes Presented Using Virtual Reality." AEROSPACE MEDICINE AND HUMAN PERFORMANCE 88(6): 520-526. | wrong design | |
| 123 | Anderson, J. D. and R. O. Pitner (2021). "They Are Coming Home: The Effect of Trauma-Related Cognitions on Vocational Readiness of Incarcerated Women." JOURNAL OF INTERPERSONAL VIOLENCE 36(13-14): 6227-6246. | wrong design | |
| 124 | Anderson, K. A. (2019). "The virtual care farm: A preliminary evaluation of an innovative approach to addressing loneliness and building community through nature and technology." Activities, Adaptation & Aging 43(4): 334-344. | wrong design | |
| 125 | Anderson, P. L., et al. (2017). "Virtual Reality and Exposure Group Therapy for Social Anxiety Disorder: Results from a 4-6 Year Follow-Up." COGNITIVE THERAPY AND RESEARCH 41(2): 230-236. | wrong outcomes | |
| 126 | Anderson, P. L., et al. (2013). "Virtual Reality Exposure Therapy for Social Anxiety Disorder: A Randomized Controlled Trial." JOURNAL OF CONSULTING AND CLINICAL PSYCHOLOGY 81(5): 751-760. | wrong population | |
| 127 | Anderson, S., et al. (2023). "Mindfulness training is associated with improved quality of life in female collegiate athletes." JOURNAL OF AMERICAN COLLEGE HEALTH. | wrong population | |
| 128 | Anderson, W. K. Z. and L. A. Davis (2023). "White capitalism within communities of craftivism: mask making and health maintenance disparities during COVID-19." FRONTIERS IN COMMUNICATION 8. | wrong population | |
| 129 | Anderson, Y. C., et al. (2017). "Assessment of health-related quality of life and psychological well-being of children and adolescents with obesity enrolled in a New Zealand community-based intervention programme: An observational study." BMJ Open 7(8). | wrong population | |
| 130 | Anderson-Hanley, C., et al. (2018). "The Aerobic and Cognitive Exercise Study (ACES) for community-dwelling older adults with or at-risk for mild cognitive impairment (MCI): Neuropsychological, neurobiological and neuroimaging outcomes of a randomized clinical trial." Frontiers in Aging Neuroscience 10: 25. | wrong intervention | |
| 131 | Anderson-Hanley, C., et al. (2011). "Social facilitation in virtual reality-enhanced exercise: competitiveness moderates exercise effort of older adults." Clin Interv Aging 6: 275-280. | wrong outcomes | |
| 132 | Andréasson, C., et al. (2019). "Mitochondria orchestrate proteostatic and metabolic stress responses." EMBO Rep 20(10): e47865. | wrong intervention | |
| 133 | Andrew, J. J., et al. (2020). "Effect of Intra-Ply Hybrid Patches and Hydrothermal Aging on Local Bending Response of Repaired GFRP Composite Laminates." Molecules 25(10). | wrong intervention | |
| 134 | Androulakis, II, et al. (2000). Pseudo-Cushing’s States. Endotext. K. R. Feingold, B. Anawalt, M. R. Blackman et al. South Dartmouth (MA), MDText.com, Inc. | wrong outcomes | |
| 135 | Ang, C. S., et al. (2023). "Editorial for advances in human-centred dementia technology." INTERNATIONAL JOURNAL OF HUMAN-COMPUTER STUDIES 170. | wrong design | |
| 136 | Antal, H. and S. Bhutani (2023). "Identifying Linkages Between Climate Change, Urbanisation, and Population Ageing for Understanding Vulnerability and Risk to Older People: A Review." AGEING INTERNATIONAL 48(3): 816-839. | wrong design | |
| 137 | Antoniou, P., et al. (2015). DISCOVER-ING OPENSIM. DESIGN GUIDELINES AND IMPLEMENTATION OF SCENARIO BASED LEARNING FOR CARERS OF THE ELDERLY. EDULEARN15: 7TH INTERNATIONAL CONFERENCE ON EDUCATION AND NEW LEARNING TECHNOLOGIES: 7006-7014. | wrong design | |
| 138 | Anttila, V., et al. (2018). "Analysis of shared heritability in common disorders of the brain." Science 360(6395). | wrong population | |
| 139 | Antunes, T. P., et al. (2017). "Computer classes and games in virtual reality environment to reduce loneliness among students of an elderly reference center: Study protocol for a randomised cross-over design(vol 96, e5954, 2017)." MEDICINE 96(20). | wrong population | |
| 140 | Antunes, T. P. C., et al. (2017). "Computer classes and games in virtual reality environment to reduce loneliness among students of an elderly reference center Study protocol for a randomised cross-over design." MEDICINE 96(10). | wrong design | |
| 141 | Antunes, T. P. C., et al. (2017). "Computer classes and games in virtual reality environment to reduce loneliness among students of an elderly reference center: Study protocol for a randomised cross-over design." Medicine (Baltimore) 96(10): e5954. | wrong design | |
| 142 | Apostolidis, H., et al. (2024). Work in Progress: STAYinBowling, Sensor Based Training for Athletes and Youngsters in Bowling. SMART MOBILE COMMUNICATION & ARTIFICIAL INTELLIGENCE, VOL 1, IMCL 2023. 936: 188-194. | wrong population | |
| 143 | Appel, L., et al. (2019). "Older Adults With Cognitive and/or Physical Impairments Can Benefit From Immersive Virtual Reality Experiences: A Feasibility Study." Front Med (Lausanne) 6: 329. | wrong design | |
| 144 | Appel, L., et al. (2020). "Older Adults With Cognitive and/or Physical Impairments Can Benefit From Immersive Virtual Reality Experiences: A Feasibility Study." Frontiers in Medicine 6. | wrong design | |
| 145 | Appel, L., et al. (2022). "Virtual Reality for Veteran Relaxation: Can VR Therapy Help Veterans Living With Dementia Who Exhibit Responsive Behaviors?" Frontiers in Virtual Reality 2. | wrong design | |
| 146 | Appel, L., et al. (2021). "Virtual Reality for Veteran Relaxation (VR<SUP>2</SUP>) - Introducing VR-Therapy for Veterans With Dementia - Challenges and Rewards of the Therapists Behind the Scenes." FRONTIERS IN VIRTUAL REALITY 2. | wrong design | |
| 147 | Appel, L., et al. (2021). "Vrct: Randomized controlled trial evaluating the impact of virtual reality-therapy on BPSD and QOL of acute care in-patients with dementia." Journal of Prevention of Alzheimer's Disease 8(SUPPL 1): S158. | wrong outcomes | |
| 148 | Appenzeller, T. (2013). "Old masters: The earliest known cave paintings fuel arguments about whether Neanderthals were the mental equals of modern humans." Nature 497(7449): 302-304. | wrong intervention | |
| 149 | Applegate, M. E., et al. (2018). "Determining Physiological and Psychological Predictors of Time to Task Failure on a Virtual Reality Sorensen Test in Participants With and Without Recurrent Low Back Pain: Exploratory Study." JMIR SERIOUS GAMES 6(3). | wrong design | |
| 150 | Arachchige, S., et al. (2023). "Physiological and Subjective Measures of Anxiety with Repeated Exposure to Virtual Construction Sites at Different Heights." SAFETY AND HEALTH AT WORK 14(3): 303-308. | wrong intervention | |
| 151 | Aram, K., et al. (2017). "Walking in fully immersive virtual environments: an evaluation of potential adverse effects in older adults and individuals with Parkinson's disease." Journal of NeuroEngineering & Rehabilitation (JNER) 14(1): 1-12. | wrong design | |
| 152 | Arczewska-Wlosek, A., et al. (2018). "Effect of Dietary Crude Protein Level and Supplemental Herbal Extract Blend on Selected Blood Variables in Broiler Chickens Vaccinated against Coccidiosis." ANIMALS 8(11). | wrong population | |
| 153 | Areces, D., et al. (2021). "The Influence of State and Trait Anxiety on the Achievement of a Virtual Reality Continuous Performance Test in Children and Adolescents with ADHD Symptoms." JOURNAL OF CLINICAL MEDICINE 10(12). | wrong population | |
| 154 | Arencibia, A. D., et al. (2016). "Tolerance to heavy metal stress in seedlings of three pine species from contrasting environmental conditions in Chile." IFOREST-BIOGEOSCIENCES AND FORESTRY 9: 937-945. | wrong population | |
| 155 | Argandar, G. D., et al. (2016). "Measuring situations that stress Mexicans while driving." TRANSPORTATION RESEARCH PART F-TRAFFIC PSYCHOLOGY AND BEHAVIOUR 37: 154-161. | wrong population | |
| 156 | Arlati, S., et al. (2021). "Acceptance and usability of immersive virtual reality in older adults with objective and subjective cognitive decline." Journal of Alzheimer's Disease 80(3): 1025-1038. | Wrong design | |
| 157 | Arlati, S., et al. (2018). A Virtual Reality-Based Physical and Cognitive Training System Aimed at Preventing Symptoms of Dementia. WIRELESS MOBILE COMMUNICATION AND HEALTHCARE. 247: 117-125. | wrong outcomes | |
| 158 | Arlati, S., et al. (2017). Virtual Environments for Cognitive and Physical Training in Elderly with Mild Cognitive Impairment: A Pilot Study. AUGMENTED REALITY, VIRTUAL REALITY, AND COMPUTER GRAPHICS, AVR 2017, PT II. 10325: 86-106. | wrong design | |
| 159 | Arokina, N. K., et al. (2022). "THE INFLUENCE OF VESSEL POLYPEPTIDE COMPLEX ON THE VIABILITY OF RATS UNDER COLD STRESS." YAKUT MEDICAL JOURNAL(2): 46-48. | wrong population | |
| 160 | Arquissandas, P., et al. (2023). "Moving from VR into AR using bio-cybernetic loops and physiological sensory devices for intervention on anxiety disorders." VIRTUAL REALITY 27(1): 233-243. | wrong population | |
| 161 | Arruda, A. G., et al. (2022). "Reliability of water-based medium-expansion foam as a depopulation method for nursery pigs and cull sows." TRANSBOUNDARY AND EMERGING DISEASES 69(5): E2719-E2730. | wrong population | |
| 162 | Arsenault-Lapierre, G., et al. (2011). "Mild cognitive impairment subcategories depend on the source of norms." Journal of Clinical and Experimental Neuropsychology 33(5): 596-603. | wrong population | |
| 163 | Arshad, H., et al. (2021). "Effect of Brain Training Game on Mild Cognitive Impairment (MCI) in Older Adults." PAKISTAN JOURNAL OF MEDICAL & HEALTH SCIENCES 15(9): 2272-2275. | wrong design | |
| 164 | Arts, E., et al. (2022). "Improving social emotional functioning in adolescents with Developmental Language Disorders: A mini review and recommendations." FRONTIERS IN PSYCHIATRY 13. | wrong design | |
| 165 | Aruanno, B. and F. Garzotto (2019). "MemHolo: mixed reality experiences for subjects with Alzheimer’s disease." Multimedia Tools and Applications 78(10): 13517-13537. | wrong outcomes | |
| 166 | Arvind Pala, P., et al. (2014). "Everyday-like memory and its cognitive correlates in healthy older adults and in young patients with traumatic brain injury: a pilot study based on virtual reality." Disabil Rehabil Assist Technol 9(6): 463-473. | wrong outcomes | |
| 167 | Asbee, J. and T. D. Parsons (2021). "Exploratory Factor Analysis of the Virtual Reality Stroop Task." ANNUAL REVIEW OF CYBERTHERAPY AND TELEMEDICINE 19: 61-65. | wrong design | |
| 168 | Ascione, M., et al. (2024). "Improving Anorexia Nervosa Treatment with Virtual Reality Body Exposure and Attentional Bias Modification: A Single Case Study." APPLIED SCIENCES-BASEL 14(11). | wrong design | |
| 169 | Ascone, L., et al. (2017). "The effect of unfavourable and favourable social comparisons on paranoid ideation: An experimental study." JOURNAL OF BEHAVIOR THERAPY AND EXPERIMENTAL PSYCHIATRY 56: 97-105. | wrong population | |
| 170 | Asfour, L., et al. (2000). Male Androgenetic Alopecia. Endotext. K. R. Feingold, B. Anawalt, M. R. Blackman et al. South Dartmouth (MA), MDText.com, Inc. | wrong outcomes | |
| 171 | Ashkenazi, H., et al. (2021). "MICROHISTORY IN ARCHAEOLOGY AND ITS CONTRIBUTION TO THE ARCHAEOLOGICAL RESEARCH The Burial from "The Cave of the Warrior" as a Test Case." JOURNAL OF EASTERN MEDITERRANEAN ARCHAEOLOGY AND HERITAGE STUDIES 9(4): 376-394. | wrong population | |
| 172 | Ashmore, J., et al. (2019). "A Free Virtual Reality Experience to Prepare Pediatric Patients for Magnetic Resonance Imaging: Cross-Sectional Questionnaire Study." JMIR PEDIATRICS AND PARENTING 2(1). | wrong population | |
| 173 | Asiri, S., et al. (2022). "The effectiveness of using virtual reality technology for perioperative anxiety among adults undergoing elective surgery: a randomised controlled trial protocol." TRIALS 23(1). | wrong design | |
| 174 | Astasio-Picado, A., et al. (2022). "Efficacy of Interventions Based on the Use of Information and Communication Technologies for the Promotion of Active Aging." INTERNATIONAL JOURNAL OF ENVIRONMENTAL RESEARCH AND PUBLIC HEALTH 19(3). | wrong design | |
| 175 | Astasio-Picado, Á., et al. (2022). "Efficacy of Interventions Based on the Use of Information and Communication Technologies for the Promotion of Active Aging." Int J Environ Res Public Health 19(3). | wrong design | |
| 176 | Asvanund, Y., et al. (2015). "Effect of audiovisual eyeglasses during local anesthesia injections in 5-to 8-year-old children." QUINTESSENCE INTERNATIONAL 46(6): 513-521. | wrong population | |
| 177 | Athanasiou, A., et al. (2022). "Neurorehabilitation Through Synergistic Man-Machine Interfaces Promoting Dormant Neuroplasticity in Spinal Cord Injury: Protocol for a Nonrandomized Controlled Trial." JMIR RESEARCH PROTOCOLS 11(9). | wrong design | |
| 178 | Athar, F., et al. (2024). Limited Cell-Autonomous Anticancer Mechanisms in Long-Lived Bats. | wrong population | |
| 179 | Atkins, A. S., et al. (2018). "Assessment of Instrumental Activities of Daily Living in Older Adults with Subjective Cognitive Decline Using the Virtual Reality Functional Capacity Assessment Tool (VRFCAT)." J Prev Alzheimers Dis 5(4): 216-234. | wrong design | |
| 180 | Atzori, B., et al. (2018). "Virtual Reality Analgesia for Pediatric Dental Patients." FRONTIERS IN PSYCHOLOGY 9. | wrong population | |
| 181 | Atzori, B., et al. (2022). "An Exploratory Study on the Effectiveness of Virtual Reality Analgesia for Children and Adolescents with Kidney Diseases Undergoing Venipuncture." INTERNATIONAL JOURNAL OF ENVIRONMENTAL RESEARCH AND PUBLIC HEALTH 19(4). | wrong population | |
| 182 | Austin, B. S., et al. (2019). "Vocational Rehabilitation Outcomes for Individuals with Intellectual Disabilities and Co-Occurring Psychiatric Disorders." JOURNAL OF REHABILITATION 85(4): 14-23. | wrong intervention | |
| 183 | Au-Yeung, A., et al. (2023). "Exploring the feasibility of a mental health application (JoyPop(TM)) for Indigenous youth." Front Psychiatry 14: 1269347. | wrong population | |
| 184 | Averbach, J. and J. Monin (2022). "Impact of a Virtual Art Tour Intervention on the Emotional Well-Being of Older Adults." Gerontologist 62(10): 1496-1506. | wrong intervention | |
| 185 | Avola, D., et al. (2019). "An interactive and low-cost full body rehabilitation framework based on 3D immersive serious games." Journal of Biomedical Informatics 89: 81-100. | wrong design | |
| 186 | Awan, N., et al. (2021). "Evaluating the Cross-Sectional and Longitudinal Relationships Predicting Suicidal Ideation Following Traumatic Brain Injury." J Head Trauma Rehabil 36(1): E18-e29. | wrong design | |
| 187 | Ayme, K., et al. (2014). "Effect of head-out water immersion on vascular function in healthy subjects." APPLIED PHYSIOLOGY NUTRITION AND METABOLISM 39(4): 425-431. | wrong intervention | |
| 188 | Aziz, M., et al. (2020). "Audiovisual Distraction: A Pricking Pain Reduction Modality among Ladies Receiving Intraoral Injections." JCPSP-JOURNAL OF THE COLLEGE OF PHYSICIANS AND SURGEONS PAKISTAN 30(1): 4-8. | wrong population | |
| 189 | Azizoddin, D. R., et al. (2021). "Chronic pain severity, impact, and opioid use among patients with cancer: An analysis of biopsychosocial factors using the CHOIR learning health care system." Cancer 127(17): 3254-3263. | wrong design | |
| 190 | Babbage, C., et al. (2018). "Desired Features of a Digital Technology Tool for Self-Management of Well-Being in a Nonclinical Sample of Young People: Qualitative Study." JMIR MENTAL HEALTH 5(4). | wrong design | |
| 191 | Badjate, D., et al. (2021). "Effect of Oculus Guided Physical Therapy in Adjunct to Conventional Therapy in Lateral Epicondylitis Patients." JOURNAL OF PHARMACEUTICAL RESEARCH INTERNATIONAL 33(43B): 191-196. | wrong intervention | |
| 192 | Badke, C. M., et al. (2022). "Virtual Reality in the Pediatric Intensive Care Unit: Patient Emotional and Physiologic Responses." FRONTIERS IN DIGITAL HEALTH 4. | wrong population | |
| 193 | Baetzner, A. S., et al. (2022). "Preparing medical first responders for crises: a systematic literature review of disaster training programs and their effectiveness." Scand J Trauma Resusc Emerg Med 30(1): 76. | wrong design | |
| 194 | Bagayogo, I. P., et al. (2018). "Providing Mental Health Services in the Primary Care Setting: the Experiences and Perceptions of General Practitioners at a New York City Clinic." PSYCHIATRIC QUARTERLY 89(4): 897-908. | wrong design | |
| 195 | Bagde, M. N. (2016). "Characterization of failure modes and planned stabilization measures for the Ajanta caves in India." INTERNATIONAL JOURNAL OF ROCK MECHANICS AND MINING SCIENCES 81: 12-18. | wrong design | |
| 196 | Bagger, B., et al. (2024). "Virtual Reality Technology and Digitalized Forest Bathing in Nursing Care - The Experience of Well-being and Quality of Life." NORDISK SYGEPLEJEFORSKNING-NORDIC NURSING RESEARCH 14(1). | wrong design | |
| 197 | Bagger, B., et al. (2024). "Virtual Reality Technology and Digitalized Forest Bathing in Nursing Care - The Experience of Well-being and Quality of Life." Nordic Nursing Research / Nordisk Sygeplejeforskning 14(1): 1-12. | wrong design | |
| 198 | Bagher, S. M., et al. (2023). "The effect of virtual reality distraction on anxiety level during dental treatment among anxious pediatric patients: a randomized clinical trial." JOURNAL OF CLINICAL PEDIATRIC DENTISTRY 47(4): 63-71. | wrong population | |
| 199 | Bagheri, R., et al. (2010). "The effect of aging on the fracture toughness of esthetic restorative materials." Am J Dent 23(3): 142-146. | wrong intervention | |
| 200 | Bahcivan, O., et al. (2022). "A single-session Mindfulness-Based Swinging Technique vs. cognitive disputation intervention among women with breast cancer: A pilot randomised controlled study examining the efficacy at 8-week follow-up." FRONTIERS IN PSYCHOLOGY 13. | wrong population | |
| 201 | Bahr, L. M., et al. (2021). "Dissociation of endocrine responses to the Trier Social Stress Test in Virtual Reality (VR-TSST) by the benzodiazepine alprazolam and the translocator protein 18 kDa (TSPO) ligand etifoxine." PSYCHONEUROENDOCRINOLOGY 124. | wrong population | |
| 202 | Bahrololoomi, Z., et al. (2024). "Efficacy of Virtual Reality Distraction in Reduction of Pain and Anxiety of Pediatric Dental Patients in an Iranian Population: A Split-Mouth Randomized Crossover Clinical Trial." INTERNATIONAL JOURNAL OF DENTISTRY 2024. | wrong population | |
| 203 | Baiocco, R., et al. (2011). "Daily patterns of communication and contact between Italian early adolescents and their friends." Cyberpsychology, Behavior, and Social Networking 14(7-8): 467-471. | wrong population | |
| 204 | Bajunaid, K., et al. (2017). "Impact of acute stress on psychomotor bimanual performance during a simulated tumor resection task." JOURNAL OF NEUROSURGERY 126(1): 71-80. | wrong intervention | |
| 205 | Bakare, A. A., et al. (2022). "Clinical Outcomes After 4-and 5-Level Anterior Cervical Discectomy and Fusion for Treatment of Symptomatic Multilevel Cervical Spondylosis." WORLD NEUROSURGERY 163: E363-E376. | wrong intervention | |
| 206 | Bakbak, E., et al. (2024). "Icosapent ethyl modulates circulating vascular regenerative cell content: The IPE-PREVENTION CardioLink-14 trial." Med 5(7): 718-734.e714. | wrong intervention | |
| 207 | Baker, B. I., et al. (2020). "Defining characteristics of immersion carbon dioxide gas for successful euthanasia of neonatal and young broilers." POULTRY SCIENCE 99(9): 4408-4416. | wrong population | |
| 208 | Bakhaider, A. A., et al. (2024). "Effect of Virtual Reality on Dental Pain in Children during Local Anesthesia Administration." JOURNAL OF THE INTERNATIONAL CLINICAL DENTAL RESEARCH ORGANIZATION 16(1): 66-69. | wrong population | |
| 209 | Bakhsh, A., et al. (2019). "An Evaluation of the Impact of High-Fidelity Endovascular Simulation on Surgeon Stress and Technical Performance." JOURNAL OF SURGICAL EDUCATION 76(3): 864-871. | wrong population | |
| 210 | Baldimtsi, E., et al. (2023). "Effects of Virtual Reality Physical and Cognitive Training Intervention On Cognitive Abilities of Elders with Mild Cognitive Impairment." Journal of Alzheimer's Disease Reports 7(1): 1475-1490. | wrong intervention | |
| 211 | Balki, E., et al. (2022). "Effectiveness of Technology Interventions in Addressing Social Isolation, Connectedness, and Loneliness in Older Adults: Systematic Umbrella Review." JMIR Aging 5(4): e40125. | wrong design | |
| 212 | Ball, S., et al. (2015). myShoes: An Immersive Simulation of Dementia. PROCEEDINGS OF THE 10TH INTERNATIONAL CONFERENCE ON E-LEARNING (ICEL 2015): 16-23. | wrong outcomes | |
| 213 | Ballantyne, R. and P. M. Rea (2019). A Game Changer: 'The Use of Digital Technologies in the Management of Upper Limb Rehabilitation'. BIOMEDICAL VISUALISATION, VOL 5. P. M. Rea. 1205: 117-147. | wrong design | |
| 214 | Baloch, G. M., et al. (2021). "Coping with COVID-19: The Strategies Adapted by Pakistani Students to Overcome Implications." INTERNATIONAL JOURNAL OF ENVIRONMENTAL RESEARCH AND PUBLIC HEALTH 18(4). | wrong population | |
| 215 | Baltacioglu, I. H., et al. (2024). "Marginal adaptation of bulk-fill resin composites with different viscosities in class II restorations: a micro-CT evaluation." BMC ORAL HEALTH 24(1). | wrong population | |
| 216 | Banducci, S. E., et al. (2017). "Active experiencing training improves episodic memory recall in older adults." Frontiers in Aging Neuroscience 9: 11. | wrong intervention | |
| 217 | Bandukda, M., et al. (2021). Rethinking the Senses: A Workshop on Multisensory Embodied Experiences and Disability Interactions. EXTENDED ABSTRACTS OF THE 2021 CHI CONFERENCE ON HUMAN FACTORS IN COMPUTING SYSTEMS (CHI'21). | wrong design | |
| 218 | Bandurska, H. and M. Cieslak (2013). "The interactive effect of water deficit and UV-B radiation on salicylic acid accumulation in barley roots and leaves." ENVIRONMENTAL AND EXPERIMENTAL BOTANY 94: 9-18. | wrong population | |
| 219 | Bang, P. (2000). Pediatric Implications of Normal Insulin-GH-IGF Axis Physiology. Endotext. K. R. Feingold, B. Anawalt, M. R. Blackman et al. South Dartmouth (MA), MDText.com, Inc. | wrong outcomes | |
| 220 | Baniebrahimi, G., et al. (2022). "Effects of Virtual Reality Versus Game Applications on Children?s Dental Fear: A Randomized Clinical Trial." INTERNATIONAL JOURNAL OF PEDIATRICS-MASHHAD 10(12): 17068-17076. | wrong population | |
| 221 | Baños, R. M., et al. (2012). "Positive mood induction procedures for virtual environments designed for elderly people." Interacting with Computers 24(3): 131-138. | wrong outcomes | |
| 222 | Banville, F., et al. (2017). Using Virtual Reality to Assess the Elderly: The Impact of Human-Computer Interfaces on Cognition. HUMAN INTERFACE AND THE MANAGEMENT OF INFORMATION: SUPPORTING LEARNING, DECISION-MAKING AND COLLABORATION, HCI INTERNATIONAL 2017, PT II. 10274: 113-123. | wrong outcomes | |
| 223 | Bao, M. H., et al. (2018). "Resident Participation is Not Associated With Worse Outcomes After TKA." CLINICAL ORTHOPAEDICS AND RELATED RESEARCH 476(7): 1375-1390. | wrong population | |
| 224 | Bao, X., et al. (2021). "Preparation of basalt fibers grafted with amine terminated urea-based oligomer and its application in reinforcing conventional glass ionomer cement." J Mech Behav Biomed Mater 123: 104785. | wrong population | |
| 225 | Bapka, V., et al. (2018). Brain Plasticity in Older Adults: Could It Be Better Enhanced by Cognitive Training via an Adaptation of the Virtual Reality Platform FitForAll or via a Commercial Video Game? INTERACTIVE MOBILE COMMUNICATION TECHNOLOGIES AND LEARNING. 725: 728-742. | wrong outcomes | |
| 226 | Barabanschikov, V. A. and V. V. Selivanov (2023). "REDUCING ANXIETY AND DEPRESSION THROUGH PROGRAMS ON A HIGH IMMERSIVE VIRTUAL REALITY HEADSET." EKSPERIMENTALNAYA PSIKHOLOGIYA 16(2): 36-48. | wrong design | |
| 227 | Baranowski, T. and E. J. Lyons (2020). "Scoping Review of Pokémon Go: Comprehensive Assessment of Augmented Reality for Physical Activity Change." Games Health J 9(2): 71-84. | wrong design | |
| 228 | Barberia, I., et al. (2018). "Virtual mortality and near-death experience after a prolonged exposure in a shared virtual reality may lead to positive life-attitude changes." PLoS ONE 13(11): 31. | wrong population | |
| 229 | Barcatta, K., et al. (2022). "When Less Is More: Investigating Factors Influencing the Distraction Effect of Virtual Reality From Pain." FRONTIERS IN PAIN RESEARCH 2. | wrong population | |
| 230 | Barclay, R. E., et al. (2015). "Interventions for improving community ambulation in individuals with stroke." COCHRANE DATABASE OF SYSTEMATIC REVIEWS(3). | wrong design | |
| 231 | Barhorst-Cates, E. M., et al. (2022). "Does spatial perspective in virtual reality affect imitation accuracy in stroke patients?" FRONTIERS IN VIRTUAL REALITY 3. | wrong outcomes | |
| 232 | Barhoun, P., et al. (2021). "Mental rotation performance in young adults with and without developmental coordination disorder." HUMAN MOVEMENT SCIENCE 77. | wrong population | |
| 233 | Barnett, M. D. and C. J. Chek (2023). "Inhibition mediates the relationship between age cohort and virtual reality-based prospective memory." J Clin Exp Neuropsychol 45(4): 411-422. | wrong outcomes | |
| 234 | Barnett, M. D. and C. J.-W. Chek (2023). "Inhibition mediates the relationship between age cohort and virtual reality-based prospective memory." Journal of Clinical and Experimental Neuropsychology 45(4): 411-422. | wrong outcomes | |
| 235 | Barnett, M. D. and C. J. W. Chek (2023). "Inhibition mediates the relationship between age cohort and virtual reality-based prospective memory." JOURNAL OF CLINICAL AND EXPERIMENTAL NEUROPSYCHOLOGY 45(4): 411-422. | wrong outcomes | |
| 236 | Barnett, M. D. and A. M. Coldiron (2023). "Development of the Virtual Kitchen Protocol for Prospective Memory: a virtual reality-based measure of everyday prospective memory abilities." J Clin Exp Neuropsychol 45(6): 618-635. | wrong population | |
| 237 | Baron, I., et al. (2013). "Paleostress analysis of a gigantic gravitational mass movement in active tectonic setting: The Qoshadagh slope failure, Ahar, NW Iran." TECTONOPHYSICS 605: 70-87. | wrong population | |
| 238 | Barrett, K. T., et al. (2021). "Impaired cardiorespiratory responses to hypercapnia in neonatal mice lacking PAC1 but not VPAC2 receptors." AMERICAN JOURNAL OF PHYSIOLOGY-REGULATORY INTEGRATIVE AND COMPARATIVE PHYSIOLOGY 320(2): R116-R128. | wrong population | |
| 239 | Barsasella, D., et al. (2021). "Effects of Virtual Reality Sessions on the Quality of Life, Happiness, and Functional Fitness among the Older People: A Randomized Controlled Trial from Taiwan." COMPUTER METHODS AND PROGRAMS IN BIOMEDICINE 200. | wrong intervention | |
| 240 | Barsasella, D., et al. (2021). "Effects of Virtual Reality Sessions on the Quality of Life, Happiness, and Functional Fitness among the Older People: A Randomized Controlled Trial from Taiwan." Comput Methods Programs Biomed 200: 105892. | duplicate |  |
| 241 | Barsasella, D., et al. (2020). Opinions regarding Virtual Reality among Older People in Taiwan. PROCEEDINGS OF THE 6TH INTERNATIONAL CONFERENCE ON INFORMATION AND COMMUNICATION TECHNOLOGIES FOR AGEING WELL AND E-HEALTH (ICT4AWE): 165-171. | duplicate |  |
| 242 | Bartalena, L. (2000). Graves’ Disease: Complications. Endotext. K. R. Feingold, B. Anawalt, M. R. Blackman et al. South Dartmouth (MA), MDText.com, Inc. | duplicate |  |
| 243 | Bartawi, E. H., et al. (2022). "Electron microscopy analysis of grain boundaries and intergranular corrosion in aged Al-Mg-Si alloy doped with 0.05 wt% Cu." CORROSION SCIENCE 209. | duplicate |  |
| 244 | Barton, H., et al. (2018). "Use of grass seed resources c.31 ka by modern humans at the Haua Fteah cave, northeast Libya." JOURNAL OF ARCHAEOLOGICAL SCIENCE 99: 99-111. | duplicate |  |
| 245 | Baschong, A., et al. (2021). "Itch reduction using immersive virtual reality-An experimental pilot study." DERMATOLOGIC THERAPY 34(4). | duplicate |  |
| 246 | Baschong, A., et al. (2021). "Itch reduction using immersive virtual reality—An experimental pilot study." Dermatologic Therapy 34(4). | duplicate |  |
| 247 | Basdekidou, C., et al. (2023). "A Low-cost Feasibility Training Study for DCD Children's Perceptual-motor Therapy." BALTIC JOURNAL OF MODERN COMPUTING 11(4): 726-754. | duplicate |  |
| 248 | Basharat, A., et al. (2023). "Virtual reality as a tool to explore multisensory processing before and after engagement in physical activity." Frontiers in Aging Neuroscience: 1-22. | duplicate |  |
| 249 | Basit, M. M., et al. (2014). The Effects of Aging on the Anand Viscoplastic Constitutive Model for SAC305 Solder. 2014 IEEE INTERSOCIETY CONFERENCE ON THERMAL AND THERMOMECHANICAL PHENOMENA IN ELECTRONIC SYSTEMS (ITHERM): 112-126. | duplicate |  |
| 250 | Basson, I., et al. (2017). "Structural analysis and 3D modelling of major mineralizing structures at the Phalaborwa copper deposit." ORE GEOLOGY REVIEWS 83: 30-42. | duplicate |  |
| 251 | Bateman, R. M., et al. (2016). "36th International Symposium on Intensive Care and Emergency Medicine : Brussels, Belgium. 15-18 March 2016." Crit Care 20(Suppl 2): 94. | duplicate |  |
| 252 | Baumgartner, E., et al. (2022). "Exploring the Impact of Extended Reality (XR) on Spatial Reasoning of Elementary Students." TECHTRENDS 66(5): 825-836. | duplicate |  |
| 253 | Bautista, J. C. C. and A. Sánchez-Suricalday (2023). "Effective interventions for improving social skills in people with high-functioning autism spectrum disorder: a systematic review." BORDON-REVISTA DE PEDAGOGIA 75(3). | duplicate |  |
| 254 | Bautista-Ortega, J., et al. (2013). "Effects of arginine and antioxidant vitamins on pulmonary artery reactivity to phenylephrine in the broiler chicken." POULTRY SCIENCE 92(4): 1062-1072. | duplicate |  |
| 255 | Baytar, C. and K. Bollucuoglu (2023). "Effect of virtual reality on preoperative anxiety in patients undergoing septorhinoplasty." BRAZILIAN JOURNAL OF ANESTHESIOLOGY 73(2): 159-164. | duplicate |  |
| 256 | Bazargani, J. S., et al. (2024). "Alzheimer's disease diagnosis in the metaverse." Comput Methods Programs Biomed 255: 108348. | duplicate |  |
| 257 | Bazargani, J. S., et al. (2024). "Alzheimer's disease diagnosis in the metaverse." COMPUTER METHODS AND PROGRAMS IN BIOMEDICINE 255. | duplicate |  |
| 258 | Baziliansky, S., et al. (2023). "Longitudinal trajectories of depression and quality of life in a cohort of cancer survivors and individuals without cancer in Europe." J Cancer Surviv. | duplicate |  |
| 259 | Beasley-Hall, P. G., et al. (2018). "A revised phylogeny of macropathine cave crickets (Orthoptera: Rhaphidophoridae) uncovers a paraphyletic Australian fauna." MOLECULAR PHYLOGENETICS AND EVOLUTION 126: 153-161. | duplicate |  |
| 260 | Beaudoin, M., et al. (2020). "The impact of embodying an "elderly" body avatar on motor imagery." Exp Brain Res 238(6): 1467-1478. | duplicate |  |
| 261 | Becam, G. and T. Chevalier (2019). "Neandertal features of the deciduous and permanent teeth from Portel-Ouest Cave (Ariege, France)." AMERICAN JOURNAL OF PHYSICAL ANTHROPOLOGY 168(1): 45-69. | duplicate |  |
| 262 | Beck, H. B., et al. (2021). "Longitudinal associations between internalizing symptoms and driving avoidance in newly licensed adolescents." JOURNAL OF CLINICAL PSYCHOLOGY 77(4): 1131-1148. | duplicate |  |
| 263 | Becker, L., et al. (2023). "Differences in stress system (re-)activity between single and dual- or multitasking in healthy adults: a systematic review and meta-analysis." HEALTH PSYCHOLOGY REVIEW 17(1): 78-103. | duplicate |  |
| 264 | Behera, S. K., et al. (2024). "A Prospective Randomized Controlled Trial Using Virtual Reality in Pediatric Pre-intervention Echocardiograms to Decrease Child Anxiety and Fear." PEDIATRIC CARDIOLOGY. | duplicate |  |
| 265 | Bekelis, K., et al. (2017). "Effect of an Immersive Preoperative Virtual Reality Experience on Patient Reported Outcomes <i>A Randomized Controlled Trial</i>." ANNALS OF SURGERY 265(6): 1068-1073. | duplicate |  |
| 266 | Bele, A., et al. (2016). "Aging behavior of the silicone dielectric elastomers in a simulated marine environment." RSC ADVANCES 6(11): 8941-8955. | duplicate |  |
| 267 | Bell, I. H., et al. (2022). "Ownership, Use of, and Interest in Digital Mental Health Technologies Among Clinicians and Young People Across a Spectrum of Clinical Care Needs: Cross-sectional Survey." JMIR MENTAL HEALTH 9(5). | duplicate |  |
| 268 | Bell, J. A., et al. (2023). "Should an Age Cutoff Be Considered for Elective Total Knee Arthroplasty Patients? An Analysis of Operative Success Based on Patient-Reported Outcomes." JOURNAL OF KNEE SURGERY 36(09): 1001-1011. | duplicate |  |
| 269 | Bell, V., et al. (2011). "Correlates of perceptual distortions in clinical and non-clinical populations using the Cardiff Anomalous Perceptions Scale (CAPS): Associations with anxiety and depression and a re-validation using a representative population sample." PSYCHIATRY RESEARCH 189(3): 451-457. | duplicate |  |
| 270 | Bella, P., et al. (2022). "Speleogenesis in a lens of metamorphosed limestone and ankerite: Ochtina Aragonite Cave, Slovakia." INTERNATIONAL JOURNAL OF SPELEOLOGY 51(1): 13-28. | duplicate |  |
| 271 | Bella, P., et al. (2022). "Sulfuric acid speleogenesis and surface landform evolution along the Vienna Basin Transfer Fault: Plavecky Karst, Slovakia." INTERNATIONAL JOURNAL OF SPELEOLOGY 51(2): 105-122. | duplicate |  |
| 272 | Bella, P., et al. (2016). "Josvafo paleo-polje: morphology and relation to the landform evolution of Aggtelek Karst and Josva River valley, Hungary." ZEITSCHRIFT FUR GEOMORPHOLOGIE 60(3): 219-235. | duplicate |  |
| 273 | Bellenguez, C., et al. (2022). "New insights into the genetic etiology of Alzheimer's disease and related dementias." Nat Genet 54(4): 412-436. | duplicate |  |
| 274 | Beloncle, F., et al. (2021). "Longitudinal changes in compliance, oxygenation and ventilatory ratio in COVID-19 versus non-COVID-19 pulmonary acute respiratory distress syndrome." CRITICAL CARE 25(1). | duplicate |  |
| 275 | Beltrán, J. E. P., et al. (2017). Video games as an influence in meaningful learning. 2017 EUROPEAN CONFERENCE ON ELECTRICAL ENGINEERING AND COMPUTER SCIENCE (EECS): 347-350. | duplicate |  |
| 276 | Beltran-Alacreu, H., et al. (2022). "A serious game for performing task-oriented cervical exercises among older adult patients with chronic neck pain: Development, suitability, and crossover pilot study." JMIR Serious Games 10(1): 1-14. | duplicate |  |
| 277 | Ben Abdessalem, H., et al. (2021). "Virtual Reality Zoo Therapy for Alzheimer's Disease Using Real-Time Gesture Recognition." Adv Exp Med Biol 1338: 97-105. | duplicate |  |
| 278 | Ben Abdessalem, H., et al. (2021). Virtual Reality Zoo Therapy for Alzheimer's Disease Using Real-Time Gesture Recognition. GENEDIS 2020: COMPUTATIONAL BIOLOGY AND BIOINFORMATICS. P. Vlamos. 1338: 97-105. | duplicate |  |
| 279 | Ben Khelifa, M. M., et al. (2021). "A Muscular and Cerebral Physiological Indices Assessment for Stress Measuring during Virtual Wheelchair Guidance." BRAIN SCIENCES 11(2). | duplicate |  |
| 280 | Benchimol-Elkaim, B., et al. (2024). "Nature-based mindfulness programs using virtual reality to reduce pediatric perioperative anxiety: a narrative review." FRONTIERS IN PEDIATRICS 12. | duplicate |  |
| 281 | Benfield, R., et al. (2018). "Culture, bathing and hydrotherapy in labor: An exploratory descriptive pilot study." Midwifery 64: 110-114. | duplicate |  |
| 282 | Benfield, R. D., et al. (2010). "The Effects of Hydrotherapy on Anxiety, Pain, Neuroendocrine Responses, and Contraction Dynamics During Labor." BIOLOGICAL RESEARCH FOR NURSING 12(1): 28-36. | duplicate |  |
| 283 | Benham, S., et al. (2019). "Immersive virtual reality for the management of pain in community-dwelling older adults." OTJR: Occupation, Participation and Health 39(2): 90-96. | duplicate |  |
| 284 | Benham, S., et al. (2022). "Effects of community-based virtual reality on daily activities and quality of life." Physical & Occupational Therapy in Geriatrics. | duplicate |  |
| 285 | Benitez-Lugo, M. L., et al. (2022). "Effectiveness of feedback-based technology on physical and cognitive abilities in the elderly." FRONTIERS IN AGING NEUROSCIENCE 14. | duplicate |  |
| 286 | Bennewith, C., et al. (2024). "Sublime and extended reality experiences to enhance emotional wellbeing for autistic people: A state of the art review and narrative synthesis." INTERNATIONAL JOURNAL OF SOCIAL PSYCHIATRY. | duplicate |  |
| 287 | Benoit, J., et al. (2023). "Early-Stage Contactin-Associated Protein-like 2 Limbic Encephalitis: Clues for Diagnosis." Neurol Neuroimmunol Neuroinflamm 10(1). | duplicate |  |
| 288 | Benoit, M., et al. (2015). "Is it possible to use highly realistic virtual reality in the elderly? A feasibility study with image-based rendering." NEUROPSYCHIATRIC DISEASE AND TREATMENT 11: 557-563. | duplicate |  |
| 289 | Benoit, M., et al. (2015). "Is it possible to use highly realistic virtual reality in the elderly? A feasibility study with image-based rendering." Neuropsychiatric Disease and Treatment 11: 7. | duplicate |  |
| 290 | Berard, J., et al. (2012). "Impact of aging on visual reweighting during locomotion." Clinical Neurophysiology 123(7): 1422-1428. | duplicate |  |
| 291 | Berard, J. R., et al. (2011). "Evidence for the use of rotational optic flow cues for locomotor steering in healthy older adults." Journal of Neurophysiology 106(3): 1089-1096. | duplicate |  |
| 292 | Berberyan, A. S., et al. (2023). "Virtual Reality as Anxiety Management Tool." INTERNATIONAL JOURNAL OF COGNITIVE RESEARCH IN SCIENCE ENGINEERING AND EDUCATION-IJCRSEE 11(3): 449-459. | duplicate |  |
| 293 | Berendonk, C., et al. (2020). "A Narrative Care approach for persons living with dementia in institutional care settings." International journal of older people nursing 15(1): e12278. | duplicate |  |
| 294 | Berezina, T., et al. (2022). "Using Virtual Reality Techniques to Alleviate Cognitive Fatigue in Graduate Students Working while in College." EUROPEAN JOURNAL OF CONTEMPORARY EDUCATION 11(1): 36-46. | duplicate |  |
| 295 | Berger, L. R., et al. (2017). "<i>Homo naledi</i> and Pleistocene hominin evolution in subequatorial Africa." ELIFE 6. | duplicate |  |
| 296 | Bergoli, C. D., et al. (2016). "Ceramic Repair Without Hydrofluoric Acid." J Adhes Dent 18(4): 283-287. | duplicate |  |
| 297 | Berketis, K. and D. Tzetzis (2010). "The compression-after-impact strength of woven and non-crimp fabric reinforced composites subjected to long-term water immersion ageing." JOURNAL OF MATERIALS SCIENCE 45(20): 5611-5623. | duplicate |  |
| 298 | Berkhof, M., et al. (2024). "Exploring the role of clinical and demographic characteristics on the effects of virtual reality cognitive behavioral therapy for psychosis: A moderator analysis." ACTA PSYCHIATRICA SCANDINAVICA. | duplicate |  |
| 299 | Berkowitz, S. A., et al. (2022). "Changes in Food Insecurity and Changes in Patient-Reported Outcomes: a Nationally Representative Cohort Study." JOURNAL OF GENERAL INTERNAL MEDICINE 37(14): 3638-3644. | duplicate |  |
| 300 | Berman, J., et al. (2020). "The impact of Age-Tastic! On health literacy about depression among older adults: A pilot study." Educational Gerontology 46(3): 117-128. | duplicate |  |
| 301 | Bernaerts, S., et al. (2022). "Virtual Reality for Distraction and Relaxation in a Pediatric Hospital Setting: An Interventional Study With a Mixed-Methods Design." FRONTIERS IN DIGITAL HEALTH 4. | duplicate |  |
| 302 | Bernardes, M., et al. (2018). "A Case of Cephalic Tetanus in an Elderly Patient with Trismus." Case Rep Infect Dis 2018: 1247256. | duplicate |  |
| 303 | Berruti, G. L. F., et al. (2020). "The use of blades and pointed tools during middle palaeolithic, the example of Riparo Tagliente (VR)." QUATERNARY INTERNATIONAL 554: 45-59. | duplicate |  |
| 304 | Berry, H. G. and L. J. Caplan (2010). "Employment and Earnings Growth Among Transition-Age Supplemental Security Income Program Participants." JOURNAL OF DISABILITY POLICY STUDIES 21(3): 152-159. | duplicate |  |
| 305 | Bertolo, M. and I. Mariani (2013). GAME AND PLAY AS MEANS FOR LEARNING EXPERIENCES. 7TH INTERNATIONAL TECHNOLOGY, EDUCATION AND DEVELOPMENT CONFERENCE (INTED2013): 698-707. | duplicate |  |
| 306 | Bertrand, P. J., et al. (2021). "Quality of life in patients with uveitis: data from the ULISSE study (Uveitis: cLInical and medico-economic evaluation of a Standardised Strategy for the Etiological diagnosis)." BRITISH JOURNAL OF OPHTHALMOLOGY 105(7): 935-940. | duplicate |  |
| 307 | Besirik, S. A. and N. C. Sahiner (2024). "Comparison of the effectiveness of three different distraction methods in reducing pain and anxiety during blood drawing in children: A randomized controlled study." JOURNAL OF PEDIATRIC NURSING-NURSING CARE OF CHILDREN & FAMILIES 79: 225-233. | duplicate |  |
| 308 | Bessette, M. C., et al. (2019). "Predictors of Pain and Function Before Knee Arthroscopy." Orthop J Sports Med 7(5): 2325967119844265. | duplicate |  |
| 309 | Bessette, M. C., et al. (2019). "Predictors of Pain and Function Before Knee Arthroscopy." ORTHOPAEDIC JOURNAL OF SPORTS MEDICINE 7(5). | duplicate |  |
| 310 | Beverly, E., et al. (2022). "A tranquil virtual reality experience to reduce subjective stress among COVID-19 frontline healthcare workers." PLoS ONE 17(2 February). | duplicate |  |
| 311 | Bexson, C., et al. (2024). "Safety of virtual reality use in children: a systematic review." EUROPEAN JOURNAL OF PEDIATRICS 183(5): 2071-2090. | duplicate |  |
| 312 | Bhalla, A. K., et al. (2021). "The Association Between Ventilatory Ratio and Mortality in Children and Young Adults." RESPIRATORY CARE 66(2): 205-212. | duplicate |  |
| 313 | Bhalla, A. K., et al. (2024). "Noninvasive Surrogate for Physiologic Dead Space Using the Carbon Dioxide Ventilatory Equivalent: Testing in a Single-Center Cohort, 2017-2023." PEDIATRIC CRITICAL CARE MEDICINE 25(9): 784-794. | duplicate |  |
| 314 | Bhasin, S., et al. (2018). "Strategies to Reduce Injuries and Develop Confidence in Elders (STRIDE): A Cluster-Randomized Pragmatic Trial of a Multifactorial Fall Injury Prevention Strategy: Design and Methods." J Gerontol A Biol Sci Med Sci 73(8): 1053-1061. | duplicate |  |
| 315 | Bhasin, S., et al. (2000). Age-Related Changes in the Male Reproductive System. Endotext. K. R. Feingold, B. Anawalt, M. R. Blackman et al. South Dartmouth (MA), MDText.com, Inc. | duplicate |  |
| 316 | Bidgoli, Z. A., et al. (2023). "Does a 30-minute introductory visit to the operating room reduce patients' anxiety before elective surgery? a prospective controlled observational study." PATIENT SAFETY IN SURGERY 17(1). | duplicate |  |
| 317 | Bigelsen, J. and C. Schupak (2011). "Compulsive fantasy: Proposed evidence of an under-reported syndrome through a systematic study of 90 self-identified non-normative fantasizers." CONSCIOUSNESS AND COGNITION 20(4): 1634-1648. | duplicate |  |
| 318 | Bigga, G., et al. (2015). "Paleoenvironment and possibilities of plant exploitation in the Middle Pleistocene of Schoningen (Germany). Insights from botanical macro-remains and pollen." JOURNAL OF HUMAN EVOLUTION 89: 92-104. | duplicate |  |
| 319 | Bilello, D., et al. (2023). "Adolescent risk-taking and decision making: a qualitative investigation of a virtual reality experience of gangs and violence." FRONTIERS IN VIRTUAL REALITY 4. | duplicate |  |
| 320 | Billet, M., et al. (2024). "The effects of age on objective and subjective recollection after visiting a virtual apartment." Neuropsychol Dev Cogn B Aging Neuropsychol Cogn 31(2): 340-361. | duplicate |  |
| 321 | Biradar, S. P., et al. (2022). "Polyherbal decoction modulates redox homeostasis during Malachite green induced metabolic stress in <i>Saccharomyces cerevisiae</i>." INDIAN JOURNAL OF EXPERIMENTAL BIOLOGY 60(1): 17-26. | duplicate |  |
| 322 | Birnie, K. A., et al. (2018). "Usability Testing of an Interactive Virtual Reality Distraction Intervention to Reduce Procedural Pain in Children and Adolescents With Cancer." JOURNAL OF PEDIATRIC ONCOLOGY NURSING 35(6): 406-416. | duplicate |  |
| 323 | Bishawi, M., et al. (2022). "Health-related quality of life impacts upon 5-year survival after coronary artery bypass surgery." JOURNAL OF CARDIAC SURGERY 37(12): 4899-4905. | duplicate |  |
| 324 | Björling, E., et al. (2022). "Exploring the Effect of a Nature-based Virtual Reality Environment on Stress in Adolescents." FRONTIERS IN VIRTUAL REALITY 3. | duplicate |  |
| 325 | Björling, E. A., et al. (2020). The Experience and Effect of Adolescent to Robot Stress Disclosure: A Mixed-Methods Exploration. SOCIAL ROBOTICS, ICSR 2020. 12483: 604-615. | duplicate |  |
| 326 | Blackburn, C., et al. (2018). "Mechanical properties of resin-ceramic CAD-CAM materials after accelerated aging." J Prosthet Dent 119(6): 954-958. | duplicate |  |
| 327 | Blasco-Peris, C., et al. (2022). "Effects of Exergaming in Patients with Cardiovascular Disease Compared to Conventional Cardiac Rehabilitation: A Systematic Review and Meta-Analysis." INTERNATIONAL JOURNAL OF ENVIRONMENTAL RESEARCH AND PUBLIC HEALTH 19(6). | duplicate |  |
| 328 | Blazquez-González, P., et al. (2024). "Impact of virtual reality-based therapy on post-stroke depression: A systematic review and meta-analysis of randomized controlled trials." WORLDVIEWS ON EVIDENCE-BASED NURSING 21(2): 194-201. | duplicate |  |
| 329 | Bloch, F. (2017). "Literature review and meta-analysis of risk factors for delayed post-traumatic stress disorder in older adults after a fall." INTERNATIONAL JOURNAL OF GERIATRIC PSYCHIATRY 32(2): 136-140. | duplicate |  |
| 330 | Bloch, F., et al. (2013). "Virtual Reality Exposure Therapy in posttraumatic stress disorder: A brief review to open new opportunities for post-fall syndrome in elderly subjects." EUROPEAN GERIATRIC MEDICINE 4(6): 427-430. | duplicate |  |
| 331 | Blokzijl, S. J., et al. (2019). "Willingness to undergo colonoscopy with virtual reality instead of procedural sedation and analgesia." EUROPEAN JOURNAL OF GASTROENTEROLOGY & HEPATOLOGY 31(3): 334-339. | duplicate |  |
| 332 | Blomstrom, M., et al. (2022). "Addressing fear of death and dying: traditional and innovative interventions." MORTALITY 27(1): 18-37. | duplicate |  |
| 333 | Boffi, M., et al. (2022). "Visual post-occupancy evaluation of a restorative garden using virtual reality photography: Restoration, emotions, and behavior in older and younger people." FRONTIERS IN PSYCHOLOGY 13. | duplicate |  |
| 334 | Bogdanowicz, W., et al. (2020). "Pollen assemblage and environmental DNA changes: A 4300-year-old bat guano deposit from Jamaica." QUATERNARY INTERNATIONAL 558: 47-58. | duplicate |  |
| 335 | Bogdanski, E. (2023). "The Effects of Virtual Reality Telemedicine With Pediatric Patients Diagnosed With Posttraumatic Stress Disorder: Exploratory Research Method Case Report." JMIR FORMATIVE RESEARCH 7. | duplicate |  |
| 336 | Bogon, J., et al. (2024). "Age-related changes in time perception: Effects of immersive virtual reality and spatial location of stimuli." ACTA PSYCHOLOGICA 249. | duplicate |  |
| 337 | Boisseau, A. and C. Peyrac (2015). LONG TERM DURABILITY OF COMPOSITES IN MARINE ENVIRONMENT: COMPARATIVE STUDY OF FATIGUE BEHAVIOR. FATIGUE DESIGN 2015, INTERNATIONAL CONFERENCE PROCEEDINGS, 6TH EDITION. 133: 535-544. | duplicate |  |
| 338 | Boitor, A. A., et al. (2023). "The Impact of Simulated Bruxism Forces and Surface Aging Treatments on Two Dental Nano-Biocomposites-A Radiographic and Tomographic Analysis." MEDICINA-LITHUANIA 59(2). | duplicate |  |
| 339 | Bojar, A. V., et al. (2024). "<i>Ursus spelaeus</i> (Rosenmuller, 1794) during the MIS 3: new evidence from the Cioclovina Uscata Cave and radiocarbon age overview for the Carpathians." ISOTOPES IN ENVIRONMENTAL AND HEALTH STUDIES. | duplicate |  |
| 340 | Bökberg, C., et al. (2019). "Evaluation of person-centeredness in nursing homes after a palliative care intervention: pre- and post-test experimental design." BMC Palliat Care 18(1): 44. | duplicate |  |
| 341 | Bolado-Sarabia, J. L., et al. (2018). "Effect of immunocastration on behaviour and blood parameters (cortisol and testosterone) of Holstein bulls." AUSTRAL JOURNAL OF VETERINARY SCIENCES 50(2): 77-81. | duplicate |  |
| 342 | Boldrini, R., et al. (2010). "The Middle Pleistocene fossiliferous sequence of Grotta dei Fiori (Sardinia, Italy): multidisciplinary analysis." BOLLETTINO DELLA SOCIETA PALEONTOLOGICA ITALIANA 49(2): 123-134. | duplicate |  |
| 343 | Boleracki, M., et al. (2015). Developing an Animal Counting Game in Second Life for a Young Adult with Down Syndrome. ASSISTIVE TECHNOLOGY: BUILDING BRIDGES. 217: 71-77. | duplicate |  |
| 344 | Bolgova, O. and V. Mavrych (2024). "Pediatric Solid-State 3D Models of Lumbar Vertebrae and Spine." CUREUS JOURNAL OF MEDICAL SCIENCE 16(4). | duplicate |  |
| 345 | Boller, B., et al. (2021). "Using virtual reality to assess and promote transfer of memory training in older adults with memory complaints: A randomized controlled trial." Frontiers in Psychology 12: 16. | duplicate |  |
| 346 | Bolouki, A., et al. (2024). "Optimizing Virtual Nature for Psychological and Physiological Well-Being: A Systematic Review of the Moderating Effects of Duration, Nature Type, Sample Characteristics, and Immersiveness and Potential Risks of Bias." INTERNATIONAL JOURNAL OF HUMAN-COMPUTER INTERACTION. | duplicate |  |
| 347 | Bonab, H. S., et al. (2024). "The Impact of Virtual Reality Intervention on Emotion Regulation and Executive Functions in Autistic Children." GAMES FOR HEALTH JOURNAL. | duplicate |  |
| 348 | Boo, C., et al. (2022). "Conversation During a Virtual Reality Task Reveals New Structural Language Profiles of Children with ASD, ADHD, and Comorbid Symptoms of Both." JOURNAL OF AUTISM AND DEVELOPMENTAL DISORDERS 52(7): 2970-2983. | duplicate |  |
| 349 | Boot, W. (2020). "International perspectives on technology use: Adoption, proficiency, and relationships with health and well-being...International Society for Gerontechnology's (ISG) 12th World Conference of Gerontechnology, October 6-9, 2020 (Virtual)." Gerontechnology 19: 65-65. | duplicate |  |
| 350 | Borgwardt, A., et al. (2017). "A randomised, controlled clinical study on total hip arthroplasty using 4 different bearings: Results after 10 years." HIP International 27(1): 96-103. | duplicate |  |
| 351 | Bormans, K., et al. (2016). "Virtual memory palaces to improve quality of life in Alzheimer's disease." Annual Review of CyberTherapy and Telemedicine 14: 227-231. | duplicate |  |
| 352 | Bosso, L., et al. (2023). "Analgesic and Anxiolytic Effects of Virtual Reality During Minor Procedures in an Emergency Department: A Randomized Controlled Study." ANNALS OF EMERGENCY MEDICINE 81(1): 84-94. | duplicate |  |
| 353 | Botella, C., et al. (2011). "Treating cockroach phobia using a serious game on a mobile phone and augmented reality exposure: A single case study." COMPUTERS IN HUMAN BEHAVIOR 27(1): 217-227. | duplicate |  |
| 354 | Bottiroli, S., et al. (2021). "The smart aging platform for assessing early phases of cognitive impairment in patients with neurodegenerative diseases." Frontiers in Psychology 12: 13. | duplicate |  |
| 355 | Bottiroli, S., et al. (2017). "Smart aging platform for evaluating cognitive functions in aging: A comparison with the MoCA in a normal population." Frontiers in Aging Neuroscience 9: 14. | duplicate |  |
| 356 | Bottone, F. G., et al. (2013). "The relationship between body mass index and quality of life in community-living older adults living in the United States." JOURNAL OF NUTRITION HEALTH & AGING 17(6): 495-501. | duplicate |  |
| 357 | Boulos, L. J., et al. (2021). "An Iterative and Collaborative End-to-End Methodology Applied to Digital Mental Health." FRONTIERS IN PSYCHIATRY 12. | duplicate |  |
| 358 | Bourassa, K. J., et al. (2020). "The impact of prolonged exposure therapy on social support and PTSD symptoms." J Affect Disord 260: 410-417. | duplicate |  |
| 359 | Bourassa, K. J., et al. (2020). "The Impact of Exposure Therapy on Resting Heart Rate and Heart Rate Reactivity Among Active-Duty Soldiers With Posttraumatic Stress Disorder." PSYCHOSOMATIC MEDICINE 82(1): 108-114. | duplicate |  |
| 360 | Bourrelier, J., et al. (2021). "Enhancement of Anticipatory Postural Adjustments by Virtual Reality in Older Adults with Cognitive and Motor Deficits: A Randomised Trial." GERIATRICS 6(3). | duplicate |  |
| 361 | Bouvet, G., et al. (2016). "In situ monitoring of organic coating swelling by dynamic mechanical analysis and scanning electrochemical microscopy." PROGRESS IN ORGANIC COATINGS 96: 13-18. | duplicate |  |
| 362 | Bouwmeester Stjernetun, B., et al. (2024). ""It´s like walking in a bubble", nursing students´ perspectives on age suit simulation in a home environment - group interviews from reflection seminars." BMC Nurs 23(1): 124. | duplicate |  |
| 363 | Bouwmeester Stjernetun, B., et al. (2024). ""It´s like walking in a bubble", nursing students´ perspectives on age suit simulation in a home environment – group interviews from reflection seminars." BMC Nursing 23(1): 1-13. | duplicate |  |
| 364 | Bower, I. S., et al. (2023). "Functional brain connectivity during exposure to the scale and color of interior built environments." HUMAN BRAIN MAPPING 44(2): 447-457. | wrong population | |
| 365 | Bracq, M. S., et al. (2019). "Learning procedural skills with a virtual reality simulator: An acceptability study." NURSE EDUCATION TODAY 79: 153-160. | wrong population | |
| 366 | Bradfield, J. (2013). "Investigating the potential of micro-focus computed tomography in the study of ancient bone tool function: results from actualistic experiments." JOURNAL OF ARCHAEOLOGICAL SCIENCE 40(6): 2606-2613. | wrong population | |
| 367 | Bradfield, J. (2018). "Identifying animal taxa used to manufacture bone tools during the Middle Stone Age at Sibudu, South Africa: Results of a CT-rendered histological analysis." PLoS ONE 13(11). | wrong population | |
| 368 | Bradfield, J., et al. (2020). "Further evidence for bow hunting and its implications more than 60 000 years ago: Results of a use-trace analysis of the bone point from Klasies River Main site, South Africa." QUATERNARY SCIENCE REVIEWS 236. | wrong intervention | |
| 369 | Bradley, C. F., et al. (2013). "Contributors to Successful VR Outcomes among Non-Latino (Caucasian) and Latino Consumers with Hearing Loss." JOURNAL OF REHABILITATION 79(2): 24-33. | wrong design | |
| 370 | Bragazzi, N. L., et al. (2022). "Planetary sleep medicine: Studying sleep at the individual, population, and planetary level." FRONTIERS IN PUBLIC HEALTH 10. | wrong intervention | |
| 371 | Brazaitis, M., et al. (2017). "Heat transfer and loss by whole-body hyperthermia during severe lower-body heating are impaired in healthy older men." EXPERIMENTAL GERONTOLOGY 96: 12-18. | wrong intervention | |
| 372 | Breslow, A. S., et al. (2024). "Lessons Learned from a Community-led, Pilot Teletherapy Group for Older Women Living with Depression and HIV." AIDS Behav. | wrong intervention | |
| 373 | Breuer, J., et al. (2015). "Sexist games=Sexist gamers? A longitudinal study on the relationship between video game use and sexist attitudes." Cyberpsychology, Behavior, and Social Networking 18(4): 197-202. | wrong population | |
| 374 | Breuss, A., et al. (2024). "Nocturnal vestibular stimulation using a rocking bed improves a severe sleep disorder in a patient with mitochondrial disease." JOURNAL OF SLEEP RESEARCH. | wrong population | |
| 375 | Breysse, J., et al. (2015). "Self-Reported Health Outcomes Associated With Green-Renovated Public Housing Among Primarily Elderly Residents." JOURNAL OF PUBLIC HEALTH MANAGEMENT AND PRACTICE 21(4): 355-367. | wrong intervention | |
| 376 | Brimelow, R. E., et al. (2020). "Preliminary research: Virtual reality in residential aged care to reduce apathy and improve mood." Cyberpsychology, Behavior, and Social Networking 23(3): 165-170. | wrong design | |
| 377 | Brimelow, R. E., et al. (2022). "Feasibility of Group-Based Multiple Virtual Reality Sessions to Reduce Behavioral and Psychological Symptoms in Persons Living in Residential Aged Care." JOURNAL OF THE AMERICAN MEDICAL DIRECTORS ASSOCIATION 23(5): 831-+. | wrong design | |
| 378 | Brito, D. V. C., et al. (2023). "Assessing cognitive decline in the aging brain: lessons from rodent and human studies." NPJ AGING 9(1). | wrong design | |
| 379 | Brito, H., et al. (2021). "Effect of sensorimotor rehabilitation based on an immersive virtual reality model on mental health." Int J Geriatr Psychiatry 37(1). |  |  |
| 380 | Brito, H., et al. (2022). "Effect of sensorimotor rehabilitation based on an immersive virtual reality model on mental health." International Journal of Geriatric Psychiatry 37(1): 1-9. | wrong design | |
| 381 | Brook, G. A., et al. (2011). "Radiocarbon Ages for Coatings on Cupules Ground in Quartzite Bedrock at Rhino Cave in the Kalahari Desert of Botswana, and their Paleoclimatic Significance." GEOARCHAEOLOGY-AN INTERNATIONAL JOURNAL 26(1): 61-82. | wrong population | |
| 382 | Brookman, R., et al. (2024). "Evaluation of an exercise program incorporating an international cycling competition: a multimodal intervention model for physical, psychological, and social wellbeing in residential aged care." BMC GERIATRICS 24(1). | wrong intervention | |
| 383 | Brooks, H., et al. (2021). "Improving mental health literacy among young people aged 11-15 years in Java, Indonesia: the co-development of a culturally-appropriate, user-centred resource (The IMPeTUs Intervention)." CHILD AND ADOLESCENT PSYCHIATRY AND MENTAL HEALTH 15(1). | wrong population | |
| 384 | Brown, N. J., et al. (2015). "Cost-Effectiveness of a Nonpharmacological Intervention in Pediatric Burn Care." VALUE IN HEALTH 18(5): 631-637. | wrong population | |
| 385 | Browning, M., et al. (2023). "Daily exposure to virtual nature reduces symptoms of anxiety in college students." SCIENTIFIC REPORTS 13(1). | wrong population | |
| 386 | Browning, M. H. E. M., et al. (2021). "Psychological impacts from COVID-19 among university students: Risk factors across seven states in the United States." PLoS ONE 16(1): 27. | wrong population | |
| 387 | Bruce, C., et al. (2024). "Revolutionizing Mental Health Nursing Education: Virtual Reality Simulation for Understanding and Intervening in Major Depressive Disorder and Suicidal Thoughts." Nurs Educ Perspect 45(5): 322-324. | wrong population | |
| 388 | Brummelman, E., et al. (2022). "Parental praise and children's exploration: a virtual reality experiment." SCIENTIFIC REPORTS 12(1). | wrong population | |
| 389 | Brungardt, A., et al. (2024). "Patient Outcomes of a Virtual Reality-Based Music Therapy Pilot in Palliative Care." PALLIATIVE MEDICINE REPORTS 5(1): 278-285. | wrong outcomes | |
| 390 | Bruni, F., et al. (2022). "Cognition Meets Gait: Where and How Mind and Body Weave Each Other in a Computational Psychometrics Approach in Aging." FRONTIERS IN AGING NEUROSCIENCE 14. | wrong intervention | |
| 391 | Bruni, F., et al. (2023). "A cross-platform application for the ecological and remote assessment of memory impairment in aging: ECO-MEMORY." VIRTUAL REALITY 27(3): 2757-2767. | wrong intervention | |
| 392 | Bruni, F., et al. (2022). "ObReco-2: Two-step validation of a tool to assess memory deficits using 360° videos." Frontiers in Aging Neuroscience 14: 10. | wrong intervention | |
| 393 | Bruni, F., et al. (2024). "Beyond traditional training: Integrating data from semi-immersive VR dual-task intervention in Parkinsonian Syndromes. A study protocol." PLoS ONE 19(2): 13. | wrong design | |
| 394 | Bruno, R. R., et al. (2020). "Virtual reality-assisted conscious sedation during transcatheter aortic valve implantation: a randomised pilot study." EUROINTERVENTION 16(12): E1014-+. | wrong population | |
| 395 | Buchholz, I., et al. (2021). "Translation and adaptation of the German version of the Veterans Rand-36/12 Item Health Survey." HEALTH AND QUALITY OF LIFE OUTCOMES 19(1). | wrong intervention | |
| 396 | Buele, J., et al. (2024). "Effects of a dual intervention (motor and virtual reality-based cognitive) on cognition in patients with mild cognitive impairment: a single-blind, randomized controlled trial." J Neuroeng Rehabil 21(1): 130. | wrong intervention | |
| 397 | Buele, J., et al. (2024). "Effects of a dual intervention (motor and virtual reality-based cognitive) on cognition in patients with mild cognitive impairment: a single-blind, randomized controlled trial." JOURNAL OF NEUROENGINEERING AND REHABILITATION 21(1). | duplicate |  |
| 398 | Bulas, A. M., et al. (2022). "Preinjury Health Status of Adults With Traumatic Brain Injury: A Preliminary Matched Case-Control Study." JOURNAL OF HEAD TRAUMA REHABILITATION 37(3): E186-E195. | wrong population | |
| 399 | Buldur, B. and M. Candan (2021). "Does Virtual Reality Affect Children's Dental Anxiety, Pain, And Behaviour? A Randomised, Placebo-Controlled, Cross-Over Trial." PESQUISA BRASILEIRA EM ODONTOPEDIATRIA E CLINICA INTEGRADA 21. | wrong population | |
| 400 | Bullock, G. S., et al. (2021). "Health Conditions, Substance Use, Physical Activity, and Quality of Life in Current and Former Baseball Players." ORTHOPAEDIC JOURNAL OF SPORTS MEDICINE 9(11). | wrong population | |
| 401 | Bülthoff, I., et al. (2019). "Face recognition of full-bodied avatars by active observers in a virtual environment." VISION RESEARCH 157: 242-251. | wrong population | |
| 402 | Bunce, D., et al. (2012). "Age and inconsistency in driving performance." Accident Analysis and Prevention 49: 293-299. | wrong intervention | |
| 403 | Buonocore, S., et al. (2022). Virtual Teleoperation Setup for a Bimanual Bartending Robot. EXTENDED REALITY, XR SALENTO 2022, PT II. 13446: 306-325. | wrong intervention | |
| 404 | Burdea, G., et al. (2015). "Feasibility study of theBrightBrainer™ integrative cognitive rehabilitation system for elderly with dementia." Disability and Rehabilitation: Assistive Technology 10(5): 421-432. | wrong design | |
| 405 | Burin, D., et al. (2022). "Neuroendocrine Response and State Anxiety Due to Psychosocial Stress Decrease after a Training with Subject’s Own (but Not Another) Virtual Body: An RCT Study." International Journal of Environmental Research and Public Health 19(10). | wrong population | |
| 406 | Burke, S. L., et al. (2021). "Brief Report: Improving Employment Interview Self-efficacy Among Adults with Autism and Other Developmental Disabilities Using Virtual Interactive Training Agents (ViTA)." JOURNAL OF AUTISM AND DEVELOPMENTAL DISORDERS 51(2): 741-748. | wrong population | |
| 407 | Burkhart, L. and W. Schmidt (2012). "Measuring effectiveness of a spiritual care pedagogy in nursing education." J Prof Nurs 28(5): 315-321. | wrong population | |
| 408 | Burrows, B. T., et al. (2023). "Virtual Reality Mindfulness and Personalized Exercise for Patients on Hemodialysis with Depressive Symptoms: A Feasibility Study." KIDNEY AND DIALYSIS 3(3): 297-310. | wrong population | |
| 409 | Buscariollo, D. L., et al. (2019). "Impact of pre-diagnosis depressive symptoms and health-related quality of life on treatment choice for ductal carcinoma in situ and stage I breast cancer in older women." BREAST CANCER RESEARCH AND TREATMENT 173(3): 709-717. | wrong intervention | |
| 410 | Butt, M., et al. (2022). "Take-Pause: Efficacy of mindfulness-based virtual reality as an intervention in the pediatric emergency department." ACADEMIC EMERGENCY MEDICINE 29(3): 270-277. | wrong population | |
| 411 | Buttazzoni, A., et al. (2021). "Investigating the mental health implications of urban environments with neuroscientific methods and mobile technologies: A systematic literature review." HEALTH & PLACE 70. | wrong design | |
| 412 | Butts, C. L., et al. (2016). "Effects of mild hypohydration on cooling during cold-water immersion following exertional hyperthermia." EUROPEAN JOURNAL OF APPLIED PHYSIOLOGY 116(4): 687-695. | wrong intervention | |
| 413 | Buyuk, E. T., et al. (2021). "The effect of virtual reality on Children's anxiety, fear, and pain levels before circumcision." JOURNAL OF PEDIATRIC UROLOGY 17(1). | wrong population | |
| 414 | Caballero, R., et al. (2024). "Beyond Needles: Pioneering Pediatric Care with Virtual Reality (VR) for TIVAD Access in Oncology." CANCERS 16(12). | wrong population | |
| 415 | Cábelková, I., et al. (2020). "Does Playing Video Games Increase Emotional Creativity?" INTERNATIONAL JOURNAL OF ENVIRONMENTAL RESEARCH AND PUBLIC HEALTH 17(7). | wrong population | |
| 416 | Cabinio, M., et al. (2020). "The Use of a Virtual Reality Platform for the Assessment of the Memory Decline and the Hippocampal Neural Injury in Subjects with Mild Cognitive Impairment: The Validity of Smart Aging Serious Game (SASG)." JOURNAL OF CLINICAL MEDICINE 9(5). | wrong population | |
| 417 | Cacciata, M., et al. (2019). "Effect of exergaming on health-related quality of life in older adults: A systematic review." INTERNATIONAL JOURNAL OF NURSING STUDIES 93: 30-40. | wrong design | |
| 418 | Cacciata, M., et al. (2019). "Effect of exergaming on health-related quality of life in older adults: A systematic review." International Journal of Nursing Studies 93: 30-40. | wrong design | |
| 419 | Caffò, A. O., et al. (2018). "Spatial reorientation decline in aging: The combination of geometry and landmarks." Aging & Mental Health 22(10): 1372-1383. | wrong population | |
| 420 | Cai, X., et al. (2024). "The effects of exergames for cognitive function in older adults with mild cognitive impairment: a systematic review and metaanalysis." Front Neurol 15: 1424390. | wrong design | |
| 421 | Cai, X. W., et al. (2024). "The effects of exergames for cognitive function in older adults with mild cognitive impairment: a systematic review and metaanalysis." FRONTIERS IN NEUROLOGY 15. | wrong design | |
| 422 | Çakir, S. K. and S. Evirgen (2021). "The Effect of Virtual Reality on Pain and Anxiety During Colonoscopy: A Randomized Controlled Trial." TURKISH JOURNAL OF GASTROENTEROLOGY 32(5): 451-457. | wrong population | |
| 423 | Calvo, J. P., et al. (2013). "Pattern of sedimentary infilling of fossil mammal traps formed in pseudokarst at Cerro de los Batallones, Madrid Basin, central Spain." SEDIMENTOLOGY 60(7): 1681-1708. | wrong population | |
| 424 | Camacho-Conde, J. A. (2020). "Cognitive Function Assessment of a Patient With PTSD Before and After EMDR Treatment." JOURNAL OF EMDR PRACTICE AND RESEARCH 14(4): 216-228. | wrong intervention | |
| 425 | Câmara, J., et al. (2023). "Efficacy of adaptive cognitive training through desktop virtual reality and paper-and-pencil in the treatment of mental and behavioral disorders." VIRTUAL REALITY 27(1): 291-306. | wrong population | |
| 426 | Cameirao, M. S., et al. (2016). "The impact of positive, negative and neutral stimuli in a virtual reality cognitive-motor rehabilitation task: a pilot study with stroke patients." JOURNAL OF NEUROENGINEERING AND REHABILITATION 13. | wrong population | |
| 427 | Camporota, L., et al. (2023). "Relationship between D-dimers and dead-space on disease severity and mortality in COVID-19 acute respiratory distress syndrome: A retrospective observational cohort study." JOURNAL OF CRITICAL CARE 77. | wrong intervention | |
| 428 | Campos, M. F., et al. (2018). Comparison between thermal recovery in women with Raynaud's Phenomenon and not diagnosed women using thermography. 2018 40TH ANNUAL INTERNATIONAL CONFERENCE OF THE IEEE ENGINEERING IN MEDICINE AND BIOLOGY SOCIETY (EMBC): 3886-3889. | wrong population | |
| 429 | Campuzano, M. T. G., et al. (2012). The Effect of Aging on Brain Temporal Perception using Virtual Reality Neurocognitive (VRN) Experiments. 2012 ANNUAL INTERNATIONAL CONFERENCE OF THE IEEE ENGINEERING IN MEDICINE AND BIOLOGY SOCIETY (EMBC): 4808-4811. | wrong outcomes | |
| 430 | Can, M. and G. O. Gerçeker (2024). "The effect of the Veinlite PEDI2 and passive virtual reality distraction on peripheral catheter insertion-related emotional behavior, pain, fear, and anxiety of children: A randomized controlled trial." JOURNAL OF PEDIATRIC NURSING-NURSING CARE OF CHILDREN & FAMILIES 78: e227-e235. | wrong population | |
| 431 | Canares, T., et al. (2021). "Pediatric Coping During Venipuncture With Virtual Reality: Pilot Randomized Controlled Trial." JMIR PEDIATRICS AND PARENTING 4(3). | wrong population | |
| 432 | Candela, L., et al. (2023). "The Use of a Virtual Reality Device (HypnoVR) During Extracorporeal Shockwave Lithotripsy for Treatment of Urinary Stones: Initial Results of a Clinical Protocol." UROLOGY 175: 13-17. | wrong design | |
| 433 | Cangas, A. J., et al. (2017). "Stigma-Stop: A Serious Game against the Stigma toward Mental Health in Educational Settings." FRONTIERS IN PSYCHOLOGY 8. | wrong intervention | |
| 434 | Cano, N., et al. (2024). "A multimodal group-based immersive virtual reality intervention for improving cognition and mental health in patients with post-covid-19 condition. A quasi-experimental design study." FRONTIERS IN PSYCHOLOGY 15. | wrong design | |
| 435 | Cantor, J. B., et al. (2011). Short-Term Intensive Cognitive Rehabilitation in OEF/OIF Veterans - Applying the STEP Model. COPING WITH BLAST-RELATED TRAUMATIC BRAIN INJURY IN RETURNING TROOPS: WOUNDS OF WAR III. 86: 55-71. | wrong intervention | |
| 436 | Canty, A. L., et al. (2014). "Evaluation of a virtual reality prospective memory task for use with individuals with severe traumatic brain injury." NEUROPSYCHOLOGICAL REHABILITATION 24(2): 238-265. | wrong population | |
| 437 | Cao, Z. C., et al. (2017). "Real-time Acute Stress Facilitates Allocentric Spatial Processing in a Virtual Fire Disaster." SCIENTIFIC REPORTS 7. | wrong outcomes | |
| 438 | Caputo, A. (2021). "Looking at a Beautiful Moon While Immersed in a Lake of Petroleum: Narratives from Italian Individuals with Hikikomori." PSIHOLOGIJA 54(3): 269-284. | wrong population | |
| 439 | Carannante, F., et al. (2024). "The use of Virtual Reality Hypnosis (HypnoVR) in inguinal hernia surgeries." MINERVA SURGERY. | wrong population | |
| 440 | Carbó, A., et al. (2024). "Usefulness of a virtual reality educational program for reducing preoperative anxiety in children A randomised, single-centre clinical trial." EUROPEAN JOURNAL OF ANAESTHESIOLOGY 41(9): 657-667. | wrong population | |
| 441 | Cárdenas-Egúsquiza, A. L. and D. Berntsen (2023). "Individual differences in autobiographical memory predict the tendency to engage in spontaneous thoughts." MEMORY 31(9): 1134-1146. | wrong population | |
| 442 | Cardi, V., et al. (2012). "The Use of a Nonimmersive Virtual Reality Programme in Anorexia Nervosa: A Single Case-Report." EUROPEAN EATING DISORDERS REVIEW 20(3): 240-245. | wrong design | |
| 443 | Carelli, L., et al. (2011). "The transfer from survey (map-like) to route representations into Virtual Reality Mazes: effect of age and cerebral lesion." JOURNAL OF NEUROENGINEERING AND REHABILITATION 8. | wrong population | |
| 444 | Carpenter, J. H. (2021). "Forty-year natural history study of <i>Bahalana geracei</i> Carpenter, 1981, an anchialine cave-dwelling isopod (Crustacea, Isopoda, Cirolanidae) from San Salvador Island, Bahamas: reproduction, growth, longevity, and population structure." SUBTERRANEAN BIOLOGY 37: 105-156. | wrong population | |
| 445 | Carr, S., et al. (2019). "Multisensory, Multi-Tasking Performance of Older Adults With and Without Subjective Cognitive Decline." MULTISENSORY RESEARCH 32(8): 797-829. | wrong intervention | |
| 446 | Carroll, J., et al. (2021). "A Scoping Review of Augmented/Virtual Reality Health and Wellbeing Interventions for Older Adults: Redefining Immersive Virtual Reality." FRONTIERS IN VIRTUAL REALITY 2. | wrong design | |
| 447 | Carson Iii, C., et al. (2016). "Hypogonadal men with sexual function disorder benefit from LPCN 1021 (oral testosterone)-SOAR (study of androgen replacement) trial." Journal of Urology 195(4): e1010-e1011. | wrong outcomes | |
| 448 | Caruso, T. J., et al. (2020). "Virtual reality during pediatric vascular access: A pragmatic, prospective randomized, controlled trial." PEDIATRIC ANESTHESIA 30(2): 116-123. | wrong population | |
| 449 | Caruso, T. J., et al. (2020). "Retrospective Review of the Safety and Efficacy of Virtual Reality in a Pediatric Hospital." PEDIATRIC QUALITY & SAFETY 5(2). | wrong population | |
| 450 | Cassarino, M., et al. (2019). "Testing Attention Restoration in a Virtual Reality Driving Simulator." FRONTIERS IN PSYCHOLOGY 10. | wrong population | |
| 451 | Cassola, E. G., et al. (2024). "Systematic Review of Music Therapy and Musical Interventions for Patients with Moderate and Severe Mental Disorders." JOURNAL OF INTEGRATIVE AND COMPLEMENTARY MEDICINE 30(9): 819-831. | wrong design | |
| 452 | Cassola, E. G., et al. (2024). "Systematic Review of Music Therapy and Musical Interventions for Patients with Moderate and Severe Mental Disorders." J Integr Complement Med 30(9): 819-831. | wrong design | |
| 453 | Cassola, E. G., et al. (2024). "Systematic Review of Music Therapy and Musical Interventions for Patients with Moderate and Severe Mental Disorders." Journal of Integrative & Complementary Medicine 30(9): 819-831. | wrong design | |
| 454 | Castegnaro, A., et al. (2022). "Assessing mild cognitive impairment using object‐location memory in immersive virtual environments." Hippocampus 32(9): 660-678. | wrong intervention | |
| 455 | Castilla, A., et al. (2022). "Age and sex impact on visuospatial working memory (VSWM), mental rotation, and cognitive strategies during navigation." NEUROSCIENCE RESEARCH 183: 84-96. | wrong intervention | |
| 456 | Castilla, D., et al. (2020). "Designing ICTs for Users with Mild Cognitive Impairment: A Usability Study." INTERNATIONAL JOURNAL OF ENVIRONMENTAL RESEARCH AND PUBLIC HEALTH 17(14). | wrong intervention | |
| 457 | Catalán, J. M., et al. (2023). "Patients' physiological reactions to competitive rehabilitation therapies assisted by robotic devices." JOURNAL OF NEUROENGINEERING AND REHABILITATION 20(1). | wrong intervention | |
| 458 | Catelan, A., et al. (2010). "Effect of artificial aging on the roughness and microhardness of sealed composites." J Esthet Restor Dent 22(5): 324-330. | wrong population | |
| 459 | Catissi, G., et al. (2024). "Nature-Based Interventions Targeting Elderly People's Health and Well-Being: An Evidence Map." Int J Environ Res Public Health 21(1). | wrong intervention | |
| 460 | Cavallo, V., et al. (2019). "A street-crossing simulator for studying and training pedestrians." Transportation Research Part F: Traffic Psychology and Behaviour 61: 217-228. | wrong intervention | |
| 461 | Cave, A. E., et al. (2019). "Efficacy of Cognition Support Formula® on cognitive function in older adults with subjective cognitive impairment: A protocol for a 26-week, randomised, double-blind, placebo-controlled trial." Trials 20(1). | wrong intervention | |
| 462 | Cave, A. E., et al. (2023). "A systematic review of the safety and efficacy on cognitive function of herbal and nutritional medicines in older adults with and without subjective cognitive impairment." Systematic Reviews 12(1). | wrong design | |
| 463 | Cave, L., et al. (2020). "Racial discrimination and child and adolescent health in longitudinal studies: A systematic review." Social Science and Medicine 250. | wrong design | |
| 464 | Cazzoli, B., et al. (2024). Enhancing Physical activity through Motivational stimuli in Augmented Reality. PROCEEDINGS OF THE INTERNATIONAL CONFERENCE ON ADVANCED VISUAL INTERFACES, AVI 2024. | wrong outcomes | |
| 465 | Cerdán-de-Las-Heras, J., et al. (2021). "Tele-Rehabilitation Program in Idiopathic Pulmonary Fibrosis-A Single-Center Randomized Trial." INTERNATIONAL JOURNAL OF ENVIRONMENTAL RESEARCH AND PUBLIC HEALTH 18(19). | wrong population | |
| 466 | Ceru, T., et al. (2017). "Geomorphological Dating of Pleistocene Conglomerates in Central Slovenia Based on Spatial Analyses of Dolines Using LiDAR and Ground Penetrating Radar." REMOTE SENSING 9(12). | wrong population | |
| 467 | Ceylan, E. and E. Gurbuz (2024). "The effect of virtual reality glasses on dental anxiety during scaling and root planing in patients with periodontitis: A randomized controlled clinical trial." INTERNATIONAL JOURNAL OF DENTAL HYGIENE 22(3): 749-757. | wrong population | |
| 468 | Cha, K. (2024). "The Moderating Role of Cortisol and Negative Emotionality in the Effects of Classroom Size and Window View on Young Children's Executive Functions." BEHAVIORAL SCIENCES 14(1). | wrong population | |
| 469 | Cha, K. J. (2023). "The Influence of Classroom Size and Window View on Young Children's Executive Functions and Physiological Responses, Based on VR Technology." BEHAVIORAL SCIENCES 13(11). | wrong population | |
| 470 | Chaby, L., et al. (2022). "Embodied Virtual Patients as a Simulation-Based Framework for Training Clinician-Patient Communication Skills: An Overview of Their Use in Psychiatric and Geriatric Care." FRONTIERS IN VIRTUAL REALITY 3. | wrong population | |
| 471 | Chai, R., et al. (2017). "Hybrid brain-computer interface for biomedical cyber-physical system application using wireless embedded EEG systems." BioMedical Engineering Online 16(1). | wrong population | |
| 472 | Chakraverty, A. P., et al. (2015). Sea Water Ageing of GFRP Composites and the Dissolved salts. 4TH NATIONAL CONFERENCE ON PROCESSING AND CHARACTERIZATION OF MATERIALS. 75. | wrong outcomes | |
| 473 | Chaleewong, N., et al. (2024). "Knowledge, attitudes, and perceived barriers regarding pain assessment and management among Thai critical care nurses: A cross-sectional study." INTENSIVE AND CRITICAL CARE NURSING 84. | wrong population | |
| 474 | Challands, K. G., et al. (2017). "Does online social connectedness buffer risk of depression following driving cessation? An analysis of older drivers and ex-drivers." Cyberpsychology, Behavior, and Social Networking 20(4): 232-237. | wrong population | |
| 475 | Cham, T. H., et al. (2024). "Virtually reality in tourism: adoption scepticism and resistance." TOURISM REVIEW 79(2): 337-354. | wrong population | |
| 476 | Chamberland, C., et al. (2024). "The effect of augmented reality on preoperative anxiety in children and adolescents: A randomized controlled trial." PEDIATRIC ANESTHESIA 34(2): 153-159. | wrong population | |
| 477 | Chan, C. L. F., et al. (2010). "Effect of the adapted virtual reality cognitive training program among Chinese older adults with chronic schizophrenia: A pilot study." International Journal of Geriatric Psychiatry 25(6): 643-649. | wrong design | |
| 478 | Chan, H. H. L., et al. (2024). "Does Freehand, Patient-specific Instrumentation or Surgical Navigation Perform Better for Allograft Reconstruction After Tumor Resection? A Preclinical Synthetic Bone Study." Clin Orthop Relat Res 482(10): 1896-1908. | wrong population | |
| 479 | Chan, J. Y. C., et al. (2020). "Effects of virtual reality on moods in community older adults. A multicenter randomized controlled trial." International Journal of Geriatric Psychiatry 35(8): 926-933. |  |  |
| 480 | Chan, S. H. M., et al. (2021). "Nature in virtual reality improves mood and reduces stress: evidence from young adults and senior citizens." Virtual Real: 1-16. | wrong design | |
| 481 | Chan, S. H. M., et al. (2023). "Nature in virtual reality improves mood and reduces stress: evidence from young adults and senior citizens." VIRTUAL REALITY 27(4): 3285-3300. | wrong design | |
| 482 | Chan, S. H. M., et al. (2023). "Understanding experiences in metaverse: How virtual nature impacts affect, pro-environmental attitudes, and intention to engage with physical nature." COMPUTERS IN HUMAN BEHAVIOR 149. | wrong design | |
| 483 | Chander, H., et al. (2022). "The Walls Are Closing In: Postural Responses to a Virtual Reality Claustrophobic Simulation." CLINICAL AND TRANSLATIONAL NEUROSCIENCE 6(2). | wrong population | |
| 484 | Chandrasiri, A., et al. (2020). "A virtual reality approach to mindfulness skills training." VIRTUAL REALITY 24(1): 143-149. | wrong design | |
| 485 | Chang, C. M., et al. (2012). "An interactive game-based shoulder wheel system for rehabilitation." PATIENT PREFERENCE AND ADHERENCE 6: 821-828. | wrong intervention | |
| 486 | Chang, L., et al. (2020). Augmented Reality Narratives for Post-Traumatic Stress Disorder Treatment. ADJUNCT PROCEEDINGS OF THE 2020 IEEE INTERNATIONAL SYMPOSIUM ON MIXED AND AUGMENTED REALITY (ISMAR-ADJUNCT 2020): 306-309. | wrong outcomes | |
| 487 | Chang, L.-C., et al. (2014). "Virtual reality improves sleep quality amongst older adults with disabilities." International Journal of Geriatric Psychiatry 29(12): 1312-1313. | wrong design | |
| 488 | Chang, Z. Y., et al. (2022). "Immersive Virtual Reality in Alleviating Pain and Anxiety in Children During Immunization in Primary Care: A Pilot Randomized Controlled Trial." FRONTIERS IN PEDIATRICS 10. | wrong population | |
| 489 | Channing, A. (2018). "A review of active hot-spring analogues of Rhynie: environments, habitats and ecosystems." PHILOSOPHICAL TRANSACTIONS OF THE ROYAL SOCIETY B-BIOLOGICAL SCIENCES 373(1739). | wrong population | |
| 490 | Chao, C. M. and T. K. Yu (2023). "Internet Use and Adolescents' Physical and Mental Health: the Mediating Role of Self-consciousness and Peer Relationships." INTERNATIONAL JOURNAL OF MENTAL HEALTH AND ADDICTION 21(2): 911-928. | wrong population | |
| 491 | Chao, C.-M. and T.-K. Yu (2021). "Internet use and adolescents’ physical and mental health: The mediating role of self-consciousness and peer relationships." International Journal of Mental Health and Addiction. | wrong population | |
| 492 | Chao, Y. P., et al. (2021). "Using a 360° Virtual Reality or 2D Video to Learn History Taking and Physical Examination Skills for Undergraduate Medical Students: Pilot Randomized Controlled Trial." JMIR SERIOUS GAMES 9(4). | wrong population | |
| 493 | Chao, Y. Y., et al. (2015). "Effects of Using Nintendo Wii™ Exergames in Older Adults: A Review of the Literature." JOURNAL OF AGING AND HEALTH 27(3): 379-402. | wrong design | |
| 494 | Chao, Y. Y., et al. (2015). "Physical and Psychosocial Effects of Wii Fit Exergames Use in Assisted Living Residents: A Pilot Study." CLINICAL NURSING RESEARCH 24(6): 589-603. | wrong design | |
| 495 | Charissis, V., et al. (2024). Virtual Rehabilitation: XR Design for Senior Users in Immersive Exergame Environments. 2024 IEEE GAMING, ENTERTAINMENT, AND MEDIA CONFERENCE, GEM 2024: 638-643. | wrong outcomes | |
| 496 | Chassiakos, Y. R., et al. (2016). "Children and Adolescents and Digital Media." PEDIATRICS 138(5). | wrong intervention | |
| 497 | Chaudhary, P., et al. (2019). "The effects of visuomotor training using Pablo system on hand grip strength and wrist movements in adults and elderly." Iranian Rehabilitation Journal 17(3): 215-224. | wrong population | |
| 498 | Chauhan, U., et al. (2018). Real-Time Stress Assessment Through PPG Sensor for VR Biofeedback. ICMI'18: PROCEEDINGS OF THE 20TH INTERNATIONAL CONFERENCE ON MULTIMODAL INTERACTION: ADJUNCT. | wrong outcomes | |
| 499 | Chauhan, V. S., et al. (2020). "Impact on anxiety of COVID-19 and lockdown." JOURNAL OF MARINE MEDICAL SOCIETY 22(3): 78-82. | wrong population | |
| 500 | Chauvel, H., et al. (2023). "<i>Changes in the empathy of fourth-year French medical students following a mandatory module on doctor-patient relationships</i>." ANNALES MEDICO-PSYCHOLOGIQUES 181(10): 871-879. | wrong population | |
| 501 | Chavant, M. and Z. Kapoula (2022). "Eye-Movement Deficits in Seniors with Hearing Aids: Cognitive and Multisensory Implications." Brain Sciences 12(11). | wrong intervention | |
| 502 | Chavkin, A. (2020). "A Family in Crisis: A Family Systems Theory Approach to Arthur Miller's "I Don't Need You Any More"." ARTHUR MILLER JOURNAL 15(2): 141-162. | wrong intervention | |
| 503 | Checa, J. and J. M. Aran (2020). "Reactive Oxygen Species: Drivers of Physiological and Pathological Processes." JOURNAL OF INFLAMMATION RESEARCH 13: 1057-1073. | wrong intervention | |
| 504 | Cheetham, M., et al. (2014). "Identifying with fictive characters: structural brain correlates of the personality trait 'fantasy'." SOCIAL COGNITIVE AND AFFECTIVE NEUROSCIENCE 9(11): 1836-1844. | wrong population | |
| 505 | Cheetham, T. J. and J. M. Turner-Cobb (2016). "Panel manipulation in social stress testing: The Bath Experimental Stress Test for Children (BEST-C)." PSYCHONEUROENDOCRINOLOGY 63: 78-85. | wrong population | |
| 506 | Chen, A. W., et al. (2018). "Selective Debridement With Labral Preservation Using Narrow Indications in the Hip: Minimum 5-Year Outcomes With a Matched-Pair Labral Repair Control Group." AMERICAN JOURNAL OF SPORTS MEDICINE 46(2): 297-304. | wrong population | |
| 507 | Chen, C., et al. (2020). "Biodegradable Zn-1.5Cu-1.5Ag alloy with anti-aging ability and strain hardening behavior for cardiovascular stents." Mater Sci Eng C Mater Biol Appl 116: 111172. | wrong population | |
| 508 | Chen, F. G., et al. (2024). "Mechanical Properties and Durability of Steel Slag-mineral Powder-coal Gangue Mixture by Uniform Design for Pavement Base." MATERIALS SCIENCE-MEDZIAGOTYRA 30(3): 388-395. | wrong population | |
| 509 | Chen, G. R., et al. (2021). "Educating Outpatients for Bowel Preparation Before Colonoscopy Using Conventional Methods vs Virtual Reality Videos Plus Conventional Methods A Randomized Clinical Trial." JAMA NETWORK OPEN 4(11). | wrong population | |
| 510 | Chen, H. J., et al. (2024). "Applying Microbial-Induced Calcium Carbonate Precipitation Technology to Improve the Bond Strength of Lightweight Aggregate Concrete after High-Temperature Damage." APPLIED SCIENCES-BASEL 14(4). | wrong intervention | |
| 511 | Chen, J., et al. (2018). An Emotion Management System via Face Tracking, Data Management, and Visualization. HCI INTERNATIONAL 2018 - POSTERS' EXTENDED ABSTRACTS, PT II. 851: 380-386. | wrong outcomes | |
| 512 | Chen, J., et al. (2023). "Observational evidence for the non-suppression effect of atmospheric chemical modification on the ice nucleation activity of East Asian dust." Sci Total Environ 861: 160708. | wrong design | |
| 513 | Chen, J. F., et al. (2014). "Effect of age-forming on corrosion properties of an Al-Zn-Mg-Cu alloy." MATERIALS AND CORROSION-WERKSTOFFE UND KORROSION 65(7): 670-677. | wrong population | |
| 514 | Chen, J. Q., et al. (2011). STEAM GENERATOR AGEING MANAGEMENT DATABASE. PROCEEDINGS OF THE 18TH INTERNATIONAL CONFERENCE ON NUCLEAR ENGINEERING 2010, VOL 5: 375-378. | wrong outcomes | |
| 515 | Chen, N., et al. (2021). "Experimental Study on the Evaluation and Influencing Factors on Individual's Emergency Escape Capability in Subway Fire." INTERNATIONAL JOURNAL OF ENVIRONMENTAL RESEARCH AND PUBLIC HEALTH 18(19). | wrong population | |
| 516 | Chen, P. Y., et al. (2012). "Interactive wiimote gaze stabilization exercise training system for patients with vestibular hypofunction." JOURNAL OF NEUROENGINEERING AND REHABILITATION 9. | wrong population | |
| 517 | Chen, Y. E., et al. (2016). "Effect of salicylic acid on the antioxidant system and photosystem II in wheat seedlings." BIOLOGIA PLANTARUM 60(1): 139-147. | wrong population | |
| 518 | Chen, Y. J., et al. (2023). "Effects of virtual reality on preoperative anxiety in children: A systematic review and meta-analysis of randomised controlled trials." JOURNAL OF CLINICAL NURSING 32(11-12): 2494-2504. | wrong design | |
| 519 | Cheng, C. and O. Ebrahimi (2023). "A meta-analytic review of gamified interventions in mental health enhancement." COMPUTERS IN HUMAN BEHAVIOR 141. | wrong design | |
| 520 | Cheng, V. Y., et al. (2020). "Combination of 3-Dimensional Virtual Reality and Hands-On Aromatherapy in Improving Institutionalized Older Adults' Psychological Health: Quasi-Experimental Study." J Med Internet Res 22(7): e17096. | wrong design | |
| 521 | Cheng, V. Y. W., et al. (2020). "Combination of 3-Dimensional Virtual Reality and Hands-On Aromatherapy in Improving Institutionalized Older Adults' Psychological Health: Quasi-Experimental Study." JOURNAL OF MEDICAL INTERNET RESEARCH 22(7). | duplicate |  |
| 522 | Cheng, V. Y.-W., et al. (2020). "Combination of 3-Dimensional Virtual Reality and Hands-On Aromatherapy in Improving Institutionalized Older Adults' Psychological Health: Quasi-Experimental Study." Journal of Medical Internet Research 22(7): N.PAG-N.PAG. | duplicate |  |
| 523 | Cheng, W. H., et al. (2010). Applying Situation Awareness Approach to Cooperative Play in Interactive Installation Storytelling System. COOPERATIVE DESIGN, VISUALIZATION, AND ENGINEERING. 6240: 31-+. | wrong population | |
| 524 | Cherniack, E. P. (2011). "Not just fun and games: Applications of virtual reality in the identification and rehabilitation of cognitive disorders of the elderly." Disability and Rehabilitation: Assistive Technology 6(4): 283-289. | wrong design | |
| 525 | Chessa, M., et al. (2019). Human-Computer Interaction Approaches for the Assessment and the Practice of the Cognitive Capabilities of Elderly People. COMPUTER VISION - ECCV 2018 WORKSHOPS, PT VI. 11134: 66-81. | wrong design | |
| 526 | Ching, S. M., et al. (2024). "Prevalence and factors associated with burnout among healthcare providers in Malaysia: a web-based cross-sectional study." Ir J Med Sci 193(2): 851-863. | wrong population | |
| 527 | Chipperfield, S. R. and P. Bissell (2023). ""I Hear the Music and My Spirits Lift!" Pleasure and Ballroom Dancing for Community-Dwelling Older Adults." J Aging Phys Act 31(2): 276-288. | wrong intervention | |
| 528 | Chirico, A., et al. (2024). "Exploring the Psychological Nexus of Virtual and Augmented Reality on Physical Activity in Older Adults: A Rapid Review." BEHAVIORAL SCIENCES 14(1). | wrong design | |
| 529 | Chirico, A., et al. (2020). "Virtual reality for the assessment of everyday cognitive functions in older adults: An evaluation of the Virtual Reality Action Test and two interaction devices in a 91-year-old woman." Frontiers in Psychology 11: 12. | wrong design | |
| 530 | Chirico, A., et al. (2022). "Inspiring awe in high school teachers: Design and preliminary test of a virtual training on Altspace VR." ANNUAL REVIEW OF CYBERTHERAPY AND TELEMEDICINE 20: 31-35. | wrong population | |
| 531 | Chitlange, N. M. and V. Yadav (2023). "Impact of Controlled Breathing Techniques in Virtual Reality Environments on the Psychological Status of Oral Cancer Patients: A Case Report." CUREUS JOURNAL OF MEDICAL SCIENCE 15(12). | wrong population | |
| 532 | Chiu, C. C., et al. (2023). "Immersive Virtual Reality to Distract From Pain in Children Treated With L-asparaginase by Intramuscular Injection." CUREUS JOURNAL OF MEDICAL SCIENCE 15(1). | wrong population | |
| 533 | Chiu, H. M., et al. (2023). "Effects of incorporating virtual reality training intervention into health care on cognitive function and wellbeing in older adults with cognitive impairment: A randomized controlled trial." INTERNATIONAL JOURNAL OF HUMAN-COMPUTER STUDIES 170. | wrong intervention | |
| 534 | Chiu, H.-M., et al. (2023). "Effects of incorporating virtual reality training intervention into health care on cognitive function and wellbeing in older adults with cognitive impairment: A randomized controlled trial." International Journal of Human-Computer Studies 170: 1-12. | duplicate |  |
| 535 | Chiu, H. Y., et al. (2018). "Reality orientation therapy benefits cognition in older people with dementia: A meta-analysis." Int J Nurs Stud 86: 20-28. | wrong design | |
| 536 | Chiu, H.-Y., et al. (2018). "Reality orientation therapy benefits cognition in older people with dementia: A meta-analysis." International Journal of Nursing Studies 86: 20-28. | wrong design | |
| 537 | Chiu, P. L., et al. (2023). "Virtual Reality-Based Intervention to Reduce Preoperative Anxiety in Adults Undergoing Elective Surgery: A Randomized Clinical Trial." JAMA Netw Open 6(10): e2340588. | wrong population | |
| 538 | Chiu, P. L., et al. (2023). "Virtual Reality–Based Intervention to Reduce Preoperative Anxiety in Adults Undergoing Elective Surgery A Randomized Clinical Trial." JAMA Network Open: E2340588. | duplicate |  |
| 539 | Chiu, P. L., et al. (2023). "Virtual Reality–Based Intervention to Reduce Preoperative Anxiety in Adults Undergoing Elective Surgery: A Randomized Clinical Trial." JAMA Network Open 6(10): e2340588-e2340588. | duplicate |  |
| 540 | Chiu, P. L., et al. (2023). "Virtual Reality-Based Intervention to Reduce Preoperative Anxiety in Adults Undergoing Elective Surgery A Randomized Clinical Trial." JAMA NETWORK OPEN 6(10). | duplicate |  |
| 541 | Chiulan, I., et al. (2020). "Comprehensive characterization of silica-modified silicon rubbers." J Mech Behav Biomed Mater 101: 103427. | wrong population | |
| 542 | Chockanathan, U. and K. Padmanabhan (2024). "Differential disruptions in population coding along the dorsal-ventral axis of CA1 in the APP/PS1 mouse model of Aβ pathology." PLOS COMPUTATIONAL BIOLOGY 20(5). | wrong population | |
| 543 | Choi, C., et al. (2024). "Virtual Golf, "Exergaming", Using Virtual Reality for Healthcare in Older Adults: Focusing on Leisure Constraints, Participation Benefits, and Continuous Participation Intention." HEALTHCARE 12(10). | wrong outcomes | |
| 544 | Choi, C., et al. (2024). "Virtual Golf, "Exergaming", Using Virtual Reality for Healthcare in Older Adults: Focusing on Leisure Constraints, Participation Benefits, and Continuous Participation Intention." Healthcare (2227-9032) 12(10): 962. | wrong outcomes | |
| 545 | Choi, S. U., et al. (2019). "Comparison of vision-related quality of life and mental health between congenital and acquired low-vision patients." EYE 33(10): 1540-1546. | wrong population | |
| 546 | Choo, K. T. W., et al. (2016). Empath-D: Empathetic Design for Accessibility. HOTMOBILE'17: PROCEEDINGS OF THE 18TH INTERNATIONAL WORKSHOP ON MOBILE COMPUTING SYSTEMS AND APPLICATIONS: 55-60. | wrong outcomes | |
| 547 | Chopik, W. J. (2016). "The benefits of social technology use among older adults are mediated by reduced loneliness." Cyberpsychology, Behavior, and Social Networking 19(9): 551-556. | wrong intervention | |
| 548 | Chopra, H. K., et al. (2023). "Angiotensin Receptor-Neprilysin Inhibitor Therapy and Cardiac Remodeling in Heart Failure: Consensus Statement from India." J Assoc Physicians India 71(4): 11-12. | wrong population | |
| 549 | Chou, P. H., et al. (2021). "Efficacy and acceptability of different interventions for acrophobia: A network meta-analysis of randomised controlled trials." J Affect Disord 282: 786-794. | wrong design | |
| 550 | Chou, P.-H., et al. (2021). "Efficacy and acceptability of different interventions for acrophobia: A network meta-analysis of randomised controlled trials." Journal of Affective Disorders 282: 786-794. | wrong design | |
| 551 | Chou, Y. H., et al. (2023). "Potential Mobile Health Applications for Improving the Mental Health of the Elderly: A Systematic Review." Clin Interv Aging 18: 1523-1534. | wrong design | |
| 552 | Choukou, M. A., et al. (2023). "Feasibility of a Virtual-Reality-Enabled At-Home Telerehabilitation Program for Stroke Survivors: A Case Study." JOURNAL OF PERSONALIZED MEDICINE 13(8). | wrong design | |
| 553 | Chu, X. L., et al. (2024). "Case report: Virtual reality-based arm and leg cycling combined with transcutaneous electrical spinal cord stimulation for early treatment of a cervical spinal cord injured patient." FRONTIERS IN NEUROSCIENCE 18. | wrong design | |
| 554 | Chua, S. I. L., et al. (2019). "Virtual reality for screening of cognitive function in older persons: Comparative study." Journal of Medical Internet Research 21(8): 14. | wrong outcomes | |
| 555 | Chua, S. I. L., et al. (2019). "Virtual Reality for Screening of Cognitive Function in Older Persons: Comparative Study." Journal of Medical Internet Research 21(8): N.PAG-N.PAG. | wrong outcomes | |
| 556 | Chuan, A., et al. (2024). "Using Virtual Reality to teach ultrasound-guided needling skills for regional anaesthesia: A randomised controlled trial." JOURNAL OF CLINICAL ANESTHESIA 97. | wrong population | |
| 557 | Chuang, C. S., et al. (2022). "Effects of modern technology (exergame and virtual reality) assisted rehabilitation vs conventional rehabilitation in patients with Parkinson?s disease: a network meta-analysis of randomised controlled trials." PHYSIOTHERAPY 117: 35-42. | wrong design | |
| 558 | Chueng, K. F., et al. (2024). "Paleobiogeoclimatic reconstruction of the Monte Cristo cave, Minas Gerais, Brazil, through phytolith analysis." JOURNAL OF SOUTH AMERICAN EARTH SCIENCES 133. | wrong intervention | |
| 559 | Chung, J. S. (2014). "An insulin-like growth factor found in hepatopancreas implicates carbohydrate metabolism of the blue crab Callinectes sapidus." Gen Comp Endocrinol 199: 56-64. | wrong population | |
| 560 | Chung, O. S., et al. (2022). "Are Australian Mental Health Services Ready for Therapeutic Virtual Reality? An Investigation of Knowledge, Attitudes, Implementation Barriers and Enablers." FRONTIERS IN PSYCHIATRY 13. | wrong intervention | |
| 561 | Chung, O. S., et al. (2022). "Implementation of Therapeutic Virtual Reality Into Psychiatric Care: Clinicians' and Service Managers' Perspectives." FRONTIERS IN PSYCHIATRY 12. | wrong intervention | |
| 562 | Churchill, R., et al. (2023). "Feasibility of an Interdisciplinary Intervention to Promote Balance Confidence in Lower-Limb Prosthesis Users: A Case Study." JOURNAL OF PROSTHETICS AND ORTHOTICS 35(2): E73-E80. | wrong design | |
| 563 | Cicconi, S. and M. Marchese (2019). AUGMENTED LEARNING: AN E-LEARNING ENVIRONMENT IN AUGMENTED REALITY FOR OLDER ADULTS. 13TH INTERNATIONAL TECHNOLOGY, EDUCATION AND DEVELOPMENT CONFERENCE (INTED2019): 3652-3662. | wrong outcomes | |
| 564 | Cieślik, B., et al. (2023). "Immersive virtual reality as support for the mental health of elderly women: a randomized controlled trial." Virtual Reality 27(3): 2227-2235. |  |  |
| 565 | Cilindre, C., et al. (2010). "Foaming properties of various Champagne wines depending on several parameters: Grape variety, aging, protein and CO2 content." Analytica Chimica Acta 660(1-2): 164-170. | wrong intervention | |
| 566 | Cinalioglu, K., et al. (2023). "Effects of virtual reality guided meditation in older adults: the protocol of a pilot randomized controlled trial." FRONTIERS IN PSYCHOLOGY 14. | wrong design | |
| 567 | Cioffi, R. and A. V. Lubetzky (2023). "<i>BOXVR</i> Versus Guided YouTube Boxing for Stress, Anxiety, and Cognitive Performance in Adolescents: A Pilot Randomized Controlled Trial." GAMES FOR HEALTH JOURNAL 12(3): 259-268. | wrong population | |
| 568 | Clark, J. L. (2011). "The evolution of human culture during the later Pleistocene: Using fauna to test models on the emergence and nature of "modern" human behavior." JOURNAL OF ANTHROPOLOGICAL ARCHAEOLOGY 30(3): 273-291. | wrong population | |
| 569 | Claydon, V. E., et al. (2019). "Evaluation of forearm vascular resistance during orthostatic stress: Velocity is proportional to flow and size doesn't matter." PLOS ONE 14(11). | wrong population | |
| 570 | Clemenson, G. D., et al. (2020). "Enriching hippocampal memory function in older adults through video games." Behavioural Brain Research 390: 7. | wrong intervention | |
| 571 | Clemente, D., et al. (2023). "Forest therapy using virtual reality in the older population: a systematic review." Front Psychol 14: 1323758. | wrong design | |
| 572 | Clemente, D., et al. (2024). "Forest therapy using virtual reality in the older population: a systematic review." FRONTIERS IN PSYCHOLOGY 14. | wrong design | |
| 573 | Clerc, P. G. B., et al. (2021). "A Randomized Controlled Trial of Virtual Reality in Awake Minor Pediatric Plastic Surgery Procedures." PLASTIC AND RECONSTRUCTIVE SURGERY 148(2): 400-408. | wrong population | |
| 574 | Climent, G., et al. (2021). "New virtual reality tool (Nesplora Aquarium) for assessing attention and working memory in adults: A normative study." APPLIED NEUROPSYCHOLOGY-ADULT 28(4): 403-415. | wrong population | |
| 575 | Close, R., et al. (2022). "VIRTUAL REALITY HEADSET AS AN ALTERNATIVE TO ENTONOX FOR INTRAARTICULAR CORTICOSTEROID INJECTION." Rheumatology Advances in Practice 6: i43. | wrong outcomes | |
| 576 | Coelho, T., et al. (2020). "Promoting Reminiscences with Virtual Reality Headsets: A Pilot Study with People with Dementia." INTERNATIONAL JOURNAL OF ENVIRONMENTAL RESEARCH AND PUBLIC HEALTH 17(24). | wrong design | |
| 577 | Cœugnet, S., et al. (2017). "A vibrotactile wristband to help older pedestrians make safer street-crossing decisions." Accident Analysis and Prevention 109: 1-9. | wrong intervention | |
| 578 | Coid, J. W., et al. (2013). "The Relationship Between Delusions and Violence <i>Findings From the East London First Episode Psychosis Study</i>." JAMA PSYCHIATRY 70(5): 465-471. | wrong intervention | |
| 579 | Coldham, G., et al. (2017). VR Usability from Elderly Cohorts: Preparatory Challenges in Overcoming Technology Rejection. 2017 NATIONAL INFORMATION TECHNOLOGY CONFERENCE (NITC): 131-135. | wrong outcomes | |
| 580 | Cole, E., et al. (2023). "Short-term heat acclimation protocols for an aging population: Systematic review." PLoS One 18(3): e0282038. | wrong design | |
| 581 | Cole, R., et al. (2023). "Operation Bushmaster's Impact on Military Medical Student Deployment Readiness." MILITARY MEDICINE 188: 56-62. | wrong population | |
| 582 | Coleman-Belin, J., et al. (2023). "Aging Effects on Optic Nerve Neurodegeneration." Int J Mol Sci 24(3). | wrong intervention | |
| 583 | Collado-Mateo, D., et al. (2017). "Effects of Exergames on Quality of Life, Pain, and Disease Effect in Women With Fibromyalgia: A Randomized Controlled Trial." ARCHIVES OF PHYSICAL MEDICINE AND REHABILITATION 98(9): 1725-1731. | wrong population | |
| 584 | Collij, L. E., et al. (2023). "Quantification of [(18) F]florbetaben amyloid-PET imaging in a mixed memory clinic population: The ABIDE project." Alzheimers Dement 19(6): 2397-2407. | wrong population | |
| 585 | Colò, G., et al. (2020). "The efficacy of shoe modifications and foot orthoses in treating patients with hallux rigidus: a comprehensive review of literature." Acta Biomed 91(14-s): e2020016. | wrong design | |
| 586 | Colò, G., et al. (2021). "May footwear be a predisposing factor for the development of hallux rigidus? A review of recent findings." Acta Biomed 92(S3): e2021010. | wrong design | |
| 587 | Colombini, G., et al. (2021). "LEAP Motion Technology and Psychology: A Mini-Review on Hand Movements Sensing for Neurodevelopmental and Neurocognitive Disorders." INTERNATIONAL JOURNAL OF ENVIRONMENTAL RESEARCH AND PUBLIC HEALTH 18(8). | wrong design | |
| 588 | Combalia, A., et al. (2024). "Immersive virtual reality in orthopaedics-a narrative review." INTERNATIONAL ORTHOPAEDICS 48(1): 21-30. | wrong design | |
| 589 | Connors, E. C., et al. (2014). "Virtual environments for the transfer of navigation skills in the blind: a comparison of directed instruction vs. video game based learning approaches." FRONTIERS IN HUMAN NEUROSCIENCE 8. | wrong intervention | |
| 590 | Cook, N. E., et al. (2021). "Safety and Tolerability of an Innovative Virtual Reality-Based Deep Breathing Exercise in Concussion Rehabilitation: A Pilot Study." DEVELOPMENTAL NEUROREHABILITATION 24(4): 222-229. | wrong population | |
| 591 | Corella, D., et al. (2014). "MicroRNA-410 regulated lipoprotein lipase variant rs13702 is associated with stroke incidence and modulated by diet in the randomized controlled PREDIMED trial." Am J Clin Nutr 100(2): 719-731. | wrong population | |
| 592 | Corey, R. M., et al. (2022). "Factors Associated With Pain and Function Before Medial Patellofemoral Ligament Reconstruction." ORTHOPAEDIC JOURNAL OF SPORTS MEDICINE 10(8). | wrong population | |
| 593 | Corneliusson, L., et al. (2019). "Residing in sheltered housing versus ageing in place - Population characteristics, health status and social participation." Health Soc Care Community 27(4): e313-e322. | wrong population | |
| 594 | Corno, G., et al. (2014). "Usability assessment of the Virtual Multitasking Test (V-MT) for elderly people." Annual Review of CyberTherapy and Telemedicine 12: 168-172. | wrong population | |
| 595 | Cornwell, B. R., et al. (2010). "Abnormal Hippocampal Functioning and Impaired Spatial Navigation in Depressed Individuals: Evidence From Whole-Head Magnetoencephalography." AMERICAN JOURNAL OF PSYCHIATRY 167(7): 836-844. | wrong intervention | |
| 596 | Corriette, B., et al. (2023). Using VR to Elicit Empathy in Current and Future Psychiatrists for their Patients of Color. 2023 IEEE CONFERENCE ON VIRTUAL REALITY AND 3D USER INTERFACES ABSTRACTS AND WORKSHOPS, VRW: 187-190. | wrong outcomes | |
| 597 | Corrigan, N., et al. (2023). "Immersive virtual reality for improving cognitive deficits in children with ADHD: a systematic review and meta-analysis." VIRTUAL REALITY 27(4): 3545-3564. | wrong population | |
| 598 | Cortés-Pérez, I., et al. (2021). "Virtual Reality-Based Therapy Improves Fatigue, Impact, and Quality of Life in Patients with Multiple Sclerosis. A Systematic Review with a Meta-Analysis." SENSORS 21(21). | wrong design | |
| 599 | Cosco, T. D., et al. (2017). "Education and successful aging trajectories: A longitudinal population-based latent variable modelling analysis." Canadian Journal on Aging 36(4): 427-434. | wrong population | |
| 600 | Costa, M. T. S., et al. (2019). "Virtual Reality-Based Exercise with Exergames as Medicine in Different Contexts: A Short Review." Clin Pract Epidemiol Ment Health 15: 15-20. | wrong design | |
| 601 | Costa, M. T. S., et al. (2019). "Virtual reality-based exercise with exergames as medicine in different contexts: A short review." Clinical Practice and Epidemiology in Mental Health 15: 6. | wrong design | |
| 602 | Costa, P. V. M., et al. (2024). "The effect of the simulated aging by thermocycling on the elastic modulus of ethylene-vinyl acetate brands and stress/strain development during an impact: An in vitro and 3D-FEA analysis." Dent Traumatol 40(2): 204-212. | wrong design | |
| 603 | Costanzo, M. G., et al. (2023). "Virtual reality for the assessment and treatment of cognitive impairment in the elderly: A scoping review." Life Span and Disability 26(2): 293-328. | wrong intervention | |
| 604 | Cottingham, A. H., et al. (2022). "ASPIRE: A Program for Developing Clinician Educators' Scholarship, Advancement, and Sense of Community." J Gen Intern Med 37(8): 1953-1962. | wrong design | |
| 605 | Coulibaly, I., et al. (2022). "Virtual Reality Hypnosis in the Electrophysiology Lab: When Human Treatments Are Better than Virtual Ones." JOURNAL OF CLINICAL MEDICINE 11(13). | wrong outcomes | |
| 606 | Coventry, P. A., et al. (2020). "Psychological and pharmacological interventions for posttraumatic stress disorder and comorbid mental health problems following complex traumatic events: Systematic review and component network meta-analysis." PLOS MEDICINE 17(8). | wrong population | |
| 607 | Cowley, T., et al. (2016). "Evaluation of undergraduate nursing students' clinical confidence following a mental health recovery camp." INTERNATIONAL JOURNAL OF MENTAL HEALTH NURSING 25(1): 33-41. | wrong design | |
| 608 | Coyle, H., et al. (2015). "Computerized and Virtual Reality Cognitive Training for Individuals at High Risk of Cognitive Decline: Systematic Review of the Literature." AMERICAN JOURNAL OF GERIATRIC PSYCHIATRY 23(4): 335-359. | wrong population | |
| 609 | Cozzi, G., et al. (2021). "Distraction Using Buzzy or Handheld Computers During Venipuncture." PEDIATRIC EMERGENCY CARE 37(9): E512-E516. | wrong design | |
| 610 | Craig, M., et al. (2015). "Rest boosts the long-term retention of spatial associative and temporal order information." Hippocampus 25(9): 1017-1027. | wrong population | |
| 611 | Craig, M., et al. (2015). "Rest boosts the long‐term retention of spatial associative and temporal order information." Hippocampus 25(9): 1017-1027. | wrong population | |
| 612 | Craig, S. D. and N. L. Schroeder (2017). "Reconsidering the voice effect when learning from a virtual human." Computers & Education 114: 193-205. | wrong population | |
| 613 | Craig, T. V., et al. (2024). "Examining and Comparing the Energy Expenditure of Two Modes of a Virtual Reality Fitness Game (Supernatural): Indirect Calorimetry Study." JMIR SERIOUS GAMES 12. | wrong outcomes | |
| 614 | Crane, B. M., et al. (2023). "Older Adults and Three-Dimensional Exergaming: Motivators and Barriers to Participation and Retention." Games Health J 12(2): 150-157. | wrong design | |
| 615 | Crawford, M. A., et al. (2014). "Nutritional Armor in Evolution: Docosahexaenoic Acid as a Determinant of Neural, Evolution and Hominid Brain Development." MILITARY MEDICINE 179(11): 61-75. | wrong population | |
| 616 | Crevatin, F., et al. (2016). "Hand-held computers can help to distract children undergoing painful venipuncture procedures." ACTA PAEDIATRICA 105(8): 930-934. | wrong population | |
| 617 | Crevecoeur, I., et al. (2010). "The Spy VI child: A newly discovered Neandertal infant." JOURNAL OF HUMAN EVOLUTION 59(6): 641-656. | wrong population | |
| 618 | Croft, J. L., et al. (2013). "Responses to Sudden Cold-Water Immersion in Inexperienced Swimmers Following Training." AVIATION SPACE AND ENVIRONMENTAL MEDICINE 84(8): 850-855. | wrong population | |
| 619 | Croghan, I. T., et al. (2022). "Virtual Reality for Health Care Professionals During a Pandemic: A Pilot Program." JOURNAL OF PRIMARY CARE AND COMMUNITY HEALTH 13. | wrong design | |
| 620 | Crone, C. L. and R. W. Kallen (2022). "Interview with an avatar: Comparing online and virtual reality perspective taking for gender bias in STEM hiring decisions." PLOS ONE 17(6). | wrong population | |
| 621 | Crooms, R. C. and L. P. Gelfman (2020). "Palliative Care and End-of-Life Considerations for the Frail Patient." Anesth Analg 130(6): 1504-1515. | wrong population | |
| 622 | Cuffaro, M., et al. (2016). "The Ventotene Volcanic Ridge: a newly explored complex in the central Tyrrhenian Sea (Italy)." BULLETIN OF VOLCANOLOGY 78(12). | wrong population | |
| 623 | Cui, Y. J., et al. (2018). Research on Influence of Mechanical Stress on Insulation Characteristics of Oil-immersed Pressboard. 2018 IEEE INTERNATIONAL POWER MODULATOR AND HIGH VOLTAGE CONFERENCE (IPMHVC): 264-268. | wrong outcomes | |
| 624 | Cumerlato, M., et al. (2017). "Effect of surface treatment of prefabricated teeth on shear bond strength of orthodontic brackets." Dental Press J Orthod 22(4): 47-52. | wrong population | |
| 625 | Cummings, J., et al. (2015). "Agitation in cognitive disorders: International Psychogeriatric Association provisional consensus clinical and research definition." Int Psychogeriatr 27(1): 7-17. | wrong design | |
| 626 | Cupitra, N. I., et al. (2020). "Influence of Ageing on Vascular Reactivity and Receptor Expression in Rabbit Aorta: A Complement to Elastocalcinosis and Smooth Muscle Mechanisms." CLINICAL INTERVENTIONS IN AGING 15: 537-545. | wrong design | |
| 627 | Curtin, A., et al. (2019). "Coping with mental health issues among older Hispanic adults." Geriatr Nurs 40(2): 123-128. | wrong intervention | |
| 628 | Custódio, N. B., et al. (2021). "Efficacy of audiovisual distraction using eyeglasses during dental care: a randomized clinical trial." BRAZILIAN ORAL RESEARCH 35. | wrong intervention | |
| 629 | Custódio, N. B., et al. (2020). "Effectiveness of Virtual Reality Glasses as a Distraction for Children During Dental Care." PEDIATRIC DENTISTRY 42(2): 91-+. | wrong population | |
| 630 | Czaja, S. J. and M. Ceruso (2022). "The promise of Artificial Intelligence in supporting an aging population." Journal of Cognitive Engineering and Decision Making 16(4): 182-193. | wrong intervention | |
| 631 | Czech, O., et al. (2023). "Virtual reality in chemotherapy support for the treatment of physical functions, fear, and quality of life in pediatric cancer patients: A systematic review and meta-analysis." FRONTIERS IN PUBLIC HEALTH 11. | wrong design | |
| 632 | Czub, M., et al. (2024). "Virtual Reality Distraction for Needle-Related Pain and Distress in Children: A Multicenter Randomized Controlled Trial." CYBERPSYCHOLOGY BEHAVIOR AND SOCIAL NETWORKING 27(6): 409-419. | wrong population | |
| 633 | D’Alessio, A. (2023). "Unraveling the Cave: A Seventy-Year Journey into the Caveolar Network, Cellular Signaling, and Human Disease." Cells 12(23). | wrong design | |
| 634 | da Costa, R. Q. M., et al. (2022). "Two immersive virtual reality tasks for the assessment of spatial orientation in older adults with and without cognitive impairment: Concurrent validity, group comparison, and accuracy results." Journal of the International Neuropsychological Society 28(5): 460-472. | wrong design | |
| 635 | da Fonseca, R. S. A., et al. (2023). "Mechanical Power in Prone Position Intubated Patients with COVID-19-Related ARDS: A Cohort Study." CRITICAL CARE RESEARCH AND PRACTICE 2023. | wrong population | |
| 636 | da Rocha, M. L., et al. (2021). "Accelerated aging effects in composites used as repair for pipes in oil industry." POLYMER COMPOSITES 42(11): 5918-5929. | wrong population | |
| 637 | Da Rocha, P. A., et al. (2015). "Complementary physical therapies for movement disorders in Parkinson's disease: a systematic review." EUROPEAN JOURNAL OF PHYSICAL AND REHABILITATION MEDICINE 51(6): 693-704. | wrong design | |
| 638 | da Silva, E. M., et al. (2014). "Stability of the bond between two resin cements and an yttria-stabilized zirconia ceramic after six months of aging in water." J Prosthet Dent 112(3): 568-575. | wrong intervention | |
| 639 | Da Silva Júnior, J. L. A., et al. (2021). "A bowling exergame to improve functional capacity in older adults: Co-design, development, and testing to compare the progress of playing alone versus playing with peers." JMIR Serious Games 9(1): 13. | wrong intervention | |
| 640 | Daegling, D. J., et al. (2014). "Geometric properties and comparative biomechanics of <i>Homo floresiensis</i> mandibles." JOURNAL OF HUMAN EVOLUTION 68: 36-46. | wrong intervention | |
| 641 | Dahms, R., et al. (2019). Developing a VR Training Program for Geriatric Patients with Chronic Back Pain A Process Analysis. VIRTUAL, AUGMENTED AND MIXED REALITY: APPLICATIONS AND CASE STUDIES, VAMR 2019, PT II. 11575: 243-255. | wrong outcomes | |
| 642 | Dalal, V. K. and B. C. Tripathy (2012). "Modulation of chlorophyll biosynthesis by water stress in rice seedlings during chloroplast biogenesis." PLANT CELL AND ENVIRONMENT 35(9): 1685-1703. | wrong intervention | |
| 643 | Dalberny, C. and J. Mauro (2012). B-LEARNING EXPERIENCE IN HIGHER EDUCATION: STRENGHT AND WEAKNESS. EDULEARN12: 4TH INTERNATIONAL CONFERENCE ON EDUCATION AND NEW LEARNING TECHNOLOGIES: 6727-6729. | wrong outcomes | |
| 644 | Daminov, I., et al. (2021). "Energy limit of oil-immersed transformers: A concept and its application in different climate conditions." IET GENERATION TRANSMISSION & DISTRIBUTION 15(3): 495-507. | wrong design | |
| 645 | Dangare, M. and V. Yadav (2023). "Gaming on an Immersive Virtual Reality Platform to Ameliorate the Level of Anxiety in Patients Undergoing Congenital Heart Disease." CUREUS JOURNAL OF MEDICAL SCIENCE 15(12). | wrong population | |
| 646 | D'Antonio, E., et al. (2020). "Stable or able? Effect of virtual reality stimulation on static balance of post-stroke patients and healthy subjects." HUMAN MOVEMENT SCIENCE 70. | wrong population | |
| 647 | Dapschauskas, R., et al. (2022). "The Emergence of Habitual Ochre Use in Africa and its Significance for The Development of Ritual Behavior During The Middle Stone Age." JOURNAL OF WORLD PREHISTORY 35(3-4): 233-319. | wrong intervention | |
| 648 | Darnall, B. D., et al. (2020). "Self-Administered Skills-Based Virtual Reality Intervention for Chronic Pain: Randomized Controlled Pilot Study." JMIR FORMATIVE RESEARCH 4(7). | wrong population | |
| 649 | Davey, A. P., et al. (2024). "Patient-Reported Outcomes of Total Hip Arthroplasty at an Ambulatory Surgery Center Versus a Hospital-Based Center." JOURNAL OF THE AMERICAN ACADEMY OF ORTHOPAEDIC SURGEONS GLOBAL RESEARCH AND REVIEWS 8(6). | wrong intervention | |
| 650 | Davies, H., et al. (2020). "'Ward for the day': A case study of extended immersive ward-based simulation." NURSE EDUCATION TODAY 90. | wrong population | |
| 651 | Davis, A., et al. (2016). "Aging and Hearing Health: The Life-course Approach." The Gerontologist 56: S256-S267. | wrong design | |
| 652 | Davis, L. L., et al. (2020). "The methods and baseline characteristics of a VA randomized controlled study evaluating supported employment provided in primary care patient aligned care teams." BMC MEDICAL RESEARCH METHODOLOGY 20(1). | wrong population | |
| 653 | Davis, M., et al. (2022). "Collaboration Between Mental Health and Vocational Rehabilitation Programs for Transition-Age Youth Vocational Outcomes." PSYCHIATRIC REHABILITATION JOURNAL 45(4): 303-313. | wrong population | |
| 654 | Davis, R. and J. Ohman (2016). "Wayfinding in ageing and Alzheimer's disease within a virtual senior residence: Study protocol." Journal of Advanced Nursing 72(7): 1677-1688. | wrong design | |
| 655 | Davis, R., et al. (2017). "Salient Cues and Wayfinding in Alzheimer's Disease Within a Virtual Senior Residence." ENVIRONMENT AND BEHAVIOR 49(9): 1038-1065. | wrong population | |
| 656 | Davis, R. and A. Sikorskii (2020). "Eye tracking analysis of visual cues during wayfinding in early stage Alzheimer’s disease." Dementia and Geriatric Cognitive Disorders 49(1): 91-97. | wrong intervention | |
| 657 | Davis, R. L. and C. Weisbeck (2015). "Search Strategies Used by Older Adults in a Virtual Reality Place Learning Task." GERONTOLOGIST 55: S118-S127. | wrong design | |
| 658 | D'Cunha, N. M., et al. (2024). "A pilot study of an intergenerational program for people in residential aged care with cognitive impairment and children from a co-located early learning centre during COVID-19." Dementia (London, England) 23(6): 927-948. | wrong population | |
| 659 | D'Cunha, N. M., et al. (2019). "A Mini-Review of Virtual Reality-Based Interventions to Promote Well-Being for People Living with Dementia and Mild Cognitive Impairment." GERONTOLOGY 65(4): 430-440. | wrong design | |
| 660 | de Abreu, N. M. R., et al. (2023). "Influence of carbonated acid beverage on fracture resistance and marginal gap formation in different restorative approaches to non-carious cervical lesions." Clin Oral Investig 27(5): 2245-2253. | wrong intervention | |
| 661 | de Campos, M. F., et al. (2019). Raynaud's Phenomenon Differentiating After Cold Stress Using Thermal Parameters from Fingers. XXVI BRAZILIAN CONGRESS ON BIOMEDICAL ENGINEERING, CBEB 2018, VOL. 2. 70: 869-874. | wrong intervention | |
| 662 | De Cock, R., et al. (2014). "Compulsive use of social networking sites in Belgium: Prevalence, profile, and the role of attitude toward work and school." Cyberpsychology, Behavior, and Social Networking 17(3): 166-171. | wrong intervention | |
| 663 | De Dios Perez, B., et al. (2024). "A qualitative study exploring how vocational rehabilitation for people with multiple sclerosis can be integrated within existing healthcare services in the United Kingdom." BMC Health Serv Res 24(1): 995. | wrong intervention | |
| 664 | De Dios Pérez, B., et al. (2024). "How does mentoring occupational therapists improve intervention fidelity in a randomised controlled trial? A realist evaluation." BMC Med Res Methodol 24(1): 142. | wrong intervention | |
| 665 | De Dios Pérez, B., et al. (2022). "Experiences of people with multiple sclerosis at work: Towards the understanding of the needs for a job retention vocational rehabilitation intervention." Work 72(1): 303-313. | wrong intervention | |
| 666 | de Gortari, A. B. O. and M. D. Griffiths (2016). "Prevalence and Characteristics of Game Transfer Phenomena: A Descriptive Survey Study." INTERNATIONAL JOURNAL OF HUMAN-COMPUTER INTERACTION 32(6): 470-480. | wrong intervention | |
| 667 | de Herder, W. W. and J. Hofland (2000). Glucagon & Glucagonoma Syndrome. Endotext. K. R. Feingold, B. Anawalt, M. R. Blackman et al. South Dartmouth (MA), MDText.com, Inc. | wrong intervention | |
| 668 | de Jong, A. E. E., et al. (2015). "The visual analogue thermometer and the graphic numeric rating scale: A comparison of self-report instruments for pain measurement in adults with burns." BURNS 41(2): 333-340. | wrong intervention | |
| 669 | de Kloet, A. J., et al. (2012). "Gaming supports youth with acquired brain injury? A pilot study." BRAIN INJURY 26(7-8): 1021-1029. | wrong population | |
| 670 | de la O-Gómez, A. T., et al. (2023). "Effectiveness of Balance Rehabilitation Unit (BRU) Posturography Versus Conventional Rehabilitation in Patients With Unilateral Peripheral Vestibular Dysfunction." CUREUS JOURNAL OF MEDICAL SCIENCE 15(9). | wrong intervention | |
| 671 | De Luca, R., et al. (2021). "Innovative use of virtual reality in autism spectrum disorder: A case-study." APPLIED NEUROPSYCHOLOGY-CHILD 10(1): 90-100. | wrong design | |
| 672 | De Luca, R., et al. (2019). "Use of virtual reality in improving poststroke neglect: Promising neuropsychological and neurophysiological findings from a case study." APPLIED NEUROPSYCHOLOGY-ADULT 26(1): 96-100. | wrong design | |
| 673 | De Luca, R., et al. (2019). "Improving neuropsychiatric symptoms following stroke using virtual reality A case report." MEDICINE 98(19). | wrong design | |
| 674 | De Luca, R., et al. (2022). "Advances in neuroRehabilitation of TREM2-related dementia A case report on a novel multimodal approach using virtual reality." MEDICINE 101(21). | wrong design | |
| 675 | De Luca, R., et al. (2021). "Improvement of brain functional connectivity in autism spectrum disorder: an exploratory study on the potential use of virtual reality." JOURNAL OF NEURAL TRANSMISSION 128(3): 371-380. | wrong intervention | |
| 676 | De Luca, R., et al. (2018). "Improving post-stroke cognitive and behavioral abnormalities by using virtual reality: A case report on a novel use of nirvana." APPLIED NEUROPSYCHOLOGY-ADULT 25(6): 581-585. | wrong design | |
| 677 | De Lucia, A., et al. (2024). "eHealth-integrated psychosocial and physical interventions for chronic pain in older adults: Scoping review." Journal of Medical Internet Research 26: 20. | wrong intervention | |
| 678 | de Oliveira, G. L., et al. (2017). "Characterization of seawater and weather aged polyurethane elastomer for bend stiffeners." POLYMER TESTING 59: 290-295. | wrong intervention | |
| 679 | de Pablo, G. S., et al. (2024). "Use of virtual reality in bipolar disorder: a systematic review." PSYCHOLOGICAL MEDICINE. | wrong population | |
| 680 | De Santis, K. K., et al. (2023). "Digital Technologies for Health Promotion and Disease Prevention in Older People: Scoping Review." JOURNAL OF MEDICAL INTERNET RESEARCH 25. | wrong intervention | |
| 681 | De Silva, A. P., et al. (2015). "A randomized control study of audio versus visual distraction to reduce patient discomfort during colonoscopy." Gastrointestinal Endoscopy 81(5): AB207-AB208. | wrong intervention | |
| 682 | de Sousa, J. C. S., et al. (2018). "Aerobic Stimulus Induced by Virtual Reality Games in Stroke Survivors." ARCHIVES OF PHYSICAL MEDICINE AND REHABILITATION 99(5): 927-933. | wrong outcomes | |
| 683 | De Vos, E. S., et al. (2024). "Morphologic development of the first-trimester utero-placental vasculature is positively associated with embryonic and fetal growth: the Rotterdam Periconception Cohort." HUMAN REPRODUCTION 39(5). | wrong intervention | |
| 684 | de Vries, A. W., et al. (2018). "Understanding motivations and player experiences of older adults in virtual reality training." Games for Health 7(6): 369-376. | wrong design | |
| 685 | Deadmond, A., et al. (2000). Ovarian Reserve Testing. Endotext. K. R. Feingold, B. Anawalt, M. R. Blackman et al. South Dartmouth (MA), MDText.com, Inc. | wrong intervention | |
| 686 | Dean, S., et al. (2020). "KARST LANDFORMS AND PREHISTORIC SETTLEMENT PATTERNS: A CASE STUDY FROM KORCULA ISLAND (CROATIA)." GEOGRAFIA FISICA E DINAMICA QUATERNARIA 43(1): 31-43. | wrong intervention | |
| 687 | Debarnot, U., et al. (2015). "Intermittent theta burst stimulation over left BA10 enhances virtual reality-based prospective memory in healthy aged subjects." Neurobiology of Aging 36(8): 2360-2369. | wrong intervention | |
| 688 | Deblock-Bellamy, A., et al. (2021). "Virtual reality-based assessment of cognitive-locomotor interference in healthy young adults." J Neuroeng Rehabil 18(1): 53. | wrong population | |
| 689 | DeCouto, B. S., et al. (2021). "Nationality and sociocultural factors influence athlete development and sport outcomes: Perspectives from United States and Austrian youth alpine ski racing." JOURNAL OF SPORTS SCIENCES 39(10): 1153-1163. | wrong population | |
| 690 | Dehghan, B., et al. (2021). "The Effect of Virtual Reality on Emotional Response and Symptoms Provocation in Patients With OCD: A Systematic Review and Meta-Analysis." Front Psychiatry 12: 733584. | wrong design | |
| 691 | Dehghan, B., et al. (2022). "The effect of virtual reality on emotional response and symptoms provocation in patients with OCD: A systematic review and meta-analysis." Frontiers in Psychiatry 12: 13. | wrong design | |
| 692 | Dekabryov, I. K. and A. V. Cheremnykh (2023). "THE ROLE OF FAULTS IN THE FORMATION OF THE OKHOTNICHYA CAVE (BAIKAL REGION)." GEODYNAMICS & TECTONOPHYSICS 14(3). | wrong design | |
| 693 | del Castillo, B. T., et al. (2019). "Reducing the pain in invasive procedures during paediatric hospital admissions: Fiction, reality or virtual reality?" ANALES DE PEDIATRIA 91(2): 80-87. | wrong population | |
| 694 | Del Piccolo, L., et al. (2012). "How emotions emerge and are dealt with in first diagnostic consultations in psychiatry." PATIENT EDUCATION AND COUNSELING 88(1): 29-35. | wrong intervention | |
| 695 | Del Piccolo, L., et al. (2015). "Patient expression of emotions and neurologist responses in first multiple sclerosis consultations." PLoS ONE 10(6): 12. | wrong intervention | |
| 696 | Del Piccolo, L., et al. (2015). "Patient Expression of Emotions and Neurologist Responses in First Multiple Sclerosis Consultations." PLOS ONE 10(6). | wrong intervention | |
| 697 | Del Pozzo, J., et al. (2024). "Detecting and Predicting Cognitive Decline in Individuals with Traumatic Brain Injury: A Longitudinal Telephone-Based Study." J Neurotrauma 41(15-16): 1937-1947. | wrong intervention | |
| 698 | Delannoy, J. J., et al. (2024). "Investigating Human Activities in Caves Through the Study of Broken Stalagmite Structures: The Case of the Saint-Marcel Cave (France) During the Early Holocene." JOURNAL OF ARCHAEOLOGICAL METHOD AND THEORY 31(3): 1543-1569. | wrong population | |
| 699 | deLeyer-Tiarks, J. M., et al. (2023). "Advancing autism technology." PSYCHOLOGY IN THE SCHOOLS 60(2): 495-506. | wrong intervention | |
| 700 | Deligöz, A., et al. (2011). "THE EFFECT OF DESICCATION STRESS AND HYDROGEL USE ON SEEDLING PHYSIOLOGY AND GROWTH IN <i>Juniperus foetidissima</i>." FRESENIUS ENVIRONMENTAL BULLETIN 20(2A): 528-533. | wrong intervention | |
| 701 | Della Longa, L., et al. (2022). "Interpersonal Affective Touch in a Virtual World: Feeling the Social Presence of Others to Overcome Loneliness." FRONTIERS IN PSYCHOLOGY 12. | wrong population | |
| 702 | Delle Monache, S., et al. (2021). "Methylglyoxal-Dependent Glycative Stress Is Prevented by the Natural Antioxidant Oleuropein in Human Dental Pulp Stem Cells through Nrf2/Glo1 Pathway." ANTIOXIDANTS 10(5). | wrong intervention | |
| 703 | Dell'Osso, L., et al. (2024). "Video Gaming in Older People: What Are the Implications for Cognitive Functions?" BRAIN SCIENCES 14(7). | wrong intervention | |
| 704 | Demir, E., et al. (2024). "Oxidative stress responses of virtual reality use in refugee children undergoing elective surgery: A randomized controlled trial." JOURNAL OF PEDIATRIC NURSING-NURSING CARE OF CHILDREN & FAMILIES 75: 80-88. | wrong population | |
| 705 | Demir, E., et al. (2024). "Changes of oxidative stress in 3D film to be prepared for echocardiography: A randomized controlled study." MEDICINE 103(35). | wrong intervention | |
| 706 | Demori, I., et al. (2021). "Effects of winter sea bathing on psychoneuroendocrinoimmunological parameters." Explore (NY) 17(2): 122-126. | wrong population | |
| 707 | Dengel, A., et al. (2021). Indicators and Predictors of the Suspension of Disbelief: Children's Individual Presence Tendencies. 2021 IEEE CONFERENCE ON VIRTUAL REALITY AND 3D USER INTERFACES ABSTRACTS AND WORKSHOPS (VRW 2021): 494-495. | wrong population | |
| 708 | Dennehy, R., et al. (2020). "Young people's conceptualizations of the nature of cyberbullying: A systematic review and synthesis of qualitative research." AGGRESSION AND VIOLENT BEHAVIOR 51. | wrong population | |
| 709 | Denneson, L. M., et al. (2011). "Alcohol consumption and health status in very old veterans." Journal of Geriatric Psychiatry and Neurology 24(1): 39-43. | wrong intervention | |
| 710 | Dennis, M. C., et al. (2022). "Taking the plunge: When is best for hot water immersion to complement exercise in heat and hypoxia." J Sports Sci 40(18): 2055-2061. | wrong population | |
| 711 | Derkarabetian, S., et al. (2010). "Repeated and Time-Correlated Morphological Convergence in Cave-Dwelling Harvestmen (Opiliones, Laniatores) from Montane Western North America." PLOS ONE 5(5). | wrong population | |
| 712 | Dermody, G., et al. (2020). "The role of virtual reality in improving health outcomes for community-dwelling older adults: Systematic review." Journal of Medical Internet Research 22(6). | wrong design | |
| 713 | Derundere, U., et al. (2024). "Health Behavior Assessment and Rehabilitation of Vestibular Migraine Patients: Interventions through Magnetic Resonance Imaging (MRI)." AMERICAN JOURNAL OF HEALTH BEHAVIOR 48(1): 19-29. | wrong intervention | |
| 714 | Desai, B., et al. (2024). "Virtual reality use in pediatric patients for orthopedic clinical procedures: A randomized prospective trial of efficacy." JOURNAL OF CHILDRENS ORTHOPAEDICS 18(4): 414-420. | wrong population | |
| 715 | Dev, S. I. and L. T. Eyler (2017). "Bipolar patients with vascular risk display a steeper age-related negative slope in inhibitory performance but not processing speed: A preliminary study." The American Journal of Geriatric Psychiatry 25(3): 272-276. | wrong population | |
| 716 | Devassy, S. M., et al. (2023). "Development of immersive learning framework (ILF) in achieving the goals of higher education: measuring the impact using a pre-post design." SCIENTIFIC REPORTS 13(1). | wrong population | |
| 717 | Devi, K. N., et al. (2022). Sensor based posture detection system. MATERIALS TODAY-PROCEEDINGS. 55: 359-364. | wrong population | |
| 718 | Dewar, G. and B. A. Stewart (2012). "Preliminary results of excavations at Spitzkloof Rockshelter, Richtersveld, South Africa." QUATERNARY INTERNATIONAL 270: 30-39. | wrong population | |
| 719 | Dhahir, M. K. and S. Marx (2023). "Development of expansive concrete for chemical prestressing applications." CASE STUDIES IN CONSTRUCTION MATERIALS 19. | wrong population | |
| 720 | Dhamodharan, T., et al. (2020). Cognitive Rehabilitation for Autism Children Mental Status Observation Using Virtual Reality Based Interactive Environment. INTELLIGENT HUMAN SYSTEMS INTEGRATION 2020. 1131: 1213-1218. | wrong population | |
| 721 | Di Corrado, D., et al. (2020). "Mental Imagery Skills in Competitive Young Athletes and Non-athletes." FRONTIERS IN PSYCHOLOGY 11. | wrong population | |
| 722 | Di Credico, G., et al. (2019). "Joint effects of intensity and duration of cigarette smoking on the risk of head and neck cancer: A bivariate spline model approach." Oral Oncol 94: 47-57. | wrong population | |
| 723 | Di Fiore, A., et al. (2024). "Comparison of mechanical and surface properties of two 3D printed composite resins for definitive restoration." J Prosthet Dent 132(4): 839.e831-839.e837. | wrong population | |
| 724 | di Santolo, A. S., et al. (2013). The Fontanelle Cemetery: Between legend and reality. GEOTECHNICAL ENGINEERING FOR THE PRESERVATION OF MONUMENTS AND HISTORIC SITES: 641-648. | wrong population | |
| 725 | Dias, J. C. D. and L. D. P. Bacellar (2021). "A hydrogeological conceptual model for the groundwater dynamics in the ferricretes of Capao Xavier, Iron Quadrangle, Southeastern Brazil." CATENA 207. | wrong population | |
| 726 | Diaz, G. M., et al. (2024). "Risk Factors and Contemporary Management Options for Pain and Discomfort Experienced During a Prostate Biopsy." CURRENT UROLOGY REPORTS 25(10): 243-252. | wrong population | |
| 727 | Diaz-Orueta, U., et al. (2012). ""Isla Calma'', a Novel Virtual Reality Environment for Pain and Anxiety Distraction: Report on Usability, Acceptability, and Subjective Experience." GAMES FOR HEALTH JOURNAL 1(5): 353-361. | wrong design | |
| 728 | Diedrich, C. G. (2011). "The largest European lion <i>Panthera leo spelaea</i> (Goldfuss 1810) population from the Zoolithen Cave, Germany: specialised cave bear predators of Europe." HISTORICAL BIOLOGY 23(2-3): 271-311. | wrong population | |
| 729 | Diedrich, C. G. (2012). "Cave bear killers and scavengers from the last ice age of central Europe: Feeding specializations in response to the absence of mammoth steppe fauna from mountainous regions." QUATERNARY INTERNATIONAL 255: 59-78. | wrong population | |
| 730 | Dijkstra-Soudarissanane, S., et al. (2022). Virtual Visits: Life-size Immersive Communication. PROCEEDINGS OF THE 13TH ACM MULTIMEDIA SYSTEMS CONFERENCE, MMSYS 2022: 310-314. | wrong population | |
| 731 | Dijkstra-Soudarissanane, S., et al. (2021). Towards XR Communication for Visiting Elderly at Nursing Homes. PROCEEDINGS OF THE 2021 ACM INTERNATIONAL CONFERENCE ON INTERACTIVE MEDIA EXPERIENCES, IMX 2021: 319-321. | wrong population | |
| 732 | Dilanchian, A. T., et al. (2021). "A Pilot Study Exploring Age Differences in Presence, Workload, and Cybersickness in the Experience of Immersive Virtual Reality Environments." FRONTIERS IN VIRTUAL REALITY 2. | wrong outcomes | |
| 733 | Dilek, S., et al. (2024). "Technology versus nostalgia; A randomized controlled trial of the effect of virtual reality and kaleidescop on pediatric pain, fear and anxiety management during immunization<SUP>1</SUP>." JOURNAL OF PEDIATRIC NURSING-NURSING CARE OF CHILDREN & FAMILIES 78: e383-e388. | wrong population | |
| 734 | Dimitropoulos, G., et al. (2024). "Implementation of an Electronic Mental Health Platform for Youth and Young Adults in a School Context Across Alberta, Canada: Thematic Analysis of the Perspectives of Stakeholders." JMIR Ment Health 11: e49099. | wrong population | |
| 735 | Dimitrova, M., et al. (2023). "WATER SORPTION AND WATER SOLUBILITY OF 3D PRINTED AND CONVENTIONAL PMMA DEN- TURE BASE POLYMERS." JOURNAL OF IMAB 29(2): 4939-4942. | wrong outcomes | |
| 736 | Dindar, M. and Y. Akbulut (2015). "Role of self-efficacy and social appearance anxiety on gaming motivations of MMOFPS players." COMPUTERS & EDUCATION 81: 26-34. | wrong intervention | |
| 737 | Dinet, J., et al. (2023). Breaking Social Isolation for Older People Living Alone with Technology. PROCEEDINGS OF THE EUROPEAN CONFERENCE ON COGNITIVE ERGONOMICS, ECCE 2023: Responsible Technology Community, Culture, and Sustainability. | wrong intervention | |
| 738 | Dinet, J., et al. (2024). "Breaking social isolation for older people living alone with technology." Behaviour & Information Technology 43(9): 1740. | wrong intervention | |
| 739 | Ding, R. and N. Bowler (2015). "Permittivity and Electrical Breakdown Response of Nylon 6 to Chemical Exposure." IEEE TRANSACTIONS ON DIELECTRICS AND ELECTRICAL INSULATION 22(2): 1151-1160. | wrong intervention | |
| 740 | Ding, X. F., et al. (2015). "SPECIFIC EFFECTS OF ANGER RUMINATION ON PARTICULAR EXECUTIVE FUNCTIONS." PSYCHOLOGICAL REPORTS 117(3): 825-841. | wrong intervention | |
| 741 | Diniz, V., et al. (2019). "Impact of Acid Concentration and Firing on the Long-term Bond Strength of a Zirconia-Lithium Silicate Ceramic Following Adhesive Cementation." J Adhes Dent 21(4): 355-363. | wrong intervention | |
| 742 | Dino, M. J. S., et al. (2024). "Mixed reality technology for older adults: Evaluating the impact of a novel virtual humanoid coach in a community-based physical exercise program in the Philippines." Health Informatics J 30(3): 14604582241267793. | wrong outcomes | |
| 743 | Dinu, D. G. and A. Stoian-Karadeli (2019). COMPETITIVE INTELLIGENCE IN THE DIGITAL AGE: NEUROTECHNOLOGIES FOR HUMAN COGNITIVE AUGMENTATION. BUSINESS MANAGEMENT THEORIES AND PRACTICES IN A DYNAMIC COMPETITIVE ENVIRONMENT: 1619-1621. | wrong population | |
| 744 | Dionne-Odom, J. N., et al. (2018). "Participation and interest in support services among family caregivers of older adults with cancer." Psychooncology 27(3): 969-976. | wrong population | |
| 745 | Dirks, P. and L. R. Berger (2013). "Hominin-bearing caves and landscape dynamics in the Cradle of Humankind, South Africa." JOURNAL OF AFRICAN EARTH SCIENCES 78: 109-131. | wrong population | |
| 746 | Ditchburn, J. L., et al. (2020). "The effects of exergaming on pain, postural control, technology acceptance and flow experience in older people with chronic musculoskeletal pain: a randomised controlled trial." BMC SPORTS SCIENCE MEDICINE AND REHABILITATION 12(1). | wrong intervention | |
| 747 | Djaiani, G., et al. (2016). "Dexmedetomidine versus Propofol Sedation Reduces Delirium after Cardiac Surgery: A Randomized Controlled Trial." Anesthesiology 124(2): 362-368. | wrong intervention | |
| 748 | Dockx, K., et al. (2017). "Fall-prone older people's attitudes towards the use of virtual reality technology for fall prevention." Gerontology 63(6): 590-598. | wrong outcomes | |
| 749 | Dogra, N., et al. (2024). "Vision-related quality of life after unilateral occipital stroke." BRAIN AND BEHAVIOR 14(7). | wrong intervention | |
| 750 | Dogra, P., et al. (2023). "Influence of augmented reality on shopping behavior." MANAGEMENT DECISION. | wrong population | |
| 751 | Dolins, F. L., et al. (2017). "Technology advancing the study of animal cognition: using virtual reality to present virtually simulated environments to investigate nonhuman primate spatial cognition." CURRENT ZOOLOGY 63(1): 97-108. | wrong population | |
| 752 | Doll, K. M., et al. (2017). "Pre-diagnosis health-related quality of life, surgery, and survival in women with advanced epithelial ovarian cancer: A SEER-MHOS study." GYNECOLOGIC ONCOLOGY 144(2): 348-353. | wrong population | |
| 753 | Dolu, U. and A. F. Camliguney (2022). "The Effect of Virtual Reality on Isometric Muscle Strength." PROGRESS IN NUTRITION 24(1). | wrong population | |
| 754 | Domb, B. G., et al. (2021). "Arthroscopic Circumferential Acetabular Labral Reconstruction for Irreparable Labra in the Revision Setting: Patient-Reported Outcome Scores and Rate of Achieving the Minimal Clinically Important Difference at a Minimum 2-Year Follow-up." AMERICAN JOURNAL OF SPORTS MEDICINE 49(7): 1750-1758. | wrong population | |
| 755 | Domenicucci, R., et al. (2022). "Efficacy of ICT-based interventions in improving psychological outcomes among older adults with MCI and dementia: A systematic review and meta-analysis." AGEING RESEARCH REVIEWS 82. | wrong intervention | |
| 756 | Domingues, C. A., et al. (2015). Prolonged Physical Effort Affects Cognitive Processes During Special Forces Training. FOUNDATIONS OF AUGMENTED COGNITION, AC 2015. 9183: 570-582. | wrong population | |
| 757 | Dommes, A., et al. (2014). "Crossing a two-way street: Comparison of young and old pedestrians." Journal of Safety Research 50: 27-34. | wrong population | |
| 758 | Dong, S., et al. (2014). "Electrospun nanofibrous scaffolds of poly (L-lactic acid)-dicalcium silicate composite via ultrasonic-aging technique for bone regeneration." Mater Sci Eng C Mater Biol Appl 35: 426-433. | wrong intervention | |
| 759 | Dong, S. J., et al. (2014). "Electrospun nanofibrous scaffolds of poly (L-lactic acid)-dicalcium silicate composite via ultrasonic-aging technique for bone regeneration." MATERIALS SCIENCE & ENGINEERING C-MATERIALS FOR BIOLOGICAL APPLICATIONS 35: 426-433. | wrong population | |
| 760 | Dong, W. H., et al. (2023). "Parental phubbing and chinese adolescents' SNSs addiction: loneliness as a mediator and self-esteem as a moderator." CURRENT PSYCHOLOGY 42(19): 16560-16572. | wrong population | |
| 761 | Donisi, V., et al. (2024). "Combining acceptance and commitment therapy with adventure therapy to face vulnerability: Examples and insights from a sailing experience." JOURNAL OF CONTEXTUAL BEHAVIORAL SCIENCE 32. | wrong population | |
| 762 | Donker, T., et al. (2019). "Effectiveness of Self-guided App-Based Virtual Reality Cognitive Behavior Therapy for Acrophobia: A Randomized Clinical Trial." JAMA PSYCHIATRY 76(7): 682-690. | wrong population | |
| 763 | Donker, T., et al. (2018). "0Phobia-towards a virtual cure for acrophobia: study protocol for a randomized controlled trial." TRIALS 19. | wrong population | |
| 764 | Donnari, S., et al. (2019). "NEW TECHNOLOGIES FOR ART THERAPY INTERVENTIONS TAILORED TO SEVERE DISABILITIES." PSYCHIATRIA DANUBINA 31: S462-S466. | wrong intervention | |
| 765 | Donovan, G. K. and D. P. Wilson (2000). Understanding Ethical Dilemmas in Pediatric Lipidology- Genetic Testing in Youth. Endotext. K. R. Feingold, B. Anawalt, M. R. Blackman et al. South Dartmouth (MA), MDText.com, Inc. | wrong population | |
| 766 | Döpfner, M., et al. (2019). "Affective dysregulation in childhood - optimizing prevention and treatment: protocol of three randomized controlled trials in the ADOPT study." BMC Psychiatry 19(1): 264. | wrong population | |
| 767 | Döring, N., et al. (2022). "Can Communication Technologies Reduce Loneliness and Social Isolation in Older People? A Scoping Review of Reviews." Int J Environ Res Public Health 19(18). | wrong intervention | |
| 768 | Dragoo, J. L., et al. (2020). "Outcomes of Arthroscopic All-Inside Repair Versus Observation in Older Patients With Meniscus Root Tears." AMERICAN JOURNAL OF SPORTS MEDICINE 48(5): 1127-1133. | wrong intervention | |
| 769 | Draper, B., et al. (2010). "Workforce and advanced training survey of the RANZCP Faculty of Psychiatry of Old Age: issues and challenges for the field." AUSTRALASIAN PSYCHIATRY 18(2): 142-145. | wrong intervention | |
| 770 | Drazich, B. F., et al. (2023). "Motivating older adults through immersive virtual exercise (MOTIVE): A randomized pilot study." Geriatr Nurs 54: 229-236. | wrong outcomes | |
| 771 | Drazich, B. F., et al. (2023). "Motivating older adults through immersive virtual exercise (MOTIVE): A randomized pilot study." GERIATRIC NURSING 54: 229-236. | wrong outcomes | |
| 772 | Drazich, B. F., et al. (2020). "Exergames and depressive symptoms in older adults: A systematic review." Games for Health 9(5): 339-345. | wrong intervention | |
| 773 | Dreesmann, N. J., et al. (2023). "Virtual Reality Meditation for Fatigue in Persons With Rheumatoid Arthritis: Mixed Methods Pilot Study." JMIR Formative Research 7(1). | wrong population | |
| 774 | Dreesmann, N. J., et al. (2023). "Virtual Reality Meditation for Fatigue in Persons With Rheumatoid Arthritis: Mixed Methods Pilot Study." JMIR Form Res 7: e46209. | wrong population | |
| 775 | Dreesmann, N. J., et al. (2023). "Virtual Reality Meditation for Fatigue in Persons With Rheumatoid Arthritis: Mixed Methods Pilot Study." JMIR FORMATIVE RESEARCH 7. | wrong population | |
| 776 | Droc, G., et al. (2023). "Postoperative Cognitive Impairment and Pain Perception after Abdominal Surgery-Could Immersive Virtual Reality Bring More? A Clinical Approach." MEDICINA-LITHUANIA 59(11). | wrong population | |
| 777 | Drozdova, A., et al. (2024). "Comparing Conventional Physician-Led Education with VR Education for Pacemaker Implantation: A Randomized Study." HEALTHCARE 12(10). | wrong population | |
| 778 | Drozdova, A., et al. (2024). "Comparing Conventional Physician-Led Education with VR Education for Pacemaker Implantation: A Randomized Study." Healthcare (Basel) 12(10). | wrong intervention | |
| 779 | Drummond, P. D. and K. J. R. Clark (2023). "The sensory and affective components of pain differentially shape pupillary dilatation during cold pressor tests." Auton Neurosci 246: 103084. | wrong population | |
| 780 | Duan, Y., et al. (2016). "Effect of desiccation and resubmersion on the oxidative stress response of the kuruma shrimp Marsupenaeus japonicus." Fish Shellfish Immunol 49: 91-99. | wrong population | |
| 781 | Dumitrescu, L., et al. (2020). "Genetic variants and functional pathways associated with resilience to Alzheimer's disease." Brain 143(8): 2561-2575. | wrong intervention | |
| 782 | Dumoulin, S., et al. (2019). "A Randomized Controlled Trial of the Use of Virtual Reality for Needle-Related Procedures in Children and Adolescents in the Emergency Department." GAMES FOR HEALTH JOURNAL 8(4): 285-293. | wrong population | |
| 783 | Dunn, A., et al. (2019). "A Novel Clinician-Orchestrated Virtual Reality Platform for Distraction During Pediatric Intravenous Procedures in Children With Hemophilia: Randomized Controlled Trial." JMIR SERIOUS GAMES 7(1). | wrong population | |
| 784 | Dunsky, A. (2019). "The Effect of Balance and Coordination Exercises on Quality of Life in Older Adults: A Mini-Review." FRONTIERS IN AGING NEUROSCIENCE 11. | wrong outcomes | |
| 785 | Dupoué, A., et al. (2023). "Intertidal limits shape covariation between metabolic plasticity, oxidative stress and telomere dynamics in Pacific oyster<i> (Crassostrea</i><i> gigas)</i>." MARINE ENVIRONMENTAL RESEARCH 191. | wrong intervention | |
| 786 | Durán, L., et al. (2021). Digital audiovisual contents for literacy in depression: a pilot study with university students. INTERNATIONAL CONFERENCE ON ENTERPRISE INFORMATION SYSTEMS / INTERNATIONAL CONFERENCE ON PROJECT MANAGEMENT / INTERNATIONAL CONFERENCE ON HEALTH AND SOCIAL CARE INFORMATION SYSTEMS AND TECHNOLOGIES 2020 (CENTERIS/PROJMAN/HCIST 2020). 181: 239-246. | wrong population | |
| 787 | Dutta, R., et al. (2022). "Evaluating system usability of mobile augmented reality application for teaching Karnaugh-Maps." SMART LEARNING ENVIRONMENTS 9(1). | wrong population | |
| 788 | Dwivedi, S., et al. (2018). VR-Based "9-Square Matrix" Aerobic Exercise for Prevention of Physical Decline in Older Adults. 2018 1ST INTERNATIONAL ECTI NORTHERN SECTION CONFERENCE ON ELECTRICAL, ELECTRONICS, COMPUTER AND TELECOMMUNICATIONS ENGINEERING (ECTI-NCON: 106-110. | wrong outcomes | |
| 789 | Easton, K., et al. (2019). "A virtual agent to support individuals living with physical and mental comorbidities: Co-design and acceptability testing." Journal of Medical Internet Research 21(5): 15. | wrong population | |
| 790 | Ebrahimi, E., et al. (2022). Designing Virtual Environments for Smoking Cessation: A Preliminary Investigation. VIRTUAL, AUGMENTED AND MIXED REALITY: DESIGN AND DEVELOPMENT, PT I. 13317: 410-422. | wrong population | |
| 791 | Echt, M., et al. (2023). "Comparison of minimally invasive decompression alone versus minimally invasive short-segment fusion in the setting of adult degenerative lumbar scoliosis: a propensity score-matched analysis." JOURNAL OF NEUROSURGERY-SPINE 39(3): 394-403. | wrong population | |
| 792 | Ecker, A., et al. (2024). "Multifactorial stress reactivity to virtual TSST-C in healthy children and adolescents-It works, but not as well as a real TSST-C." PSYCHONEUROENDOCRINOLOGY 160. | wrong population | |
| 793 | Edjokola, J. M., et al. (2024). "Chemical Oxidation-Induced Degradation in Gas Diffusion Layers for PEFC: Mechanisms and Performance Implications." JOURNAL OF THE ELECTROCHEMICAL SOCIETY 171(9). | wrong population | |
| 794 | Edmonds, C., et al. (2019). "Immersion pulmonary edema: an analysis of 31 cases from Oceania." Undersea Hyperb Med 46(5): 603-610. | wrong population | |
| 795 | Ege, F., et al. (2024). "Effects of the Cold Pressor Test on Popliteal Vein Diameter, Flow Velocity, and Blood Flow in the Lower Limb in 60 Healthy Individuals." MEDICAL SCIENCE MONITOR 30. | wrong population | |
| 796 | Eggenberger, P., et al. (2015). "Does multicomponent physical exercise with simultaneous cognitive training boost cognitive performance in older adults? A 6-month randomized controlled trial with a 1-year follow-up." CLINICAL INTERVENTIONS IN AGING 10: 1335-1349. | wrong intervention | |
| 797 | Eggenberger, P., et al. (2015). "Multicomponent physical exercise with simultaneous cognitive training to enhance dual-task walking of older adults: a secondary analysis of a 6-month randomized controlled trial with 1-year follow-up." CLINICAL INTERVENTIONS IN AGING 10: 1711-1732. | wrong outcomes | |
| 798 | Ehgoetz Martens, K. A., et al. (2016). "Evaluating the Link Between Dopaminergic Treatment, Gait Impairment, and Anxiety in Parkinson's Disease." Mov Disord Clin Pract 3(4): 389-394. | wrong intervention | |
| 799 | Ehrlich, Y., et al. (2022). "Olive pits as a high-resolution proxy archive of climate: Δ13C in modern and archaeological olive pits reflecting environmental conditions." QUATERNARY SCIENCE REVIEWS 294. | wrong population | |
| 800 | Eichenbaum, J. W. (2012). "Geriatric Vision Loss Due to Cataracts, Macular Degeneration, and Glaucoma." MOUNT SINAI JOURNAL OF MEDICINE 79(2): 276-294. | wrong population | |
| 801 | Eijlers, R., et al. (2019). "Virtual reality exposure before elective day care surgery to reduce anxiety and pain in children A randomised controlled trial." EUROPEAN JOURNAL OF ANAESTHESIOLOGY 36(10): 728-737. | wrong population | |
| 802 | Eijlers, R., et al. (2021). "Predicting Intense Levels of Child Anxiety During Anesthesia Induction at Hospital Arrival." JOURNAL OF CLINICAL PSYCHOLOGY IN MEDICAL SETTINGS 28(2): 313-322. | wrong population | |
| 803 | Eijlers, R., et al. (2019). "Systematic Review and Meta-analysis of Virtual Reality in Pediatrics: Effects on Pain and Anxiety." ANESTHESIA AND ANALGESIA 129(5): 1344-1353. | wrong population | |
| 804 | Eisen, A. M., et al. (2024). "Susceptibility to stress and nature exposure: Unveiling differential susceptibility to physical environments; a randomized controlled trial." PLOS ONE 19(4). | wrong population | |
| 805 | El Garchani, F. E. and M. R. Kabiri (2023). "Intergranular corrosion and mechanical property evolution in AA2024 alloy through heat treatment." INTERNATIONAL JOURNAL OF ADVANCED MANUFACTURING TECHNOLOGY 128(7-8): 3273-3282. | wrong population | |
| 806 | El Garchani, F. E., et al. (2023). "Effects of heat treatment on the corrosion behavior and mechanical properties of aluminum alloy 2024." JOURNAL OF MATERIALS RESEARCH AND TECHNOLOGY-JMR&T 25: 1355-1363. | wrong population | |
| 807 | Elena, P., et al. (2021). "Differences Between Exergaming Rehabilitation and Conventional Physiotherapy on Quality of Life in Parkinson's Disease: A Systematic Review and Meta-Analysis." FRONTIERS IN NEUROLOGY 12. | wrong intervention | |
| 808 | Eliason, M. J., et al. (2016). "The “something else” of sexual orientation: Measuring sexual identities of older lesbian and bisexual women using National Health Interview Survey questions." Women's Health Issues 26(Suppl 1): S71-S80. | wrong population | |
| 809 | Elies, C. and É. Serra (2022). "Réalité virtuelle et santé des personnes âgées institutionnalisées. Vers un EHPAD 2.0 ?" Pratiques Psychologiques 28(4): 191-207. | wrong design | |
| 810 | Elies, C. and É. Serra (2022). "Virtual reality and health among elderly in retirement home. Towards French EHPAD 2.0?" Pratiques Psychologiques 28(4): 191-207. | wrong design | |
| 811 | El-Kafy, E. M. A., et al. (2021). "The effect of virtual reality-based therapy on improving upper limb functions in individuals with stroke: A randomized control trial." Frontiers in Aging Neuroscience 13: 8. | wrong outcomes | |
| 812 | El-Khoury, M., et al. (2024). "A new protocol to evaluate the behaviour and durability of marine structures." OCEAN ENGINEERING 302. | wrong population | |
| 813 | Ellerton, K., et al. (2020). "The VRIMM study: Virtual Reality for IMMunisation pain in young children-protocol for a randomised controlled trial." BMJ OPEN 10(8). | wrong population | |
| 814 | Ellerton, K., et al. (2023). "Virtual Reality for IMMunisation (VRIMM) pain in young children <i>Results of a randomised controlled trial in general practice</i>." AUSTRALIAN JOURNAL OF GENERAL PRACTICE 52(10): 704-710. | wrong population | |
| 815 | Elliott, V., et al. (2015). "Virtual Reality Rehabilitation as a Treatment Approach for Older Women With Mixed Urinary Incontinence: A Feasibility Study." NEUROUROLOGY AND URODYNAMICS 34(3): 236-243. | wrong outcomes | |
| 816 | Ellis, B. H., et al. (2021). "Risk and Protective Factors Associated With Support of Violent Radicalization: Variations by Geographic Location." INTERNATIONAL JOURNAL OF PUBLIC HEALTH 66. | wrong population | |
| 817 | Elshazly, M. B., et al. (2021). "Exercise Ventricular Rates, Cardiopulmonary Exercise Performance, and Mortality in Patients With Heart Failure With Atrial Fibrillation." CIRCULATION-HEART FAILURE 14(2). | wrong population | |
| 818 | Ende, V. J., et al. (2021). "Survival of COVID-19 Patients With Respiratory Failure is Related to Temporal Changes in Gas Exchange and Mechanical Ventilation." J Intensive Care Med 36(10): 1209-1216. | wrong population | |
| 819 | Ende, V. J., et al. (2021). "Survival of COVID-19 Patients With Respiratory Failure is Related to Temporal Changes in Gas Exchange and Mechanical Ventilation." JOURNAL OF INTENSIVE CARE MEDICINE 36(10): 1209-1216. | wrong intervention | |
| 820 | Eraslan Boz, H., et al. (2020). "A new tool to assess amnestic mild cognitive impairment in Turkish older adults: Virtual Supermarket (VSM)." Aging, Neuropsychology, and Cognition 27(5): 639-653. | wrong population | |
| 821 | Erdogan, B. and A. A. Ozdemir (2021). "The Effect of Three Different Methods on Venipuncture Pain and Anxiety in Children: Distraction Cards, Virtual Reality, and Buzzy® (Randomized Controlled Trial)." JOURNAL OF PEDIATRIC NURSING-NURSING CARE OF CHILDREN & FAMILIES 58. | wrong population | |
| 822 | Erdos, S. and K. Horváth (2023). "The Impact of Virtual Reality (VR) on Psychological and Physiological Variables in Children Receiving Chemotherapy: A Pilot Cross-Over Study." INTEGRATIVE CANCER THERAPIES 22. | wrong population | |
| 823 | Erkul, M., et al. (2022). "The effect of a peer-assisted video-based training programme and counselling to reduce anxiety in children newly diagnosed with cancer: Non-randomised controlled trial." EUROPEAN JOURNAL OF CANCER CARE 31(6). | wrong population | |
| 824 | Ersin, K., et al. (2023). "Appropriate Image Selection With Virtual Reality in Vestibular Rehabilitation: Cross-sectional Study." JMIR SERIOUS GAMES 11. | wrong outcomes | |
| 825 | Escamilla, J. C., et al. (2023). "Aging: working memory capacity and spatial strategies in a virtual orientation task." GEROSCIENCE 45(1): 159-175. | wrong intervention | |
| 826 | Esposito, C., et al. (2022). "Efficacy of a Virtual Reality Program in Pediatric Surgery to Reduce Anxiety and Distress Symptoms in the Preoperative Phase: A Prospective Randomized Clinical Trial." Journal of Laparoendoscopic and Advanced Surgical Techniques 32(2): 197-203. | wrong population | |
| 827 | Esposito, D., et al. (2023). "Young children can use their subjective straight-ahead to remap visuo-motor alterations." SCIENTIFIC REPORTS 13(1). | wrong population | |
| 828 | Esumi, R., et al. (2020). "Virtual reality as a non-pharmacologic analgesic for fasciotomy wound infections in acute compartment syndrome: a case report." JOURNAL OF MEDICAL CASE REPORTS 14(1). | wrong population | |
| 829 | Etchemendy, E., et al. (2011). "An e-health platform for the elderly population: The butler system." COMPUTERS & EDUCATION 56(1): 275-279. | wrong outcomes | |
| 830 | Euhus, D. M., et al. (2019). "Change in health-related quality of life in older women after diagnosis of a small breast cancer." CANCER 125(11): 1807-1814. | wrong outcomes | |
| 831 | Eun, S. J., et al. (2022). "Development and Evaluation of an Artificial Intelligence-Based Cognitive Exercise Game: A Pilot Study." JOURNAL OF ENVIRONMENTAL AND PUBLIC HEALTH 2022. | wrong population | |
| 832 | Evadgian, A., et al. (2022). "The Use of Cheminformatics to Determine Potential Drug Interactions between Popular Barbadian Botanical Medicines and Antihypertensive Drugs." West Indian Medical Journal 70: 49-50. | wrong population | |
| 833 | Evans, A. J., et al. (2023). "Physiologic Fidelity as a Domain in Assessing Mixed Reality Trauma Simulation." Mil Med 188(11-12): 3322-3329. | wrong population | |
| 834 | Evans, C. P., et al. (2020). "The Potential Benefits of Personalized 360 Video Experiences on Affect: A Proof-of-Concept Study." CYBERPSYCHOLOGY BEHAVIOR AND SOCIAL NETWORKING 23(2): 134-138. | wrong population | |
| 835 | Evered, L., et al. (2018). "Recommendations for the nomenclature of cognitive change associated with anaesthesia and surgery-2018." Br J Anaesth 121(5): 1005-1012. | wrong population | |
| 836 | Ewais, A. M. R., et al. (2018). "17-year elevated temperature study of HDPE geomembrane longevity in air, water and leachate." GEOSYNTHETICS INTERNATIONAL 25(5): 525-544. | wrong population | |
| 837 | Faber, A. W., et al. (2013). "Repeated Use of Immersive Virtual Reality Therapy to Control Pain During Wound Dressing Changes in Pediatric and Adult Burn Patients." JOURNAL OF BURN CARE & RESEARCH 34(5): 563-568. | wrong population | |
| 838 | Fabricant, P. D., et al. (2024). "Virtual Reality Distraction Is No Better Than Simple Distraction Techniques for Reducing Pain and Anxiety During Pediatric Orthopaedic Outpatient Procedures: A Randomized Controlled Trial." CLINICAL ORTHOPAEDICS AND RELATED RESEARCH 482(5): 854-863. | wrong population | |
| 839 | Fabritz, L., et al. (2024). "Blood-based cardiometabolic phenotypes in atrial fibrillation and their associated risk: EAST-AFNET 4 biomolecule study." Cardiovasc Res 120(8): 855-868. | wrong population | |
| 840 | Fadakar, F., et al. (2024). "Treeing modeling and the effect of rejuvenation fluid in aged XLPE power cables based on the finite element method." ELECTRICAL ENGINEERING. | wrong population | |
| 841 | Færevik, H. and R. E. Reinertsen (2012). "Initial Heat Stress on Subsequent Responses to Cold Water Immersion While Wearing Protective Clothing." AVIATION SPACE AND ENVIRONMENTAL MEDICINE 83(8): 746-750. | wrong population | |
| 842 | Fan, C. C., et al. (2022). "The effects of a combination of 3D virtual reality and hands-on horticultural activities on mastery, achievement motives, self-esteem, isolation and depression: a quasi-experimental study." BMC GERIATRICS 22(1). | wrong design | |
| 843 | Fan, C.-C., et al. (2022). "The effects of a combination of 3D virtual reality and hands-on horticultural activities on mastery, achievement motives, self-esteem, isolation and depression: a quasi-experimental study." BMC Geriatrics 22(1): 744-744. | wrong design | |
| 844 | Fan, X., et al. (2020). "Processing Induced Nonequilibrium Behavior of Polyvinylpyrrolidone Nanofilms Revealed by Dewetting." Langmuir 36(50): 15430-15441. | wrong population | |
| 845 | Fang, T. Y., et al. (2023). "Development of a mobile tele-education system to assist remote otolaryngology learning during COVID-19 pandemic." Comput Methods Programs Biomed Update 3: 100102. | wrong population | |
| 846 | Faqihi, F. A., et al. (2024). "Examining the Effects of Social Media on Mental Health Among Adolescents in Saudi Arabia." CUREUS JOURNAL OF MEDICAL SCIENCE 16(1). | wrong population | |
| 847 | Farran, E. K., et al. (2012). "How useful are landmarks when learning a route in a virtual environment? Evidence from typical development and Williams syndrome." Journal of Experimental Child Psychology 111(4): 571-586. | wrong population | |
| 848 | Farran, E. K., et al. (2012). "Colour as an environmental cue when learning a route in a virtual environment: Typical and atypical development." Research in Developmental Disabilities 33(3): 900-908. | wrong population | |
| 849 | Farrell, L. J., et al. (2021). "Virtual Reality One-Session Treatment of Child-Specific Phobia of Dogs: A Controlled, Multiple Baseline Case Series." BEHAVIOR THERAPY 52(2): 478-491. | wrong population | |
| 850 | Farrer, L., et al. (2013). "Technology-Based Interventions for Mental Health in Tertiary Students: Systematic Review." JOURNAL OF MEDICAL INTERNET RESEARCH 15(5). | wrong population | |
| 851 | Farrow, L. D., et al. (2022). "Evaluation of Health Care Disparities in Patients With Anterior Cruciate Ligament Injury: Does Race and Insurance Matter?" ORTHOPAEDIC JOURNAL OF SPORTS MEDICINE 10(9). | wrong population | |
| 852 | Farzan, R., et al. (2023). "Effects of non-pharmacological interventions on pain intensity of children with burns: A systematic review and meta-analysis." INTERNATIONAL WOUND JOURNAL 20(7): 2898-2913. | wrong population | |
| 853 | Fasilis, T., et al. (2018). "A pilot study and brief overview of rehabilitation via virtual environment in patients suffering from dementia." Psychiatriki 29(1): 42-51. | wrong design | |
| 854 | Fatah, N. K. A., et al. (2024). "Effect of Immersive Virtual Reality Reminiscence versus Traditional Reminiscence Therapy on Cognitive Function and Psychological Well- being among Older Adults in Assisted Living Facilities: A randomized controlled trial." GERIATRIC NURSING 55: 191-203. |  |  |
| 855 | Faw, M. H., et al. (2021). "Being there: Exploring virtual symphonic experience as a salutogenic design intervention for older adults." Frontiers in Psychology 12: 8. | wrong design | |
| 856 | Feder, A., et al. (2019). "The Biology of Human Resilience: Opportunities for Enhancing Resilience Across the Life Span." Biol Psychiatry 86(6): 443-453. | wrong population | |
| 857 | Federico, F. (2020). "Natural Environment and Social Relationship in the Development of Attentional Network." Front Psychol 11: 1345. | wrong population | |
| 858 | Fehribach, J. R., et al. (2021). "Virtual Reality Self-help Treatment for Aviophobia: Protocol for a Randomized Controlled Trial." JMIR RESEARCH PROTOCOLS 10(4). | wrong population | |
| 859 | Feitosa, S. A., et al. (2015). "Effect of cleansing methods on saliva-contaminated zirconia--an evaluation of resin bond durability." Oper Dent 40(2): 163-171. | wrong population | |
| 860 | Felemban, O. M., et al. (2021). "Effect of virtual reality distraction on pain and anxiety during infiltration anesthesia in pediatric patients: a randomized clinical trial." BMC ORAL HEALTH 21(1). | wrong population | |
| 861 | Ferdiana, A., et al. (2014). "Assessment of health-related quality of life of clients in vocational rehabilitation: association with depressive symptoms and type of services." EUROPEAN JOURNAL OF PHYSICAL AND REHABILITATION MEDICINE 50(3): 343-353. | wrong population | |
| 862 | Ferguson, C. and H. van Oostendorp (2020). "Lost in Learning: Hypertext Navigational Efficiency Measures Are Valid for Predicting Learning in Virtual Reality Educational Games." FRONTIERS IN PSYCHOLOGY 11. | wrong population | |
| 863 | Fernandez, A. B., et al. (2010). "Tendency to Angry Rumination Predicts Stress-Provoked Endothelin-1 Increase in Patients With Coronary Artery Disease." PSYCHOSOMATIC MEDICINE 72(4): 348-353. | wrong population | |
| 864 | Fernandez Esmerats, J., et al. (2019). "Disturbed Flow Increases UBE2C (Ubiquitin E2 Ligase C) via Loss of miR-483-3p, Inducing Aortic Valve Calcification by the pVHL (von Hippel-Lindau Protein) and HIF-1α (Hypoxia-Inducible Factor-1α) Pathway in Endothelial Cells." Arterioscler Thromb Vasc Biol 39(3): 467-481. | wrong population | |
| 865 | Fernández, I. (2022). "The Need for Experimental and Numerical Analyses of Thermal Ageing in Power Transformers." ENERGIES 15(17). | wrong population | |
| 866 | Fernández, I., et al. (2016). "Thermal degradation assessment of Kraft paper in power transformers insulated with natural esters." APPLIED THERMAL ENGINEERING 104: 129-138. | wrong population | |
| 867 | Fernández, M. D. M., et al. (2017). "Using communication and visualization technologies with senior citizens to facilitate cultural access and self-improvement." COMPUTERS IN HUMAN BEHAVIOR 66: 329-344. | wrong design | |
| 868 | Fernández, S., et al. (2021). "New palynological data from the Late Pleistocene glacial refugium of South-West Iberia: The case of Donana." REVIEW OF PALAEOBOTANY AND PALYNOLOGY 290. | wrong population | |
| 869 | Fernández-Buendía, S., et al. (2024). "Technology-supported treatments for adjustment disorder: A systematic review and preliminary meta-analysis." Journal of Affective Disorders 347: 29-38. | wrong population | |
| 870 | Fernández-Crespo, T., et al. (2022). "The "post-weanling's conundrum": exploring the impact of infant and child feeding practices on early mortality in the Bronze Age burial cave of Moro de Alins, north-eastern Iberia, through stable isotope analysis." ARCHAEOLOGICAL AND ANTHROPOLOGICAL SCIENCES 14(10). | wrong population | |
| 871 | Fernández-Diego, C., et al. (2021). "Fracture toughness as an alternative approach to quantify the ageing of insulation paper in oil." CELLULOSE 28(18): 11533-11550. | wrong population | |
| 872 | Ferrand, M., et al. (2015). "A Cognitive and Virtual Reality Treatment Program for the Fear of Flying." AEROSPACE MEDICINE AND HUMAN PERFORMANCE 86(8): 723-727. | wrong population | |
| 873 | Ferrari, M., et al. (2020). "Digital Game Interventions for Youth Mental Health Services (Gaming My Way to Recovery): Protocol for a Scoping Review." JMIR RESEARCH PROTOCOLS 9(6). | wrong population | |
| 874 | Ferrari, M. A., et al. (2021). "Fully-resolved simulations of a sphere settling in an initially unstructured thixo-viscoplastic fluid." JOURNAL OF NON-NEWTONIAN FLUID MECHANICS 294. | wrong population | |
| 875 | Ferraz, D. D., et al. (2018). "The Effects of Functional Training, Bicycle Exercise, and Exergaming on Walking Capacity of Elderly Patients With Parkinson Disease: A Pilot Randomized Controlled Single-blinded Trial." Arch Phys Med Rehabil 99(5): 826-833. | wrong intervention | |
| 876 | Ferraz, D. D., et al. (2018). "The Effects of Functional Training, Bicycle Exercise, and Exergaming on Walking Capacity of Elderly Patients With Parkinson Disease: A Pilot Randomized Controlled Single-blinded Trial." ARCHIVES OF PHYSICAL MEDICINE AND REHABILITATION 99(5): 826-833. | wrong intervention | |
| 877 | Ferraz-Torres, M., et al. (2022). ""Passive or interactive virtual reality? The effectiveness for pain and anxiety reduction in pediatric patients"." VIRTUAL REALITY 26(4): 1307-1316. | wrong population | |
| 878 | Ferraz-Torres, M., et al. (2023). "Can virtual reality reduce pain and anxiety in pediatric emergency care and promote positive response of parents of children? A quasi-experimental study." INTERNATIONAL EMERGENCY NURSING 68. | wrong population | |
| 879 | Ferreira, L. D. A., et al. (2020). "User Experience of Interactive Technologies for People With Dementia: Comparative Observational Study." JMIR SERIOUS GAMES 8(3). | wrong design | |
| 880 | Ferreira, S. S., et al. (2022). "Elderberry (Sambucus nigra L.) extracts promote anti-inflammatory and cellular antioxidant activity." Food Chem X 15: 100437. | wrong population | |
| 881 | Ferreira-Brito, F., et al. (2020). "Photo-Realistic Interactive Virtual Environments for Neurorehabilitation in Mild Cognitive Impairment (NeuroVRehab.PT): A Participatory Design and Proof-of-Concept Study." J Clin Med 9(12). | wrong design | |
| 882 | Festari, C., et al. (2023). "European consensus for the diagnosis of MCI and mild dementia: Preparatory phase." Alzheimers Dement 19(5): 1729-1741. | wrong population | |
| 883 | Filbay, S., et al. (2019). "Quality of Life and Life Satisfaction in Former Athletes: A Systematic Review and Meta-Analysis." SPORTS MEDICINE 49(11): 1723-1738. | wrong design | |
| 884 | Filbay, S. R., et al. (2022). "Long-term quality of life, work limitation, physical activity, economic cost and disease burden following ACL and meniscal injury: a systematic review and meta-analysis for the OPTIKNEE consensus." Br J Sports Med 56(24): 1465-1474. | wrong design | |
| 885 | Finch, C. E. and S. N. Austad (2011). "Blind cave salamanders age very slowly: A new member of Methuselah's Bestiary." BIOESSAYS 33(1): 27-29. | wrong design | |
| 886 | Finkelstein, J. B., et al. (2020). "Anxiety, distress, and pain in pediatric urodynamics." NEUROUROLOGY AND URODYNAMICS 39(4): 1178-1184. | wrong population | |
| 887 | Finlay, M. J., et al. (2022). "The prevalence of pre-conditioning and recovery strategies in senior elite and non-elite amateur boxing." Phys Sportsmed 50(4): 323-331. | wrong population | |
| 888 | Finnegan, D. J. and S. Campbell (2023). "Tackling Loneliness and Isolation in Older Adults With Virtual Reality: How do We Move Forward?" GERONTOLOGY AND GERIATRIC MEDICINE 9. | wrong design | |
| 889 | Fiocco, A. J., et al. (2021). "Virtual tourism for older adults living in residential care: A mixed-methods study." PLoS ONE 16(5): 15. | wrong intervention | |
| 890 | Firth, J., et al. (2018). "Digital Technologies in the Treatment of Anxiety: Recent Innovations and Future Directions." Curr Psychiatry Rep 20(6): 44. | wrong design | |
| 891 | Firth, J., et al. (2024). "From "online brains" to "online lives": understanding the individualized impacts of Internet use across psychological, cognitive and social dimensions." WORLD PSYCHIATRY 23(2): 176-190. | wrong design | |
| 892 | Firth, J., et al. (2019). "The "online brain": how the Internet may be changing our cognition." World Psychiatry 18(2): 119-129. | wrong design | |
| 893 | Firth, J., et al. (2019). "The “online brain”: How the Internet may be changing our cognition." World Psychiatry 18(2): 119-129. | wrong design | |
| 894 | Fishbein, D. H. and J. Williams (2021). "Latent Class Analysis of Individual-Level Characteristics Predictive of Intervention Outcomes in Urban Male Adolescents." RESEARCH ON CHILD AND ADOLESCENT PSYCHOPATHOLOGY 49(9): 1139-1149. | wrong population | |
| 895 | Fisher, J. M., et al. (2016). "Training tomorrow's doctors to safeguard the patients of today: Using medical student simulation training to explore barriers to recognition of elder abuse." Journal of the American Geriatrics Society 64(1): 168-173. | wrong population | |
| 896 | Fitzgerald, K., et al. (2022). "Entertainment and Social Media Use During Social Distancing: Examining Trait Differences in Transportability and Need for Social Assurance." PSYCHOLOGY OF POPULAR MEDIA 11(3): 305-310. | wrong population | |
| 897 | Fleming, T. M., et al. (2017). "Serious Games and Gamification for Mental Health: Current Status and Promising Directions." FRONTIERS IN PSYCHIATRY 7. | wrong design | |
| 898 | Flores, A., et al. (2023). "Using Immersive Virtual Reality Distraction to Reduce Fear and Anxiety before Surgery." HEALTHCARE 11(19). | wrong design | |
| 899 | Flores, A., et al. (2018). "The Use of Virtual Reality to Facilitate Mindfulness Skills Training in Dialectical Behavioral Therapy for Spinal Cord Injury: A Case Study." FRONTIERS IN PSYCHOLOGY 9. | wrong design | |
| 900 | Flück, C. E. and T. Güran (2000). Ambiguous Genitalia in the Newborn. Endotext. K. R. Feingold, B. Anawalt, M. R. Blackman et al. South Dartmouth (MA), MDText.com, Inc. | wrong population | |
| 901 | Flynn, A., et al. (2024). "A Multi-User Virtual Reality Social Connecting Space for People Living with Dementia and Their Support Persons: A Participatory Action Research Study." INTERNATIONAL JOURNAL OF HUMAN-COMPUTER INTERACTION. | wrong design | |
| 902 | Foloppe, D. A., et al. (2018). "The potential of virtual reality-based training to enhance the functional autonomy of Alzheimer's disease patients in cooking activities: A single case study." NEUROPSYCHOLOGICAL REHABILITATION 28(5): 709-733. | wrong design | |
| 903 | Fong, D. Y. T., et al. (2022). "Average and individual differences between the 12-item MOS Short-form Health Survey version 2 (SF-12 V.2) and the veterans RAND 12-item Health Survey (VR-12) in the Chinese population." HEALTH AND QUALITY OF LIFE OUTCOMES 20(1). | wrong intervention | |
| 904 | Formosa, N. J., et al. (2018). "Testing the efficacy of a virtual reality-based simulation in enhancing users' knowledge, attitudes, and empathy relating to psychosis." AUSTRALIAN JOURNAL OF PSYCHOLOGY 70(1): 57-65. | wrong population | |
| 905 | Fortuna, K., et al. (2022). "Certified Peer Support Specialists Training in Technology and Delivery of Digital Peer Support Services: Cross-sectional Study." JMIR FORMATIVE RESEARCH 6(12). | wrong design | |
| 906 | Foster, P. P. (2015). "Role of physical and mental training in brain network configuration." Frontiers in Aging Neuroscience 7: 13. | wrong design | |
| 907 | Franzen, M., et al. (2021). "Victims of Bullying: Emotion Recognition and Understanding." FRONTIERS IN PSYCHOLOGY 12. | wrong design | |
| 908 | Frediani, S., et al. (2024). "Case Report: Double Pectus Up in severe pectus excavatum, the new frontier of modified taulinoplasty." FRONTIERS IN PEDIATRICS 12. | wrong design | |
| 909 | Fredj, N., et al. (2011). "Effect of mechanical stresses on marine organic coating ageing approached by EIS measurements." PROGRESS IN ORGANIC COATINGS 72(3): 260-268. | wrong design | |
| 910 | Fredj, N., et al. (2012). "Ageing of marine coating in natural and artificial seawater under mechanical stresses." PROGRESS IN ORGANIC COATINGS 74(2): 391-399. | wrong design | |
| 911 | Freedman, S. A., et al. (2014). "Early intervention for preventing posttraumatic stress disorder: an Internet-based virtual reality treatment." EUROPEAN JOURNAL OF PSYCHOTRAUMATOLOGY 6. | wrong population | |
| 912 | Freedman, S. A., et al. (2010). "Prolonged Exposure and Virtual Reality-Enhanced Imaginal Exposure for PTSD following a Terrorist Bulldozer Attack: A Case Study." CYBERPSYCHOLOGY BEHAVIOR AND SOCIAL NETWORKING 13(1): 95-101. | wrong population | |
| 913 | Freeman, A. M. and P. Matto (2024). Adenopathy. StatPearls. Treasure Island (FL), StatPearls Publishing | wrong design | |
| 914 | Freeman, D., et al. (2021). "Comparison of a theoretically driven cognitive therapy (the Feeling Safe Programme) with befriending for the treatment of persistent persecutory delusions: a parallel, single-blind, randomised controlled trial." Lancet Psychiatry 8(8): 696-707. | wrong population | |
| 915 | Freeman, D., et al. (2023). "Randomised controlled trial of automated VR therapy to improve positive self-beliefs and psychological well-being in young people diagnosed with psychosis: a study protocol for the Phoenix VR self-confidence therapy trial." BMJ OPEN 13(12). | wrong population | |
| 916 | Freeman, D., et al. (2018). "Automated psychological therapy using immersive virtual reality for treatment of fear of heights: A single-blind, parallel-group, randomised controlled trial." The Lancet Psychiatry 5(8): 625-632. | wrong population | |
| 917 | Freeman, D., et al. (2022). "Automated virtual reality therapy to treat agoraphobic avoidance and distress in patients with psychosis (gameChange): a multicentre, parallel-group, single-blind, randomised, controlled trial in England with mediation and moderation analyses." Lancet Psychiatry 9(5): 375-388. | duplicate |  |
| 918 | Freeman, D., et al. (2022). "Automated virtual reality therapy to treat agoraphobic avoidance and distress in patients with psychosis gameChange): a multicentre, parallel-group, single-blind, randomised, controlled trial in England with mediation and moderation analyses." LANCET PSYCHIATRY 9(5): 375-388. | duplicate |  |
| 919 | Freeman, D., et al. (2022). "Automated virtual reality therapy to treat agoraphobic avoidance and distress in patients with psychosis (gameChange): A multicentre, parallel-group, single-blind, randomised, controlled trial in England with mediation and moderation analyses." The Lancet Psychiatry 9(5): 375-388. | duplicate |  |
| 920 | Freeman, D., et al. (2023). "Automated virtual reality cognitive therapy versus virtual reality mental relaxation therapy for the treatment of persistent persecutory delusions in patients with psychosis (THRIVE): a parallel-group, single-blind, randomised controlled trial in England with mediation analyses." LANCET PSYCHIATRY 10(11): 836-847. | duplicate |  |
| 921 | Freeman, D., et al. (2023). "Automated virtual reality cognitive therapy versus virtual reality mental relaxation therapy for the treatment of persistent persecutory delusions in patients with psychosis (THRIVE): A parallel-group, single-blind, randomised controlled trial in England with mediation analyses." The Lancet Psychiatry 10(11): 836-847. | duplicate |  |
| 922 | Frueh, B. C. (2015). "Solving Mental Healthcare Access Problems in the Twenty-first Century." AUSTRALIAN PSYCHOLOGIST 50(4): 304-306. | wrong population | |
| 923 | Frueh, B. C. (2015). "Solving mental healthcare access problems in the twenty‐first century." Australian Psychologist 50(4): 304-306. | wrong population | |
| 924 | Fu, E. K., et al. (2022). "Exploring the influence of residential courtyard space landscape elements on people's emotional health in an immersive virtual environment." FRONTIERS IN PUBLIC HEALTH 10. | wrong design | |
| 925 | Fu, X. X., et al. (2024). "Implementing mobile eye tracking in psychological research: A practical guide." BEHAVIOR RESEARCH METHODS. | wrong design | |
| 926 | Fu, Y., et al. (2022). "Conceptual Design of an Extended Reality Exercise Game for the Elderly." APPLIED SCIENCES-BASEL 12(13). | wrong design | |
| 927 | Fuchs, C. J., et al. (2020). "Hot-water immersion does not increase postprandial muscle protein synthesis rates during recovery from resistance-type exercise in healthy, young males." JOURNAL OF APPLIED PHYSIOLOGY 128(4): 1012-1022. | wrong population | |
| 928 | Fuchs, L., et al. (2022). "The influence of early virtual reality intervention on pain, anxiety, and function following primary total knee arthroplasty." COMPLEMENTARY THERAPIES IN CLINICAL PRACTICE 49. | wrong population | |
| 929 | Fukase, H., et al. (2015). "Brief Communication: Size and Placement of Developing Anterior Teeth in Immature Neanderthal Mandibles From Dederiyeh Cave, Syria: Implications for Emergence of the Modern Human Chin." AMERICAN JOURNAL OF PHYSICAL ANTHROPOLOGY 156(3): 482-488. | wrong population | |
| 930 | Fukuda, T., et al. (2012). DISTRIBUTED AND SYNCHRONISED VR MEETING USING CLOUD COMPUTING <i>Availability and application to a spatial design study</i>. PROCEEDINGS OF THE 17TH INTERNATIONAL CONFERENCE ON COMPUTER-AIDED ARCHITECTURAL DESIGN RESEARCH IN ASIA (CAADRIA 2012): BEYOND CODES AND PIXELS: 203-210. | wrong population | |
| 931 | Fuller, S. J., et al. (2023). "Nasogastric tube feeding under physical restraint on paediatric wards: ethical, legal and practical considerations regarding this lifesaving intervention." BJPsych Bull 47(2): 105-110. | wrong design | |
| 932 | Fundaro, C., et al. (2023). "Technology Assisted Rehabilitation Patient Perception Questionnaire (TARPP-Q): development and implementation of an instrument to evaluate patients' perception during training." JOURNAL OF NEUROENGINEERING AND REHABILITATION 20(1). | wrong design | |
| 933 | Fundarò, C., et al. (2019). "Self-selected speed gait training in Parkinson's disease: robot-assisted gait training with virtual reality <i>versus</i> gait training on the ground." EUROPEAN JOURNAL OF PHYSICAL AND REHABILITATION MEDICINE 55(4): 456-462. | wrong population | |
| 934 | Furui, A., et al. (2018). "Mechanical simulation study of postoperative displacement of trochanteric fractures using the finite element method." Journal of orthopaedic surgery and research 13(1): 300. | wrong population | |
| 935 | Furuzawa, M., et al. (2014). "Chewing ameliorates chronic mild stress-induced bone loss in senescence-accelerated mouse (SAMP8), a murine model of senile osteoporosis." Exp Gerontol 55: 12-18. | wrong population | |
| 936 | Gadige, P. and R. Bandyopadhyay (2018). "Electric field induced gelation in aqueous nanoclay suspensions." SOFT MATTER 14(34). | wrong population | |
| 937 | Gaertner, R. J., et al. (2023). "Relaxing effects of virtual environments on the autonomic nervous system indicated by heart rate variability: A systematic review." JOURNAL OF ENVIRONMENTAL PSYCHOLOGY 88. | wrong design | |
| 938 | Gaggioli, A., et al. (2020). "Psycho-physiological Effects of a Virtual Reality Relaxation Experience after Acute Stressor Exposure." ANNUAL REVIEW OF CYBERTHERAPY AND TELEMEDICINE 18: 123-126. | wrong population | |
| 939 | Gaggioli, A., et al. (2017). "“Positive bike”—An immersive biking experience for combined physical and cognitive training of elderly patients." Annual Review of CyberTherapy and Telemedicine 15: 196-199. | wrong outcomes | |
| 940 | Gaggioli, A., et al. (2011). Ubiquitous Health in Practice: The Interreality Paradigm. MEDICINE MEETS VIRTUAL REALITY 18. 163: 185-191. | wrong population | |
| 941 | Gagnier, J., et al. (2021). "A 5-Year Follow-up of Patients Treated for Full-Thickness Rotator Cuff Tears: A Prospective Cohort Study." ORTHOPAEDIC JOURNAL OF SPORTS MEDICINE 9(9). | wrong design | |
| 942 | Gagnon, D., et al. (2010). "Cold-Water Immersion and the Treatment of Hyperthermia: Using 38.6 °C as a Safe Rectal Temperature Cooling Limit." JOURNAL OF ATHLETIC TRAINING 45(5): 439-444. | wrong design | |
| 943 | Gaikwad, S. B., et al. (2018). "Effect of Gaze Stability Exercises on Chronic Motion Sensitivity: A Randomized Controlled Trial." JOURNAL OF NEUROLOGIC PHYSICAL THERAPY 42(2): 72-79. | wrong intervention | |
| 944 | Gall, D., et al. (2016). Remind Me: An Adaptive Recommendation-Based Simulation of Biographic Associations. PROCEEDINGS OF THE 21ST INTERNATIONAL CONFERENCE ON INTELLIGENT USER INTERFACES (IUI'16): 191-195. | wrong design | |
| 945 | Gallagher, A., et al. (2021). "Findings from a mixed-methods pragmatic cluster trial evaluating the impact of ethics education interventions on residential care-givers." NURSING INQUIRY 28(2). | wrong design | |
| 946 | Gallagher, A., et al. (2021). "Findings from a mixed‐methods pragmatic cluster trial evaluating the impact of ethics education interventions on residential care‐givers." Nursing Inquiry 28(2): 12. | wrong intervention | |
| 947 | Gallant, L. R., et al. (2020). "A bat guano deposit in Jamaica recorded agricultural changes and metal exposure over the last > 4300 years." PALAEOGEOGRAPHY PALAEOCLIMATOLOGY PALAEOECOLOGY 538. | wrong design | |
| 948 | Gallardo, D. and R. F. Hoyt (2018). "Surgical loupes: A useful tool for small target rodent procedures." Journal of the American Association for Laboratory Animal Science 57(5): 553. | wrong design | |
| 949 | Galovic, M., et al. (2021). "Seizures and Epilepsy After Stroke: Epidemiology, Biomarkers and Management." Drugs and Aging 38(4): 285-299. | wrong design | |
| 950 | Galperin, I., et al. (2023). "Treadmill training with virtual reality to enhance gait and cognitive function among people with multiple sclerosis: a randomized controlled trial." JOURNAL OF NEUROLOGY 270(3): 1388-1401. | wrong population | |
| 951 | Gaman, A., et al. (2017). "Understanding Patterns of Intimate Partner Abuse in Male-Male, Male-Female, and Female-Female Couples." PSYCHIATRIC QUARTERLY 88(2): 335-347. | wrong intervention | |
| 952 | Gamaoun, F. (2021). "Strain Rate Effect upon Mechanical Behaviour of Hydrogen-Charged Cycled NiTi Shape Memory Alloy." Materials (Basel) 14(16). | wrong population | |
| 953 | Gambella, E., et al. (2022). "An integrated intervention of computerized cognitive training and physical exercise in virtual reality for people with Alzheimer's disease: The jDome study protocol." FRONTIERS IN NEUROLOGY 13. | wrong intervention | |
| 954 | Gamito, P., et al. (2020). "Virtual reality-based cognitive stimulation to improve cognitive functioning in community elderly: A controlled study." Cyberpsychology, Behavior, and Social Networking 23(3): 150-156. | wrong design | |
| 955 | Gamito, P., et al. (2019). "Cognitive stimulation of elderly individuals with instrumental virtual reality-based activities of daily life: Pre-post treatment study." Cyberpsychology, Behavior, and Social Networking 22(1): 69-75. | wrong design | |
| 956 | Gamito, P., et al. (2010). "PTSD elderly war veterans: A clinical controlled pilot study." Cyberpsychology, Behavior, and Social Networking 13(1): 43-48. | wrong intervention | |
| 957 | Ganschow, B., et al. (2024). "Feeling connected but dissimilar to one's future self reduces the intention-behavior gap." PLOS ONE 19(7). | wrong design | |
| 958 | Gao, D., et al. (2024). "The application of virtual reality meditation and mind-body exercises among older adults." Front Psychol 15: 1303880. | wrong design | |
| 959 | Gao, D., et al. (2024). "The application of virtual reality meditation and mind-body exercises among older adults." FRONTIERS IN PSYCHOLOGY 15. | wrong design | |
| 960 | Gao, X. D., et al. (2019). "Prediction of Corrosive Fatigue Life of Submarine Pipelines of API 5L X56 Steel Materials." MATERIALS 12(7). | wrong design | |
| 961 | Gao, Y. (2022). "Application of Virtual Reality Technology in the Recognition System for Overcoming Anxiety and Psychological Pressure of Family Elderly." MOBILE INFORMATION SYSTEMS 2022. | wrong design | |
| 962 | Gao, Y., et al. (2021). "Effects of virtual reality-based intervention on cognition, motor function, mood, and activities of daily living in patients with chronic stroke: A systematic review and meta-analysis of randomized controlled trials." Frontiers in Aging Neuroscience 13: 13. | wrong design | |
| 963 | Gao, Y., et al. (2021). "Effects of Virtual Reality-Based Intervention on Cognition, Motor Function, Mood, and Activities of Daily Living in Patients With Chronic Stroke: A Systematic Review and Meta-Analysis of Randomized Controlled Trials." FRONTIERS IN AGING NEUROSCIENCE 13. | wrong design | |
| 964 | Gao, Z. and T. Braud (2023). "VR-driven museum opportunities: digitized archives in the age of the metaverse." ARTNODES(32). | wrong design | |
| 965 | García, A. T., et al. (2024). "Efficacy of a Virtual Reality Intervention for Reducing Anxiety, Depression, and Increasing Disease Coping in Patients with Breast Cancer Before Their First Chemotherapy Dose." COGNITIVE THERAPY AND RESEARCH 48(3): 451-465. | wrong design | |
| 966 | Garcia Campuzano, M. T. and Z. Moussavi (2012). "The effect of aging on brain temporal perception using Virtual Reality Neurocognitive (VRN) experiments." Annu Int Conf IEEE Eng Med Biol Soc 2012: 4808-4811. | wrong design | |
| 967 | Garcia, L., et al. (2023). "The usability of virtual reality to train individuals in responding to behaviors related to dementia." Front Dement 2: 1237127. | wrong design | |
| 968 | Garcia, L. M., et al. (2021). "An 8-Week Self-Administered At-Home Behavioral Skills-Based Virtual Reality Program for Chronic Low Back Pain: Double-Blind, Randomized, Placebo-Controlled Trial Conducted During COVID-19." JOURNAL OF MEDICAL INTERNET RESEARCH 23(2). | wrong population | |
| 969 | Garcia, M. K., et al. (2017). "Cardiorespiratory performance of coronary artery disease patients on land versus underwater treadmill tests: a comparative study." CLINICS 72(11): 667-674. | wrong design | |
| 970 | García-Alvarez, S. M., et al. (2012). "Numb Chin Syndrome with Vagal and Hypoglossal Paralysis: An Initial Sign of an Uncommon Diagnosis." AMERICAN JOURNAL OF THE MEDICAL SCIENCES 344(3): 241-244. | wrong design | |
| 971 | García-Batista, Z. E., et al. (2022). "Design and validation of virtual environments for the treatment of cleaning obsessive-compulsive disorder." HELIYON 8(12). | wrong design | |
| 972 | García-Betances, R. I., et al. (2015). "A succinct overview of virtual reality technology use in Alzheimer's disease." FRONTIERS IN AGING NEUROSCIENCE 7. | wrong design | |
| 973 | García-Muñoz, C., et al. (2022). "Immersive Virtual Reality and Vestibular Rehabilitation in Multiple Sclerosis: Case Report." JMIR SERIOUS GAMES 10(1). | wrong design | |
| 974 | Garrido, L. E., et al. (2022). "Focusing on cybersickness: pervasiveness, latent trajectories, susceptibility, and effects on the virtual reality experience." VIRTUAL REALITY 26(4): 1347-1371. | wrong design | |
| 975 | Garrocho-Rangel, A., et al. (2018). "A video eyeglasses/earphones system as distracting method during dental treatment in children: A crossover randomised and controlled clinical trial." EUROPEAN JOURNAL OF PAEDIATRIC DENTISTRY 19(1): 74-79. | wrong population | |
| 976 | Garza-Teran, G., et al. (2022). "Impact of Contact With Nature on the Wellbeing and Nature Connectedness Indicators After a Desertic Outdoor Experience on Isla Del Tiburon." FRONTIERS IN PSYCHOLOGY 13. | wrong population | |
| 977 | Gass, C. S., et al. (2021). "The Cognitive Difficulties Scale (CDS): Psychometric characteristics in a clinical referral sample." Journal of the International Neuropsychological Society 27(4): 351-364. | wrong population | |
| 978 | Gates, N. J., et al. (2019). "Computerised cognitive training for maintaining cognitive function in cognitively healthy people in midlife." Cochrane Database Syst Rev 3(3): Cd012278. | wrong intervention | |
| 979 | Gates, N. J., et al. (2020). "Computerised cognitive training for 12 or more weeks for maintaining cognitive function in cognitively healthy people in late life." Cochrane Database Syst Rev 2(2): Cd012277. | wrong intervention | |
| 980 | Gates, N. J., et al. (2019). "Computerised cognitive training for maintaining cognitive function in cognitively healthy people in midlife." COCHRANE DATABASE OF SYSTEMATIC REVIEWS(3). | wrong intervention | |
| 981 | Gates, N. J., et al. (2019). "Computerised cognitive training for preventing dementia in people with mild cognitive impairment." Cochrane Database Syst Rev 3(3): Cd012279. | wrong intervention | |
| 982 | Gates, N. J., et al. (2019). "Computerised cognitive training for preventing dementia people with mild cognitive impairment." COCHRANE DATABASE OF SYSTEMATIC REVIEWS(3). | wrong intervention | |
| 983 | Gauthier, B., et al. (2020). "First-person body view modulates the neural substrates of episodic memory and autonoetic consciousness: A functional connectivity study." Neuroimage 223: 117370. | wrong population | |
| 984 | Gawronski, S. W., et al. (2010). AIR PHYTOREMEDIATION - REMOVAL OF PARTICULAR MATTER, PAHs AND METALS BY HIGHER PLANTS. BIOTECHNIQUES FOR AIR POLLUTION CONTROL: 243-246. | wrong design | |
| 985 | Gayer-Anderson, C., et al. (2024). "Immersive virtual reality as a novel approach to investigate the association between adverse events and adolescent paranoid ideation." SOCIAL PSYCHIATRY AND PSYCHIATRIC EPIDEMIOLOGY. | wrong design | |
| 986 | Ge, S. Q., et al. (2018). "Technology-based cognitive training and rehabilitation interventions for individuals with mild cognitive impairment: a systematic review." BMC GERIATRICS 18. | wrong design | |
| 987 | Geers, A. L., et al. (2024). "A test of pre-exposure spacing and multiple context pre-exposure on the mechanisms of latent inhibition of dental fear: A study protocol." BMC PSYCHOLOGY 12(1). | wrong design | |
| 988 | Geers, A. L., et al. (2024). "A study protocol testing pre-exposure dose and compound pre-exposure on the mechanisms of latent inhibition of dental fear." BMC PSYCHOLOGY 12(1). | wrong design | |
| 989 | Gega, L., et al. (2022). "Digital interventions in mental health: evidence syntheses and economic modelling." Health Technol Assess 26(1): 1-182. | wrong design | |
| 990 | Genç, S. G., et al. (2023). "Effects of structured exercise program on severity of dizziness, kinesiophobia, balance, fatigue, quality of sleep, activities of daily living, and quality of life in bilateral vestibular hypofunction." Medicine (Baltimore) 102(30): e34435. | wrong design | |
| 991 | Gendron, D. J. and M. R. Hamblin (2019). "Applications of Photobiomodulation Therapy to Musculoskeletal Disorders and Osteoarthritis with Particular Relevance to Canada." Photobiomodul Photomed Laser Surg 37(7): 408-420. | wrong intervention | |
| 992 | Genova, H. M., et al. (2024). "A Combined Interventional Approach to Train Interview Skills in Autistic Transition-Age Youth." JOURNAL OF AUTISM AND DEVELOPMENTAL DISORDERS. | wrong population | |
| 993 | Genova, H. M., et al. (2021). "A pilot RCT of virtual reality job interview training in transition-age youth on the autism spectrum." RESEARCH IN AUTISM SPECTRUM DISORDERS 89. | wrong population | |
| 994 | George, C., et al. (2023). "MENTAL HEALTH NEEDS OF WOMEN IN SMALL ISLAND DEVELOPING STATES DURING COVID 19: A POPULATION SURVEY." Journal of Epidemiology and Community Health 77: A148. | wrong design | |
| 995 | George, C. L., et al. (2023). "Virtual Reality Biking Reduces Cortisol Levels and Repetitive Behaviors in Adults with Autism Spectrum Disorder." ADVANCES IN NEURODEVELOPMENTAL DISORDERS 7(4): 616-628. | wrong design | |
| 996 | George, J., et al. (2017). "Predicting Functional Outcomes After Above Knee Amputation for Infected Total Knee Arthroplasty." JOURNAL OF ARTHROPLASTY 32(2): 532-536. | wrong population | |
| 997 | Georgiopoulos, P., et al. (2014). "Τhe effect of silica nanoparticles on the thermomechanical properties and degradation behavior of polylactic acid." J Biomater Appl 29(5): 662-674. | wrong population | |
| 998 | Geraghty, A. W., et al. (2014). "Internet-based vestibular rehabilitation for adults aged 50 years and over: a protocol for a randomised controlled trial." BMJ Open 4(7): e005871. | wrong design | |
| 999 | Geraghty, A. W. A., et al. (2014). "Internet-based vestibular rehabilitation for adults aged 50 years and over: a protocol for a randomised controlled trial." BMJ OPEN 4(7). | wrong design | |
| 1000 | Gerber, K., et al. (2022). "'Unprepared for the depth of my feelings'-Capturing grief in older people through research poetry." AGE AND AGEING 51(3). | wrong design | |
| 1001 | Gerber, S. M., et al. (2019). "Perception and Performance on a Virtual Reality Cognitive Stimulation for Use in the Intensive Care Unit: A Non-randomized Trial in Critically Ill Patients." FRONTIERS IN MEDICINE 6. | wrong population | |
| 1002 | Gerber, S. M., et al. (2017). "Visuo-acoustic stimulation that helps you to relax: A virtual reality setup for patients in the intensive care unit." Scientific reports 7(1): 13228. | wrong design | |
| 1003 | Gerçeker, G., et al. (2020). "Effects of virtual reality on pain, fear and anxiety during blood draw in children aged 5-12 years old: A randomised controlled study." JOURNAL OF CLINICAL NURSING 29(7-8): 1151-1161. | wrong population | |
| 1004 | Gerçeker, G., et al. (2018). "Effects of Virtual Reality and External Cold and Vibration on Pain in 7-to 12-Year-Old Children During Phlebotomy: A Randomized Controlled Trial." JOURNAL OF PERIANESTHESIA NURSING 33(6): 981-989. | wrong population | |
| 1005 | Gerçeker, G. O., et al. (2024). "The effects of virtual reality and stress ball distraction on procedure-related emotional appearance, pain, fear, and anxiety during phlebotomy in children: A randomized controlled study." JOURNAL OF PEDIATRIC NURSING-NURSING CARE OF CHILDREN & FAMILIES 79: 197-204. | wrong population | |
| 1006 | Gessert, A. (2016). "Geomorphology of the Slovak Karst (Eastern Part)." JOURNAL OF MAPS 12: 285-288. | wrong population | |
| 1007 | Ghasempeyvandi, M. and H. Torkan (2023). "The effect of virtual reality exposure therapy on focus of attention, self-criticism, and interpretation bias in university students with social anxiety." JOURNAL OF EDUCATION AND HEALTH PROMOTION 12(1). | wrong population | |
| 1008 | Ghelich, S., et al. (2014). "Effect of lead treatment on medicarpin accumulation and on the gene expression of key enzymes involved in medicarpin biosynthesis in <i>Medicago sativa</i> L." ENVIRONMENTAL SCIENCE AND POLLUTION RESEARCH 21(24): 14091-14098. | wrong population | |
| 1009 | Ghita, A., et al. (2021). "Attentional Bias, Alcohol Craving, and Anxiety Implications of the Virtual Reality Cue-Exposure Therapy in Severe Alcohol Use Disorder: A Case Report." FRONTIERS IN PSYCHOLOGY 12. | wrong population | |
| 1010 | Ghita, A., et al. (2019). "Craving and Anxiety Responses as Indicators of the Efficacy of Virtual Reality-Cue Exposure Therapy in Patients Diagnosed with Alcohol use Disorder." ANNUAL REVIEW OF CYBERTHERAPY AND TELEMEDICINE 17: 77-82. | wrong design | |
| 1011 | Ghita, A., et al. (2019). "Identifying Triggers of Alcohol Craving to Develop Effective Virtual Environments for Cue Exposure Therapy." FRONTIERS IN PSYCHOLOGY 10. | wrong design | |
| 1012 | Ghous, M., et al. (2024). "Comparison of Nonimmersive Virtual Reality and Task-Oriented Circuit Training on Gait, Balance and Cognition Among Elderly Population: A Single-Blind Randomized Control Trial." GAMES FOR HEALTH JOURNAL 13(3): 164-171. | wrong intervention | |
| 1013 | Ghous, M., et al. (2024). "Comparison of Nonimmersive Virtual Reality and Task-Oriented Circuit Training on Gait, Balance, and Cognition Among Elderly Population: A Single-Blind Randomized Control Trial." Games Health J 13(3): 164-171. | wrong intervention | |
| 1014 | Giani, L., et al. (2022). "Age-related hallmarks of psychopathology in Cornelia de Lange and Rubinstein-Taybi syndromes." RESEARCH IN DEVELOPMENTAL DISABILITIES 126. | wrong design | |
| 1015 | Gicas, K. M., et al. (2020). "Cognitive decline and mortality in a community-based sample of homeless and precariously housed adults: 9-year prospective study." BJPsych Open 6(2): e21. | wrong design | |
| 1016 | Gil, T. E. and J. L. Gil (2023). "Artificially Sweetened Beverages Beyond the Metabolic Risks: A Systematic Review of the Literature." CUREUS JOURNAL OF MEDICAL SCIENCE 15(1). | wrong design | |
| 1017 | Gilat, M., et al. (2019). "Functional MRI to Study Gait Impairment in Parkinson's Disease: a Systematic Review and Exploratory ALE Meta-Analysis." CURRENT NEUROLOGY AND NEUROSCIENCE REPORTS 19(8). | wrong design | |
| 1018 | Gilbertson, C. J., et al. (2024). "Effect of using virtual reality to manage needle phobia in adults undergoing medical procedures: A rapid review." Journal of Perioperative Nursing 37(1): e-29-e-40. | wrong design | |
| 1019 | Gilboa, Y., et al. (2019). "Effectiveness of a tele-rehabilitation intervention to improve performance and reduce morbidity for people post hip fracture - study protocol for a randomized controlled trial." BMC Geriatr 19(1): 135. | wrong design | |
| 1020 | Gilchrist, A. S., et al. (2010). "Do Biomechanical Properties of Anterior Vaginal Wall Prolapse Tissue Predict Outcome of Surgical Repair?" JOURNAL OF UROLOGY 183(3): 1069-1073. | wrong population | |
| 1021 | Giordano, C., et al. (2022). "ERECTILE DYSFUNCTION (ED) IN PATIENTS WITH LIGHT-CHAIN AMYLOIDOSIS (AL): DIAGNOSIS AND CORRELATION WITH THE HEMATOLOGIC DISEASE." Haematologica 107(SUPPL 1): 85-86. | wrong design | |
| 1022 | Giordano, C., et al. (2021). "ERECTILE DYSFUNCTION (ED) IN PATIENTS WITH LIGHTCHAIN AMYLOIDOSIS (AL): DIAGNOSIS AND CORRELATION WITH THE HEMATOLOGIC DISEASE." Haematologica 106: 171-172. | wrong design | |
| 1023 | Girbino, K. L., et al. (2021). "Understanding the Main Predictors of Length of Stay After Total Hip Arthroplasty: Patient-Related or Procedure-Related Risk Factors?" JOURNAL OF ARTHROPLASTY 36(5): 1663-+. | wrong design | |
| 1024 | Girishan Prabhu, V., et al. (2024). "Designing and developing a nature-based virtual reality with heart rate variability biofeedback for surgical anxiety and pain management: Evidence from total knee arthroplasty patients." Aging & Mental Health 28(5): 738-753. | wrong design | |
| 1025 | Girondini, M., et al. (2023). "Speaking in front of cartoon avatars: A behavioral and psychophysiological study on how audience design impacts on public speaking anxiety in virtual environments." INTERNATIONAL JOURNAL OF HUMAN-COMPUTER STUDIES 179. | wrong design | |
| 1026 | Giwa, S., et al. (2020). "Virtual Social Work Care with Older Black Adults: A Culturally Relevant Technology-Based Intervention to Reduce Social Isolation and Loneliness in a Time of Pandemic." Journal of Gerontological Social Work 63(6/7): 679-681. | wrong design | |
| 1027 | Gjærde, L. K., et al. (2021). "Play interventions for paediatric patients in hospital: a scoping review." BMJ OPEN 11(7). | wrong design | |
| 1028 | Gjerde, B., et al. (2017). "Morphological and molecular characterization of four <i>Sarcocystis</i> spp., including <i>Sarcocystis linearis</i> n. sp., from roe deer (<i>Capreolus capreolus</i>) in Italy." PARASITOLOGY RESEARCH 116(4): 1317-1338. | wrong design | |
| 1029 | Glassburn, S. L., et al. (2022). "Preparing advanced learners for geriatric team care: A short-term curricular model that works." Gerontol Geriatr Educ 43(1): 102-118. | wrong design | |
| 1030 | Goedeken, S., et al. (2018). "Encoding strategy training and self-reported everyday prospective memory in people with Parkinson disease: a randomized-controlled trial." CLINICAL NEUROPSYCHOLOGIST 32(7): 1282-1302. | wrong intervention | |
| 1031 | Gokceoglu, C., et al. (2016). "Safety assessment of limestone-based engineering structures to be partially flooded by dam water: A case study from northeastern Turkey." ENGINEERING GEOLOGY 209: 44-55. | wrong design | |
| 1032 | Goktas, N. and D. Avci (2023). "The effect of visual and/or auditory distraction techniques on children's pain, anxiety and medical fear in invasive procedures: A randomized controlled trial." JOURNAL OF PEDIATRIC NURSING-NURSING CARE OF CHILDREN & FAMILIES 73: e27-e35. | wrong population | |
| 1033 | Gold, J. I., et al. (2024). "Exploring Relations Between Unique Patient Characteristics and Virtual Reality Immersion Level on Anxiety and Pain in Patients Undergoing Venipuncture: Secondary Analysis of a Randomized Control Trial." JOURNAL OF MEDICAL INTERNET RESEARCH 26. | wrong design | |
| 1034 | Gold, J. I., et al. (2021). ""Doc McStuffins: Doctor for a Day" Virtual Reality (DocVR) for Pediatric Preoperative Anxiety and Satisfaction: Pediatric Medical Technology Feasibility Study." JOURNAL OF MEDICAL INTERNET RESEARCH 23(4). | wrong population | |
| 1035 | Gold, J. I. and N. E. Mahrer (2018). "Is Virtual Reality Ready for Prime Time in the Medical Space? A Randomized Control Trial of Pediatric Virtual Reality for Acute Procedural Pain Management." JOURNAL OF PEDIATRIC PSYCHOLOGY 43(3): 266-275. | wrong population | |
| 1036 | Gold, J. I., et al. (2021). "Effect of an Immersive Virtual Reality Intervention on Pain and Anxiety Associated With Peripheral Intravenous Catheter Placement in the Pediatric Setting A Randomized Clinical Trial." JAMA NETWORK OPEN 4(8). | wrong population | |
| 1037 | Goldberg, J., et al. (2014). "The association of PTSD with physical and mental health functioning and disability (VA Cooperative Study #569: The course and consequences of posttraumatic stress disorder in Vietnam-era Veteran twins)." Quality of Life Research: An International Journal of Quality of Life Aspects of Treatment, Care & Rehabilitation 23(5): 1579-1591. | wrong design | |
| 1038 | Goldberg, T. E., et al. (2020). "Development of an UPSA Short Form for Use in Longitudinal Studies in the Early Alzheimer's Disease Spectrum." J Prev Alzheimers Dis 7(3): 179-183. | wrong design | |
| 1039 | Goldman, R. D. and A. Behboudi (2021). "Pilot Randomized Controlled Trial of Virtual Reality vs. Standard-of-Care During Pediatric Laceration Repair." JOURNAL OF CHILD & ADOLESCENT TRAUMA 14(2): 295-298. | wrong design | |
| 1040 | Goldman, R. D. and A. Behboudi (2021). "Virtual reality for intravenous placement in the emergency department-a randomized controlled trial." EUROPEAN JOURNAL OF PEDIATRICS 180(3): 725-731. | wrong intervention | |
| 1041 | Gomaa, A. A. M., et al. (2024). "Effects of immersing Japanese quail eggs in various doses of riboflavin on reproductive, growth performance traits, blood indices and economics." POULTRY SCIENCE 103(8). | wrong intervention | |
| 1042 | Gomaa, Y. S., et al. (2024). "Role of virtual reality in examining the effect of fear of falling (FOF) on postural stability in individuals without and with Parkinson's disease in Egypt: a mixed-methods feasibility study protocol." BMJ OPEN 14(5). | wrong design | |
| 1043 | Gomberg, J., et al. (2024). "Risk of Recurrent Stroke and Mortality Among Black and White Patients With Poststroke Depression." Stroke 55(5): 1308-1316. | wrong design | |
| 1044 | Gomes, T., et al. (2014). Effect of fresh spent coffee grounds on the oxidative stress and antioxidant response in lettuce plants. VII CONGRESO IBERICO DE AGROINGENIERIA Y CIENCIAS HORTICOLAS: INNOVAR Y PRODUCIR PARA EL FUTURO. INNOVATING AND PRODUCING FOR THE FUTURE: 1395-1399. | wrong design | |
| 1045 | Gómez, I., et al. (2019). "A Virtual Reality-Based Psychological Treatment in Long-Term Hospitalization: A Case Study." CLINICAL CASE STUDIES 18(1): 3-17. | wrong design | |
| 1046 | Gomez, J., et al. (2017). "The Use of Virtual Reality Facilitates Dialectical Behavior Therapy® "Observing Sounds and Visuals" Mindfulness Skills Training Exercises for a Latino Patient with Severe Burns: A Case Study." FRONTIERS IN PSYCHOLOGY 8. | wrong design | |
| 1047 | Gómez-Morales, A., et al. (2023). "Behind the Scenes of a Technologically Enhanced Intervention for Caregivers of People With Dementia: Protocol for a Feasibility and Acceptability Study." JMIR RESEARCH PROTOCOLS 12. | wrong design | |
| 1048 | Gómez-Morales, A., et al. (2024). "Through alzheimer’s eyes: A virtual pilot intervention for family caregivers of people with dementia." Clinical Gerontologist: The Journal of Aging and Mental Health. | wrong intervention | |
| 1049 | Gómez-Polo, C., et al. (2021). "Behaviour and Anxiety Management of Paediatric Dental Patients through Virtual Reality: A Randomised Clinical Trial." JOURNAL OF CLINICAL MEDICINE 10(14). | wrong population | |
| 1050 | Gong, Z. Z., et al. (2022). "Research of 3D image processing of VR technology in medicine based on DNN." KSII TRANSACTIONS ON INTERNET AND INFORMATION SYSTEMS 16(5): 1584-1596. | wrong design | |
| 1051 | González-González, J. A., et al. (2023). "Use of audiovisual devices in transnasal endoscopy without sedation to improve tolerance. A prospective clinical trial." REVISTA DE GASTROENTEROLOGIA DE MEXICO 88(4): 347-353. | wrong intervention | |
| 1052 | González-Gualda, L. M., et al. (2024). "An exploratory study of the effect of age and gender on face scanning during affect recognition in immersive virtual reality." Scientific reports 14(1): 5553. | wrong design | |
| 1053 | González-Reimers, E., et al. (2022). "A Prehispanic infant from Tenerife with diffuse microporotic lesions." ANTHROPOLOGISCHER ANZEIGER. | wrong population | |
| 1054 | Gonzalez-Ruiz, Y., et al. (2024). "Role of projective psychological tests in patients with Prader-Willi syndrome." CHILD CARE HEALTH AND DEVELOPMENT 50(4). | wrong intervention | |
| 1055 | Gorawara-Bhat, R., et al. (2017). "Exploring physicians’ verbal and nonverbal responses to cues/concerns: Learning from incongruent communication." Patient Education and Counseling 100(11): 1979-1989. | wrong intervention | |
| 1056 | Gorinelli, S., et al. (2023). "Virtual reality acceptance and commitment therapy intervention for social and public speaking anxiety: A randomized controlled trial." JOURNAL OF CONTEXTUAL BEHAVIORAL SCIENCE 28: 289-299. | wrong population | |
| 1057 | Gormley, G., et al. (2019). "'I'M SORRY DOCTOR BUT I DIDN'T HEAR THAT.': DEVELOPING A VIRTUAL REALITY (VR) HEARING IMPAIRMENT LEARNING EXPERIENCE FOR MEDICAL STUDENTS." BMJ Simulation and Technology Enhanced Learning 5: A45. | wrong population | |
| 1058 | Gosselin, J., et al. (2019). "Wireless measurement of rectal temperature during exercise: Comparing an ingestible thermometric telemetric pill used as a suppository against a conventional rectal probe." J Therm Biol 83: 112-118. | wrong intervention | |
| 1059 | Goude, G., et al. (2020). "Multi-proxy stable isotope analyses of dentine microsections reveal diachronic changes in life history adaptations, mobility, and tuberculosis-induced wasting in prehistoric Liguria (Finale Ligure, Italy, northwestern Mediterranean)." INTERNATIONAL JOURNAL OF PALEOPATHOLOGY 28: 99-111. | wrong design | |
| 1060 | Gouin, E., et al. (2022). "Pulmonary Effects of One Week of Repeated Recreational Closed-Circuit Rebreather Dives in Cold Water." Medicina (Kaunas) 59(1). | wrong design | |
| 1061 | Goumopoulos, C., et al. (2022). Participatory Design of Fall Prevention Exergames using Multiple Enabling Technologies. ICT4AWE: PROCEEDINGS OF THE 8TH INTERNATIONAL CONFERENCE ON INFORMATION AND COMMUNICATION TECHNOLOGIES FOR AGEING WELL AND E-HEALTH: 70-80. | wrong design | |
| 1062 | Gozt, A. K., et al. (2021). "Predicting outcome following mild traumatic brain injury: protocol for the longitudinal, prospective, observational Concussion Recovery (CREST) cohort study." BMJ Open 11(5): e046460. | wrong design | |
| 1063 | Grab, M., et al. (2023). "New perspectives in patient education for cardiac surgery using 3D-printing and virtual reality." FRONTIERS IN CARDIOVASCULAR MEDICINE 10. | wrong population | |
| 1064 | Grabowski, A., et al. (2021). "Teleoperated mobile robot with two arms: The influence of a human-machine interface, VR training and operator age." International Journal of Human-Computer Studies 156: 18. | wrong intervention | |
| 1065 | Grade, E. (2015). "Importance of Computerized Dynamic Posturography in Vestibular Rehabilitation...International Congress of Audiology, 9-10 October 2015, Portugal." Journal of Hearing Science 5(4): 56-57. | wrong design | |
| 1066 | Grady, B. (2023). "The Role of Information Technology in Enhancing Sport Psychology Interventions for Athlete Development and Performance Optimization"." REVISTA DE PSICOLOGIA DEL DEPORTE 32(4): 41-50. | wrong design | |
| 1067 | Gramlich, M. A. and S. M. Neer (2018). "Firefighter-Paramedic With Posttraumatic Stress Disorder, Horrific Images, and Depression: A Clinical Case Study." CLINICAL CASE STUDIES 17(3): 150-165. | wrong design | |
| 1068 | Grampurohit, N. and S. Benham (2019). "Immersive Virtual Reality for the Management of Pain in Community-Dwelling Older Adults." Archives of Physical Medicine and Rehabilitation 100(10): e84. | wrong design | |
| 1069 | Grange, L., et al. (2024). "Virtual reality for interventional radiology patients: a preliminary study." Supportive Care in Cancer 32(7). | wrong design | |
| 1070 | Grangeiro, M., et al. (2023). "Effect of Multiple Firings on the Microshear Bond Strength Between a Translucent Zirconia and a Resin Cement." Oper Dent 48(3): 329-336. | wrong design | |
| 1071 | Grashorn, W., et al. (2013). "Age-dependent decline of endogenous pain control: exploring the effect of expectation and depression." PLoS One 8(9): e75629. | wrong design | |
| 1072 | Grau, L. C., et al. (2021). "Survivorship, Clinical and Radiographic Outcomes of a Novel Cementless Metal-Backed Patella Design." JOURNAL OF ARTHROPLASTY 36(7): S221-S226. | wrong design | |
| 1073 | Greaves, D., et al. (2021). "4 Day in dry immersion reproduces partially the aging effect on the arteries as observed during 6 month spaceflight or confinement." NPJ Microgravity 7(1): 43. | wrong design | |
| 1074 | Greaves, N., et al. (2010). "Effects of a concurrent task on gaze behaviors during locomotor tasks." Parkinsonism and Related Disorders 16: S47-S48. | wrong design | |
| 1075 | Green, A. C., et al. (2012). "The delay between symptom onset and seeking professional treatment for anxiety and depressive disorders in a rural Australian sample." Social Psychiatry and Psychiatric Epidemiology: The International Journal for Research in Social and Genetic Epidemiology and Mental Health Services 47(9): 1475-1487. | wrong intervention | |
| 1076 | Green, A. C., et al. (2012). "The delay between symptom onset and seeking professional treatment for anxiety and depressive disorders in a rural Australian sample." Social Psychiatry & Psychiatric Epidemiology 47(9): 1475-1487. | wrong intervention | |
| 1077 | Greenan, E., et al. (2023). "Relationship between clinical parameters and quality of life in primary Sjogren's Syndrome: a prospective study." EYE 37(13): 2685-2692. | wrong intervention | |
| 1078 | Gremeau-Richard, C., et al. (2022). "Enhanced pain facilitation rather than impaired pain inhibition in burning mouth syndrome female patients." JOURNAL OF HEADACHE AND PAIN 23(1). | wrong intervention | |
| 1079 | Grenier, S., et al. (2015). "Using virtual reality to improve the efficacy of cognitive-behavioral therapy (CBT) in the treatment of late-life anxiety: preliminary recommendations for future research." Int Psychogeriatr 27(7): 1217-1225. | wrong design | |
| 1080 | Grenier, S., et al. (2015). "Using virtual reality to improve the efficacy of cognitive-behavioral therapy (CBT) in the treatment of late-life anxiety: preliminary recommendations for future research." INTERNATIONAL PSYCHOGERIATRICS 27(7): 1217-1225. | wrong design | |
| 1081 | Grenier, S., et al. (2015). "Using virtual reality to improve the efficacy of cognitive-behavioral therapy (CBT) in the treatment of late-life anxiety: Preliminary recommendations for future research." International Psychogeriatrics 27(7): 1217-1225. | wrong design | |
| 1082 | Grenier, S., et al. (2015). "Using virtual reality to improve the efficacy of cognitive-behavioral therapy (CBT) in the treatment of late-life anxiety: preliminary recommendations for future research." International Psychogeriatrics 27(7): 1217-1225. | wrong design | |
| 1083 | Griffiths, F. E., et al. (2018). Health Services and Delivery Research. The role of digital communication in patient–clinician communication for NHS providers of specialist clinical services for young people [the Long-term conditions Young people Networked Communication (LYNC) study]: a mixed-methods study. Southampton (UK), NIHR Journals Library | wrong design | |
| 1084 | Grits, D., et al. (2023). "Preoperative Veteran RAND-12 Mental Composite Score of >60 Associated With Increased Likelihood of Patient Satisfaction After Total Hip Arthroplasty." JOURNAL OF ARTHROPLASTY 38(7): S258-S264. | wrong design | |
| 1085 | Groes, S. (2017). "Information overload in literature." TEXTUAL PRACTICE 31(7): 1481-1508. | wrong design | |
| 1086 | Groninger, H., et al. (2024). "Virtual reality for pain management in hospitalized patients with cancer: A randomized controlled trial." CANCER 130(14): 2552-2560. | wrong population | |
| 1087 | Grosso, F., et al. (2024). "Imagining flying increases jumping performance in volleyball players: A pilot study." ACTA PSYCHOLOGICA 248. | wrong population | |
| 1088 | Growney, C. M. and T. English (2023). "Age and Cognitive Ability Predict Emotion Regulation Strategy Use." Journals of Gerontology Series B: Psychological Sciences & Social Sciences 78(6): 987-997. | wrong design | |
| 1089 | Grube, A., et al. (2017). "Eemian fossil caves and other karst structures in Cretaceous chalk and succeeding Quaternary sediments covering the salt structure Krempe-Lagerdorf (SW Schleswig-Holstein, North Germany)." ZEITSCHRIFT DER DEUTSCHEN GESELLSCHAFT FUR GEOWISSENSCHAFTEN 168(2): 263-284. | wrong design | |
| 1090 | Grube, A. and B. H. Rickert (2018). "Karstification on the Elmshorn salt diapir (SW Schleswig-Holstein, Germany)." ZEITSCHRIFT DER DEUTSCHEN GESELLSCHAFT FUR GEOWISSENSCHAFTEN 169(4): 547-566. | wrong design | |
| 1091 | Gruber, N., et al. (2024). "Virtual reality's impact on children with type 1 diabetes: a proof-of-concept randomized cross-over trial on anxiety, pain, adherence, and glycemic control." ACTA DIABETOLOGICA 61(2): 215-224. | wrong population | |
| 1092 | Grudzen, C. R., et al. (2010). "Palliative care needs of seriously ill, older adults presenting to the emergency department." Acad Emerg Med 17(11): 1253-1257. | wrong design | |
| 1093 | Grundy, J., et al. (2018). "Supporting Diverse Challenges of Ageing with Digital Enhanced Living Solutions." Stud Health Technol Inform 246: 75-90. | wrong design | |
| 1094 | Grundy, J., et al. (2018). "Supporting Diverse Challenges of Ageing with Digital Enhanced Living Solutions...5th Global Telehealth meeting, Adelaide, Australia, November 2017." Studies in Health Technology & Informatics 246: 75-90. | wrong design | |
| 1095 | Gualino, V., et al. (2020). "Patient experience of anti-vegf intravitreal injection." Journal francais d'ophtalmologie 43(10): 1047-1053. | wrong design | |
| 1096 | Guckert, M., et al. (2022). "The Disruption of Trust in the Digital Transformation Leading to Health 4.0." FRONTIERS IN DIGITAL HEALTH 4. | wrong design | |
| 1097 | Guede, I., et al. (2018). "Isotopic evidence for the reconstruction of diet and mobility during village formation in the Early Middle Ages: Las Gobas (Burgos, northern Spain)." ARCHAEOLOGICAL AND ANTHROPOLOGICAL SCIENCES 10(8): 2047-2058. | wrong intervention | |
| 1098 | Guenther, M., et al. (2022). "Virtual reality reduces pain in palliative care-A feasibility trial." BMC PALLIATIVE CARE 21(1). | wrong design | |
| 1099 | Guerrier, G., et al. (2021). "Improving Wellness of Operating Room Personnel: A Light-Based Intervention on Perceived Nursing-Related Stress." FRONTIERS IN PSYCHIATRY 12. | wrong intervention | |
| 1100 | Guevara-Aladino, P., et al. (2024). "Urban Care for Unpaid Caregivers: Community Voices in the Care Block Program, in Bogotá, Colombia." JOURNAL OF URBAN HEALTH-BULLETIN OF THE NEW YORK ACADEMY OF MEDICINE. | wrong design | |
| 1101 | Guglielmi, S., et al. (2021). "'I Just Keep Quiet': Addressing the Challenges of Married Rohingya Girls and Creating Opportunities for Change." EUROPEAN JOURNAL OF DEVELOPMENT RESEARCH 33(5): 1232-1251. | wrong design | |
| 1102 | Gui, W., et al. (2015). "Quality of Life in Patients with Noninfectious Uveitis Treated with or without Systemic Anti-inflammatory Therapy." OCULAR IMMUNOLOGY AND INFLAMMATION 23(2): 135-143. | wrong design | |
| 1103 | Guida, A., et al. (2020). "The development of working memory spatialization revealed by using the cave paradigm in a two-alternative spatial choice." ANNALS OF THE NEW YORK ACADEMY OF SCIENCES 1477(1): 54-70. | wrong design | |
| 1104 | Guillari, A., et al. (2024). "Non-pharmacological interventions to reduce procedural needle pain in children (6-12 years): A systematic review." JOURNAL OF PEDIATRIC NURSING-NURSING CARE OF CHILDREN & FAMILIES 78: e102-e116. | wrong population | |
| 1105 | Guinot, F., et al. (2021). "Comparison of active versus passive audiovisual distraction tools on children's behaviour, anxiety and pain in paediatric dentistry: a randomised crossover clinical trial." EUROPEAN JOURNAL OF PAEDIATRIC DENTISTRY 22(3): 230-236. | wrong intervention | |
| 1106 | Guldager, J. D., et al. (2023). "User experience, game satisfaction and engagement with the virtual simulation VR FestLab for alcohol prevention: A quantitative analysis among Danish adolescents." PLOS ONE 18(5). | wrong population | |
| 1107 | Gullo, G., et al. (2023). "Virtually Augmented Self-Hypnosis in Peripheral Vascular Intervention: A Randomized Controlled Trial." CardioVascular and Interventional Radiology 46(6): 786-793. | wrong outcomes | |
| 1108 | Gunnar, M. R., et al. (2021). "Validation of an online version of the Trier Social Stress Test in a study of adolescents." PSYCHONEUROENDOCRINOLOGY 125. | wrong population | |
| 1109 | Guo, S. C. and Y. J. Lan (2023). "Virtual world-supported contextualized multimodal EFL learning at a library." LANGUAGE LEARNING & TECHNOLOGY 27(2): 176-198. | wrong design | |
| 1110 | Guo, X., et al. (2023). "Psycho-physiological measures on a bicycle simulator in immersive virtual environments: how protected/curbside bike lanes may improve perceived safety." TRANSPORTATION RESEARCH PART F-TRAFFIC PSYCHOLOGY AND BEHAVIOUR 92: 317-336. | wrong design | |
| 1111 | Guo, Z. X., et al. (2023). Who's Watching Me?: Exploring the Impact of Audience Familiarity on Player Performance, Experience, and Exertion in Virtual Reality Exergames. 2023 IEEE INTERNATIONAL SYMPOSIUM ON MIXED AND AUGMENTED REALITY, ISMAR: 622-631. | wrong design | |
| 1112 | Gupta, A., et al. (2017). "Integrated Noninvasive Physiological Assessment of Coronary Circulatory Function and Impact on Cardiovascular Mortality in Patients With Stable Coronary Artery Disease." Circulation 136(24): 2325-2336. | wrong design | |
| 1113 | Gupta, A. and J. J. Thomas (2019). "Use of Virtual Reality as a Surrogate for Parental Presence During Anesthetic Induction: A Case Report." A & A PRACTICE 13(12): 454-456. | wrong design | |
| 1114 | Gurbuz, E. and A. A. Gurbuz (2024). "Investigation of the effect of virtual reality distraction in patients undergoing mandibular periodontal surgery: A randomized controlled study." JOURNAL OF ESTHETIC AND RESTORATIVE DENTISTRY 36(5): 813-822. | wrong population | |
| 1115 | Gutiérrez, L., et al. (2024). "Predictive value of invasive mechanical ventilation parameters for mortality in COVID-19 related ARDS: a retrospective cohort study." SCIENTIFIC REPORTS 14(1). | wrong design | |
| 1116 | Guzsvinecz, T., et al. (2020). "Analyzing the Spatial Skills of University Students with a Virtual Reality Application using a Desktop Display and the Gear VR." ACTA POLYTECHNICA HUNGARICA 17(2): 35-56. | wrong population | |
| 1117 | Guzsvinecz, T., et al. (2023). "The Effect of Engineering Education, on Spatial Ability, in Virtual Environments." ACTA POLYTECHNICA HUNGARICA 20(5): 11-30. | wrong design | |
| 1118 | Guzsvinecz, T., et al. (2022). An Overview of Received Results on MRT, MCT, and PSVT Spatial Ability Tests in Virtual Environments. 2022 IEEE 1st International Conference on Internet of Digital Reality, IoD 2022. | wrong design | |
| 1119 | Habas-Ulloa, A., et al. (2010). "Creep Behavior of High Density Polyethylene after Aging in Contact with Different Oil Derivates." POLYMER ENGINEERING AND SCIENCE 50(11): 2122-2130. | wrong population | |
| 1120 | Hadavi, S., et al. (2022). "VisualEars: How an immersive art exhibit impacts mood during the COVID-19 pandemic." FRONTIERS IN PSYCHOLOGY 13. | wrong population | |
| 1121 | Haddad, E. B. L., et al. (2024). "Enhancing mouthguard longevity: Impact of surface treatment against aging from brushing and disinfectant exposure." Dent Traumatol 40(4): 453-459. | wrong population | |
| 1122 | Hadjipanayi, C. and D. Michael-Grigoriou (2022). "Arousing a wide range of emotions within educational virtual reality simulation about major depressive disorder affects knowledge retention." VIRTUAL REALITY 26(1): 343-359. | wrong population | |
| 1123 | Hadley, W., et al. (2019). "Moving Beyond Role-Play: Evaluating the Use of Virtual Reality to Teach Emotion Regulation for the Prevention of Adolescent Risk Behavior Within a Randomized Pilot Trial." JOURNAL OF PEDIATRIC PSYCHOLOGY 44(4): 425-435. | wrong population | |
| 1124 | Hafskjold, L., et al. (2016). "Older persons’ worries expressed during home care visits: Exploring the content of cues and concerns identified by the Verona coding definitions of emotional sequences." Patient Education and Counseling 99(12): 1955-1963. | wrong design | |
| 1125 | Hafskjold, L., et al. (2017). "The use of supportive communication when responding to older people's emotional distress in home care - An observational study." BMC NURSING 16. | wrong design | |
| 1126 | Hafskjold, L., et al. (2017). "The use of supportive communication when responding to older people's emotional distress in home care -- An observational study." BMC Nursing 16: 1-12. | wrong design | |
| 1127 | Hagan, D. P., et al. (2023). "Outcomes of Biceps Tenodesis Variations With Concomitant Rotator Cuff Repair: A Multicenter Database Analysis." ORTHOPAEDIC JOURNAL OF SPORTS MEDICINE 11(6). | wrong design | |
| 1128 | Hajahmadi, S. and G. Marfia (2023). "Effects of the Uncertainty of Interpersonal Communications on Behavioral Responses of the Participants in an Immersive Virtual Reality Experience: A Usability Study." SENSORS 23(4). | wrong design | |
| 1129 | Halenar, L. B., et al. (2017). "New cranium of the endemic Caribbean platyrrhine, <i>Antillothrix bernensis</i>, from La Altagracia Province, Dominican Republic." JOURNAL OF HUMAN EVOLUTION 106: 133-153. | wrong design | |
| 1130 | Hales, H., et al. (2019). "What next for adolescent forensic mental health research?" CRIMINAL BEHAVIOUR AND MENTAL HEALTH 29(4): 196-206. | wrong population | |
| 1131 | Halfon, E. and R. Barkai (2020). "The material and mental effects of animal disappearance on indigenous hunter-gatherers, past and present." TIME & MIND-THE JOURNAL OF ARCHAEOLOGY CONSCIOUSNESS AND CULTURE 13(1): 5-33. | wrong design | |
| 1132 | Hali, K., et al. (2024). "Use of virtual reality for the management of phantom limb pain: A systematic review." Disability and Rehabilitation: An International, Multidisciplinary Journal 46(4): 629-636. | wrong design | |
| 1133 | Hall, D. S., et al. (2014). "Applications of in Situ Raman Spectroscopy for Identifying Nickel Hydroxide Materials and Surface Layers during Chemical Aging." ACS APPLIED MATERIALS & INTERFACES 6(5): 3141-3149. | wrong population | |
| 1134 | Hall, L., et al. (2011). FOSTERING EMPATHIC BEHAVIOUR IN CHILDREN AND YOUNG PEOPLE: INTERACTION WITH INTELLIGENT CHARACTERS EMBODYING CULTURALLY SPECIFIC BEHAVIOUR IN VIRTUAL WORLD SIMULATIONS. INTED2011: 5TH INTERNATIONAL TECHNOLOGY, EDUCATION AND DEVELOPMENT CONFERENCE: 2804-2814. | wrong design | |
| 1135 | Hall, M. E., et al. (2000). Hormones of the Cardiovascular System. Endotext. K. R. Feingold, B. Anawalt, M. R. Blackman et al. South Dartmouth (MA), MDText.com, Inc. | wrong population | |
| 1136 | Halldorsson, B., et al. (2021). "Immersive virtual reality and digital applied gaming interventions for the treatment of mental health problems in children and young people: the need for rigorous treatment development and clinical evaluation." JOURNAL OF CHILD PSYCHOLOGY AND PSYCHIATRY 62(5): 584-605. | wrong population | |
| 1137 | Hamdani, S. U., et al. (2021). "Scaling-up school mental health services in low resource public schools of rural Pakistan: the Theory of Change (ToC) approach." INTERNATIONAL JOURNAL OF MENTAL HEALTH SYSTEMS 15(1). | wrong design | |
| 1138 | Hamdy, S. F., et al. (2024). "Enhancing Pediatric Dental Care: The Influence of Virtual Reality." EUROPEAN JOURNAL OF DENTISTRY. | wrong population | |
| 1139 | Hamid, S., et al. (2023). "Emerging Technology for Healthy Lifestyle of the Middle-Age and Elderly: A Scoping Review." Iran J Public Health 52(2): 230-242. | wrong design | |
| 1140 | Hamid, S., et al. (2023). "Emerging Technology for Healthy Lifestyle of the Middle-Age and Elderly: A Scoping Review." IRANIAN JOURNAL OF PUBLIC HEALTH 52(2): 230-242. | wrong design | |
| 1141 | Hamilton-Giachritsis, C., et al. (2018). "Reducing risk and improving maternal perspective-taking and empathy using virtual embodiment." SCIENTIFIC REPORTS 8. | wrong design | |
| 1142 | Hammond, A., et al. (2017). "Job retention vocational rehabilitation for employed people with inflammatory arthritis (WORK-IA): a feasibility randomized controlled trial." BMC Musculoskelet Disord 18(1): 315. | wrong population | |
| 1143 | Hammond, A., et al. (2023). "The Work Experience Survey - Rheumatic conditions (United Kingdom): Psychometric properties and identifying the workplace barriers of employed people with inflammatory arthritis receiving vocational rehabilitation." MUSCULOSKELETAL CARE 21(4): 1578-1591. | wrong design | |
| 1144 | Hammond, F. M., et al. (2019). "Prevalence of Medical and Psychiatric Comorbidities Following Traumatic Brain Injury." J Head Trauma Rehabil 34(4): E1-e10. | wrong design | |
| 1145 | Han, K. J., et al. (2021). "Mobile Augmented Reality Serious Game for Improving Old Adults' Working Memory." APPLIED SCIENCES-BASEL 11(17). | wrong intervention | |
| 1146 | Han, S. H., et al. (2019). "Effect of Immersive Virtual Reality Education Before Chest Radiography on Anxiety and Distress Among Pediatric Patients A Randomized Clinical Trial." JAMA PEDIATRICS 173(11): 1026-1031. | wrong population | |
| 1147 | Han, S. W., et al. (2017). "Mechanical property behavior and aging mechanism of carbon-black-filled EPDM rubber reinforced by carbon nano-tubes subjected to electro-chemical and thermal degradation." JOURNAL OF MECHANICAL SCIENCE AND TECHNOLOGY 31(9): 4073-4078. | wrong intervention | |
| 1148 | Haney, L. J., et al. (2021). "Persistent Pain, Physical Dysfunction, and Decreased Quality of Life After Combat Extremity Vascular Trauma." ANNALS OF VASCULAR SURGERY 71: 167-180. | wrong intervention | |
| 1149 | Hanifah, H., et al. (2022). "Promoting Sports Engagement during the COVID-19 Pandemic via Virtual Reality Games." OCCUPATIONAL THERAPY INTERNATIONAL 2022. | wrong population | |
| 1150 | Hankala, M., et al. (2017). Towards a scenario of virtual mental health environments for school-aged children. PROCEEDINGS OF THE 21ST INTERNATIONAL ACADEMIC MINDTREK CONFERENCE (ACADEMIC MINDTREK): 239-242. | wrong population | |
| 1151 | Hanke, T., et al. (2013). "Haemodynamic performance of a new pericardial aortic bioprosthesis during exercise and recovery: comparison with pulmonary autograft, stentless aortic bioprosthesis and healthy control groups." EUROPEAN JOURNAL OF CARDIO-THORACIC SURGERY 44(4): E295-E301. | wrong population | |
| 1152 | Hannoush, Z. C. and R. E. Weiss (2000). Hypopituitarism: Emergencies. Endotext. K. R. Feingold, B. Anawalt, M. R. Blackman et al. South Dartmouth (MA), MDText.com, Inc. | wrong intervention | |
| 1153 | Hao, J., et al. (2018). "Comparative Study of DC Breakdown and Space Charge Characteristics of Insulation Paper Impregnated with Natural Ester and Mineral Oil." JOURNAL OF ELECTRICAL ENGINEERING & TECHNOLOGY 13(4): 1681-1690. | wrong intervention | |
| 1154 | Hardisty, M. R., et al. (2013). "Stress-whitening occurs in demineralized bone." BONE 57(2): 367-374. | wrong intervention | |
| 1155 | Hardisty, M. R., et al. (2014). "Strain-induced optical changes in demineralized bone." JOURNAL OF BIOMEDICAL OPTICS 19(3). | wrong intervention | |
| 1156 | Hardisty, M. R., et al. (2013). "Do stress-whitening and optical clearing of collagenous tissue occur by the same mechanism?" JOURNAL OF BIOMECHANICS 46(14): 2411-2418. | wrong intervention | |
| 1157 | Hariri, I., et al. (2012). "The effects of aging on shear bond strength and nanoleakage expression of an etch-and-rinse adhesive on human enamel and dentin." J Adhes Dent 14(3): 235-243. | wrong intervention | |
| 1158 | Harris, D. M., et al. (2015). "Exergaming as a Viable Therapeutic Tool to Improve Static and Dynamic Balance among Older Adults and People with Idiopathic Parkinson's Disease: A Systematic Review and Meta-Analysis." Front Aging Neurosci 7: 167. | wrong intervention | |
| 1159 | Harris, Z. D. and J. T. Burns (2019). "The effect of isothermal heat treatment on hydrogen environment-assisted cracking susceptibility in Monel K-500." MATERIALS SCIENCE AND ENGINEERING A-STRUCTURAL MATERIALS PROPERTIES MICROSTRUCTURE AND PROCESSING 764. | wrong intervention | |
| 1160 | Harris, Z. D., et al. (2016). "The Effect of Microstructural Variation on the Hydrogen Environment-Assisted Cracking of Monel K-500." METALLURGICAL AND MATERIALS TRANSACTIONS A-PHYSICAL METALLURGY AND MATERIALS SCIENCE 47A(7): 3488-3510. | wrong intervention | |
| 1161 | Harrow, S. (2024). "Thinking Madame Raquin: Consciousness and Cognition in Emile Zola's <i>Therese </i>Raquin (1867)." ESPRIT CREATEUR 64(2). | wrong intervention | |
| 1162 | Hartanto, A., et al. (2023). "Does a 15-Minute Brief Mindfulness Breathing Exercise Temporarily Enhance Inhibitory Control and Cognitive Flexibility? A Within-Subject Experimental Approach." COLLABRA-PSYCHOLOGY 9(1). | wrong intervention | |
| 1163 | Hartke, R. J. and R. Trierweiler (2015). "Survey of survivors' perspective on return to work after stroke." TOPICS IN STROKE REHABILITATION 22(5): 326-334. | wrong intervention | |
| 1164 | Harvey, P. D., et al. (2020). "Factor structure of cognitive performance and functional capacity in schizophrenia: Evidence for differences across functional capacity measures." Schizophr Res 223: 297-304. | wrong design | |
| 1165 | Harvey, P. D., et al. (2017). "Using the Positive and Negative Syndrome Scale (PANSS) to Define Different Domains of Negative Symptoms: Prediction of Everyday Functioning by Impairments in Emotional Expression and Emotional Experience." Innov Clin Neurosci 14(11-12): 18-22. | wrong design | |
| 1166 | Harvey, P. D. and R. S.e. Keefe (2012). "Technology, Society, and Mental Illness: Challenges and Opportunities for Assessment and Treatment." Innovations in Clinical Neuroscience 9(11/12): 47-50. | wrong design | |
| 1167 | Harvey, P. D., et al. (2023). "Mild Cognitive Impairment, But Not HIV Status, is Related to Reduced Awareness of Level of Cognitive Performance Among Older Adults." American Journal of Geriatric Psychiatry 31(12): 1117-1128. | wrong intervention | |
| 1168 | Hasei, J., et al. (2024). "Utilizing the Metaverse to Provide Innovative Psychosocial Support for Pediatric, Adolescent, and Young Adult Patients with Rare Cancer." CANCERS 16(15). | wrong population | |
| 1169 | Hashem, S. and I. V. Vahia (2024). "Digital Mental Health for Older Adults: Foe or Friend?" GENERATIONS 48(1): 6-11. | wrong intervention | |
| 1170 | Hashemzadeh Segherloo, I., et al. (2018). "Genetic and morphological support for possible sympatric origin of fish from subterranean habitats." Scientific reports 8(1): 2909. | wrong intervention | |
| 1171 | Hashiguchi, N., et al. (2021). "Psychological Effects of Heart Rate and Physical Vibration on the Operation of Construction Machines: Experimental Study." JMIR MHEALTH AND UHEALTH 9(9). | wrong intervention | |
| 1172 | Hashim, K., et al. (2015). SOCIAL MEDIA IMPACT ON STUDENTS' SOCIAL BEHAVIOR: A FACULTY PERCEPTION COMPARISON BETWEEN GENDERS. ICERI2015: 8TH INTERNATIONAL CONFERENCE OF EDUCATION, RESEARCH AND INNOVATION: 3618-3627. | wrong population | |
| 1173 | Hasselmann, V., et al. (2015). "Are exergames promoting mobility an attractive alternative to conventional self-regulated exercises for elderly people in a rehabilitation setting? Study protocol of a randomized controlled trial." BMC GERIATRICS 15. | wrong intervention | |
| 1174 | Hawes, D. and A. Arya (2023). "Technology Solutions to Reduce Anxiety and Increase Cognitive Availability in Students." IEEE TRANSACTIONS ON LEARNING TECHNOLOGIES 16(2): 278-291. | wrong intervention | |
| 1175 | Hawila, B., et al. (2023). "Association between Covid-19 vaccination, fear of COVID-19 and female sexual satisfaction among a sample of Lebanese women: A pilot study." PHARMACY PRACTICE-GRANADA 21(2). | wrong intervention | |
| 1176 | Hawkins, K., et al. (2012). "The prevalence of hearing impairment and its burden on the quality of life among adults with Medicare Supplement Insurance." Quality of Life Research: An International Journal of Quality of Life Aspects of Treatment, Care & Rehabilitation 21(7): 1135-1147. | wrong intervention | |
| 1177 | Hazem, A., et al. (2024). "Orthodontic System Modeled and Simulated with the Lingual Technique to Assess Tooth Forces." DIAGNOSTICS 14(11). | wrong intervention | |
| 1178 | He, E. M., et al. (2024). "Awe Inducing Elements in Virtual Reality Applications: A Prospective Study of Hospitalized Children and Caregivers." GAMES FOR HEALTH JOURNAL. | wrong population | |
| 1179 | He, J., et al. (2024). "Integration of tai chi and repetitive transcranial magnetic stimulation for sleep disturbances in older adults: A pilot randomized controlled trial." Sleep Medicine 122: 35-44. | wrong intervention | |
| 1180 | He, J., et al. (2023). "Intercultural sensitivity as a mediator in the relationship between implicit intercultural identification and emotional disturbance-An exploratory study of international high school students." Front Psychiatry 14: 1098671. | wrong intervention | |
| 1181 | He, J. Y., et al. (2023). "Intercultural sensitivity as a mediator in the relationship between implicit intercultural identification and emotional disturbance-An exploratory study of international high school students." FRONTIERS IN PSYCHIATRY 14. | wrong intervention | |
| 1182 | He, S. Y., et al. (2015). DESIGN OF INFORMATION COLLECTING SYSTEM TOWARDS THE SONG DYNASTY WOODEN HALL OF BAOGUO TEMPLE, NINGBO. 25TH INTERNATIONAL CIPA SYMPOSIUM 2015. 40-5: 513-518. | wrong intervention | |
| 1183 | He, X., et al. (2012). "The Relationship Between Concave Angle of Vertebral Endplate and Lumbar Intervertebral Disc Degeneration." SPINE 37(17): E1068-E1073. | wrong intervention | |
| 1184 | He, X. Y. and X. L. Yang (2022). "Effects of exercise on flow characteristics in human carotids." PHYSICS OF FLUIDS 34(1). | wrong intervention | |
| 1185 | He, Z. Q., et al. (2019). Research on law of front abutment pressure change of top-coal caving. DEEP ROCK MECHANICS: FROM RESEARCH TO ENGINEERING: 343-351. | wrong intervention | |
| 1186 | Head, D. and M. Isom (2010). "Age effects on wayfinding and route learning skills." Behavioural Brain Research 209(1): 49-58. | wrong intervention | |
| 1187 | Healey, A., et al. (2017). "An experimental test of the role of control in spider fear." JOURNAL OF ANXIETY DISORDERS 49: 12-20. | wrong intervention | |
| 1188 | Healy, D., et al. (2021). "Older Adults' Experiences and Perceptions of Immersive Virtual Reality: A Protocol for a Systematic Review and Thematic Synthesis." INTERNATIONAL JOURNAL OF QUALITATIVE METHODS 20. | wrong design | |
| 1189 | Healy, D., et al. (2022). "Older Adults’ Experiences and Perceptions of Immersive Virtual Reality: Systematic Review and Thematic Synthesis." JMIR Serious Games 10(4). | wrong design | |
| 1190 | Healy, P., et al. (2023). "An Exposure-Based Video Game (Dr. Zoo) to Reduce Needle Phobia in Children Aged 3 to 6 Years: Development and Mixed Methods Pilot Study." JMIR SERIOUS GAMES 11. | wrong population | |
| 1191 | Heck, M., et al. (2024). "Impact of a pre-test measurement of alcohol craving in cue-exposure studies: Relationship with social desirability and demand effects." ALCOHOL 115: 41-52. | wrong intervention | |
| 1192 | Hedna, K., et al. (2018). "Antidepressants and suicidal behaviour in late life: a prospective population-based study of use patterns in new users aged 75 and above." Eur J Clin Pharmacol 74(2): 201-208. | wrong intervention | |
| 1193 | Heena, A., et al. (2024). "Comparison of different behaviour management techniques while administering injectable la with minimum pain perception and anxiety in children." J Clin Exp Dent 16(8): e1021-e1026. | wrong population | |
| 1194 | Heilman, B. M., et al. (2024). "Effect of compound treatments on mouse lens viscoelasticity." EXPERIMENTAL EYE RESEARCH 246. | wrong intervention | |
| 1195 | Helminen, E. C., et al. (2019). "A meta-analysis of cortisol reactivity to the Trier Social Stress Test in virtual environments." PSYCHONEUROENDOCRINOLOGY 110. | wrong intervention | |
| 1196 | Helminen, E. C., et al. (2021). "Stress Reactivity to the Trier Social Stress Test in Traditional and Virtual Environments: A Meta-Analytic Comparison." PSYCHOSOMATIC MEDICINE 83(3): 200-211. | wrong intervention | |
| 1197 | Hendricks, T. M., et al. (2020). "The Use of Virtual Reality to Reduce Preoperative Anxiety in First-Time Sternotomy Patients: A Randomized Controlled Pilot Trial." MAYO CLINIC PROCEEDINGS 95(6): 1148-1157. | wrong population | |
| 1198 | Hensel, J. M., et al. (2016). "A pragmatic randomized control trial and realist evaluation on the implementation and effectiveness of an internet application to support self-management among individuals seeking specialized mental health care: A study protocol." BMC Psychiatry 16: 11. | wrong intervention | |
| 1199 | Henssen, D., et al. (2020). "Neuroanatomy Learning: Augmented Reality vs. Cross-Sections." ANATOMICAL SCIENCES EDUCATION 13(3): 350-362. | wrong intervention | |
| 1200 | Heo, J., et al. (2015). "Internet use and well-being in older adults." Cyberpsychology, Behavior, and Social Networking 18(5): 268-272. | wrong intervention | |
| 1201 | Hepburn, K., et al. (2016). "Initiation of drug dealing among a prospective cohort of street-involved youth." AMERICAN JOURNAL OF DRUG AND ALCOHOL ABUSE 42(5): 507-512. | wrong intervention | |
| 1202 | Hermans, A. N. L., et al. (2023). "360° Virtual reality to improve patient education and reduce anxiety towards atrial fibrillation ablation." EUROPACE 25(3): 855-862. | wrong intervention | |
| 1203 | Hernandez, R., et al. (2021). "Mindfulness-based Virtual Reality Intervention in Hemodialysis Patients: A Pilot Study on End-user Perceptions and Safety." KIDNEY360 2(3): 435-444. | wrong design | |
| 1204 | Hernandez, R., et al. (2023). "Positive Psychological Intervention Delivered Using Virtual Reality in Patients on Hemodialysis With Comorbid Depression: Protocol and Design for the Joviality Randomized Controlled Trial." JMIR RESEARCH PROTOCOLS 12. | wrong design | |
| 1205 | Hernández-Serrano, O., et al. (2020). "Predictors of Changes in Alcohol Craving Levels during a Virtual Reality Cue Exposure Treatment among Patients with Alcohol Use Disorder." JOURNAL OF CLINICAL MEDICINE 9(9). | wrong design | |
| 1206 | Herrera, M. D., et al. (2022). "Use of Virtual Reality in the Reduction of Pain After the Administration of Vaccines Among Children in Primary Care Centers: Protocol for a Randomized Clinical Trial." JMIR RESEARCH PROTOCOLS 11(4). | wrong design | |
| 1207 | Herscu, O., et al. (2023). "Mindfulness Meditation and Self-Monitoring Reduced Maladaptive Daydreaming Symptoms: A Randomized Controlled Trial of a Brief Self-Guided Web-Based Program." JOURNAL OF CONSULTING AND CLINICAL PSYCHOLOGY 91(5): 285-300. | wrong intervention | |
| 1208 | Hesko, C., et al. (2019). "Use of virtual reality (VR) during mediport accessing in pediatric hematology oncology patients." Pediatric Blood and Cancer 66: S187. | wrong design | |
| 1209 | Hess, H. W., et al. (2021). "Carotid body chemosensitivity is not attenuated during cold water diving." AMERICAN JOURNAL OF PHYSIOLOGY-REGULATORY INTEGRATIVE AND COMPARATIVE PHYSIOLOGY 321(2): R197-R207. | wrong intervention | |
| 1210 | Heyn, L. G., et al. (2021). "Older persons’ expressed worries during nursing care at home: Do health complexity and nature of nursing care in the visit matter?" Patient Education and Counseling 104(10): 2418-2424. | wrong intervention | |
| 1211 | Higaki, A., et al. (2023). "Automated categorization of virtual reality studies in cardiology based on the device usage: a bibliometric analysis (2010-2022)." Eur Heart J Digit Health 4(2): 119-124. | wrong population | |
| 1212 | Hill, A., et al. (2022). "Predicting program outcomes for vocational rehabilitation customers: A machine learning approach." JOURNAL OF VOCATIONAL REHABILITATION 56(2): 107-121. | wrong intervention | |
| 1213 | Hill, N. T., et al. (2017). "Computerized Cognitive Training in Older Adults With Mild Cognitive Impairment or Dementia: A Systematic Review and Meta-Analysis." Am J Psychiatry 174(4): 329-340. | wrong intervention | |
| 1214 | Hill, N. T. M., et al. (2017). "Computerized cognitive training in older adults with mild cognitive impairment or dementia: A systematic review and meta-analysis." The American Journal of Psychiatry 174(4): 329-340. | wrong intervention | |
| 1215 | Hill, P. P., et al. (2024). "Remembering to Resume: A Randomized Trial Comparing Combined Interruption Management Training and Simulation-Based Education to Simulation-Based Education Alone." Nurs Educ Perspect 45(1): 5-11. | wrong intervention | |
| 1216 | Hines, A. C., et al. (2022). "Mental health status, not resilience, influences functional recovery after arthroscopic rotator cuff repairs." JOURNAL OF SHOULDER AND ELBOW SURGERY 31(6): S117-S122. | wrong intervention | |
| 1217 | Hitzerd, E., et al. (2020). "Larger First-Trimester Placental Volumetric Parameters Are Associated With Lower Pressure and More Flow-Mediated Vasodilation of the Fetoplacental Vasculature After Delivery." FRONTIERS IN PHYSIOLOGY 11. | wrong intervention | |
| 1218 | Hoag, J. A., et al. (2022). "Distracting Through Procedural Pain and Distress Using Virtual Reality and Guided Imagery in Pediatric, Adolescent, and Young Adult Patients: Randomized Controlled Trial." JOURNAL OF MEDICAL INTERNET RESEARCH 24(4). | wrong population | |
| 1219 | Hodgskiss, T. (2014). "Cognitive Requirements for Ochre Use in the Middle Stone Age at Sibudu, South Africa." CAMBRIDGE ARCHAEOLOGICAL JOURNAL 24(3): 405-428. | wrong intervention | |
| 1220 | Hodgson, D. D., et al. (2023). "Visual feedback-dependent modulation of arousal, postural control, and muscle stretch reflexes assessed in real and virtual environments." FRONTIERS IN HUMAN NEUROSCIENCE 17. | wrong intervention | |
| 1221 | Hoeffding, L. K., et al. (2017). "A manual-based vocational rehabilitation program for patients with an acquired brain injury: study protocol of a pragmatic randomized controlled trial (RCT)." TRIALS 18. | wrong intervention | |
| 1222 | Hoeg, E. R., et al. (2023). "Hospitalized older adults' experiences of virtual reality-based group exercise therapy with cycle ergometers: An early feasibility study." COMPUTERS IN HUMAN BEHAVIOR REPORTS 11. | wrong intervention | |
| 1223 | Hoeg, E. R., et al. (2023). "Buddy biking: a user study on social collaboration in a virtual reality exergame for rehabilitation." VIRTUAL REALITY 27(1): 245-262. | wrong outcomes | |
| 1224 | Hoeksema, L. and L. I. Los (2014). "Vision-Related Quality of Life in Herpetic Anterior Uveitis Patients." PLOS ONE 9(1). | wrong intervention | |
| 1225 | Hoekstra, M., et al. (2023). "3MDR treatment in an adolescent with PTSD: a case report." EUROPEAN JOURNAL OF PSYCHOTRAUMATOLOGY 14(2). | wrong population | |
| 1226 | Hoenig, H. M., et al. (2018). "Testing fine motor coordination via telehealth: Effects of video characteristics on reliability and validity." Journal of Telemedicine and Telecare 24(5): 365-372. | wrong intervention | |
| 1227 | Hoffman, H. G., et al. (2014). "Feasibility of Articulated Arm Mounted Oculus Rift Virtual Reality Goggles for Adjunctive Pain Control During Occupational Therapy in Pediatric Burn Patients." CYBERPSYCHOLOGY BEHAVIOR AND SOCIAL NETWORKING 17(6): 397-401. | wrong population | |
| 1228 | Hoffman, H. G., et al. (2019). "Immersive Virtual Reality as an Adjunctive Non-opioid Analgesic for Pre-dominantly Latin American Children With Large Severe Burn Wounds During Burn Wound Cleaning in the Intensive Care Unit: A Pilot Study." FRONTIERS IN HUMAN NEUROSCIENCE 13. | wrong population | |
| 1229 | Hoffman, H. G., et al. (2024). "Increasing presence via a more immersive VR system increases virtual reality analgesia and draws more attention into virtual reality in a randomized crossover study." FRONTIERS IN VIRTUAL REALITY 5. | wrong population | |
| 1230 | Höglander, J., et al. (2017). "Registered nurses’ and nurse assistants’ responses to older persons’ expressions of emotional needs in home care." Journal of Advanced Nursing 73(12): 2923-2932. | wrong population | |
| 1231 | Höglander, J., et al. (2019). "Emotional communication with older people: A cross-sectional study of home care." NURSING & HEALTH SCIENCES 21(3): 382-389. | wrong intervention | |
| 1232 | Holdnack, J. A. and P. F. Brennan (2021). "Usability and Effectiveness of Immersive Virtual Grocery Shopping for Assessing Cognitive Fatigue in Healthy Controls: Protocol for a Randomized Controlled Trial." JMIR RESEARCH PROTOCOLS 10(8). | wrong design | |
| 1233 | Holinger, M., et al. (2015). "Group composition for fattening entire male pigs under enriched housing conditions-Influences on behaviour, injuries and boar taint compounds." APPLIED ANIMAL BEHAVIOUR SCIENCE 165: 47-56. | wrong intervention | |
| 1234 | Holloway, H., et al. (2024). "Immersive virtual reality in the promotion of health and well-being for people in residential aged care without cognitive impairment: A scoping review." DIGITAL HEALTH 10. | wrong intervention | |
| 1235 | Holmberg, T. T., et al. (2020). "Social Anxiety Can Be Triggered by 360-Degree Videos in Virtual Reality: A Pilot Study Exploring Fear of Shopping." CYBERPSYCHOLOGY BEHAVIOR AND SOCIAL NETWORKING 23(7): 495-499. | wrong intervention | |
| 1236 | Holt, D. E., et al. (2021). "Improving the Pediatric Patient Experience During Radiation Therapy-A Children's Oncology Group Study." International Journal of Radiation Oncology, Biology, Physics 109(2): 505-514. | wrong intervention | |
| 1237 | Holt, D. E., et al. (2021). "Improving the Pediatric Patient Experience During Radiation Therapy-A Children's Oncology Group Study." INTERNATIONAL JOURNAL OF RADIATION ONCOLOGY BIOLOGY PHYSICS 109(2): 505-514. | wrong population | |
| 1238 | Homagain, A. and K. A. Ehgoetz Martens (2023). Emotional states affect walking performance. | wrong population | |
| 1239 | Homagain, A. and K. Ehgoetz-Martens (2023). "EMOTIONS AFFECT WALKING PERFORMANCE." IBRO Neuroscience Reports 15: S848-S849. | wrong intervention | |
| 1240 | Homel, J., et al. (2020). "Changes in positive and negative peer influences and depressive symptoms from adolescence to young adulthood." J Adolesc 84: 113-122. | wrong population | |
| 1241 | Honeycutt, T. C., et al. (2017). "Public Provision of Postsecondary Education for Transition-Age Youth With Mental Health Conditions." PSYCHIATRIC REHABILITATION JOURNAL 40(2): 183-196. | wrong population | |
| 1242 | Hong, F. F., et al. (2023). "Evaluating a technologically enhanced rehabilitation programme for wound healing in patients with coronary heart disease." INTERNATIONAL WOUND JOURNAL. | wrong intervention | |
| 1243 | Hong, J. C., et al. (2018). Effects of Gender and Age on Learning Spatial Concepts from a Virtual Reality Game. PROCEEDINGS OF 2018 IEEE INTERNATIONAL CONFERENCE ON TEACHING, ASSESSMENT, AND LEARNING FOR ENGINEERING (TALE): 1206-1207. | wrong outcomes | |
| 1244 | Hong, J. C., et al. (2024). "The entity belief of concentration ability predicts cognitive load, failure-attribution, and flow experience when using a virtual reality device." INTERACTIVE LEARNING ENVIRONMENTS 32(1): 34-51. | wrong intervention | |
| 1245 | Hong, J.-C., et al. (2022). "The entity belief of concentration ability predicts cognitive load, failure-attribution, and flow experience when using a virtual reality device." Interactive Learning Environments. | wrong intervention | |
| 1246 | Hong, Y., et al. (2024). "Characterizing the supraspinal sensorimotor control of walking using MRI-compatible system: a systematic review." J Neuroeng Rehabil 21(1): 34. | wrong intervention | |
| 1247 | Hong, Y. L., et al. (2024). "Characterizing the supraspinal sensorimotor control of walking using MRI-compatible system: a systematic review." JOURNAL OF NEUROENGINEERING AND REHABILITATION 21(1). | wrong intervention | |
| 1248 | Hook, J. (2024). "Virtual Reality + Heart Rate Variability Biofeedback Pain Effects in Older Adults with Knee Osteoarthritis...American Society for Pain Management Nursing (ASPMN) 33rd National Conference, September 20-23, 2023, Minneapolis, Minnesota." Pain Management Nursing 25(2): e171-e171. | wrong outcomes | |
| 1249 | Hooper, P. L., et al. (2014). "The importance of the cellular stress response in the pathogenesis and treatment of type 2 diabetes." Cell Stress Chaperones 19(4): 447-464. | wrong intervention | |
| 1250 | Hoppes, C. W., et al. (2022). "Treatment of Mal de Debarquement Syndrome in a Computer-Assisted Rehabilitation Environment." MILITARY MEDICINE 187(7-8): E1011-E1015. | wrong intervention | |
| 1251 | Horesh, D., et al. (2022). "Virtual Reality Combined with Artificial Intelligence (VR-AI) Reduces Hot Flashes and Improves Psychological Well-Being in Women with Breast and Ovarian Cancer: A Pilot Study." HEALTHCARE 10(11). | wrong population | |
| 1252 | Hornsby, N., et al. (2020). "Psychosocial Interventions Targeting Recovery in Child and Adolescent Burns: A Systematic Review." JOURNAL OF PEDIATRIC PSYCHOLOGY 45(1): 15-33. | wrong intervention | |
| 1253 | Hosick, P. A., et al. (2010). "Relationship between change in core temperature and change in cortisol and TNFα during exercise." JOURNAL OF THERMAL BIOLOGY 35(7): 348-353. | wrong intervention | |
| 1254 | Hosseini, M., et al. (2023). "Assessing virtual reality acceptance in long-term care facilities: a quantitative study with older adults." DISABILITY AND REHABILITATION-ASSISTIVE TECHNOLOGY. | wrong design | |
| 1255 | Hosseini, M., et al. (2024). "Acceptance of physical activity virtual reality games by residents of long-term care facilities: A qualitative study." PLoS ONE 19(6): 18. | wrong design | |
| 1256 | Hosseini, M., et al. (2024). "Assessing virtual reality acceptance in long-term care facilities: a quantitative study with older adults." Disabil Rehabil Assist Technol 19(7): 2602-2614. | wrong design | |
| 1257 | Hosseini, S. J., et al. (2023). "Age group as a predictor of the effect of virtual reality on pain management in burn patients." BURNS 49(3). | wrong design | |
| 1258 | Hoteit, M., et al. (2022). "Prevalence, correlates, and gender disparities related to eating disordered behaviors among health science students and healthcare practitioners in Lebanon: Findings of a national cross sectional study." FRONTIERS IN NUTRITION 9. | wrong intervention | |
| 1259 | Hou, K. M., et al. (2024). "Influence of functional boundary for corridor spaces in elderly facilities in China." BUILDING RESEARCH AND INFORMATION 52(6): 658-679. | wrong intervention | |
| 1260 | Houkamau, C., et al. (2021). "Cyberbullying toward Māori is rife in New Zealand: Incidences and demographic differences in experiences of cyberbullying among Māori." Cyberpsychology, Behavior, and Social Networking 24(12): 822-830. | wrong intervention | |
| 1261 | House, G., et al. (2015). BrightArm Duo integrative rehabilitation for post-stroke maintenance in Skilled Nursing Facilities. 2015 INTERNATIONAL CONFERENCE ON VIRTUAL REHABILITATION PROCEEDINGS (ICVR): 207-214. | wrong intervention | |
| 1262 | House, G., et al. (2016). "Integrative rehabilitation of residents chronic post-stroke in skilled nursing facilities: the design and evaluation of the BrightArm Duo." Disabil Rehabil Assist Technol 11(8): 683-694. | wrong intervention | |
| 1263 | House, G., et al. (2016). "A serious gaming alternative to pen-and-paper cognitive scoring: A pilot study of BrightScreener™." Journal of Pain Management 9(3): 255-264. | wrong intervention | |
| 1264 | Howard, A. (2019). "Ministerial Responsibility in Cases of Serious Policy Failure: The Role of Extra-Parliamentary Inquiry in Upholding Political Legitimacy." AUSTRALASIAN PARLIAMENTARY REVIEW 34(1): 48-69. | wrong intervention | |
| 1265 | Hsiao, A. F., et al. (2012). "A Randomized Controlled Study to Evaluate the Efficacy of Noninvasive Limb Cover for Chronic Phantom Limb Pain Among Veteran Amputees." ARCHIVES OF PHYSICAL MEDICINE AND REHABILITATION 93(4): 617-622. | wrong intervention | |
| 1266 | Hsieh, C.-C., et al. (2018). "The effectiveness of a virtual reality-based tai chi exercise on cognitive and physical function in older adults with cognitive impairment." Dementia and Geriatric Cognitive Disorders 46(5-6): 358-370. | wrong intervention | |
| 1267 | Hsieh, C. H., et al. (2022). "The effects of immersive garden experience on the health care to elderly residents with mild-to-moderate cognitive impairment living in nursing homes after the COVID-19 pandemic." LANDSCAPE AND ECOLOGICAL ENGINEERING 18(1): 45-56. | wrong design | |
| 1268 | Hsieh, C. H. and D. Y. Li (2022). "Understanding how virtual reality forest experience promote physiological and psychological health for patients undergoing hemodialysis." FRONTIERS IN PSYCHIATRY 13. | wrong population | |
| 1269 | Hsieh, C. H., et al. (2023). "The effect of water sound level in virtual reality: A study of restorative benefits in young adults through immersive natural environments." JOURNAL OF ENVIRONMENTAL PSYCHOLOGY 88. | wrong population | |
| 1270 | Hsieh, P. L., et al. (2023). "Exploring Key Factors Influencing Nursing Students' Cognitive Load and Willingness to Serve Older Adults: Cross-sectional Descriptive Correlational Study." JMIR SERIOUS GAMES 11. | wrong population | |
| 1271 | Hsieh, P.-L., et al. (2023). "Exploring key factors influencing nursing students’ cognitive load and willingness to serve older adults: Cross-sectional descriptive correlational study." JMIR Serious Games 11: 10. | wrong population | |
| 1272 | Hsu, M. F., et al. (2022). "Effectiveness of Virtual Reality Interactive Play for Children During Intravenous Placement: A Randomized Controlled Trial." ASIAN NURSING RESEARCH 16(2): 87-93. | wrong population | |
| 1273 | Hu, F. H., et al. (2024). "Suicide in China: The Trends and Differentials From 2002 to 2021." CLINICAL PSYCHOLOGY & PSYCHOTHERAPY 31(5). | wrong intervention | |
| 1274 | Hu, G., et al. (2017). "Extensive glacial advances during the Last Glacial Maximum near the eastern Himalayan syntaxis." QUATERNARY INTERNATIONAL 443: 1-12. | wrong intervention | |
| 1275 | Hu, Q. F., et al. (2012). "Acute effects of warm footbath on arterial stiffness in healthy young and older women." EUROPEAN JOURNAL OF APPLIED PHYSIOLOGY 112(4): 1261-1268. | wrong population | |
| 1276 | Hu, X., et al. (2019). "Interdependent self-construal modulates the adrenocortical stress response in the socially evaluated cold-pressor test." STRESS-THE INTERNATIONAL JOURNAL ON THE BIOLOGY OF STRESS 22(6): 679-686. | wrong intervention | |
| 1277 | Hu, Y., et al. (2023). "Virtual Reality in Clinical Nursing Practice Over the Past 10 Years: Umbrella Review of Meta-Analyses." JMIR Serious Games 11(1). | wrong intervention | |
| 1278 | Hu, Y. J., et al. (2023). "Virtual Reality in Clinical Nursing Practice Over the Past 10 Years: Umbrella Review of Meta-Analyses." JMIR SERIOUS GAMES 11. | wrong intervention | |
| 1279 | Hua, Y., et al. (2015). "The Effect of Virtual Reality Distraction on Pain Relief During Dressing Changes in Children with Chronic Wounds on Lower Limbs." PAIN MANAGEMENT NURSING 16(5): 685-691. | wrong population | |
| 1280 | Huang, C. C., et al. (2022). "Concurrent chemoradiation therapy is associated with an accelerated risk of cardiovascular autonomic dysfunction in patients with nasopharyngeal carcinoma: A 9-year prospective follow-up study." RADIOTHERAPY AND ONCOLOGY 170: 129-135. | wrong intervention | |
| 1281 | Huang, C. M., et al. (2011). "Radial Pressure Pulse and Heart Rate Variability in Heat- and Cold-Stressed Humans." EVIDENCE-BASED COMPLEMENTARY AND ALTERNATIVE MEDICINE 2011: 1-9. | wrong intervention | |
| 1282 | Huang, D., et al. (2022). "Effects of virtual reality working memory training on event-based prospective memory in patients with major depressive disorder." JOURNAL OF PSYCHIATRIC RESEARCH 156: 91-99. | wrong population | |
| 1283 | Huang, K.-T. (2020). "Exergaming executive functions: An immersive virtual reality-based cognitive training for adults aged 50 and older." Cyberpsychology, Behavior, and Social Networking 23(3): 143-149. | wrong population | |
| 1284 | Huang, K. T. (2020). "Exergaming Executive Functions: An Immersive Virtual Reality-Based Cognitive Training for Adults Aged 50 and Older." CYBERPSYCHOLOGY BEHAVIOR AND SOCIAL NETWORKING 23(3): 143-149. | duplicate |  |
| 1285 | Huang, K. X., et al. (2022). "Exergame-based exercise training for depressive symptoms in adults: A systematic review and meta-analysis." PSYCHOLOGY OF SPORT AND EXERCISE 63. | wrong outcomes | |
| 1286 | Huang, L. C. and Y. H. Yang (2022). "The Long-term Effects of Immersive Virtual Reality Reminiscence in People With Dementia: Longitudinal Observational Study." JMIR SERIOUS GAMES 10(3). | wrong outcomes | |
| 1287 | Huang, L.-C. and Y.-H. Yang (2022). "The long-term effects of immersive virtual reality reminiscence in people with dementia: Longitudinal observational study." JMIR Serious Games 10(3): 1-9. | wrong outcomes | |
| 1288 | Huang, L. H. (2019). "Well-being and volunteering: Evidence from aging societies in Asia." Soc Sci Med 229: 172-180. | wrong intervention | |
| 1289 | Huang, M. X. and T. Habermas (2021). "Narrating ambiguous loss: Deficiencies in narrative processing and negative appraisal of consequences." JOURNAL OF CLINICAL PSYCHOLOGY 77(10): 2147-2166. | wrong intervention | |
| 1290 | Huang, Q. R., et al. (2022). "Using Virtual Reality Exposure Therapy in Pain Management: A Systematic Review and Meta-Analysis of Randomized Controlled Trials." VALUE IN HEALTH 25(2): 288-301. | wrong outcomes | |
| 1291 | Huang, S. P., et al. (2021). "The Contribution to Stress Recovery and Attention Restoration Potential of Exposure to Urban Green Spaces in Low-Density Residential Areas." INTERNATIONAL JOURNAL OF ENVIRONMENTAL RESEARCH AND PUBLIC HEALTH 18(16). | wrong intervention | |
| 1292 | Huang, W. H. D., et al. (2013). "Gender divide and acceptance of collaborative Web 2.0 applications for learning in higher education." INTERNET AND HIGHER EDUCATION 16: 57-65. | wrong intervention | |
| 1293 | Huang, Y., et al. (2024). "Virtual reality‐based simulation intervention for enhancing the empathy of informal caregivers of people with dementia: A mixed‐methods systematic review." International Journal of Mental Health Nursing 33(2): 241-258. | wrong outcomes | |
| 1294 | Huang, Y. Q., et al. (2024). "Virtual reality-based simulation intervention for enhancing the empathy of informal caregivers of people with dementia: A mixed-methods systematic review." INTERNATIONAL JOURNAL OF MENTAL HEALTH NURSING 33(2): 241-258. | wrong outcomes | |
| 1295 | Huber, S. K., et al. (2021). "Personalized Motor-Cognitive Exergame Training in Chronic Stroke Patients-A Feasibility Study." Front Aging Neurosci 13: 730801. | wrong intervention | |
| 1296 | Huber, S. K., et al. (2021). "Personalized motor-cognitive exergame training in chronic stroke patients—A feasibility study." Frontiers in Aging Neuroscience 13: 19. | wrong intervention | |
| 1297 | Huddleston, H. P., et al. (2023). "Outcomes of Patellar Subchondroplasty Surgery: A Case Series." JOURNAL OF KNEE SURGERY 36(09): 941-948. | wrong intervention | |
| 1298 | Huerta, A. G. G., et al. (2018). Augmented Reality in Tablets for the Yerkes Test for Older Adults. HUMAN ASPECTS OF IT FOR THE AGED POPULATION: APPLICATIONS IN HEALTH, ASSISTANCE, AND ENTERTAINMENT, PT II. 10927: 36-48. | wrong intervention | |
| 1299 | Huizeling, E., et al. (2020). "Age-Related Changes in Attentional Refocusing during Simulated Driving." BRAIN SCIENCES 10(8). | wrong intervention | |
| 1300 | Humbert, A., et al. (2023). "Acceptability, feasibility, and user satisfaction of a virtual reality relaxation intervention in a psychiatric outpatient setting during the COVID-19 pandemic." FRONTIERS IN PSYCHIATRY 14. | wrong outcomes | |
| 1301 | Humblin, E., et al. (2023). "Sustained CD28 costimulation is required for self-renewal and differentiation of TCF-1(+) PD-1(+) CD8 T cells." Sci Immunol 8(86): eadg0878. | wrong intervention | |
| 1302 | Hummer, T. A., et al. (2023). "Assessment of Boys' Responses to Interpersonal Conflict in Virtual Reality." GAMES FOR HEALTH JOURNAL 12(1): 53-62. | wrong outcomes | |
| 1303 | Humphris, G., et al. (2019). "Emotional talk of patients with breast cancer during review appointments with therapeutic radiographers: effects on fears of cancer recurrence." SUPPORTIVE CARE IN CANCER 27(6): 2143-2151. | wrong intervention | |
| 1304 | Hundert, A. S., et al. (2022). "A Pilot Randomized Controlled Trial of Virtual Reality Distraction to Reduce Procedural Pain During Subcutaneous Port Access in Children and Adolescents With Cancer." CLINICAL JOURNAL OF PAIN 38(3): 189-196. | wrong population | |
| 1305 | Hung, L., et al. (2023). "Facilitators and Barriers to Using Virtual Reality and its Impact on Social Engagement in Aged Care Settings: A Scoping Review." Gerontol Geriatr Med 9: 23337214231166355. | wrong outcomes | |
| 1306 | Hung, L. L., et al. (2023). "Facilitators and Barriers to Using Virtual Reality and its Impact on Social Engagement in Aged Care Settings: A Scoping Review." GERONTOLOGY AND GERIATRIC MEDICINE 9. | wrong outcomes | |
| 1307 | Hung, T. C., et al. (2018). "Hot Water Bathing Impairs Training Adaptation in Elite Teen Archers." CHINESE JOURNAL OF PHYSIOLOGY 61(2): 118-123. | wrong population | |
| 1308 | Hur, J. W., et al. (2021). "Virtual Reality-Based Psychotherapy in Social Anxiety Disorder: fMRI Study Using a Self-Referential Task." JMIR MENTAL HEALTH 8(4). | wrong population | |
| 1309 | Hussain, A., et al. (2023). An exploratory study on virtual reality and in person effects on loneliness. 2023 IEEE INTERNATIONAL SYMPOSIUM ON TECHNOLOGY AND SOCIETY, ISTAS. | wrong outcomes | |
| 1310 | Hutson, J. M. (2000). Cryptorchidism and Hypospadias. Endotext. K. R. Feingold, B. Anawalt, M. R. Blackman et al. South Dartmouth (MA), MDText.com, Inc. | wrong intervention | |
| 1311 | Huttner, J. P., et al. (2017). An Immersive Memory Palace: Supporting the Method of Loci with Virtual Reality. AMCIS 2017 PROCEEDINGS. | wrong outcomes | |
| 1312 | Huygelier, H., et al. (2021). "The Use of the Term Virtual Reality in Post-Stroke Rehabilitation: A Scoping Review and Commentary." PSYCHOLOGICA BELGICA 61(1): 145-162. | wrong outcomes | |
| 1313 | Hwang, G., et al. (2023). "Assessment of neuroanatomical endophenotypes of autism spectrum disorder and association with characteristics of individuals with schizophrenia and the general population." JAMA Psychiatry 80(5): 498-507. | wrong intervention | |
| 1314 | Hwang, N. K., et al. (2021). "Effects of Semi-Immersive Virtual Reality-Based Cognitive Training Combined with Locomotor Activity on Cognitive Function and Gait Ability in Community-Dwelling Older Adults." HEALTHCARE 9(7). | wrong outcomes | |
| 1315 | Hwang, S., et al. (2020). "Driving Performance Evaluation Correlated to Age and Visual Acuities Based on VR Technologies." JOURNAL OF ADVANCED TRANSPORTATION 2020. | wrong intervention | |
| 1316 | Hyland, E. J., et al. (2015). "An assessment of early Child Life Therapy pain and anxiety management: A prospective randomised controlled trial." BURNS 41(8): 1642-1652. | wrong population | |
| 1317 | Iachini, T., et al. (2019). "The Effect of Body-Related Stimuli on Mental Rotation in Children, Young and Elderly Adults." Sci Rep 9(1): 1169. | wrong population | |
| 1318 | Idkowiak, J., et al. (1993). Cytochrome P450 Oxidoreductase Deficiency. GeneReviews(®). M. P. Adam, J. Feldman, G. M. Mirzaa et al. Seattle (WA), University of Washington, Seattle | wrong intervention | |
| 1319 | Ifearulundu, I. H., et al. (2023). "Determinants of Postoperative Compliance of Patient-Reported Outcome Assessments following Lumbar Spine Surgery." SPINE SURGERY AND RELATED RESEARCH 7(2): 161-169. | wrong intervention | |
| 1320 | Ijaz, K., et al. (2019). "An immersive virtual reality platform for assessing spatial navigation memory in predementia screening: Feasibility and usability study." JMIR Mental Health 6(9). | wrong outcomes | |
| 1321 | Ikeda, Y., et al. (2022). "Effects of vasopressin receptor agonists on detrusor smooth muscle tone in young and aged bladders: Implications for nocturia treatment." Continence (Amst) 2. | wrong population | |
| 1322 | Iliadou, P., et al. (2021). "A comparison of traditional and serious game-based digital markers of cognition in older adults with mild cognitive impairment and healthy controls." Journal of Alzheimer's Disease 79(4): 1747-1759. | wrong intervention | |
| 1323 | Ilioudi, M., et al. (2023). "Physical Versus Virtual Reality-Based Calm Rooms for Psychiatric Inpatients: Quasi-Randomized Trial." JOURNAL OF MEDICAL INTERNET RESEARCH 25. | wrong population | |
| 1324 | Imai, A., et al. (2024). "Effectiveness of a Virtual Reality Open-Air Bath Program inReducing Loneliness and Improving Brain Function for DementiaPrevention in Older Adults:Protocol for a Prospective RandomizedCrossover Study." JMIR RESEARCH PROTOCOLS 13. | wrong outcomes | |
| 1325 | Imai, A., et al. (2024). "Effectiveness of a Virtual Reality Open-Air Bath Program in Reducing Loneliness and Improving Brain Function for Dementia Prevention in Older Adults: Protocol for a Prospective Randomized Crossover Study." JMIR Res Protoc 13: e57101. | wrong outcomes | |
| 1326 | Imaoka, Y., et al. (2022). "Linking cognitive functioning and postural balance control through virtual reality environmental manipulations." Frontiers in Aging Neuroscience 14. | wrong outcomes | |
| 1327 | Imataka, G., et al. (2024). "Gaming Disorders: Navigating the Fine Line between Entertainment and Addiction-Gaming History, Health Risks, Social Consequences, and Pathways to Prevention." JOURNAL OF CLINICAL MEDICINE 13(17). | wrong intervention | |
| 1328 | Inangil, D., et al. (2020). "Efficacy of Cartoon Viewing Devices During Phlebotomy in Children: A Randomized Controlled Trial." JOURNAL OF PERIANESTHESIA NURSING 35(4): 407-412. | wrong population | |
| 1329 | Inglis, H. (2018). "Fear plus Time." CANADIAN THEATRE REVIEW 173: 36-47. | wrong intervention | |
| 1330 | Inokoshi, M., et al. (2013). "Durable bonding to mechanically and/or chemically pre-treated dental zirconia." J Dent 41(2): 170-179. | wrong intervention | |
| 1331 | Inoue, K., et al. (2024). "A pilot study on the use of virtual reality to educate community members about dementia." Physical & Occupational Therapy in Geriatrics 42(2): 170-185. | wrong outcomes | |
| 1332 | Iosa, M., et al. (2015). "Leap motion controlled videogame-based therapy for rehabilitation of elderly patients with subacute stroke: A feasibility pilot study." Topics in Stroke Rehabilitation 22(4): 306-316. | wrong intervention | |
| 1333 | Ip, H. H. S., et al. (2016). Virtual Reality Enabled Training for Social Adaptation in Inclusive Education Settings for School-Aged Children with Autism Spectrum Disorder (ASD). BLENDED LEARNING: ALIGNING THEORY WITH PRACTICES, ICBL 2016. 9757: 94-102. | wrong population | |
| 1334 | Isaacson, M. (2014). "Clarifying concepts: cultural humility or competency." J Prof Nurs 30(3): 251-258. | wrong intervention | |
| 1335 | Isernia, S., et al. (2021). "Diagnostic validity of the smart aging serious game: An innovative tool for digital phenotyping of mild neurocognitive disorder." Journal of Alzheimer's Disease 83(4): 1789-1801. | wrong intervention | |
| 1336 | Isernia, S., et al. (2020). "Effects of an Innovative Telerehabilitation Intervention for People With Parkinson's Disease on Quality of Life, Motor, and Non-motor Abilities." Front Neurol 11: 846. | wrong intervention | |
| 1337 | Isernia, S., et al. (2020). "Effects of an Innovative Telerehabilitation Intervention for People With Parkinson's Disease on Quality of Life, Motor, and Non-motor Abilities." FRONTIERS IN NEUROLOGY 11. | wrong intervention | |
| 1338 | Islam, A., et al. (2022). "Stress upregulates 2-arachidonoylglycerol levels in the hypothalamus, midbrain, and hindbrain, and it is sustained by green nut oil supplementation in SAMP8 mice revealed by DESI-MSI." Biochem Biophys Res Commun 609: 9-14. | wrong intervention | |
| 1339 | Ito, A., et al. (2023). "Feasibility Study of Virtual Reality-Based Cognitive Behavioral Therapy for Patients With Depression: Protocol for an Open Trial and Therapeutic Intervention." JMIR RESEARCH PROTOCOLS 12. | wrong outcomes | |
| 1340 | Ivancic, P. C. (2014). "Plough fracture of the anterior arch of the atlas: A biomechanical investigation." European Spine Journal 23(11): 2314-2320. | wrong intervention | |
| 1341 | Ivanov, I., et al. (2017). "Blast Exposure, White Matter Integrity, and Cognitive Function in Iraq and Afghanistan Combat Veterans." Front Neurol 8: 127. | wrong intervention | |
| 1342 | Iwanaga, K., et al. (2024). "Demographic covariates and vocational rehabilitation services as predictors of employment outcomes of people with physical disabilities: a hierarchical logistic regression analysis." DISABILITY AND REHABILITATION. | wrong intervention | |
| 1343 | Iyadurai, L., et al. (2023). "Reducing intrusive memories after trauma via an imagery-competing task intervention in COVID-19 intensive care staff: a randomised controlled trial." Transl Psychiatry 13(1): 290. | wrong intervention | |
| 1344 | Izountar, Y., et al. (2022). "VR-PEER: A Personalized Exer-Game Platform Based on Emotion Recognition." ELECTRONICS 11(3). | wrong intervention | |
| 1345 | Jabali, O., et al. (2024). "Political socialization and its impact on psychological resilience and PTSD among individuals engaged with Israeli occupation forces on Mount Sabih." EUROPEAN JOURNAL OF TRAUMA & DISSOCIATION 8(2). | wrong intervention | |
| 1346 | Jabbar, A. A. (2022). "Gastroprotective and Immuno-supportive Role of <i>Alcea kurdica</i> against Stress Induced Lesion in Japanese Quails." BAGHDAD SCIENCE JOURNAL 19(4): 716-724. | wrong intervention | |
| 1347 | Jacek, S. (2015). "Cave development in an uplifting fold-and-thrust belt: case study of the Tatra Mountains, Poland." INTERNATIONAL JOURNAL OF SPELEOLOGY 44(3): 341-359. | wrong intervention | |
| 1348 | Jackson, B., et al. (2017). "Re-Thinking Anxiety: Using Inoculation Messages to Reduce and Reinterpret Public Speaking Fears." PLOS ONE 12(1). | wrong intervention | |
| 1349 | Jacob, N. D., et al. (2015). Statistical Texture Analysis of Morphological Changes in Pressboard Insulation Due to Thermal Aging and Partial Discharges. 2015 IEEE ELECTRICAL INSULATION CONFERENCE (EIC): 610-613. | wrong intervention | |
| 1350 | Jacobs, B. L., et al. (2019). "Change in Functional Status After Prostate Cancer Treatment Among Medicare Advantage Beneficiaries." UROLOGY 131: 104-111. | wrong intervention | |
| 1351 | Jacobs, C. A., et al. (2017). "Preoperative Symptoms in Femoroacetabular Impingement Patients Are More Related to Mental Health Scores Than the Severity of Labral Tear or Magnitude of Bony Deformity." JOURNAL OF ARTHROPLASTY 32(12): 3603-3606. | wrong intervention | |
| 1352 | Jacobs, E., et al. (2010). "Developmental and Behavioral Performance of Internationally Adopted Preschoolers: A Pilot Study." CHILD PSYCHIATRY & HUMAN DEVELOPMENT 41(1): 15-29. | wrong population | |
| 1353 | Jacobson, M. J., et al. (2021). "Heterogenous Late Holocene Climate in the Eastern Mediterranean-The Kocain Cave Record From SW Turkey." GEOPHYSICAL RESEARCH LETTERS 48(20). | wrong intervention | |
| 1354 | Jacobson, N., et al. (2022). "The Effect of Transcranial Alternating Current Stimulation With Cognitive Training on Executive Brain Function in Individuals With Dementia: Protocol for a Crossover Randomized Controlled Trial." JMIR Res Protoc 11(4): e37282. | wrong outcomes | |
| 1355 | Jacobson, N., et al. (2022). "The Effect of Transcranial Alternating Current Stimulation With Cognitive Training on Executive Brain Function in Individuals With Dementia: Protocol for a Crossover Randomized Controlled Trial." JMIR RESEARCH PROTOCOLS 11(4). | wrong outcomes | |
| 1356 | Jahn, F. S., et al. (2021). "Cognitive training with fully immersive virtual reality in patients with neurological and psychiatric disorders: A systematic review of randomized controlled trials." PSYCHIATRY RESEARCH 300. | wrong outcomes | |
| 1357 | Jamalian, S., et al. (2023). "Modeling Alzheimer's disease progression utilizing clinical trial and ADNI data to predict longitudinal trajectory of CDR-SB." CPT Pharmacometrics Syst Pharmacol 12(7): 1029-1042. | wrong intervention | |
| 1358 | Jamin, A., et al. (2021). "Age-related alterations on the capacities to navigate on a bike: use of a simulator and entropy measures." MEDICAL & BIOLOGICAL ENGINEERING & COMPUTING 59(1): 13-22. | wrong intervention | |
| 1359 | Jamnik, P. and B. Blazina (2023). "IS<i> TURJEVA</i><i> JAMA</i> ABOVE THE NATISONE RIVER (THE KOBARID REGION) A BRONZE AGE RITUAL AREA OF FERTILITY OR NATALITY?" ANNALES-ANALI ZA ISTRSKE IN MEDITERANSKE STUDIJE-SERIES HISTORIA ET SOCIOLOGIA 33(1). | wrong intervention | |
| 1360 | Jang, S., et al. (2022). "Virtual reality-based monitoring test for MCI: A multicenter feasibility study." Front Psychiatry 13: 1057513. | wrong outcomes | |
| 1361 | Jaques, E. D., et al. (2023). "Conventional Mirror Therapy versus Immersive Virtual Reality Mirror Therapy: The Perceived Usability after Stroke." STROKE RESEARCH AND TREATMENT 2023. | wrong outcomes | |
| 1362 | Jasper, A., et al. (2023). "Predicting cybersickness using individual and task characteristics." COMPUTERS IN HUMAN BEHAVIOR 146. | wrong intervention | |
| 1363 | Jawed, Y. T., et al. (2021). "Feasibility of a virtual reality intervention in the intensive care unit." Heart and Lung 50(6): 748-753. | wrong population | |
| 1364 | Jayasinghe, N., et al. (2022). "Brief training of gait and posture using a wearable sensory feedback device with older adults who have fears of falling: A feasibility study." HEALTH AND TECHNOLOGY 12(2): 433-442. | wrong intervention | |
| 1365 | Jayasinghe, N., et al. (2017). "Systematic Review of the Clinical Application of Exposure Techniques to Community-Dwelling Older Adults with Anxiety." CLINICAL GERONTOLOGIST 40(3): 141-158. | wrong outcomes | |
| 1366 | Jebara, N., et al. (2014). "Effects of enactment in episodic memory: A pilot virtual reality study with young and elderly adults." Frontiers in Aging Neuroscience 6: 16. | wrong population | |
| 1367 | Jeffs, D., et al. (2014). "Effect of Virtual Reality on Adolescent Pain During Burn Wound Care." JOURNAL OF BURN CARE & RESEARCH 35(5): 395-408. | wrong population | |
| 1368 | Jeffs, D. A., et al. (2024). "Comparing novel virtual reality and nursing standard care on burn wound care pain in adolescents: A randomized controlled trial." JOURNAL FOR SPECIALISTS IN PEDIATRIC NURSING 29(1). | wrong population | |
| 1369 | Jelcic, N., et al. (2014). "Feasibility and efficacy of cognitive telerehabilitation in early Alzheimer's disease: a pilot study." CLINICAL INTERVENTIONS IN AGING 9: 1605-1611. | wrong intervention | |
| 1370 | Jerardino, A., et al. (2021). "VISITS TO A CLIFF CAVE AMIDST CLIMATE CHANGE: THE ARCHAEOLOGY OF SPRING CAVE, WEST COAST OF SOUTH AFRICA." SOUTH AFRICAN ARCHAEOLOGICAL BULLETIN 76(215): 109-124. | wrong intervention | |
| 1371 | Jespersen, A. E., et al. (2024). "Effect of immersive virtual reality-based cognitive remediation in patients with mood or psychosis spectrum disorders: study protocol for a randomized, controlled, double-blinded trial." TRIALS 25(1). | wrong outcomes | |
| 1372 | Jeun, Y. J., et al. (2022). "Effects of Personalized Cognitive Training with the Machine Learning Algorithm on Neural Efficiency in Healthy Younger Adults." INTERNATIONAL JOURNAL OF ENVIRONMENTAL RESEARCH AND PUBLIC HEALTH 19(20). | wrong population | |
| 1373 | Ji, W. B., et al. (2021). "Impact of early socialization environment on social behavior, physiology and growth performance of weaned piglets." APPLIED ANIMAL BEHAVIOUR SCIENCE 238. | wrong intervention | |
| 1374 | Ji, W. B., et al. (2021). "Effects of two different early socialization models on social behavior and physiology of suckling piglets." APPLIED ANIMAL BEHAVIOUR SCIENCE 243. | wrong intervention | |
| 1375 | Jia, H. M., et al. (2018). "Comparing the Performance of 2 Health Utility Measures in the Medicare Health Outcome Survey (HOS)." MEDICAL DECISION MAKING 38(8): 983-993. | wrong intervention | |
| 1376 | Jia, J. J., et al. (2015). "Dermatofibrosarcoma protuberans with pit-like lesions: A case report and literature review." ONCOLOGY LETTERS 10(6): 3765-3768. | wrong intervention | |
| 1377 | Jian, B. R., et al. (2024). "Influence of virtual heights and a cognitive task on standing postural steadiness." INTERNATIONAL JOURNAL OF INDUSTRIAL ERGONOMICS 100. | wrong intervention | |
| 1378 | Jiang, S., et al. (2017). "Effect of the Biofilm Age and Starvation on Acid Tolerance of Biofilm Formed by <i>Streptococcus mutans</i> Isolated from Caries-Active and Caries-Free Adults." INTERNATIONAL JOURNAL OF MOLECULAR SCIENCES 18(4). | wrong intervention | |
| 1379 | Jiang, W., et al. (2015). "Towards early-age performance control in precast concrete immersed tunnels." STRUCTURAL CONCRETE 16(4): 558-571. | wrong intervention | |
| 1380 | Jiang, X., et al. (2019). "Shared heritability and functional enrichment across six solid cancers." Nat Commun 10(1): 431. | wrong intervention | |
| 1381 | Jibb, L. A., et al. (2015). "Psychological and Physical Interventions for the Management of Cancer-Related Pain in Pediatric and Young Adult Patients: An Integrative Review." ONCOLOGY NURSING FORUM 42(6): E339-E357. | wrong population | |
| 1382 | Jin, F. G., et al. (2024). "Effectiveness and safety of Buzzy device in needle-related procedures for children under twelve years of age: A systematic review and meta-analysis." MEDICINE 103(15). | wrong population | |
| 1383 | Jin, R., et al. (2020). "Current Cognition Tests, Potential Virtual Reality Applications, and Serious Games in Cognitive Assessment and Non-Pharmacological Therapy for Neurocognitive Disorders." J Clin Med 9(10). | wrong intervention | |
| 1384 | Jin, R., et al. (2020). "Current Cognition Tests, Potential Virtual Reality Applications, and Serious Games in Cognitive Assessment and Non-Pharmacological Therapy for Neurocognitive Disorders." JOURNAL OF CLINICAL MEDICINE 9(10). | wrong intervention | |
| 1385 | Jin, X., et al. (2022). "Effects of pH alterations on stress- and aging-induced protein phase separation." Cell Mol Life Sci 79(7): 380. | wrong intervention | |
| 1386 | Jin, X. G., et al. (2021). "The Mechanical Properties of Early Aged Shotcrete under Internal Sulfate Attack." MATERIALS 14(13). | wrong intervention | |
| 1387 | Jivraj, B. A., et al. (2020). "The use of virtual reality in reducing anxiety during cast removal: a randomized controlled trial." JOURNAL OF CHILDRENS ORTHOPAEDICS 14(6): 574-580. | wrong population | |
| 1388 | Jo, G., et al. (2018). "Effects of virtual reality exercise for Korean adults with schizophrenia in a closed ward." J Exerc Rehabil 14(1): 39-48. | wrong population | |
| 1389 | Johansson, B. B. (2012). "Multisensory stimulation in stroke rehabilitation." FRONTIERS IN HUMAN NEUROSCIENCE 6. | wrong intervention | |
| 1390 | Johnson, J., et al. (2013). "Impact of tachycardia and sympathetic stimulation by cold pressor test on cardiac diastology and arterial function in elderly females." AMERICAN JOURNAL OF PHYSIOLOGY-HEART AND CIRCULATORY PHYSIOLOGY 304(7): H1002-H1009. | wrong intervention | |
| 1391 | Johnson, S., et al. (2014). "Stereotype confirmation concerns predict dropout from cognitive behavioral therapy for social anxiety disorder." BMC PSYCHIATRY 14. | wrong intervention | |
| 1392 | Johnson, S. B. and P. L. Anderson (2016). "Don't ask, don't tell: a systematic review of the extent to which participant characteristics are reported in social anxiety treatment studies." ANXIETY STRESS AND COPING 29(6): 589-605. | wrong intervention | |
| 1393 | Jones, K. E., et al. (2019). Reducing Anxiety for Dental Visits. HUMAN-COMPUTER INTERACTION - INTERACT 2019, PT IV. 11749: 659-663. | wrong intervention | |
| 1394 | Jongvilaikasem, K., et al. (2020). Dissolved Gas Behavior in Natural Ester under Corona, Surface, and Internal Discharges. 2020 8TH INTERNATIONAL CONFERENCE ON CONDITION MONITORING AND DIAGNOSIS (CMD 2020): 326-329. | wrong intervention | |
| 1395 | Jönsson, P., et al. (2010). "Cardiovascular and cortisol reactivity and habituation to a virtual reality version of the Trier Social Stress Test: A pilot study." PSYCHONEUROENDOCRINOLOGY 35(9): 1397-1403. | wrong outcomes | |
| 1396 | Józwik, S., et al. (2021). "Evaluation of the Impact of Virtual Reality-Enhanced Cardiac Rehabilitation on Depressive and Anxiety Symptoms in Patients with Coronary Artery Disease: A Randomised Controlled Trial." JOURNAL OF CLINICAL MEDICINE 10(10). | wrong population | |
| 1397 | Juan, S. M. A., et al. (2022). "Tau Pathology, Metal Dyshomeostasis and Repetitive Mild Traumatic Brain Injury: An Unexplored Link Paving the Way for Neurodegeneration." JOURNAL OF NEUROTRAUMA 39(13-14): 902-922. | wrong intervention | |
| 1398 | Júlio, F., et al. (2022). "Cognition, function and awareness of disease impact in early Parkinson’s and Huntington’s disease." Disability and Rehabilitation: An International, Multidisciplinary Journal 44(6): 921-939. | wrong intervention | |
| 1399 | Jun, G. R., et al. (2017). "Transethnic genome-wide scan identifies novel Alzheimer's disease loci." Alzheimers Dement 13(7): 727-738. | wrong intervention | |
| 1400 | Jung, M. J., et al. (2021). "Pediatric Distraction on Induction of Anesthesia With Virtual Reality and Perioperative Anxiolysis: A Randomized Controlled Trial." ANESTHESIA AND ANALGESIA 132(3): 798-806. | wrong population | |
| 1401 | Jusner, P., et al. (2022). "Analyzing the effects of thermal stress on insulator papers by solid-state <SUP>13</SUP>C NMR spectroscopy." CELLULOSE 29(2): 1081-1095. | wrong intervention | |
| 1402 | Jusner, P., et al. (2022). "Protocol for characterizing the molar mass distribution and oxidized functionality profiles of aged transformer papers by gel permeation chromatography (GPC)." CELLULOSE 29(4): 2241-2256. | wrong intervention | |
| 1403 | Just, S. A., et al. (2024). "Feasibility of using virtual reality in geriatric psychiatry." INTERNATIONAL JOURNAL OF GERIATRIC PSYCHIATRY 39(1). | wrong intervention | |
| 1404 | Just, S. A., et al. (2024). "Feasibility of using virtual reality in geriatric psychiatry." International Journal of Geriatric Psychiatry 39(1): 1-10. | wrong intervention | |
| 1405 | Jütten, L. H., et al. (2017). "Testing the effectivity of the mixed virtual reality training Into D'mentia for informal caregivers of people with dementia: protocol for a longitudinal, quasi-experimental study." BMJ Open 7(8): e015702. | wrong intervention | |
| 1406 | Jyskä, I., et al. (2023). "Design and User Experience of VirNE Application: Deep Breathing Exercise in a Virtual Natural Environment to Reduce Treatment Anxiety in Pediatrics." HEALTHCARE 11(24). | wrong population | |
| 1407 | Jyskä, I., et al. (2023). "Effects of Using Guided Deep Breathing Exercises in a Virtual Natural Environment to Reduce Stress during Pediatric Treatment." HEALTHCARE 11(24). | wrong population | |
| 1408 | Kabiri, M., et al. (2018). "Long-Term Health and Economic Value of Improved Mobility among Older Adults in the United States." Value in Health 21(7): 792-798. | wrong intervention | |
| 1409 | Kadhum, A., et al. (2021). "Barriers to successful dichoptic treatment for amblyopia in young children." Graefes Arch Clin Exp Ophthalmol 259(10): 3149-3157. | wrong population | |
| 1410 | Kafes, A. Y., et al. (2024). "Virtual Reality Supported Intervention Program for Trauma Symptoms of Individuals Who Experienced an Earthquake: An Effectiveness Study." NOROPSIKIYATRI ARSIVI-ARCHIVES OF NEUROPSYCHIATRY 61(1): 15-23. | wrong population | |
| 1411 | Kahlbaugh, P. E., et al. (2011). "Effects of Playing Wii on Well-Being in the Elderly: Physical Activity, Loneliness, and Mood." Activities, Adaptation & Aging 35(4): 331-344. | wrong intervention | |
| 1412 | Kahlmeter, A. (2023). "Severe violent victimization and labour market exclusion. The significance of the victim-offender overlap." EUROPEAN JOURNAL OF CRIMINOLOGY 20(3): 1081-1105. | wrong intervention | |
| 1413 | Kahlon, S., et al. (2019). "Virtual reality exposure therapy for adolescents with fear of public speaking: a non-randomized feasibility and pilot study." CHILD AND ADOLESCENT PSYCHIATRY AND MENTAL HEALTH 13(1). | wrong population | |
| 1414 | Kahlon, S., et al. (2023). "Gamified virtual reality exposure therapy for adolescents with public speaking anxiety: a four-armed randomized controlled trial." FRONTIERS IN VIRTUAL REALITY 4. | wrong population | |
| 1415 | Kaimal, G., et al. (2020). "Outcomes of Visual Self-Expression in Virtual Reality on Psychosocial Well-Being With the Inclusion of a Fragrance Stimulus: A Pilot Mixed-Methods Study." FRONTIERS IN PSYCHOLOGY 11. | wrong population | |
| 1416 | Kalantari, S., et al. (2022). "Using a Nature-based Virtual Reality Environment for Improving Mood States and Cognitive Engagement in Older Adults: A Mixed-method Feasibility Study." INNOVATION IN AGING 6(3). | wrong design | |
| 1417 | Kalemkus, F. (2024). "Trends in instructional technologies used in education of people with special needs due to intellectual disability and autism." JOURNAL OF RESEARCH IN SPECIAL EDUCATIONAL NEEDS. | wrong intervention | |
| 1418 | Kalkhoff, S., et al. (2023). "Immersive VR-Based Patient Journey." Thoracic and Cardiovascular Surgeon 71. | wrong outcomes | |
| 1419 | Kaltsas, G. and K. Tsiveriotis (2000). Fibromyalgia. Endotext. K. R. Feingold, B. Anawalt, M. R. Blackman et al. South Dartmouth (MA), MDText.com, Inc. | wrong intervention | |
| 1420 | Kambakamba, P., et al. (2024). "Applying augmented reality in teaching of surgical residents-telementoring, a "stress-free" way to surgical autonomy?" LANGENBECKS ARCHIVES OF SURGERY 409(1). | wrong population | |
| 1421 | Kambakamba, P., et al. (2024). "Applying augmented reality in teaching of surgical residents—telementoring, a “stress-free” way to surgical autonomy?" Langenbeck's Archives of Surgery 409(1). | wrong population | |
| 1422 | Kaminska, M. S., et al. (2018). "The effectiveness of virtual reality training in reducing the risk of falls among elderly people." CLINICAL INTERVENTIONS IN AGING 13: 2329-2338. | wrong design | |
| 1423 | Kampmann, I. L., et al. (2016). "Exposure to virtual social interactions in the treatment of social anxiety disorder: A randomized controlled trial." BEHAVIOUR RESEARCH AND THERAPY 77: 147-156. | wrong intervention | |
| 1424 | Kampmann, I. L., et al. (2018). "Self-report questionnaires, behavioral assessment tasks, and an implicit behavior measure: do they predict social anxiety in everyday life?" PEERJ 6. | wrong intervention | |
| 1425 | Kampmann, I. L., et al. (2019). "Cognitive predictors of treatment outcome for exposure therapy: do changes in self-efficacy, self-focused attention, and estimated social costs predict symptom improvement in social anxiety disorder?" BMC PSYCHIATRY 19. | wrong intervention | |
| 1426 | Kampmann, I. L., et al. (2020). "Social comparison modulates coping with fear in virtual environments." JOURNAL OF ANXIETY DISORDERS 72. | wrong intervention | |
| 1427 | Kanad, N., et al. (2024). "The effect of virtual reality on pain, fear and emotional appearance during blood draw in pediatric patients at the hematology-oncology outpatient clinic: A randomized controlled study." EUROPEAN JOURNAL OF ONCOLOGY NURSING 68. | wrong population | |
| 1428 | Kanai, M., et al. (2023). "A second update on mapping the human genetic architecture of COVID-19." Nature 621(7977): E7-E26. | wrong intervention | |
| 1429 | Kandi, S. and S. K. Chandaka (2021). "Management and Exploration of Physical & Mental Health Changes in Practitioners of Spirituality." INTERNATIONAL TRANSACTION JOURNAL OF ENGINEERING MANAGEMENT & APPLIED SCIENCES & TECHNOLOGIES 12(6). | wrong intervention | |
| 1430 | Kang, J. J., et al. (2019). "Voluntary wheel running activates Akt/AMPK/eNOS signaling cascades without improving profound endothelial dysfunction in mice deficient in α-galactosidase A." PLOS ONE 14(5). | wrong intervention | |
| 1431 | Kang, J. M., et al. (2021). "Effect of Cognitive Training in Fully Immersive Virtual Reality on Visuospatial Function and Frontal-Occipital Functional Connectivity in Predementia: Randomized Controlled Trial." JOURNAL OF MEDICAL INTERNET RESEARCH 23(5). | wrong population | |
| 1432 | Kang, J. M., et al. (2021). "Effect of cognitive training in fully immersive virtual reality on visuospatial function and frontal-occipital functional connectivity in predementia: Randomized controlled trial." Journal of Medical Internet Research 23(5): 18. | duplicate |  |
| 1433 | Kang, M. W. and I. J. Lee (2023). Applying Virtual Reality Technology and Physical Feedback on Aging in Spatial Orientation and Memory Ability. Lecture Notes in Computer Science (including subseries Lecture Notes in Artificial Intelligence and Lecture Notes in Bioinformatics). | wrong design | |
| 1434 | Kanyilmaz, T., et al. (2022). "Effectiveness of conventional versus virtual reality-based vestibular rehabilitation exercises in elderly patients with dizziness: a randomized controlled study with 6-month follow-up." BRAZILIAN JOURNAL OF OTORHINOLARYNGOLOGY 88: S41-S49. | wrong outcomes | |
| 1435 | Kanyılmaz, T., et al. (2022). "Effectiveness of conventional versus virtual reality-based vestibular rehabilitation exercises in elderly patients with dizziness: a randomized controlled study with 6-month follow-up." Braz J Otorhinolaryngol 88 Suppl 3(Suppl 3): S41-s49. | wrong outcomes | |
| 1436 | Kapikiran, G., et al. (2022). "The Effect of Video Training before Organ Transplant Surgery on Patient Satisfaction and Anxiety: Head Mounted Display Effect." CLINICAL SIMULATION IN NURSING 62: 99-106. | wrong intervention | |
| 1437 | Kaplan, E., et al. (2000). Chapter 21 SURGERY OF THE THYROID. Endotext. K. R. Feingold, B. Anawalt, M. R. Blackman et al. South Dartmouth (MA), MDText.com, Inc. | wrong intervention | |
| 1438 | Kappelmann, N., et al. (2020). "D-cycloserine as adjunct to brief computerised CBT for spider fear: Effects on fear, behaviour, and cognitive biases." JOURNAL OF BEHAVIOR THERAPY AND EXPERIMENTAL PSYCHIATRY 68. | wrong intervention | |
| 1439 | Karabulut, M., et al. (2010). "The effects of low-intensity resistance training with vascular restriction on leg muscle strength in older men." Eur J Appl Physiol 108(1): 147-155. | wrong intervention | |
| 1440 | Karageorgiou, Z., et al. (2021). Smart Escape Rooms for Cultural Heritage: A Systematic Review. PROCEEDINGS OF THE 15TH EUROPEAN CONFERENCE ON GAME BASED LEARNING (ECGBL 2021): 404-411. | wrong intervention | |
| 1441 | Karppa, E., et al. (2024). "Case Report: Virtual natural environment solution helped a child cope with a painful procedure." FRONTIERS IN PEDIATRICS 12. | wrong population | |
| 1442 | Karran, M., et al. (2019). "Recruitment of Older Veterans with Diabetes Risk for Alzheimer's Disease for a Randomized Clinical Trial of Computerized Cognitive Training." J Alzheimers Dis 69(2): 401-411. | wrong intervention | |
| 1443 | Karssemeijer, E. G. A., et al. (2019). "Exergaming as a Physical Exercise Strategy Reduces Frailty in People With Dementia: A Randomized Controlled Trial." J Am Med Dir Assoc 20(12): 1502-1508.e1501. | wrong intervention | |
| 1444 | Kase, N. G., et al. (2020). "The midlife transition and the risk of cardiovascular disease and cancer Part I: magnitude and mechanisms." Am J Obstet Gynecol 223(6): 820-833. | wrong intervention | |
| 1445 | Kasimoglu, Y., et al. (2020). "Robotic approach to the reduction of dental anxiety in children." ACTA ODONTOLOGICA SCANDINAVICA 78(6): 474-480. | wrong population | |
| 1446 | Kataoka, H., et al. (2017). "The influence of aging on the effectiveness of heat stress in preventing disuse muscle atrophy." PHYSIOLOGY INTERNATIONAL 104(4): 316-328. | wrong intervention | |
| 1447 | Katzman, M. A., et al. (2014). "Canadian clinical practice guidelines for the management of anxiety, posttraumatic stress and obsessive-compulsive disorders." BMC PSYCHIATRY 14. | wrong intervention | |
| 1448 | Kaufman, D., et al. (2015). "Aortic valvuloplasty in heyde's syndrome: Bridging the gap to improve bleeding outcomes." American Journal of Gastroenterology 110: S475. | wrong intervention | |
| 1449 | Kaur, P., et al. (2020). "Cost-utility analysis of hearing aid device for older adults in the community: a delayed start study." BMC Health Serv Res 20(1): 1112. | wrong intervention | |
| 1450 | Kaur, R., et al. (2018). Virtual Reality, Visual Cliffs, and Movement Disorders. 2018 40TH ANNUAL INTERNATIONAL CONFERENCE OF THE IEEE ENGINEERING IN MEDICINE AND BIOLOGY SOCIETY (EMBC): 81-84. | wrong outcomes | |
| 1451 | Kaur, R., et al. (2019). "Using Virtual Reality to Examine the Neural and Physiological Responses to Height and Perturbations in Quiet Standing." Annu Int Conf IEEE Eng Med Biol Soc 2019: 5233-5236. | wrong outcomes | |
| 1452 | Kaur, R., et al. (2019). Using Virtual Reality to Examine the Neural and Physiological Responses to Height and Perturbations in Quiet Standing. 2019 41ST ANNUAL INTERNATIONAL CONFERENCE OF THE IEEE ENGINEERING IN MEDICINE AND BIOLOGY SOCIETY (EMBC): 5233-5236. | wrong outcomes | |
| 1453 | Kavanagh, K., et al. (2016). "Effects of heated hydrotherapy on muscle HSP70 and glucose metabolism in old and young vervet monkeys." Cell Stress Chaperones 21(4): 717-725. | wrong population | |
| 1454 | Kavlakci, M., et al. (2023). "The effects of playing digital games on children's pain, fear, and anxiety levels during suturing: A randomized controlled study." TURKISH JOURNAL OF EMERGENCY MEDICINE 23(3): 162-168. | wrong population | |
| 1455 | Kavvadas, D., et al. (2023). "Stress, Anxiety, and Depression Levels among University Students: Three Years from the Beginning of the Pandemic." CLINICS AND PRACTICE 13(3): 596-609. | wrong population | |
| 1456 | Kawaguchi, R., et al. (2022). "Internal carotid artery paraclinoid aneurysms are frequently found in young female." Interventional Neuroradiology 28(1): 303-304. | wrong population | |
| 1457 | Kaya, C., et al. (2023). "The Impact of Work Incentives Benefits Counseling on Employment Outcomes: A National Vocational Rehabilitation Study." JOURNAL OF OCCUPATIONAL REHABILITATION 33(3): 538-549. | wrong outcomes | |
| 1458 | Kaya, C. and F. Chan (2017). "Vocational Rehabilitation Services and Outcomes for Working Age People with Depression and Other Mood Disorders." JOURNAL OF REHABILITATION 83(3): 44-52. | wrong intervention | |
| 1459 | Kaya, C., et al. (2022). "Demographic Covariates, Vocational Rehabilitation Services, and Employment Outcomes of Working-Age Adults with Anxiety Disorders: A Multivariate Logistic Regression Analysis." JOURNAL OF OCCUPATIONAL REHABILITATION 32(4): 743-752. | wrong outcomes | |
| 1460 | Kaya, C., et al. (2020). "Vocational rehabilitation factors associated with successful return to work outcomes for clients with Parkinson's disease." JOURNAL OF VOCATIONAL REHABILITATION 52(2): 145-156. | wrong intervention | |
| 1461 | Kaya, M. and Z. K. Özlü (2023). "The effect of virtual reality on pain, anxiety, and fear during burn dressing in children: A randomized controlled study." BURNS 49(4): 788-796. | wrong population | |
| 1462 | Kaya, M. D., et al. (2024). "Seed priming as a method of preservation and restoration of sunflower seeds." OCL-OILSEEDS AND FATS CROPS AND LIPIDS 31. | wrong intervention | |
| 1463 | Kayabinar, B., et al. (2021). "The effects of virtual reality augmented robot-assisted gait training on dual-task performance and functional measures in chronic stroke: a randomized controlled single-blind trial." EUROPEAN JOURNAL OF PHYSICAL AND REHABILITATION MEDICINE 57(2): 227-237. | wrong outcomes | |
| 1464 | Kazak, M., et al. (2020). "Marginal Microleakage of Composite Resin Materials Comprising Different Photo Initiators with Surface Sealants and Bonding Agent Application after Thermomechanical Aging." JOURNAL OF RESEARCH IN MEDICAL AND DENTAL SCIENCE 8(1): 16-25. | wrong intervention | |
| 1465 | Keatley, E. S., et al. (2023). "Cognitive Performance, Depression, and Anxiety 1 Year After Traumatic Brain Injury." J Head Trauma Rehabil 38(3): E195-e202. | wrong intervention | |
| 1466 | Keenan, B. and N. Wynne (2012). "Virtually perfect care with a click." Nursing Standard 26(19): 64-64. | wrong intervention | |
| 1467 | Kekäläinen, S., et al. (2023). "Effectiveness of Digital Counseling Before Pediatric Diagnostic Imaging." RADIOLOGIC TECHNOLOGY 94(3): 180-196. | wrong population | |
| 1468 | Kelleci, M. and S. İnal (2010). "Psychiatric symptoms in adolescents with Internet use: Comparison without Internet use." Cyberpsychology, Behavior, and Social Networking 13(2): 191-194. | wrong population | |
| 1469 | Keller, N., et al. (2022). "Virtual Reality "exergames": A promising countermeasure to improve motivation and restorative effects during long duration spaceflight missions." FRONTIERS IN PHYSIOLOGY 13. | wrong population | |
| 1470 | Kelly, N., et al. (2022). "Using a virtual reality cricket simulator to explore the effects of pressure, competition anxiety on batting performance in cricket." PSYCHOLOGY OF SPORT AND EXERCISE 63. | wrong population | |
| 1471 | Kendrick, D., et al. (2024). "Early vocational rehabilitation and psychological support for trauma patients to improve return to work (the ROWTATE trial): study protocol for an individually randomised controlled multicentre pragmatic trial." Trials 25(1): 439. | wrong population | |
| 1472 | Kennedy, C. R., et al. (2013). A Multiaxial Fatigue Damage Model for Glass Fibre Reinforced Polymer in Seawater. 9TH INTERNATIONAL CONFERENCE ON COMPOSITE SCIENCE AND TECHNOLOGY: 2020 - SCIENTIFIC AND INDUSTRIAL CHALLENGES: 1033-1033. | wrong intervention | |
| 1473 | Kenyon, K., et al. (2023). "Social virtual reality helps to reduce feelings of loneliness and social anxiety during the Covid-19 pandemic." SCIENTIFIC REPORTS 13(1). | wrong intervention | |
| 1474 | Kerklaan, J., et al. (2020). "Perspectives on life participation by young adults with chronic kidney disease: an interview study." BMJ OPEN 10(10). | wrong intervention | |
| 1475 | Keshvari, M., et al. (2021). "The effect of virtual reality distraction on reducing patients' anxiety before coronary angiography: a randomized clinical trial study." EGYPTIAN HEART JOURNAL 73(1). | wrong population | |
| 1476 | Kettlewell, J., et al. (2022). "Qualitative study exploring factors affecting the implementation of a vocational rehabilitation intervention in the UK major trauma pathway." BMJ Open 12(3): e060294. | wrong intervention | |
| 1477 | Khadra, C., et al. (2018). "Projector-based virtual reality dome environment for procedural pain and anxiety in young children with burn injuries: a pilot study." JOURNAL OF PAIN RESEARCH 11: 343-353. | wrong population | |
| 1478 | Khadra, C., et al. (2020). "Effects of a projector-based hybrid virtual reality on pain in young children with burn injuries during hydrotherapy sessions: A within-subject randomized crossover trial." BURNS 46(7): 1571-1584. | wrong population | |
| 1479 | Khalid, S., et al. (2018). "Biaryl scaffold-focused virtual screening for anti-aggregatory and neuroprotective effects in Alzheimer’s disease." BMC Neuroscience 19: 11. | wrong intervention | |
| 1480 | Khan, R. U., et al. (2022). "Perspective, Opportunities and Challenges in Using Fennel (<i>Foeniculum vulgare</i>) in Poultry Health and Production as an Eco-Friendly Alternative to Antibiotics: A Review." ANTIBIOTICS-BASEL 11(2). | wrong intervention | |
| 1481 | Khan, S., et al. (2023). "A modified mental state assessment tool for impact analysis of virtual reality-based therapeutic interventions in patients with cognitive impairment." DIGITAL HEALTH 9. | wrong intervention | |
| 1482 | Khanna, R. K., et al. (2022). "Long-term functional outcomes and vision-related quality of life after vitrectomy for epiretinal membrane: a prospective cohort study." SCIENTIFIC REPORTS 12(1). | wrong intervention | |
| 1483 | Khatutsky, G., et al. (2013). "Urinary incontinence, functional status, and health-related quality of life among Medicare beneficiaries enrolled in the program for all-inclusive care for the elderly and dual eligible demonstration special needs plans." J Ambul Care Manage 36(1): 35-49. | wrong intervention | |
| 1484 | Khazaal, W., et al. (2021). "Psychological Complications at 3 Months Following Stroke: Prevalence and Correlates Among Stroke Survivors in Lebanon." FRONTIERS IN PSYCHOLOGY 12. | wrong intervention | |
| 1485 | Khirallah Abd El Fatah, N., et al. (2024). "Effect of Immersive Virtual Reality Reminiscence versus Traditional Reminiscence Therapy on Cognitive Function and Psychological Well-being among Older Adults in Assisted Living Facilities: A randomized controlled trial." Geriatr Nurs 55: 191-203. | duplicate | |
| 1486 | Khorev, V., et al. (2024). "Review on the use of AI-based methods and tools for treating mental conditions and mental rehabilitation." EUROPEAN PHYSICAL JOURNAL-SPECIAL TOPICS. | wrong intervention | |
| 1487 | Khosravi, P., et al. (2016). "The impact of technology on older adults’ social isolation." Computers in Human Behavior 63: 594-603. | wrong intervention | |
| 1488 | Kidd, L. I., et al. (2012). "Effectiveness of a Second Life® Simulation as a Teaching Strategy for Undergraduate Mental Health Nursing Students." JOURNAL OF PSYCHOSOCIAL NURSING AND MENTAL HEALTH SERVICES 50(7): 28-37. | wrong intervention | |
| 1489 | Kikuchi, T. and Ieee (2014). Framework and Software to Build Large Scale Digital City for Virtual Walking/Cycling System. 2014 IEEE 3RD GLOBAL CONFERENCE ON CONSUMER ELECTRONICS (GCCE): 33-36. | wrong intervention | |
| 1490 | Kilbourne, A. M., et al. (2017). "Improving Physical Health in Patients With Chronic Mental Disorders: Twelve-Month Results From a Randomized Controlled Collaborative Care Trial." JOURNAL OF CLINICAL PSYCHIATRY 78(1): 129-137. | wrong intervention | |
| 1491 | Kilbourne, A. M., et al. (2014). "SMI Life Goals: Description of a randomized trial of a Collaborative Care Model to improve outcomes for persons with serious mental illness." CONTEMPORARY CLINICAL TRIALS 39(1): 74-85. | wrong intervention | |
| 1492 | Kiliç, Ü. and E. T. Büyük (2024). "The Effect of Using Virtual Reality During Burn Dressing on Pain, Anxiety and Fear Felt in Children: A Randomized Controlled Trial." JOURNAL OF BURN CARE & RESEARCH 45(4): 949-957. | wrong population | |
| 1493 | Kilic, U., et al. (2024). "The effect of VR headsets used during burn dressing in children on satisfaction and anxiety level of their parents." JOURNAL OF PEDIATRIC NURSING-NURSING CARE OF CHILDREN & FAMILIES 75: e152-e158. | wrong population | |
| 1494 | Kim, A., et al. (2017). "Walking in fully immersive virtual environments: an evaluation of potential adverse effects in older adults and individuals with Parkinson’s disease." Journal of NeuroEngineering and Rehabilitation 14(1). | wrong population | |
| 1495 | Kim, D. (2021). "Development and Effect of Virtual Reality Practice Program for Improving Practical Competency of Caregivers Specializing in Dementia." HEALTHCARE 9(10). | wrong design | |
| 1496 | Kim, D., et al. (2023). "Effect of Individual Virtual Reality Cognitive Training Programs on Cognitive Function and Depression in Middle-Aged Women: Randomized Controlled Trial." JMIR MENTAL HEALTH 10. | wrong population | |
| 1497 | Kim, D. A., et al. (2020). "A body bag can save your life: a novel method of cold water immersion for heat stroke treatment." J Am Coll Emerg Physicians Open 1(1): 49-52. | wrong intervention | |
| 1498 | Kim, D. H. and T. R. Billiar (2011). "Hypoxia Activates Toll-like Receptor 4 Signaling in Primary Mouse Hepatocytes Through the Receptor Clustering within Lipid Rafts." JOURNAL OF THE KOREAN SURGICAL SOCIETY 80(3): 194-203. | wrong intervention | |
| 1499 | Kim, D. J., et al. (2023). "Association between anxiety and skin conductance according to the intensity of shaking of virtual reality images." FRONTIERS IN PSYCHIATRY 14. | wrong intervention | |
| 1500 | Kim, D. R., et al. (2023). "Effect of Individual Virtual Reality Cognitive Training Programs on Cognitive Function and Depression in Middle-Aged Women: Randomized Controlled Trial." JMIR Ment Health 10: e48912. | wrong population | |
| 1501 | Kim, D.-R., et al. (2023). "Effect of individual virtual reality cognitive training programs on cognitive function and depression in middle-aged women: Randomized controlled trial." JMIR Mental Health 10: 11. | wrong population | |
| 1502 | Kim, G., et al. (2020). "Urban regeneration: Community engagement process for vacant land in declining cities." Cities 102. | wrong intervention | |
| 1503 | Kim, H., et al. (2021). "Clinical predictors of cybersickness in virtual reality (VR) among highly stressed people." SCIENTIFIC REPORTS 11(1). | wrong intervention | |
| 1504 | Kim, H., et al. (2021). "Effect of Virtual Reality on Stress Reduction and Change of Physiological Parameters Including Heart Rate Variability in People With High Stress: An Open Randomized Crossover Trial." FRONTIERS IN PSYCHIATRY 12. | wrong population | |
| 1505 | Kim, H. E., et al. (2017). "Effectiveness of self-training using the mobile-based virtual reality program in patients with social anxiety disorder." COMPUTERS IN HUMAN BEHAVIOR 73: 614-619. | wrong population | |
| 1506 | Kim, J., et al. (2022). "A Pilot Study of Virtual Reality (VR) Tai Chi Program on Mental Health among Older Adults during the COVID-19 Pandemic." Am J Health Behav 46(5): 576-585. | wrong design | |
| 1507 | Kim, J., et al. (2022). "A Pilot Study of Virtual Reality (VR) Tai Chi Program on Mental Health among Older Adults during the COVID-19 Pandemic." AMERICAN JOURNAL OF HEALTH BEHAVIOR 46(5): 576-585. | wrong design | |
| 1508 | Kim, J., et al. (2022). "A pilot study of virtual reality (VR) Tai Chi program on mental health among older adults during the COVID-19 pandemic." American Journal of Health Behavior 46(5): 576-585. | wrong design | |
| 1509 | Kim, J., et al. (2023). "Efficacy of An Immersive Nature-based Virtual Reality Program on Depression, Emotional Health, and Quality-of-Life among Care Facility Residents with Alzheimer's Disease (AD) and its Related Dementias (ADRD)." AMERICAN JOURNAL OF HEALTH BEHAVIOR 47(5): 1052-1061. | wrong outcomes | |
| 1510 | Kim, J., et al. (2023). "The Effects of a Nature-based Virtual Reality Program on Emotional Health and Quality of Life among Older Adults with Dementia." AMERICAN JOURNAL OF HEALTH BEHAVIOR 47(1): 3-12. | duplicate |  |
| 1511 | Kim, J. I. and G. Kim (2024). "Exploring Subjective Mental Health Change in Older Adults after Experiencing an Immersive Virtual Reality (IVR) -Based Natural Environment." JOURNAL OF APPLIED GERONTOLOGY. | wrong design | |
| 1512 | Kim, J. W., et al. (2019). "Vascular risk modulates the relationship between cerebral amyloid deposition and subjective memory complaints." NEUROPSYCHIATRIC DISEASE AND TREATMENT 15: 637-645. | wrong intervention | |
| 1513 | Kim, K., et al. (2015). "A Virtual Joy-Stick Study of Emotional Responses and Social Motivation in Children with Autism Spectrum Disorder." JOURNAL OF AUTISM AND DEVELOPMENTAL DISORDERS 45(12): 3891-3899. | wrong population | |
| 1514 | Kim, K. A. and J. A. Ahn (2024). "Effectiveness of Immersive Virtual Reality Simulation Programs Using Head-Mounted Displays in Promoting Physical Activity in Older Adults: A Systematic Review." Clinical Simulation in Nursing 94. | wrong design | |
| 1515 | Kim, K.-A. and J.-A. Ahn (2024). "Effectiveness of Immersive Virtual Reality Simulation Programs Using Head-Mounted Displays in Promoting Physical Activity in Older Adults: A Systematic Review." Clinical Simulation in Nursing 94: N.PAG-N.PAG. | wrong design | |
| 1516 | Kim, K. W., et al. (2023). "Development and preliminary validation of a virtual reality memory test for assessing visuospatial memory." Frontiers in Aging Neuroscience 15. | wrong intervention | |
| 1517 | Kim, K. W., et al. (2018). "Social Event Memory Test (SEMT): A Video-based Memory Test for Predicting Amyloid Positivity for Alzheimer's Disease." Scientific reports 8(1): 10421. | wrong intervention | |
| 1518 | Kim, S., et al. (2010). Reliability-based durability design and quality control of long span bridge of Busan-Geoje Fixed Link project in Korea. BRIDGE MAINTENANCE, SAFETY, MANAGEMENT AND LIFE-CYCLE OPTIMIZATION: 1823-1829. | wrong intervention | |
| 1519 | Kim, S. H. and S. H. Cho (2022). "Benefits of Virtual Reality Program and Motor Imagery Training on Balance and Fall Efficacy in Isolated Older Adults: A Randomized Controlled Trial." MEDICINA-LITHUANIA 58(11). | wrong intervention | |
| 1520 | Kim, S. H. and S. H. Cho (2022). "Virtual Reality Programs and Motor Imagery Training Reduce Plantar Pressure and Depression in Isolated Older Adults." INTERNATIONAL JOURNAL OF GERONTOLOGY 16(2): 100-105. | wrong intervention | |
| 1521 | Kim, W. H., et al. (2022). "Effectiveness of virtual reality exposure treatment for posttraumatic stress disorder due to motor vehicle or industrial accidents." VIRTUAL REALITY 26(4): 1539-1549. | wrong population | |
| 1522 | Kim, Y., et al. (2022). "Effects of Serious Games on Depression in Older Adults: Systematic Review and Meta-analysis of Randomized Controlled Trials." Journal of Medical Internet Research 24(9). | wrong intervention | |
| 1523 | Kim, Y., et al. (2022). "Effects of Serious Games on Depression in Older Adults: Systematic Review and Meta-analysis of Randomized Controlled Trials." JOURNAL OF MEDICAL INTERNET RESEARCH 24(9). | wrong intervention | |
| 1524 | Kim, Y. H., et al. (2015). "Evaluation of combined effects of ageing period and freezing rate on quality attributes of beef loins." Meat Sci 110: 40-45. | wrong intervention | |
| 1525 | Kim, Y. W., et al. (2022). "Optimization of tricuspid membrane mechanism for effectiveness and leaflet longevity through hemodynamic analysis." ENGINEERING APPLICATIONS OF COMPUTATIONAL FLUID MECHANICS 16(1): 1587-1600. | wrong intervention | |
| 1526 | Kimura, S., et al. (2021). "Changes of residual stresses on the surface of leucite-reinforced ceramic restoration luted with resin composite cements during aging in water." J Mech Behav Biomed Mater 123: 104711. | wrong intervention | |
| 1527 | Kimura, Y., et al. (2022). "Conservation paleobiology on Minami-Daito Island, Okinawa, Japan: anthropogenic extinction of cave-dwelling bats on a tropical oceanic island." PEERJ 10. | wrong intervention | |
| 1528 | King, D. L., et al. (2020). "Face validity evaluation of screening tools for gaming disorder: Scope, language, and overpathologizing issues." J Behav Addict 9(1): 1-13. | wrong intervention | |
| 1529 | King, K. E., et al. (2024). "Temperature-Dependent Relationship of Autophagy and Apoptotic Signaling During Cold-Water Immersion in Young and Older Males." Adv Biol (Weinh) 8(3): e2300560. | wrong intervention | |
| 1530 | King, M., et al. (2020). "Does training in the cold improve cold performance?" INTERNATIONAL JOURNAL OF INDUSTRIAL ERGONOMICS 76. | wrong intervention | |
| 1531 | Kiper, P., et al. (2022). "Effects of Immersive Virtual Therapy as a Method Supporting Recovery of Depressive Symptoms in Post-Stroke Rehabilitation: Randomized Controlled Trial." Clin Interv Aging 17: 1673-1685. |  |  |
| 1532 | Kirca, A. S. and D. K. Gül (2022). "The effect of music and skin contact with the newborn on pain and anxiety during episiotomy repair in primiparous women: A double-blind randomized controlled study." EXPLORE-THE JOURNAL OF SCIENCE AND HEALING 18(2): 210-216. | wrong intervention | |
| 1533 | Kirschstein, M. A., et al. (2023). "International Survey on the Use of Emerging Technologies Among Forensic and Correctional Mental Health Professionals." CRIMINAL JUSTICE AND BEHAVIOR 50(2): 175-196. | wrong intervention | |
| 1534 | Kirthika, S. K. and S. K. Singh (2020). "Durability studies on recycled fine aggregate concrete." CONSTRUCTION AND BUILDING MATERIALS 250. | wrong intervention | |
| 1535 | Kishishita, Y., et al. (2019). Muscleblazer: Force-Feedback Suit for Immersive Experience. 2019 26TH IEEE CONFERENCE ON VIRTUAL REALITY AND 3D USER INTERFACES (VR): 1813-1818. | wrong intervention | |
| 1536 | Kitson, A., et al. (2023). Co-Designing a Virtual Reality Intervention for Supporting Cognitive Reappraisal Skills Development with Youth. 22ND ANNUAL ACM INTERACTION DESIGN AND CHILDREN CONFERENCE, IDC 2023: Rediscovering Childhood: 14-26. | wrong outcomes | |
| 1537 | Kizony, R., et al. (2017). "Using virtual reality simulation to study navigation in a complex environment as a functional-cognitive task: A pilot study." Journal of Vestibular Research: Equilibrium & Orientation 27(1): 39-47. | wrong population | |
| 1538 | Kleffelgaard, I., et al. (2016). "Vestibular Rehabilitation After Traumatic Brain Injury: Case Series." PHYSICAL THERAPY 96(6): 839-849. | wrong outcomes | |
| 1539 | Kleim, B., et al. (2018). "Early Linguistic Markers of Trauma-Specific Processing Predict Post-trauma Adjustment." Front Psychiatry 9: 645. | wrong intervention | |
| 1540 | Kleiner, I., et al. (2024). "The use of virtual reality during extra-amniotic balloon insertion for pain and anxiety relief-a randomized controlled trial." AMERICAN JOURNAL OF OBSTETRICS & GYNECOLOGY MFM 6(1). | wrong population | |
| 1541 | Kleye, I., et al. (2022). "Children's communication of emotional cues and concerns during a preoperative needle procedure." PATIENT EDUCATION AND COUNSELING 105(6): 1518-1523. | wrong population | |
| 1542 | Klionsky, D. J., et al. (2021). "Guidelines for the use and interpretation of assays for monitoring autophagy (4th edition)(1)." Autophagy 17(1): 1-382. | wrong outcomes | |
| 1543 | Klompstra, L. V., et al. (2014). "Exergaming in older adults: A scoping review and implementation potential for patients with heart failure." EUROPEAN JOURNAL OF CARDIOVASCULAR NURSING 13(5): 388-398. | wrong intervention | |
| 1544 | Knaust, T., et al. (2022). "Exposure to virtual nature: the impact of different immersion levels on skin conductance level, heart rate, and perceived relaxation." VIRTUAL REALITY 26(3): 925-938. | wrong outcomes | |
| 1545 | Knaust, T., et al. (2022). "Nature videos for PTSD: protocol for a mixed-methods feasibility study." European journal of psychotraumatology 13(2): 2101765. | wrong intervention | |
| 1546 | Knowles, L. M., et al. (2017). "A pilot study of virtual support for grief: Feasibility, acceptability, and preliminary outcomes." COMPUTERS IN HUMAN BEHAVIOR 73: 650-658. | wrong population | |
| 1547 | Knowles, L. M., et al. (2017). "A pilot study of virtual support for grief: Feasibility, acceptability, and preliminary outcomes." Computers in Human Behavior 73: 650-658. | wrong population | |
| 1548 | Ko, D. H., et al. (2013). "FE-Simulation Coupled with CFD Analysis for Prediction of Residual Stresses Relieved by Cryogenic Heat treatment of Al6061 Tube." INTERNATIONAL JOURNAL OF PRECISION ENGINEERING AND MANUFACTURING 14(8): 1301-1309. | wrong intervention | |
| 1549 | Kokorelias, K. M., et al. (2024). "Use of Virtual Reality and Augmented Reality Technologies to Support Resilience and Skill Building in Caregivers of Persons With Dementia: A Scoping Review." CUREUS JOURNAL OF MEDICAL SCIENCE 16(7). | wrong outcomes | |
| 1550 | Kokorelias, K. M., et al. (2024). "Use of Virtual Reality and Augmented Reality Technologies to Support Resilience and Skill-Building in Caregivers of Persons With Dementia: A Scoping Review." Cureus 16(7): e64082. | wrong population | |
| 1551 | Komala, K., et al. (2013). "Comparative study of oral and vaginal misoprostol for induction of labour, maternal and foetal outcome." J Clin Diagn Res 7(12): 2866-2869. | wrong intervention | |
| 1552 | Komine, F., et al. (2012). "Effect of surface treatment on bond strength between an indirect composite material and a zirconia framework." J Oral Sci 54(1): 39-46. | wrong intervention | |
| 1553 | Koner, S., et al. (2021). "Assessment of Carbon Substrate Catabolism Pattern and Functional Metabolic Pathway for Microbiota of Limestone Caves." Microorganisms 9(8). | wrong intervention | |
| 1554 | Kong Saoane, T., et al. (2023). Key Considerations for The Design of Technology for Enrichment in Residential Aged Care: An Ethnographic Study. PROCEEDINGS OF THE 2023 CHI CONFERENCE ON HUMAN FACTORS IN COMPUTING SYSTEMS (CHI 2023). | wrong outcomes | |
| 1555 | Konishi, K., et al. (2017). "Hippocampus-dependent spatial learning is associated with higher global cognition among healthy older adults." Neuropsychologia 106: 310-321. | wrong intervention | |
| 1556 | Kono, E., et al. (2012). "Rating and issues of mechanical anastomotic staplers in surgical practice: a survey of 241 Japanese gastroenterological surgeons." SURGERY TODAY 42(10): 962-972. | wrong intervention | |
| 1557 | Kopels, M. C. and Ullah, II (2024). "Modeling post-Pleistocene megafauna extinctions as complex social-ecological systems." QUATERNARY RESEARCH. | wrong intervention | |
| 1558 | Korinek, K., et al. (2019). "Design and measurement in a study of war exposure, health, and aging: protocol for the Vietnam health and aging study." BMC Public Health 19(1): 1351. | wrong intervention | |
| 1559 | Korkmaz, E. and S. Guler (2023). "The effect of video streaming with virtual reality on anxiety and pain during bone marrow aspiration and biopsy procedure." Pain Management Nursing 24(6): 634-640. | wrong population | |
| 1560 | Kormendi, N. M. and A. D. Brown (2021). "Asian American mental health during COVID-19: A call for task-sharing interventions." SSM-MENTAL HEALTH 1. | wrong intervention | |
| 1561 | Koronczai, B., et al. (2013). "The mediating effect of self-esteem, depression and anxiety between satisfaction with body appearance and problematic internet use." AMERICAN JOURNAL OF DRUG AND ALCOHOL ABUSE 39(4): 259-265. | wrong intervention | |
| 1562 | Korsvold, L., et al. (2017). "A content analysis of emotional concerns expressed at the time of receiving a cancer diagnosis: An observational study of consultations with adolescent and young adult patients and their family members." EUROPEAN JOURNAL OF ONCOLOGY NURSING 26: 1-8. | wrong intervention | |
| 1563 | Kosti, M. V., et al. (2023). "Assessing Virtual Reality Spaces for Elders Using Image-Based Sentiment Analysis and Stress Level Detection." SENSORS 23(8). | wrong intervention | |
| 1564 | Kot, J. (2023). "When is a diver considered a sportsman?" European Journal of Translational and Clinical Medicine 6: 106. | wrong intervention | |
| 1565 | Kothgassner, O. D. and A. Felnhofer (2021). "Lack of research on efficacy of virtual reality exposure therapy (VRET) for anxiety disorders in children and adolescents A systematic review." NEUROPSYCHIATRIE 35(2): 68-75. | wrong population | |
| 1566 | Kouhi Fayegh, A., et al. (2023). "Relationship of sleep quality, chronotype, and obstructive sleep apnea with migraine in the elderly population." Neuropsychiatr. | wrong intervention | |
| 1567 | Koushede, V., et al. (2010). "Use of over-the-counter analgesics and perceived stress among 25-44-year olds." PHARMACOEPIDEMIOLOGY AND DRUG SAFETY 19(4): 351-357. | wrong population | |
| 1568 | Koushede, V., et al. (2010). "Use of over-the-counter analgesics is associated with perceived stress among 25-44-year-olds: A national cross-sectional study." Scandinavian Journal of Public Health 38(5): 474-480. | wrong population | |
| 1569 | Kovács, E., et al. (2014). CHALLENGES OF VIRTUAL ENVIRONMENT IN EDUCATION. EDULEARN14: 6TH INTERNATIONAL CONFERENCE ON EDUCATION AND NEW LEARNING TECHNOLOGIES: 179-185. | wrong intervention | |
| 1570 | Koyagasioglu, O. and C. Özgürbüz (2022). "The Effects of Virtual Reality Nonphysical Mental Training on Coordination and Skill Transfer in Healthy Adults." JOURNAL OF SPORT REHABILITATION 31(4): 442-451. | wrong population | |
| 1571 | Köyağasıoğlu, O., et al. (2022). "The Effects of Virtual Reality Nonphysical Mental Training on Balance Skills and Functional Near-Infrared Spectroscopy Activity in Healthy Adults." J Sport Rehabil 31(4): 428-441. | wrong population | |
| 1572 | Köyaǧasioǧlu, O., et al. (2022). "The Effects of Virtual Reality Nonphysical Mental Training on Balance Skills and Functional Near-Infrared Spectroscopy Activity in Healthy Adults." Journal of Sport Rehabilitation 31(4): 428-441. | duplicate |  |
| 1573 | Köyağasıoğlu, O., et al. (2022). "The Effects of Virtual Reality Nonphysical Mental Training on Balance Skills and Functional Near-Infrared Spectroscopy Activity in Healthy Adults." Journal of sport rehabilitation 31(4): 428-441. | duplicate |  |
| 1574 | Koyu, H. O. and E. K. Törüner (2023). "The effect of technology-based interventions on child and parent outcomes in pediatric oncology: A systemic review of experimental evidence." ASIA-PACIFIC JOURNAL OF ONCOLOGY NURSING 10(5). | wrong population | |
| 1575 | Kozowyk, P. R. B. and J. A. Poulis (2019). "A new experimental methodology for assessing adhesive properties shows that Neandertals used the most suitable material available." JOURNAL OF HUMAN EVOLUTION 137. | wrong intervention | |
| 1576 | Krause-Parello, C. A., et al. (2019). "Loneliness, Depression, and Physical Activity in Older Adults: The Therapeutic Role of Human-Animal Interactions." ANTHROZOOS 32(2): 239-254. | wrong intervention | |
| 1577 | Krautz, R., et al. (2020). "Tissue-autonomous immune response regulates stress signaling during hypertrophy." Elife 9. | wrong intervention | |
| 1578 | Kremen, W. S., et al. (2014). "Genetic complexity of episodic memory: A twin approach to studies of aging." Psychology and Aging 29(2): 404-417. | wrong intervention | |
| 1579 | Krishnamurthy, A., et al. (2017). "Enhanced durability of carbon nanotube grafted hierarchical ceramic microfiber-reinforced epoxy composites." Carbon N Y 125: 63-75. | wrong intervention | |
| 1580 | Kristiansen, L., et al. (2019). "Feasibility of integrating vestibular rehabilitation and cognitive behaviour therapy for people with persistent dizziness." PILOT AND FEASIBILITY STUDIES 5(1). | wrong intervention | |
| 1581 | Kritikos, J., et al. (2021). "Personalized Virtual Reality Human-Computer Interaction for Psychiatric and Neurological Illnesses: A Dynamically Adaptive Virtual Reality Environment That Changes According to Real-Time Feedback From Electrophysiological Signal Responses." FRONTIERS IN HUMAN NEUROSCIENCE 15. | wrong population | |
| 1582 | Kron, F. W., et al. (2010). "Medical student attitudes toward video games and related new media technologies in medical education." BMC MEDICAL EDUCATION 10. | wrong intervention | |
| 1583 | Krüger, M., et al. (2020). "Impact of Acoustic and Interactive Disruptive Factors during Robot-Assisted Surgery-A Virtual Surgical Training Model." SENSORS 20(20). | wrong intervention | |
| 1584 | Krupic, D., et al. (2021). "Anxiety and threat magnification in subjective and physiological responses of fear of heights induced by virtual reality." PERSONALITY AND INDIVIDUAL DIFFERENCES 169. | wrong intervention | |
| 1585 | Krupp, R., et al. (2023). "Interpositional scaffold anchor rotator cuff footprint tear repair: excellent survival, healing, and early outcomes." KNEE SURGERY SPORTS TRAUMATOLOGY ARTHROSCOPY 31(7): 2670-2680. | wrong intervention | |
| 1586 | Kruse, C. S., et al. (2022). "Leveraging mHealth and Virtual Reality to Improve Cognition for Alzheimer's Patients: A Systematic Review." HEALTHCARE 10(10). | wrong design | |
| 1587 | Krysta, K., et al. (2016). "IMPLEMENTATION OF THE MOTEK CAREN SYSTEM IN BEHAVIOURAL THERAPY FOR PATIENTS WITH ANXIETY DISORDERS." PSYCHIATRIA DANUBINA 28: S116-S120. | wrong intervention | |
| 1588 | Ku, J., et al. (2019). "Three-dimensional augmented reality system for balance and mobility rehabilitation in the elderly: A randomized controlled trial." Cyberpsychology, Behavior, and Social Networking 22(2): 132-141. | wrong intervention | |
| 1589 | Kuijsters, A., et al. (2015). "Lighting to Make You Feel Better: Improving the Mood of Elderly People with Affective Ambiences." PLOS ONE 10(7). | wrong intervention | |
| 1590 | Kumar, D., et al. (2022). "Mapping out the glassy landscape of a mesoscopic elastoplastic model." JOURNAL OF CHEMICAL PHYSICS 157(17). | wrong intervention | |
| 1591 | Kumar, V., et al. (2020). "Tension Pneumocephalus in a Tracheostomized, Chronically Ventilated, Duchenne's Muscular Dystrophy Patient Without Prior Head Trauma." CUREUS JOURNAL OF MEDICAL SCIENCE 12(9). | wrong intervention | |
| 1592 | Kupczik, L., et al. (2022). "Appraising Virtual Technologies' Impact on Older Citizens' Mental Health-A Comparative between 360° Video and Virtual Reality." INTERNATIONAL JOURNAL OF ENVIRONMENTAL RESEARCH AND PUBLIC HEALTH 19(18). | wrong design | |
| 1593 | Küpper, C., et al. (2020). "Identifying predictive features of autism spectrum disorders in a clinical sample of adolescents and adults using machine learning." Sci Rep 10(1): 4805. | wrong population | |
| 1594 | Kuscu, H. Y. (2024). "The Effect of Different Ageing Protocols on the Shear Bond Strength of the Ceromer Indirect Composite on Two Different Substructure Materials." Niger J Clin Pract 27(3): 368-375. | wrong intervention | |
| 1595 | Kuzmina, L. Y., et al. (2022). "Characterization of the Novel Plant Growth- Stimulating Strain <i>Advenella kashmirensis</i> IB-K1 and Evaluation of Its Efficiency in Saline Soil." MICROBIOLOGY 91(2): 173-183. | wrong intervention | |
| 1596 | Kvam, K. and S. Karlsson (2013). "Solubility and strength of zirconia-based dental materials after artificial aging." J Prosthet Dent 110(4): 281-287. | wrong intervention | |
| 1597 | Kviatkovsky, S. A., et al. (2023). "Collagen peptides supplementation improves function, pain, and physical and mental outcomes in active adults." JOURNAL OF THE INTERNATIONAL SOCIETY OF SPORTS NUTRITION 20(1). | wrong intervention | |
| 1598 | Kwan, R. Y. C., et al. (2021). "Feasibility and effects of virtual reality motor-cognitive training in community-dwelling older people with cognitive frailty: Pilot randomized controlled trial." JMIR Serious Games 9(3): 1-14. | wrong outcomes | |
| 1599 | Kwan, R. Y. C., et al. (2023). "The effects of therapeutic virtual reality experience to promote mental well-being in older people living with physical disabilities in long-term care facilities." TRIALS 24(1). | wrong design | |
| 1600 | Kwee-Meier, S. T., et al. (2017). "Age-related differences in decision-making for digital escape route signage under strenuous emergency conditions of tilted passenger ships." APPLIED ERGONOMICS 59: 264-273. | wrong intervention | |
| 1601 | Kwon, J. H., et al. (2020). "Feasibility of the Virtual Reality Program in Managing Test Anxiety: A Pilot Study." CYBERPSYCHOLOGY BEHAVIOR AND SOCIAL NETWORKING 23(10): 715-720. | wrong population | |
| 1602 | Kyriazis, M. (2017). "Biological ageing and clinical consequences of modern technology." Biogerontology 18(4): 711-715. | wrong intervention | |
| 1603 | Kyritsi, E. M., et al. (2000). Familial or Sporadic Adrenal Hypoplasia Syndromes. Endotext. K. R. Feingold, B. Anawalt, M. R. Blackman et al. South Dartmouth (MA), MDText.com, Inc. | wrong intervention | |
| 1604 | La Corte, V., et al. (2021). "The role of semantic memory in prospective memory and episodic future thinking: new insights from a case of semantic dementia." MEMORY 29(8): 943-962. | wrong intervention | |
| 1605 | La Rocca, S., et al. (2020). "No country for old men: Reducing age bias through virtual reality embodiment." Annual Review of CyberTherapy and Telemedicine 18: 127-131. | wrong design | |
| 1606 | Laaksonen, M., et al. (2020). "Individual- and company-level predictors of receiving vocational rehabilitation: A multilevel study of Finnish private sector workplaces." Journal of Occupational Rehabilitation 30(2): 263-273. | wrong intervention | |
| 1607 | Lacey, C., et al. (2023). "oVRcome - Self-guided virtual reality for specific phobias: A randomised controlled trial." AUSTRALIAN AND NEW ZEALAND JOURNAL OF PSYCHIATRY 57(5): 736-744. | wrong population | |
| 1608 | Lachkar, S., et al. (2022). "Hypnosis associated with 3D immersive virtual reality technology during bronchoscopy under local anesthesia." JOURNAL OF THORACIC DISEASE 14(9): 3205-3210. | wrong intervention | |
| 1609 | Lacolley, P., et al. (2020). "Mechanisms of Arterial Stiffening: From Mechanotransduction to Epigenetics." Arterioscler Thromb Vasc Biol 40(5): 1055-1062. | wrong intervention | |
| 1610 | Lacombre, C. V., et al. (2018). "Influence of internal stresses on the physicochemical and mechanical properties evolution of pigmented epoxy systems during hygrothermal ageing." SURFACE & COATINGS TECHNOLOGY 341: 86-94. | wrong intervention | |
| 1611 | Lacombre, C. V., et al. (2017). "Influence of pigment on the degradation of anticorrosion polymer coatings using a thermodynamic analysis of electrochemical impedance spectroscopy data." ELECTROCHIMICA ACTA 234: 7-15. | wrong intervention | |
| 1612 | Lacoste-Ferré, M. H., et al. (2023). "Viscoelastic behavior of oral mucosa. A rheological study using small-amplitude oscillatory shear tests." J Mech Behav Biomed Mater 143: 105898. | wrong intervention | |
| 1613 | Ladyka-Wojcik, N., et al. (2021). "Flexible Use of Spatial Frames of Reference for Object-Location Memory in Older Adults." BRAIN SCIENCES 11(11). | wrong intervention | |
| 1614 | Laghlam, D., et al. (2021). "Virtual reality vs. Kalinox® for management of pain in intensive care unit after cardiac surgery: a randomized study." ANNALS OF INTENSIVE CARE 11(1). | wrong population | |
| 1615 | Lahti, S., et al. (2020). "Virtual Reality Relaxation to Decrease Dental Anxiety: Immediate Effect Randomized Clinical Trial." JDR CLINICAL & TRANSLATIONAL RESEARCH 5(4): 312-318. | wrong population | |
| 1616 | Lai, B., et al. (2020). "Feasibility of a Commercially Available Virtual Reality System to Achieve Exercise Guidelines in Youth With Spina Bifida: Mixed Methods Case Study." JMIR SERIOUS GAMES 8(4): 304-319. | wrong population | |
| 1617 | Lai, B., et al. (2023). "Improving Social Isolation and Loneliness Among Adolescents With Physical Disabilities Through Group-Based Virtual Reality Gaming: Feasibility Pre-Post Trial Study." JMIR FORMATIVE RESEARCH 7. | wrong population | |
| 1618 | Lai, X. J., et al. (2019). Can Virtual Reality Satisfy Entertainment Needs of the Elderly? The Application of a VR Headset in Elderly Care. CROSS-CULTURAL DESIGN-CULTURE AND SOCIETY, CCD 2019, PT II. 11577: 159-172. | wrong design | |
| 1619 | Laine, J., et al. (2023). "Primary school students' experiences of immersive virtual reality use in the classroom." COGENT EDUCATION 10(1). | wrong population | |
| 1620 | Lakman, I. A., et al. (2023). "QUALITY OF HEALTH OF HOSPITALIZED COVID-19 PATIENTS IN THE POST-COVID PERIOD: TWO-YEAR FOLLOW-UP STUDY." YAKUT MEDICAL JOURNAL(4): 77-82. | wrong intervention | |
| 1621 | Lall, A. C., et al. (2020). "Effect of alcohol consumption on patient-reported outcomes in hip arthroscopy: a matched controlled study with minimum 2-year follow-up." HIP INTERNATIONAL 30(4): 457-468. | wrong intervention | |
| 1622 | Lamb, R. and E. A. Etopio (2020). "Virtual Reality: a Tool for Preservice Science Teachers to Put Theory into Practice." JOURNAL OF SCIENCE EDUCATION AND TECHNOLOGY 29(4): 573-585. | wrong population | |
| 1623 | Lamb, R., et al. (2024). "Machine learning prediction of mental health strategy selection in school aged children using neurocognitive data." COMPUTERS IN HUMAN BEHAVIOR 156. | wrong population | |
| 1624 | Lambert, V., et al. (2020). "Virtual reality distraction for acute pain in children." COCHRANE DATABASE OF SYSTEMATIC REVIEWS(10). | wrong population | |
| 1625 | Lamblin, G., et al. (2020). "Virtual reality simulation to enhance laparoscopic salpingectomy skills." JOURNAL OF GYNECOLOGY OBSTETRICS AND HUMAN REPRODUCTION 49(3). | wrong population | |
| 1626 | Lan, C. M., et al. (2023). "Monitoring of soil water content using spherical smart aggregates based on electromechanical impedance (EMI) technique." SMART MATERIALS AND STRUCTURES 32(7). | wrong intervention | |
| 1627 | Lander, R., et al. (2020). "Executive functioning and spatial processing in anorexia nervosa: an experimental study and its significance for the allocentric lock theory." EATING AND WEIGHT DISORDERS-STUDIES ON ANOREXIA BULIMIA AND OBESITY 25(4): 1039-1047. | wrong intervention | |
| 1628 | Lane, C., et al. (2023). "Personalised interventions for subgroups of children with conduct problems." Cochrane Database Syst Rev 4(4): Cd012746. | wrong population | |
| 1629 | Langen, K., et al. (2017). "Effects of ageing and inbreeding on the reproductive traits in a cichlid fish II: the female perspective." BIOLOGICAL JOURNAL OF THE LINNEAN SOCIETY 120(4): 762-770. | wrong intervention | |
| 1630 | Langer, A. I., et al. (2016). "Substance Use, Bullying, and Body Image Disturbances in Adolescents and Young Adults Under the Prism of a 3D Simulation Program: Validation of MySchool4web." TELEMEDICINE AND E-HEALTH 22(1): 18-30. | wrong population | |
| 1631 | Langi, F., et al. (2017). "Vocational Rehabilitation of Transition-Age Youth with Disabilities: A Propensity-Score Matched Study." JOURNAL OF OCCUPATIONAL REHABILITATION 27(1): 15-23. | wrong intervention | |
| 1632 | Langlet, B. S., et al. (2021). "Virtual Reality App for Treating Eating Behavior in Eating Disorders: Development and Usability Study." JMIR SERIOUS GAMES 9(2). | wrong population | |
| 1633 | Langley, M. C. and T. Suddendorf (2020). "Mobile containers in human cognitive evolution studies: Understudied and underrepresented." EVOLUTIONARY ANTHROPOLOGY 29(6): 299-309. | wrong intervention | |
| 1634 | Lanham, N. S., et al. (2023). "Does the timing of tenotomy during biceps tenodesis affect the incidence of Popeye deformity and clinical outcome? An analysis of short-term follow-up of 2 techniques." JOURNAL OF SHOULDER AND ELBOW SURGERY 32(5): 917-923. | wrong intervention | |
| 1635 | Lanz, T. A., et al. (2019). "Postmortem transcriptional profiling reveals widespread increase in inflammation in schizophrenia: a comparison of prefrontal cortex, striatum, and hippocampus among matched tetrads of controls with subjects diagnosed with schizophrenia, bipolar or major depressive disorder." Transl Psychiatry 9(1): 151. | wrong intervention | |
| 1636 | Lanzieri, N., et al. (2023). "A VR client simulation to prepare MSW social work students for practicum: A feasibility study." Journal of Technology in Human Services 41(3): 230-258. | wrong population | |
| 1637 | Lapi, F., et al. (2024). "Development and validation of a prediction score to assess the risk of depression in primary care." J Affect Disord 355: 363-370. | wrong intervention | |
| 1638 | Lara, E., et al. (2020). "Understanding the multi-dimensional mental well-being in late life: Evidence from the perspective of the oldest old population." Journal of Happiness Studies: An Interdisciplinary Forum on Subjective Well-Being 21(2): 465-484. | wrong intervention | |
| 1639 | Larentis, O., et al. (2023). "Osteological Evidence of Possible Tuberculosis from the Early Medieval Age (6th-11th Century), Northern Italy." HERITAGE 6(7): 4886-4900. | wrong intervention | |
| 1640 | Latgé-Tovar, S., et al. (2024). "The use of virtual reality as a perspective-taking manipulation to improve self-awareness in Alzheimer’s disease." Frontiers in Aging Neuroscience 16. | wrong outcomes | |
| 1641 | Lau, H. M., et al. (2017). "Serious Games for Mental Health: Are They Accessible, Feasible, and effective? A Systematic Review and Meta-analysis." FRONTIERS IN PSYCHIATRY 7. | wrong intervention | |
| 1642 | Lau, J. S. Y., et al. (2024). "Development and Usability Testing of Virtual Reality (VR)-Based Reminiscence Therapy for People with Dementia." INFORMATION SYSTEMS FRONTIERS. | wrong population | |
| 1643 | Lau, Y. Y., et al. (2020). "THE DEPLOYMENT OF VIRTUAL REALITY (VR) TO PROMOTE GREEN BURIAL." ASIA PACIFIC JOURNAL OF HEALTH MANAGEMENT 15(2). | wrong outcomes | |
| 1644 | Lauffenburger, J. C., et al. (2022). "Prescribing decision making by medical residents on night shifts: A qualitative study." Med Educ 56(10): 1032-1041. | wrong intervention | |
| 1645 | Lauffenburger, J. C., et al. (2022). "Overcoming Decisional Gaps in High-Risk Prescribing by Junior Physicians Using Simulation-Based Training: Protocol for a Randomized Controlled Trial." JMIR RESEARCH PROTOCOLS 11(4). | wrong intervention | |
| 1646 | Lauffenburger, J. C., et al. (2024). "Pragmatic trial evaluating the impact of simulation training on high-risk prescribing to older adults by junior physicians." JOURNAL OF THE AMERICAN GERIATRICS SOCIETY 72(5): 1420-1430. | wrong intervention | |
| 1647 | Lauffenburger, J. C., et al. (2024). "Pragmatic trial evaluating the impact of simulation training on high‐risk prescribing to older adults by junior physicians." Journal of the American Geriatrics Society 72(5): 1420-1430. | wrong intervention | |
| 1648 | Laurence, B. D. and L. Michel (2017). "The Fall in Older Adults: Physical and Cognitive Problems." Curr Aging Sci 10(3): 185-200. | wrong intervention | |
| 1649 | Lavenex, P. B. and P. Lavenex (2021). "A Critical Review of Spatial Abilities in Down and Williams Syndromes: Not All Space Is Created Equal." FRONTIERS IN PSYCHIATRY 12. | wrong intervention | |
| 1650 | Law, E. F., et al. (2011). "Videogame Distraction using Virtual Reality Technology for Children Experiencing Cold Pressor Pain: The Role of Cognitive Processing." JOURNAL OF PEDIATRIC PSYCHOLOGY 36(1): 84-94. | wrong population | |
| 1651 | Lazarczyk, M. J., et al. (2016). "Major Histocompatibility Complex class I proteins are critical for maintaining neuronal structural complexity in the aging brain." Sci Rep 6: 26199. | wrong population | |
| 1652 | Lazaridis, G., et al. (2024). "Evaluating the Relation of Cave Passage Formation to Stress-Field: Spatio-Temporal Correlation of Speleogenesis with Active Tectonics in Asprorema Cave (Mt. Pinovo, Greece)." GEOSCIENCES 14(5). | wrong population | |
| 1653 | Lazarus, M. E., et al. (2023). "A case study of a unique advanced clinical skills elective at the David Geffen School of Medicine at UCLA." MedEdPublish (2016) 13: 1. | wrong population | |
| 1654 | Le Du, K., et al. (2023). "A New Option for Pain Prevention Using a Therapeutic Virtual Reality Solution for Bone Marrow Biopsy (REVEH Trial): Open-Label, Randomized, Multicenter, Phase 3 Study." Journal of Medical Internet Research 25. | wrong population | |
| 1655 | Le Guen-Geffroy, A., et al. (2019). "Physical ageing of epoxy in a wet environment: Coupling between plasticization and physical ageing." POLYMER DEGRADATION AND STABILITY 168. | wrong population | |
| 1656 | Le May, S., et al. (2022). "The Efficacy of Virtual Reality Game Preparation for Children Scheduled for Magnetic Resonance Imaging Procedures (IMAGINE): Protocol for a Randomized Controlled Trial." JMIR RESEARCH PROTOCOLS 11(6). | wrong population | |
| 1657 | Le May, S., et al. (2021). "Decreasing Pain and Fear in Medical Procedures with a Pediatric Population (DREAM): A Pilot Randomized Within-Subject Trial." PAIN MANAGEMENT NURSING 22(2): 191-197. | wrong population | |
| 1658 | Le May, S., et al. (2021). "Immersive virtual reality vs. non-immersive distraction for pain management of children during bone pins and sutures removal: A randomized clinical trial protocol." JOURNAL OF ADVANCED NURSING 77(1): 439-447. | wrong population | |
| 1659 | Le Roy, B., et al. (2024). "Virtual Exercise in Medicine: A Proof of Concept in a Healthy Population." JMIR FORMATIVE RESEARCH 8. | wrong population | |
| 1660 | Lebedeva, A. V., et al. (2024). "A Method for Assessing Working Memory in Rats Using Controlled Virtual Environment." SOVREMENNYE TEHNOLOGII V MEDICINE 16(3): 12-22. | wrong population | |
| 1661 | Lecouvey, G., et al. (2019). "An impairment of prospective memory in mild Alzheimer’s disease: A ride in a virtual town." Frontiers in Psychology 10: 12. | wrong design | |
| 1662 | Lee, A., et al. (2022). "Development of usability evaluation indicator for a rehabilitation exercise enhancement device using cognitive-physical combined intervention among the elderly...International Society for Gerontechnology 13th World Conference, October 24-26, 2022, Daegu, South Korea." Gerontechnology 21: 1-1. | wrong outcomes | |
| 1663 | Lee, A. R. and L. Hon (2022). "The Effects of Age-Morphing Technology on Older Adult Issue Campaigns: The Interplay of Construal Level, Perceived Probability and Message Appeal." CYBERPSYCHOLOGY-JOURNAL OF PSYCHOSOCIAL RESEARCH ON CYBERSPACE 16(2). | wrong population | |
| 1664 | Lee, C. S., et al. (2024). Testing Anchors, User Experience and Usability Among 4 Game Elements in a Kindy Immersive Augmented Reality Game. COMPUTATIONAL SCIENCE AND ITS APPLICATIONS-ICCSA 2024 WORKSHOPS, PT XI. 14825: 154-167. | wrong population | |
| 1665 | Lee, E., et al. (2024). "Racial and Ethnic Variations in Pre-Diagnosis Comorbidity Burden and Health-Related Quality of Life Among Older Women with Breast Cancer." J Racial Ethn Health Disparities 11(3): 1587-1599. | wrong intervention | |
| 1666 | Lee, E. J. and S. J. Park (2020). "A Framework of Smart-Home Service for Elderly's Biophilic Experience." SUSTAINABILITY 12(20). | wrong intervention | |
| 1667 | Lee, E. J. and S. J. Park (2021). "A Preference-Driven Smart Home Service for the Elderly's Biophilic Experience." Sensors (Basel) 21(15). | wrong population | |
| 1668 | Lee, E. K. Y., et al. (2024). "Racial and Ethnic Variations in Pre-Diagnosis Comorbidity Burden and Health-Related Quality of Life Among Older Women with Breast Cancer." JOURNAL OF RACIAL AND ETHNIC HEALTH DISPARITIES 11(3): 1587-1599. | wrong intervention | |
| 1669 | Lee, H., et al. (2022). "Real-time realizable mobile imaging photoplethysmography." SCIENTIFIC REPORTS 12(1). | wrong population | |
| 1670 | Lee, H. J. and D. K. Lee (2019). "Do Sociodemographic Factors and Urban Green Space Affect Mental Health Outcomes Among the Urban Elderly Population?" INTERNATIONAL JOURNAL OF ENVIRONMENTAL RESEARCH AND PUBLIC HEALTH 16(5). | wrong intervention | |
| 1671 | Lee, H. N., et al. (2021). "Virtual reality environment using a dome screen for procedural pain in young children during intravenous placement: A pilot randomized controlled trial." PLOS ONE 16(8). | wrong population | |
| 1672 | Lee, H. N., et al. (2023). "Effect of a Virtual Reality Environment Using a Domed Ceiling Screen on Procedural Pain During Intravenous Placement in Young Children A Randomized Clinical Trial." JAMA PEDIATRICS 177(1): 25-31. | wrong population | |
| 1673 | Lee, I. H., et al. (2022). "The acclimatization of Haenyeo to a cold environment and occupational characteristics evaluated by orexin and irisin levels." ANNALS OF OCCUPATIONAL AND ENVIRONMENTAL MEDICINE 34: 1-12. | wrong population | |
| 1674 | Lee, J. Y., et al. (2012). "Laparoscopic Warm-up Exercises Improve Performance of Senior-Level Trainees During Laparoscopic Renal Surgery." JOURNAL OF ENDOUROLOGY 26(5): 545-550. | wrong population | |
| 1675 | Lee, K. J., et al. (2022). "Physical fitness changes in adolescents due to social distancing during the coronavirus disease pandemic in Korea." PEERJ 10. | wrong population | |
| 1676 | Lee, K. N., et al. (2023). "Effects of Fetal Images Produced in Virtual Reality on Maternal-Fetal Attachment: Randomized Controlled Trial." JOURNAL OF MEDICAL INTERNET RESEARCH 25. | wrong population | |
| 1677 | Lee, L. N., et al. (2019). "Potential of Augmented Reality and Virtual Reality Technologies to Promote Wellbeing in Older Adults." APPLIED SCIENCES-BASEL 9(17). | wrong design | |
| 1678 | Lee, M., et al. (2024). "Impact of Customized Content in 3D Virtual Reality Motionless Imagery Exercise through Avatar on Emotional Well-Being, Cognition, and Physiological Response." APPLIED SCIENCES-BASEL 14(7). | wrong population | |
| 1679 | Lee, M., et al. (2016). "Comparison of individualized virtual reality-and group-based rehabilitation in older adults with chronic stroke in community settings: a pilot randomized controlled trial." EUROPEAN JOURNAL OF INTEGRATIVE MEDICINE 8(5): 738-746. | wrong intervention | |
| 1680 | Lee, M., et al. (2015). "Individualized feedback-based virtual reality exercise improves older women’s self-perceived health: A randomized controlled trial." Archives of Gerontology and Geriatrics 61(2): 154-160. | wrong intervention | |
| 1681 | Lee, M. A. and J. H. Kang (2023). "Does having children matter? Associations between transitions in work-family role combinations and depressive symptoms among married women in Korea." SSM-POPULATION HEALTH 22. | wrong population | |
| 1682 | Lee, S. H., et al. (2010). "Assessments of Low-Temperature Aging Test Method for the Dielectric Materials Immersed in Liquid Nitrogen." IEEE TRANSACTIONS ON APPLIED SUPERCONDUCTIVITY 20(3): 1654-1657. | wrong population | |
| 1683 | Lee, S. H. Y., et al. (2024). "Life and Death: A Multicenter Study Evaluating Cardiologists' Approach to Difficult Conversations with Fontan Patients and Families." PEDIATRIC CARDIOLOGY. | wrong population | |
| 1684 | Lee, Y., et al. (2024). "Measurement of Empathy in Virtual Reality with Head-Mounted Displays: A Systematic Review." IEEE TRANSACTIONS ON VISUALIZATION AND COMPUTER GRAPHICS 30(5): 2485-2495. | wrong population | |
| 1685 | Lee, Y. S. and J. H. Huh (2024). "Enabling Physical Activity with Augmented Reality Gamification for Reducing Internet Gaming Disorder." APPLIED SCIENCES-BASEL 14(1). | wrong population | |
| 1686 | Legault, I., et al. (2013). "Healthy older observers show equivalent perceptual-cognitive training benefits to young adults for multiple object tracking." Frontiers in Psychology 4: 7. | wrong population | |
| 1687 | Lehretz, F., et al. (2020). "Enhancement of Acid Stability of Silicone Elastomers by Using Inert Fillers." IEEE TRANSACTIONS ON DIELECTRICS AND ELECTRICAL INSULATION 27(6): 2188-2194. | wrong population | |
| 1688 | Leicht, C. A., et al. (2019). "Hot water immersion acutely increases postprandial glucose concentrations." PHYSIOLOGICAL REPORTS 7(20). | wrong population | |
| 1689 | Leite, S., et al. (2019). "Physiological Arousal Quantifying Perception of Safe and Unsafe Virtual Environments by Older and Younger Adults." SENSORS 19(11). | wrong population | |
| 1690 | Lenhart, J., et al. (2022). "The Relationship Between Lifetime Book Reading and Empathy in Adolescents: Examining Transportability as a Moderator." PSYCHOLOGY OF AESTHETICS CREATIVITY AND THE ARTS 16(4): 679-693. | wrong population | |
| 1691 | Lenzi, T. L., et al. (2014). "Shortening the etching time for etch-and-rinse adhesives increases the bond stability to simulated caries-affected primary dentin." J Adhes Dent 16(3): 235-241. | wrong population | |
| 1692 | León, I., et al. (2014). "Developmental gender differences in children in a virtual spatial memory task." Neuropsychology 28(4): 485-495. | wrong population | |
| 1693 | Leopardi, A. C., et al. (2023). "Effectiveness of Virtual Reality Goggles as Distraction for Children in Dental Care-A Narrative Review." APPLIED SCIENCES-BASEL 13(3). | wrong population | |
| 1694 | Lercher, K., et al. (2023). "Distal and Proximal Predictors of Rehospitalization Over 10 Years Among Survivors of TBI: A National Institute on Disability, Independent Living, and Rehabilitation Research Traumatic Brain Injury Model Systems Study." J Head Trauma Rehabil 38(3): 203-213. | wrong population | |
| 1695 | Leroy, S. A. G., et al. (2019). "Human responses to environmental change on the southern coastal plain of the Caspian Sea during the Mesolithic and Neolithic periods." QUATERNARY SCIENCE REVIEWS 218: 343-364. | wrong population | |
| 1696 | Lesk, V. E., et al. (2014). "Using a virtual environment to assess cognition in the elderly." VIRTUAL REALITY 18(4): 271-279. | wrong outcomes | |
| 1697 | Letaief, W. E., et al. (2016). "Effect of Hydrogen on the Stress Relaxation of Aged NiTi Shape Memory Alloys." ACTA PHYSICA POLONICA A 129(4): 714-716. | wrong population | |
| 1698 | Leton, N. (2024). "Senescence Seclusion Syndrome: Appraising the Efficacy of Current Interventions." CUREUS JOURNAL OF MEDICAL SCIENCE 16(6). | wrong population | |
| 1699 | Leung, C., et al. (2022). "The application of technology to improve cognition in older adults: A review and suggestions for future directions." Psych J 11(4): 583-599. | wrong outcomes | |
| 1700 | Levins, J. G., et al. (2024). "Anatomic shoulder arthroplasty: the correlation between patient resilience, mental health, and outcome." JOURNAL OF SHOULDER AND ELBOW SURGERY 33(6): S9-S15. | wrong intervention | |
| 1701 | Levinsson, A., et al. (2021). "Conspiracy Theories, Psychological Distress, and Sympathy for Violent Radicalization in Young Adults during the COVID-19 Pandemic: A Cross-Sectional Study." INTERNATIONAL JOURNAL OF ENVIRONMENTAL RESEARCH AND PUBLIC HEALTH 18(15). | wrong population | |
| 1702 | Levkovich, I., et al. (2024). "&quot;Hearing the pupils' voices through my own struggles": A qualitative study of return to work among school counselors who are breast cancer survivors." PLOS ONE 19(5). | wrong population | |
| 1703 | Levy, F., et al. (2016). "Fear of falling: Efficacy of virtual reality associated with serious games in elderly people." Neuropsychiatric Disease and Treatment 12: 5. | wrong design | |
| 1704 | Lewandowski, J. and K. Lukaszewska (2014). "Characteristics of back pain in Polish youth depending on place of residence." ANNALS OF AGRICULTURAL AND ENVIRONMENTAL MEDICINE 21(3): 644-648. | wrong population | |
| 1705 | Li, G., et al. (2022). "Effects of virtual reality-based interventions on the physical and mental health of older residents in long-term care facilities: A systematic review." International Journal of Nursing Studies 136: 1-10. | wrong outcomes | |
| 1706 | Li, G. C., et al. (2022). "Effects of virtual reality-based interventions on the physical and mental health of older residents in long-term care facilities: A systematic review." INTERNATIONAL JOURNAL OF NURSING STUDIES 136. | duplicate |  |
| 1707 | Li, H., et al. (2022). "Association of Parental Screen Addiction with Young Children's Screen Addiction: A Chain-Mediating Model." INTERNATIONAL JOURNAL OF ENVIRONMENTAL RESEARCH AND PUBLIC HEALTH 19(19). | wrong population | |
| 1708 | Li, H. L., et al. (2022). "Effects of seawater temperature and NaCl concentration on interlaminar shear behavior of CFRP laminates." MATERIALS RESEARCH EXPRESS 9(2). | wrong population | |
| 1709 | Li, H. Y., et al. (2023). "Effects of immersion in a simulated natural environment on stress reduction and emotional arousal: A systematic review and meta-analysis." FRONTIERS IN PSYCHOLOGY 13. | wrong intervention | |
| 1710 | Li, J. G., et al. (2022). "RETRACTED: Effects of Artificial Intelligence and Virtual Reality in Martial Arts Sports on Students' Physical and Mental Health (Retracted Article)." INTERNATIONAL TRANSACTIONS ON ELECTRICAL ENERGY SYSTEMS 2022. | wrong population | |
| 1711 | Li, J. H., et al. (2014). "Game-Based Digital Interventions for Depression Therapy: A Systematic Review and Meta-Analysis." CYBERPSYCHOLOGY BEHAVIOR AND SOCIAL NETWORKING 17(8): 519-527. | wrong population | |
| 1712 | Li, J. H., et al. (2016). "Effect of Exergames on Depression: A Systematic Review and Meta-Analysis." CYBERPSYCHOLOGY BEHAVIOR AND SOCIAL NETWORKING 19(1): 34-42. | wrong population | |
| 1713 | Li, Q., et al. (2024). Embracing Virtual Reality: Understanding Factors Influencing Older Adults' Acceptance. DISTRIBUTED, AMBIENT AND PERVASIVE INTERACTIONS, PT II, DAPI 2024. 14719: 32-54. | wrong design | |
| 1714 | Li, R., et al. (2024). "Virtual Reality–Based Training in Chronic Low Back Pain: Systematic Review and Meta-Analysis of Randomized Controlled Trials." Journal of Medical Internet Research 26(1). | wrong outcomes | |
| 1715 | Li, R. Z., et al. (2021). "Rehabilitation training based on virtual reality for patients with Parkinson's disease in improving balance, quality of life, activities of daily living, and depressive symptoms: A systematic review and meta-regression analysis." CLINICAL REHABILITATION 35(8): 1089-1102. | wrong population | |
| 1716 | Li, W. H. C., et al. (2011). "The effectiveness of therapeutic play, using virtual reality computer games, in promoting the psychological well-being of children hospitalised with cancer." JOURNAL OF CLINICAL NURSING 20(15-16): 2135-2143. | wrong population | |
| 1717 | Li, W. H. C., et al. (2011). "Effectiveness and feasibility of using the computerized interactive virtual space in reducing depressive symptoms of Hong Kong Chinese children hospitalized with cancer." JOURNAL FOR SPECIALISTS IN PEDIATRIC NURSING 16(3): 190-198. | wrong population | |
| 1718 | Li, X. X., et al. (2024). "A randomized controlled trial of a self-led, virtual reality-based cognitive behavioral therapy on sick role adaptation in colorectal cancer patients: study protocol." BMC CANCER 24(1). | wrong population | |
| 1719 | Li, Y. and B. Muschalla (2024). "Virtual Reality Mental Health Interventions in Geriatric Care for Functional or Well-being Enhancement - A Scoping Review." Rehabilitation (Stuttg) 63(4): 209-219. | wrong outcomes | |
| 1720 | Li, Y. and B. Muschalla (2024). "Virtual Reality Mental Health Interventions in Geriatric Care for Functional or Well-being Enhancement – A Scoping Review." Rehabilitation 63(4): 209-219. | duplicate |  |
| 1721 | Li, Y., et al. (2024). "Impact of Virtual Reality-Based Group Activities on Activity Level and Well-Being Among Older Adults in Nursing Homes: Longitudinal Exploratory Study." JMIR Serious Games 12: e50796. | wrong outcomes | |
| 1722 | Li, Y., et al. (2015). Partial Discharge Features and Parameters Characterization Under Typical Defect Geometries in Oil-Paper Insulation: Considering the Aging States. 2015 IEEE PES ASIA-PACIFIC POWER AND ENERGY ENGINEERING CONFERENCE (APPEEC). | wrong population | |
| 1723 | Li, Y., et al. (2023). "Research on patient-centered design for post-stroke depression patients based on SEM and comprehensive evaluation." Front Public Health 11: 1120596. | wrong population | |
| 1724 | Li, Y. J., et al. (2024). "Impact of Virtual Reality-Based Group Activities on Activity Level and Well-Being Among Older Adults in Nursing Homes: Longitudinal Exploratory Study." JMIR SERIOUS GAMES 12. | duplicate |  |
| 1725 | Li, Y. M., et al. (2014). "Vision-Related Quality of Life in Patients with Infectious Keratitis." OPTOMETRY AND VISION SCIENCE 91(3): 278-283. | wrong population | |
| 1726 | Li, Y. X., et al. (2023). "Research on patient-centered design for post-stroke depression patients based on SEM and comprehensive evaluation." FRONTIERS IN PUBLIC HEALTH 11. | duplicate |  |
| 1727 | Li, Y. Y., et al. (2024). "Virtual reality-based cognitive-behavioural therapy for the treatment of anxiety in patients with acute myocardial infarction: a randomised clinical trial." GENERAL PSYCHIATRY 37(2). | wrong population | |
| 1728 | Li, Z. Y., et al. (2023). "Violent Radicalization, Mental Health, and Gender Identity Considerations for Future Research." JOURNAL OF NERVOUS AND MENTAL DISEASE 211(3): 244-247. | wrong population | |
| 1729 | Lialy, H. E., et al. (2024). "Virtual reality gaming for rehabilitation of patients with urinary incontinence: A systematic review and meta-analysis." PHYSIOTHERAPY RESEARCH INTERNATIONAL 29(4). | wrong outcomes | |
| 1730 | Liang, F. Y., et al. (2015). "Panigarh cave stalagmite evidence of climate change in the Indian Central Himalaya since AD 1256: Monsoon breaks and winter southern jet depressions." QUATERNARY SCIENCE REVIEWS 124: 145-161. | wrong population | |
| 1731 | Liang, Z. S., et al. (2017). "EFFECT OF SIMULATED CORROSION ENVIRONMENT ON MECHANICAL PERFORMANCES OF SAND FIXATION BY HYDROPHILIC POLYURETHANE." FRESENIUS ENVIRONMENTAL BULLETIN 26(10): 5797-5805. | wrong population | |
| 1732 | Liao, Y.-Y., et al. (2019). "Effects of virtual reality-based physical and cognitive training on executive function and dual-task gait performance in older adults with mild cognitive impairment: A randomized control trial." Frontiers in Aging Neuroscience 11: 10. | wrong outcomes | |
| 1733 | Libeson, L., et al. (2022). "Factors associated with employment stability following traumatic brain injury, in a sample who have received comprehensive vocational rehabilitation." Disability and Rehabilitation: An International, Multidisciplinary Journal 44(21): 6325-6332. | wrong population | |
| 1734 | Lier, E. J., et al. (2018). "Modulation of tactile perception by Virtual Reality distraction: The role of individual and VR-related factors." PLOS ONE 13(12). | wrong population | |
| 1735 | Lieu, F. K., et al. (2014). "Effect of swimming on the production of aldosterone in rats." PLoS One 9(10): e87080. | wrong population | |
| 1736 | Liew, S. L., et al. (2023). "Association of Brain Age, Lesion Volume, and Functional Outcome in Patients With Stroke." Neurology 100(20): e2103-e2113. | wrong population | |
| 1737 | Lieze, M., et al. (2020). "Using virtual reality to investigate physical environmental factors related to cycling in older adults: A comparison between two methodologies." JOURNAL OF TRANSPORT & HEALTH 19. | wrong outcomes | |
| 1738 | Ligman, K., et al. (2021). "Jealousy and electronic intrusion mediated by relationship uncertainty in married and cohabiting couples during COVID-19." Cyberpsychology, Behavior, and Social Networking 24(7): 444-449. | wrong population | |
| 1739 | Lim, C. T. and B. Khoo (2000). Normal Physiology of ACTH and GH Release in the Hypothalamus and Anterior Pituitary in Man. Endotext. K. R. Feingold, B. Anawalt, M. R. Blackman et al. South Dartmouth (MA), MDText.com, Inc. | wrong population | |
| 1740 | Lim, H., et al. (2020). "Transcranial Direct Current Stimulation Effect on Virtual Hand Illusion." CYBERPSYCHOLOGY BEHAVIOR AND SOCIAL NETWORKING 23(8): 541-549. | wrong population | |
| 1741 | Lim, J., et al. (2015). "Impact of blood pressure perturbations on arterial stiffness." Am J Physiol Regul Integr Comp Physiol 309(12): R1540-1545. | wrong population | |
| 1742 | Lim, J. E., et al. (2020). "A Fully-Immersive and Automated Virtual Reality System to Assess the Six Domains of Cognition: Protocol for a Feasibility Study." Front Aging Neurosci 12: 604670. | wrong outcomes | |
| 1743 | Lim, J. E., et al. (2021). "A fully-immersive and automated virtual reality system to assess the six domains of cognition: Protocol for a feasibility study." Frontiers in Aging Neuroscience 12: 9. | duplicate |  |
| 1744 | Lim, W. T. and D. J. Torpy (2000). Chronic Fatigue Syndrome. Endotext. K. R. Feingold, B. Anawalt, M. R. Blackman et al. South Dartmouth (MA), MDText.com, Inc. | wrong population | |
| 1745 | Limoncu, H., et al. (2021). "A Virtual Reality-Based Screening Test for Cognitive Impairment in Small Vessel Disease." JOURNAL OF ALZHEIMERS DISEASE REPORTS 5(1): 161-169. | wrong intervention | |
| 1746 | Lin, C., et al. (2023). "The effectiveness of virtual reality games in improving cognition, mobility, and emotion in elderly post-stroke patients: a systematic review and meta-analysis." Neurosurg Rev 46(1): 167. | wrong population | |
| 1747 | Lin, C. L., et al. (2023). "The effectiveness of virtual reality games in improving cognition, mobility, and emotion in elderly post-stroke patients: a systematic review and meta-analysis." NEUROSURGICAL REVIEW 46(1). | duplicate |  |
| 1748 | Lin, C. S., et al. (2018). "The Elderly Perceived Meanings and Values of Virtual Reality Leisure Activities: A Means-End Chain Approach." Int J Environ Res Public Health 15(4). | wrong design | |
| 1749 | Lin, C. W., et al. (2021). "A Novel Game-Based Intelligent Test for Detecting Elderly Cognitive Function Impairment." Comput Math Methods Med 2021: 1698406. | wrong intervention | |
[truncated: 350,147 more chars]
