# Supplementary material for: Immersive virtual reality-based intervention for psychological wellbeing among older adults: A systematic review and meta-analysis
Source: PLOS Digit Health. 2026 Jan 15;5(1):e0001110. doi: 10.1371/journal.pdig.0001110 (PMC12806852; doi:10.1371/journal.pdig.0001110)
Supplement: Attachment — Submitted filename: Response.docx [file pdig.0001110.s005.docx]

We've checked your submission and before we can proceed, we need you to address the following issues:

1. Your manuscript is a Systematic or Scoping Review. Please change the article type to 'Research Article', and ensure all submission questions are completed, noting the change in publication fees associated with Research Articles.

Response: thank you. We have changed the article type to ‘research article’ in the submission system and the change in APC fee is well-noted.

2. Where possible, we encourage authors to provide relevant data underlying their research with their submission in order to facilitate editorial and peer review, even if the data may be restricted from publication or made private by a data repository prior to article acceptance. Many repositories permit private access for review purposes, and have policies for public release at publication. For data concerning human research subjects, please anonymize the data as appropriate and follow pertinent legal and ethical protocols prior to sharing with editors and reviewers. If you are unable to share any pertinent data at the time of editorial and peer review, please kindly let the journal office know.
Response: thank you. We have added data availability statement: The numerical data used for pooling is presented in Figures 4-6, while the textual data utilized in this study is shown in Tables 1-2. For more detailed data sharing, interested parties may contact the corresponding author upon reasonable request.

3. Please include a title page at the beginning of your manuscript file, that lists full author names and institute addresses. Do not upload as a separate file. Make sure your Title is written in standard English.

Response: thank you. We have included a title page at the beginning of the manuscript.

4. Please ensure that the Author List in your manuscript file matches the Author List in the online submission form exactly. Authors should be listed in the same order, and the order of individual first and last author names must be identical in both locations.

Response: we have ensured the consistency of author list.

5. We note that this manuscript is a systematic review or meta-analysis; our author guidelines require that you upload a PRISMA checklist as supporting information. Information about the PRISMA guidance and blank checklists can be found here: <http://www.prisma-statement.org/>. Please note that if your study analyzes clinical trials, we cannot proceed with consideration of your article until we have this information.

Response: Thank you, we have uploaded our checklist.
