# Supplementary material for: Immersive virtual reality-based intervention for psychological wellbeing among older adults: A systematic review and meta-analysis
Source: PLOS Digit Health. 2026 Jan 15;5(1):e0001110. doi: 10.1371/journal.pdig.0001110 (PMC12806852; doi:10.1371/journal.pdig.0001110)
Supplement: Attachment — Submitted filename: Manuscript Review.docx [file pdig.0001110.s003.docx]

**REVIEW OF IMMERSIVE VIRTUAL REALITY-BASED INTERVENTION FOR PSYCHOLOGICAL WELLBEING AMONG OLDER ADULTS: A SYSTEMATIC REVIEW AND META-ANALYSIS**

**Abstract**

The abstract gives a clear overview of the study’s purpose, methods, and findings. It’s helpful that the authors identify immersive virtual reality (IVR) as a growing therapeutic option for older adults and break down the types of interventions used. The mention of multiple databases, a defined search period, and standard review tools (RevMan, Cochrane Risk of Bias) shows the work was methodologically organized. Including the number of trials, participants, and the proportion of each intervention type gives readers a quick sense of scope and diversity.

That said, improvements can be made. First, the statistical reporting uses both confidence intervals and p-values for every outcome, which is unnecessary. Since either can show statistical significance, it’s better to pick one. In this context, confidence intervals should be retained because they convey both effect size and precision, while p-values can be dropped. Second, there is also an inconsistency in the anxiety result, the effect size (SMD = 0.07) is outside the confidence interval (-0.99, -0.44) reported, suggesting either a reporting error or a misinterpretation. Please address this.

Lastly, the “synthesis without meta-analysis” part is too vague, as it only states there was an improvement without indicating the size or direction of the effect. Even a brief qualifier, such as whether the change was small, moderate, or large, would help readers better understand and judge its practical significance.

**Introduction**

The introduction opens with a broad statement about *“mental challenges”* in older adults, but it would help if the authors specified what these challenges are beyond anxiety and depression. Being more precise here would give the reader a clearer sense of the problem’s scope and why targeted interventions are needed. The flow is fine overall, but breaking up the long opening sentence and trimming extra words would make it easier to read. Also, the connection between the first paragraph (mental health challenges in older adults) and the second (introduction of IVR) could be improved, for example, by directly stating how IVR addresses the specific barriers or needs identified earlier, such as mobility limitations, stigma, or reduced engagement in traditional therapy. The reader has to infer this link.

Additionally, the introduction outlined the benefits of IVR, and it is heavily weighted toward the positive aspects. Acknowledging practical challenges like cost, accessibility, and user familiarity would provide a more balanced view for the reader.

Lastly, the literature gap section toward the end is important but could be clearer. The authors note that past reviews included different types of IVR interventions, which may have diluted conclusions, but they stop short of explaining why psychological interventions specifically deserve isolated focus. Clarifying this point, and linking it directly to how the current review addresses that gap would strengthen the rationale. Finally, the aim statement could be more concise and assertive, focusing on the central question without extra qualifiers, so it lands with more impact.

**Methods**

The methods section is detailed and includes all the core elements expected in a well-conducted systematic review and meta-analysis. It is a strength that the authors follow the Cochrane Handbook and PRISMA guidelines, register their protocol in PROSPERO, and structure the eligibility criteria using the PICOS framework. The inclusion of multiple databases, a clear search period starting from the commercial availability of immersive VR, and a hand search of relevant references demonstrate a thorough approach to identifying studies. The dual independent screening, quality assessment, and data extraction steps also indicate methodological rigor and an effort to reduce bias.

That said, there are areas that need improvement. The eligibility criteria, while comprehensive, could be made more concise by avoiding repetition of concepts already covered under PICOS. For example, stating *“only RCTs were included”* could be integrated into the study design criterion rather than repeated later. The rationale for excluding non-immersive VR studies should be linked more clearly to the research question so readers understand why this distinction is important for psychological outcomes. In the search methods, the decision to start in 2010 is logical, but this would be stronger if supported by a citation or milestone showing when immersive VR became broadly accessible. The MeSH search string is thorough but long, and would be better placed in an appendix to improve readability.

The study selection section lists *“team A: JJS, LB, AC” and “team B: RK, AL, AC,”* but the authors should spell out the researchers’ full names rather than initials. This improves clarity and transparency for readers and is standard practice in high-quality reviews. In the data analysis section, the interchangeable use of “Cohen’s d” and “SMD” could be misleading, since SMD in meta-analyses is not always identical to Cohen’s d depending on computation. This should be clarified. Finally, while the GRADE approach is mentioned, more detail on how it was applied (e.g., criteria for downgrading evidence quality) would improve transparency.

**Results**

The results section is comprehensive, covering study selection, intervention characteristics, participant details, and the pooled effects on key outcomes. The PRISMA flow diagram is clear, showing a substantial narrowing from nearly 4,000 records to 10 included RCTs, which signals a focused evidence base. However, the narrative could flow better if the search outcome and study characteristics were integrated rather than repeated in separate places. Currently, the study characteristics section appears twice, which creates redundancy. Also, the authors present a wide variety of intervention types and delivery methods, which is informative, but they could be more explicit about how this heterogeneity might influence pooled effect estimates.

The intervention characteristics section is rich in detail, outlining both the therapeutic themes and the technology used. The tailoring strategies, such as personalized reminiscence content and customized music, are noteworthy, and the professional involvement in some interventions adds credibility. However, the variety in participant positioning, settings, and co-interventions (e.g., fitness or rehabilitation training) introduces potential confounders. The review would be stronger if the authors addressed how these variations were handled analytically or discussed their impact on outcome comparability. At times, the level of descriptive detail (e.g., specific types of scenic images) feels excessive for the main text and could be condensed, with more space devoted to summarizing common patterns across interventions.

The pooled analysis on depressive symptoms shows a consistent and statistically significant benefit for IVR interventions, with low heterogeneity (I² = 21%). This is a strong finding, but the discussion of certainty (“moderate certainty of evidence”) could better explain why it is not high, given the low heterogeneity and consistent direction of effect. For anxiety, the initial high heterogeneity (I² = 70%) is addressed with sensitivity analysis, which is a strength, but the reporting could be streamlined by presenting the reduced heterogeneity results more prominently. The presentation of stress and affect outcomes is narrative due to limited data, but the authors do well to summarize them; still, quantifying even rough effect sizes or directions where possible would give readers a better sense of magnitude.

The psychological well-being results show no significant pooled effect and high heterogeneity (I² = 91%), which is appropriately acknowledged. The authors also correctly note risk of bias issues, particularly the lack of blinding and unclear allocation methods in several studies. The risk of bias figures are clear, but they reveal that performance bias is a consistent weakness, this deserves a more explicit link to the interpretation of results, as unblinded psychosocial interventions are particularly vulnerable to expectancy effects. Finally, the reporting of adverse events is a welcome addition, but it could be better framed in terms of feasibility and safety implications for scaling up IVR interventions in older adults.

**Discussion**

The discussion section does a good job of summarizing the main findings and placing them in the context of existing literature. The emphasis on depressive symptoms and anxiety reduction is appropriate, and the link to prior research on both traditional interventions and nature-based therapies adds depth to the interpretation. The explanation of how IVR garden therapy and reminiscence therapy may work, through sensory engagement, comfort, and memory stimulation is clear and well-supported. The acknowledgment that overall psychological well-being did not improve significantly shows a balanced view, and the suggestion that interventions need to address multiple facets of well-being (e.g., meaning, life satisfaction, social skills) is well taken.

That said, the discussion could be more concise and better structured to avoid repetition. Several ideas—such as the immersive qualities of IVR, the use of personalization, and the role of mental health professionals, are covered in multiple places, which could be condensed into tighter, more focused paragraphs. Additionally, while the physiological rationale for IVR’s effects (e.g., suppressing sympathetic nervous activity) is interesting, it could be paired with a more critical appraisal of the evidence supporting these mechanisms in older adults. The section could also acknowledge that many included studies used small samples, limiting generalizability.

The implications are practical, particularly the advice to tailor interventions to patient preferences, report participant positioning, and combine IVR with other modalities. However, the discussion might be strengthened by addressing barriers to implementation, such as cost, technical literacy, and infrastructure in care facilities, which are important for real-world adoption. The suggestion to compare IVR to conventional interventions for cost-effectiveness is valuable and could be emphasized earlier as a research priority.

The limitations are appropriate, covering heterogeneity, limited number of studies, and methodological weaknesses in the included RCTs. Still, the authors could make clearer how these limitations might affect the strength of their conclusions, for example, whether the moderate certainty ratings for depression and anxiety could be downgraded due to small-study effects or performance bias. Ending with a more concise, forward-looking statement that ties the findings, implications, and research needs together would leave the reader with a stronger overall takeaway.

**Conclusion**

The conclusion provides a clear and positive summary of the main findings, highlighting IVR’s potential in reducing depressive symptoms, anxiety, and stress, and in improving affect. It also appropriately notes the promise of integrating IVR into standard psychological care, and the potential for technological advancements to enhance its effectiveness. The mention of improving interactivity and personalization is forward-looking and aligns well with the earlier discussion points.

However, the conclusion is almost entirely optimistic and could benefit from a more balanced tone. While it acknowledges small sample sizes, it does not fully convey how this and other methodological issues (e.g., heterogeneity, lack of blinding) limit the strength of the conclusions. A brief reminder that current evidence is moderate at best for certain outcomes, and inconsistent for overall psychological well-being, would better reflect the nuances of the review’s findings.

The call for rigorous trials with larger samples and longer follow-up is important, but it could be strengthened by also recommending more diverse populations and standardized outcome measures to improve comparability across studies. Finally, the first sentence could be refined to avoid overstating effectiveness, phrasing it as *“have shown potential effectiveness” or “are associated with improvements”* would make the claim more cautious and in line with the evidence presented.

Thank you.
