# Supplementary material for: Immersive virtual reality-based intervention for psychological wellbeing among older adults: A systematic review and meta-analysis
Source: PLOS Digit Health. 2026 Jan 15;5(1):e0001110. doi: 10.1371/journal.pdig.0001110 (PMC12806852; doi:10.1371/journal.pdig.0001110)
Supplement: Attachment — Submitted filename: Comments of immersive virtual reality manuscript.docx [file pdig.0001110.s004.docx]

# **General Impressions and comments:**

I am really honored to review this significant and remarkable work. Efforts and collaboration by all authors deserve many thanks and appreciation, as the manuscript demonstrates a beacon modality for all else.

The study implemented a robust methodology to review systematically the effectiveness of a critical concerns entitled "Utilities and Interventions of Virtual Technology". Description by the authors on the research methods, strategies, and implementation are lucid clear. Kudos to that.

The study and its approach represent a significant and timely matter, and demonstrate its eligibility for a broader readerships and scientific community.

One of the strategic implementation I would like to thank authors on is the way of handling disagreement. The authors have ensured a multi-pronged approach and a well-defined implementation strategy, with exemplary impact and effectiveness, ensured a high-quality manuscript, which greatly enhances the robustness and strength of the methodology employed in this systematic review and meta-analysis. For instance, the authors considered discussion among them when it comes to disagreement on study selection. This precise excellent strategy implemented are optimum and highly effective for a key aspect associated with the overall manuscript and its quality, preparation, and development. The collaborative discussions among the authors regarding study selection demonstrate an exemplary commitment to transparency and rigor in the review process, contributing significantly to the overall quality and preparation of the manuscript. In contrast, the authors considered a third author to solve disagreement on interpretation of the extracted data among 2 authors. Such a tailored policy are conclusive and categorical for a particular aspect that doesn't effect the overall quality, preparation and development of the manuscript, particularly when it comes to personal perspectives and preferences to elucidate findings from a similar data extracted. While the involvement of a third author to resolve disagreements on the interpretation of extracted data is commendable and reflects a tailored approach to ensuring comprehensive analysis, it is important to note that this strategy does not detract from the overall quality of the manuscript. Instead, it reinforces the integrity of the findings by addressing personal perspectives and preferences.

Overall, these strategies have positively influenced the manuscript’s development, ensuring that the conclusions drawn are well-founded and reflective of a thorough and balanced assessment of the evidence.

The manuscript comprehensively discuss the entire context of the research title and the overall landscape aspects, including alignment to findings from previous literature and limitations to consider in the future directions of upcoming work.

The authors demonstrated an efficient capacity to deliver the results/findings of this research in well-written statement under the conclusion section.

# **Minor Revision Feedback:**

There is a kind of meaningless iteration/repeat of the conjunctive adverb "However" in the last paragraph of the introduction section (However, the current uncertainty..../However, existing reviews...).

Please avoid such kind of repeated writing, particularly in introducing or contrasting 2 relevant statements using the same conjunction term. You may consider synonym term instead (E.x: Nonetheless, nevertheless...etc)

There is mismatching in data. In the Results section, the content texted under the subsection "search outcome" indicate the removal of 686 duplicates. However, duplicate n was equal to 544 in the figure of PRISMA flow diagram. Kindly modify with the correct matched data.

# **Overall Comment:**

I highly recommend the publication of this remarkable piece of evidence and science, as it demonstrates its uniqueness to be the first of its kind.
