# Supplementary material for: Immersive virtual reality-based intervention for psychological wellbeing among older adults: A systematic review and meta-analysis
Source: PLOS Digit Health. 2026 Jan 15;5(1):e0001110. doi: 10.1371/journal.pdig.0001110 (PMC12806852; doi:10.1371/journal.pdig.0001110)
Supplement: Attachment — Submitted filename: Response to editor and reviewers_20251002.docx [file pdig.0001110.s006.docx]

**Responses to Editor’s and reviewers’ comments on the manuscript entitled “Immersive Virtual reality-based intervention for psychological wellbeing among older adults: A systematic review and meta-analysis” (MS: PDIG-D25-00484)**

Dear Editors and Reviewers,

Thank you so much for your very valuable comments. We discussed your comments and revised them carefully. We provided answers to the questions you raised.

| Comments | Response |
| --- | --- |
| **Editor’s comments**  1. Fix internal inconsistencies and reporting errors: - The Results text states “after removing 686 duplicates,” while the PRISMA diagram shows “Duplicates n = 544.”. Please reconcile counts across text/figure and ensure every box total is auditable from your search logs. - The abstract reports SMD = 0.07; 95% CI (−0.99, −0.44), which conflicts with the main text/forest plot (SMD ≈ −0.77; 95% CI (−1.32, −0.22)). Please correct the sign and values and harmonize across Abstract, Results, and Figures. | Thank you for identifying the errors and inconsistencies. We have corrected the duplicate count (n = 686) and the SMD =−0.77; 95% CI, −1.32 to −0.22) throughout the manuscript. Please refer to the abstract (page 2, line 37) and the flowchart figure (page 9).  Abstract (Page 2, line 37):  Data pooling suggested that IVR interventions have significantly reduced depressive symptoms [n=5; SMD=-0.83, 95%CI (-1.05, -0.60), I^2^=21%, *p*<.001]; anxiety [n=5, SMD=-0.77, 95% CI (-1.32, -0.22), I^2^=70%, *p*=.006].  Main text:  Pooled data from five of these studies showed a significant reduction in anxiety [n = 5, SMD = -0.77, 95% CI (-1.32, -0.22), I² = 70%, *p* = .006].  Figure 5. Effect on anxiety   |
| 2. Clarify and justify eligibility decisions. - You exclude IVR physical and cognitive‑only training, but include trials where IVR was delivered alongside sensorimotor or rehabilitation components when psychological outcomes were primary or secondary. Please explain this rationale, define what you considered a “psychological” IVR intervention, and justify inclusion of mixed interventions. - Please define “immersive IVR” operationally (hardware, FoV, interaction) to distinguish from non‑immersive VR. | Thank you. We have clarified in the Abstract and Results sections that, in this included study, sensorimotor rehabilitation refers to multisensory stimuli aimed at improving psychological symptoms and self-perception. We have also added a definition of psychological intervention to the second paragraph. Page 4, line 76-78.  *Psychological intervention is a structured, non-pharmacological procedure that employs psychological principles to modify a person's emotions, attitudes, or behaviors through processes such as education, training, therapy, or support [1].*  We have also added the definition of immersive VR operationally to distinguish from the non-immersive VR, please see page 4, line 81-88  *Immersive virtual reality (IVR), a computer-based technology, is increasingly used in contemporary psychological interventions to enable older adults’ participation in structured psychotherapeutic activities [2, 3]. IVR is featured by its capacity to generate a compelling sense of physical presence within a computer‑generated environment. This spatial immersion is achieved through head‑mounted displays and related peripherals that envelop the user in coherent visual, auditory, and sensory stimuli, rendering the virtual setting perceptually authentic [4]. This differs from non-immersive VR, which utilizes devices like a monitor, keyboard, and mouse to display a 3D environment without enclosing the user to produce the same feeling of "being there." It is this immersive quality that unlocks the full potential of VR technology in delivering psychological intervention [4, 5].* |
| 3. For outcomes not meta‑analyzed (e.g., stress, affect), structure the narrative per SWiM guidance (what was grouped, how direction/size of effects were decided, and certainty considerations). | Thank you for the guidance. We have specified adherence to the SWiM guidance in the Abstract (page 2, 37-38) and Methods sections (page 8, line 180). We also revised the reporting of stress and affect outcomes in the Abstract (page 2, line 35-37) and Results sections (page 15, line 298- 308 and page 16, line 319-329). Effect sizes (Hedges’ g with a small-sample correction) were calculated for each individual study and presented for stress outcomes where data were available (page 16, line 304). |
| 4. The current statement (“available upon reasonable request”) is not compliant with PLOS Digital Heath policy. Please deposit the minimal dataset required to replicate the review (search strings, screening decisions, extraction sheet, risk‑of‑bias judgments, and the RevMan or analysis files) in a public repository with a persistent identifier, and update the Data Availability statement accordingly. | We followed your advice by depositing the dataset required to replicate the review, including search strings and screening decisions. They have been attached as Supplementary File S1. The data extraction sheet, risk-of-bias judgement, and meta-analysis data are included in the manuscript. The Data Availability statement has been updated to: “All data are included in the manuscript and Supporting Information file S1.” |
| **Reviewer #1**  Immersive virtual reality (IVR) is an emerging therapeutic modality that engages older adults in psychological therapeutically orientated activities developed to improve their psychological well-being…… can be published in the journal | Reviewer 1 provided a summary and complimentary remarks on this work and raised no issues requiring a response. To conserve space, the details are included on the final page of this letter. |
| **Reviewer #2**  It is a very important and interesting article, thank you for your efforts. I think it will be a strong reference for many future related works. So, I suggest to make two points (Optional): - Regarding tailored or personalized treatment especially in this context, can you suggest how to determine the most tailored or personalized intervention? - List most effective interventions according to your analysis, to be used as a reference in future works. | Thank you for this insightful suggestion. We have added this to the implication sessions, page 21, line 427-429. A dedicated review on tailoring strategies in IVR would be valuable once a larger body of tailored IVR intervention studies becomes available; we agree that this is an important research question.  The current study cannot address these comments, as the purpose of this review is not to identify strategies for determining the most tailored intervention. Nevertheless, tailored strategies used in interventions have been shown to be more effective [6]. This review also identified some tailored IVR interventions, for example, tailoring the IVR intervention to participants’ preferences and context (e.g., music and content), see page 20, line 391-394.  Similarly, the purpose of this review is not to identify which interventions are the most effective, but to compare whether IVR psychological intervention is more effective than control conditions. This is worth studying for future studies.  We have added this idea into implication, please see page 21, line 427-429:  *A dedicated review on tailoring strategies in IVR would be valuable once a larger body of tailored IVR intervention studies becomes available to identify the most effective intervention types or tailoring features.*  Meanwhile, we also acknowledged that due to the limited number of studies included, we are unable to offer definitive recommendations at this stage. This limitation precludes comparative analyses or meta-regression to identify the most effective intervention type or tailoring feature, see limitations section (page 22, line 442-444). |
| **Reviewer #3:**  Thank you for the opportunity to review this timely and relevant manuscript on immersive virtual reality (IVR)-based psychological interventions for older adults. The topic is essential, and the systematic review is methodologically well-grounded, with clear eligibility criteria, a comprehensive search strategy, and a balanced use of meta-analysis and narrative synthesis. The manuscript also does a good job of linking its findings to prior literature and highlighting their practical implications. | Thank you for your appreciation! |
| That said, there are several areas where the paper could be strengthened before publication. These include refining the introduction to specify the mental health challenges in older adults clearly and explicitly link them to the rationale for IVR, improving the flow and avoiding repetition in the discussion, clarifying specific methodological details (e.g., rationale for exclusion criteria, full author names in the study selection section, consistent use of effect size terminology), and presenting a more balanced interpretation of findings by giving equal weight to limitations and potential biases. | Thank you. We provide brief answers and explanation here to the general comments. Detailed responses and revisions are provided in our replies to your section-by-section comments.  The introduction was refined to specify the mental challenges to older adults, and the flow was revised to better align with the rational for IVR (page 4-6). |
| Statistical reporting can also be streamlined by using confidence intervals instead of both CIs and p-values in the abstract and results. | Thank you. We have used both CIs and p-values to align with recent publications in Plos Digital Health. |
| In the discussion and conclusion, I encourage the authors to temper overly optimistic language, especially where evidence is limited or heterogeneity is high, and to address potential real-world barriers to implementation such as cost, technical literacy, and care-setting infrastructure. Finally, while the conclusion appropriately calls for larger and more rigorous trials, it would be strengthened by also recommending more diverse study populations and standardized outcome measures to improve comparability across studies.  I have attached a detailed review of the manuscript, including specific section-by-section feedback and suggested revisions, for your consideration. | We have revised the Discussion and Conclusion to temper overly optimistic language and present a more balanced interpretation (page 17-23). Detailed responses and revisions are provided in our replies to your section-by-section comments. |
| **Abstract**  The abstract gives a clear overview of the study’s purpose, methods, and findings. It’s helpful that the authors identify immersive virtual reality (IVR) as a growing therapeutic option for older adults and break down the types of interventions used. The mention of multiple databases, a defined search period, and standard review tools (RevMan, Cochrane Risk of Bias) shows the work was methodologically organized. Including the number of trials, participants, and the proportion of each intervention type gives readers a quick sense of scope and diversity. | Thank you. |
| That said, improvements can be made. First, the statistical reporting uses both confidence intervals and p-values for every outcome, which is unnecessary. Since either can show statistical significance, it’s better to pick one. In this context, confidence intervals should be retained because they convey both effect size and precision, while p-values can be dropped. Second, there is also an inconsistency in the anxiety result, the effect size (SMD = 0.07) is outside the confidence interval (-0.99, -0.44) reported, suggesting either a reporting error or a misinterpretation. Please address this.  Lastly, the “synthesis without meta-analysis” part is too vague, as it only states there was an improvement without indicating the size or direction of the effect. Even a brief qualifier, such as whether the change was small, moderate, or large, would help readers better understand and judge its practical significance. | 1. We recognize that presenting both confidence intervals and p-values can be unnecessary; however, we report both, consistent with prior meta-analyses published in PLOS Digital Health. 2. Thank you for identifying the typographical error; we have corrected the SMD to −0.77. 3. We have revised the Synthesis Without Meta-analysis (SWiM) section in accordance with the SWiM guidance. We clarified which outcome parameters were grouped and the direction of effect in the Abstract, and provided more detailed information in the Results section of the manuscript (page 2, line 37-42). |
| Introduction  The introduction opens with a broad statement about “mental challenges” in older adults, but it would help if the authors specified what these challenges are beyond anxiety and depression. Being more precise here would give the reader a clearer sense of the problem’s scope and why targeted interventions are needed. The flow is fine overall, but breaking up the long opening sentence and trimming extra words would make it easier to read. Also, the connection between the first paragraph (mental health challenges in older adults) and the second (introduction of IVR) could be improved, for example, by directly stating how IVR addresses the specific barriers or needs identified earlier, such as mobility limitations, stigma, or reduced engagement in traditional therapy. The reader has to infer this link. | Thank you for the insightful comments. We have specified mental health challenges beyond anxiety and depression and streamlined the opening sentence to enhance readability (page 4, lines 63–68).  *As populations age worldwide, older adults experience a range of mental health challenges [7, 8]. Beyond predominant concern of anxiety and depression, these include loneliness and social isolation, chronic stress, and the psychological burden of multimorbidity and intrinsic capacity decline [7-9]. In response, the World Health Organization (WHO)  calls for reorienting health systems toward healthy aging and expanding access to evidence-based psychological interventions to prevent and alleviate later-life mental health problems [10].*  We also revised the final sentence of paragraph one and the opening sentence of paragraph two to improve the logical flow regarding how IVR may address these challenges (page 4, lines 72–79). |
| Additionally, the introduction outlined the benefits of IVR, and it is heavily weighted toward the positive aspects.  Acknowledging practical challenges like cost, accessibility, and user familiarity would provide a more balanced view for the reader. | Thank you for the insightful suggestion, the practical challenges of IVR were added to provide a more balanced view (page 5, line 112-115):  *Moreover, it’s implementation faces practical challenges such as cybersickness and visual discomfort; accessibility for individuals with sensory or cognitive impairments; the need for staff training, supervision, dedicated space; and device cost, maintenance, and technical support—all of which can affect adherence, safety, and effectiveness [11, 12].* |
| Lastly, the literature gap section toward the end is important but could be clearer. The authors note that past reviews included different types of IVR interventions, which may have diluted conclusions, but they stop short of explaining why psychological interventions specifically deserve isolated focus. Clarifying this point, and linking it directly to how the current review addresses that gap would strengthen the rationale. Finally, the aim statement could be more concise and assertive, focusing on the central question without extra qualifiers, so it lands with more impact. | We added a rationale for focusing exclusively on IVR-based psychological interventions to align with the scope of the current review (page 6, lines 119–123). We also streamlined the aim statement by removing extraneous qualifiers to make it sharper and more concise.  *Psychological IVR interventions warrant isolated focus because they uniquely leverage immersion and presence, stimulating emotional, cognitive, and behavioral processes that influence anxiety, depression, affect, and subjective well-being; moreover, they require distinct dosing, facilitation, and IVR content compared with non-psychological IVR [20]* |
| **Methods**  The methods section is detailed and includes all the core elements expected in a well-conducted systematic review and meta-analysis. It is a strength that the authors follow the Cochrane Handbook and PRISMA guidelines, register their protocol in PROSPERO, and structure the eligibility criteria using the PICOS framework. The inclusion of multiple databases, a clear search period starting from the commercial availability of immersive VR, and a hand search of relevant references demonstrate a thorough approach to identifying studies. The dual independent screening, quality assessment, and data extraction steps also indicate methodological rigor and an effort to reduce bias. | Thank you! |
| That said, there are areas that need improvement. The eligibility criteria, while comprehensive, could be made more concise by avoiding repetition of concepts already covered under PICOS. For example, stating *“only RCTs were included”* could be integrated into the study design criterion rather than repeated later. | The repetition in PICOS *“only RCTs were included”* was removed. |
| The rationale for excluding non-immersive VR studies should be linked more clearly to the research question so readers understand why this distinction is important for psychological outcomes | The Exclusion Criteria section now provides an expanded rationale for excluding non-immersive VR studies (page 7, line 143-145).  *Studies without attempting to immerse the participants inside the technology generated world or step inside the synthetic world (non-immersive mode), because our focus is on IVR-specific mechanisms—such as sense of presence and multisensory immersion—that are theorized to drive psychological outcomes.* |
| In the search methods, the decision to start in 2010 is logical, but this would be stronger if supported by a citation or milestone showing when immersive VR became broadly accessible. | Thank you, citation was added to support the milestone in IVR development. Page 7, line 152.  *The initiation time was set to 2010, during which low cost immersive virtual reality technology became commercially available [13].* |
| The MeSH search string is thorough but long and would be better placed in an appendix to improve readability. | Thank you, this is removed to supplementary file S1. |
| The study selection section lists *“team A: JJS, LB, AC” and “team B: RK, AL, AC,”* but the authors should spell out the researchers’ full names rather than initials. This improves clarity and transparency for readers and is standard practice in high-quality reviews. | Thank you for highlighting the issue of clarity and transparency. Consistent with practices in PLOS Digital Health, where author names are presented as initials, we have adopted initials to align with the journal’s conventions. |
| In the data analysis section, the interchangeable use of “Cohen’s d” and “SMD” could be misleading, since SMD in meta-analyses is not always identical to Cohen’s d depending on computation. This should be clarified. | Yes. The formula implemented in RevMan is Hedges’ adjusted g, which is similar to, but not identical to, Cohen’s d. Thank you for identifying this issue. We have clarified that, for each outcome of interest, the intervention effect size was expressed as Hedges’ adjusted g [27]. To support this clarification, we have cited the Statistical Methods Group guideline of The Cochrane Collaboration (page 9, line 184) |
| Finally, while the GRADE approach is mentioned, more detail on how it was applied (e.g., criteria for downgrading evidence quality) would improve transparency. | Thank you, more details about the GRADE approach were added to the data analysis section (page 9, line 191-197).  *The certainty of evidence was assessed using the Grading of Recommendations Assessment, Development and Evaluation (GRADE) approach. Under GRADE, RCTs start as high-certainty evidence and are downgraded for risk of bias, inconsistency, indirectness, imprecision, or publication bias [14]. Tests for funnel plot asymmetry were not undertaken in this meta-analysis because fewer than ten studies were available, a number too small to reliably distinguish random variation from true asymmetry. Instead, publication bias was evaluated and reported in terms of selective outcome reporting by cross-referencing the included studies with their trial registrations and/or published protocols.* |
| Results  The results section is comprehensive, covering study selection, intervention characteristics, participant details, and the pooled effects on key outcomes. The PRISMA flow diagram is clear, showing a substantial narrowing from nearly 4,000 records to 10 included RCTs, which signals a focused evidence base. However, the narrative could flow better if the search outcome and study characteristics were integrated rather than repeated in separate places. Currently, the study characteristics section appears twice, which creates redundancy. | Thank you. We have summarized the study characteristics together with participants characteristics to avoid redundancy. |
| Also, the authors present a wide variety of intervention types and delivery methods, which is informative, but they could be more explicit about how this heterogeneity might influence pooled effect estimates. The intervention characteristics section is rich in detail, outlining both the therapeutic themes and the technology used. The tailoring strategies, such as personalized reminiscence content and customized music, are noteworthy, and the professional involvement in some interventions adds credibility.  However, the variety in participant positioning, settings, and co-interventions (e.g., fitness or rehabilitation training) introduces potential confounders. The review would be stronger if the authors addressed how these variations were handled analytically or discussed their impact on outcome comparability. At times, the level of descriptive detail (e.g., specific types of scenic images) feels excessive for the main text and could be condensed, with more space devoted to summarizing common patterns across interventions. | Thank you. We have removed fitness or rehabilitation training, as these are not co-interventions; rather, they constitute standard care received by both the intervention and control groups and should not be construed as confounders.  The therapeutic themes and tailoring strategies informed our discussion of factors that may have contributed to the observed intervention effects (page 20, lines 391–394).  We present variation in participant positioning to contextualize adverse events, and we have revised the Results (page 17, lines 331–335) and Discussion (page 21, lines 403–409) sections on adverse events to strengthen this linkage.  Thank you for the insightful suggestion regarding analytical approaches to intervention heterogeneity. However, we are unable to offer definitive recommendations at this stage because the limited number of included studies precludes adequately powered comparative subgroup analyses or meta-regression to identify the most effective intervention types or tailoring features. We have acknowledged this in the Limitations section (page 22, lines 443–444). |
| The pooled analysis on depressive symptoms shows a consistent and statistically significant benefit for IVR interventions, with low heterogeneity (I² = 21%). This is a strong finding, but the discussion of certainty (“moderate certainty of evidence”) could better explain why it is not high, given the low heterogeneity and consistent direction of effect. | Certainty of the evidence was rated as moderate, downgraded by one level for risk of bias due to lack of blinding of participants and personnel in most studies and unclear allocation concealment in two studies, which could increase the risk of performance and selection bias for subjective outcomes of self-reported depressive symptoms (page 14, line 274-278). |
| For anxiety, the initial high heterogeneity (I² = 70%) is addressed with sensitivity analysis, which is a strength, but the reporting could be streamlined by presenting the reduced heterogeneity results more prominently. | Thank you, we have revised the statement to be more prominent (page 15, line 287-290).  *A sensitivity analysis excluding one study that compared IVR with Schultz’s Autogenic Training [36], a desensitization technique, showed a large, statistically significant reduction in anxiety with minimal heterogeneity [n=4, SMD=-1.01, 95% CI (-1.35, -0.68), I^2^=1%, p<.001].* |
| The presentation of stress and affect outcomes is narrative due to limited data, but the authors do well to summarize them; still, quantifying even rough effect sizes or directions where possible would give readers a better sense of magnitude. | Thank you for the guidance. We have specified adherence to the SWiM guidance in the Abstract (page 2) and Methods sections (page 8, line 180). We also revised the reporting of stress and affect outcomes in the Abstract (page 2, line 37-42) and Results sections (page 15-16). Effect sizes (Hedges’ g with a small-sample correction) were calculated for each individual study and presented for stress outcomes where data were available (page 15, line 303-304). |
| The psychological well-being results show no significant pooled effect and high heterogeneity (I² = 91%), which is appropriately acknowledged. The authors also correctly note risk of bias issues, particularly the lack of blinding and unclear allocation methods in several studies. | Thank you. |
| The risk of bias figures are clear, but they reveal that performance bias is a consistent weakness, this deserves a more explicit link to the interpretation of results, as unblinded psychosocial interventions are particularly vulnerable to expectancy effects. | Thank you. We have clarified the certainty-of-evidence evaluation, following the GRADE approach in greater detail, and have explained how risk of bias contributed to downgrading the certainty of evidence for each outcome in the Results section (page 14, line 268-272; page 15, line 274-278). |
| Finally, the reporting of adverse events is a welcome addition, but it could be better framed in terms of feasibility and safety implications for scaling up IVR interventions in older adults. | Thank you. We have added one paragraph in the Discussion section regarding the feasibility and safety implications (page 20-21, line 303-411).  Two concerns regarding its implementation warrant attention. First, adverse events associated with IVR have been insufficiently assessed, despite their importance to research and clinical practice. High completion rates across studies, coupled with reports that cybersickness is generally mild or tolerable, indicate good usability and acceptability. Seated use and teleportation locomotion [52], together with contemporary advances in display latency, refresh rates, optics, and tracking [53], may substantially mitigate cybersickness; however, this requires further investigation among older adults. Second, feasibility issues related to cost, technical literacy, and infrastructure in healthcare facilities and setting, critical for real-world scale-up, also merit further study. |
| **Discussion**  The discussion section does a good job of summarizing the main findings and placing them in the context of existing literature. The emphasis on depressive symptoms and anxiety reduction is appropriate, and the link to prior research on both traditional interventions and nature-based therapies adds depth to the interpretation. The explanation of how IVR garden therapy and reminiscence therapy may work, through sensory engagement, comfort, and memory stimulation is clear and well-supported. The acknowledgment that overall psychological well-being did not improve significantly shows a balanced view, and the suggestion that interventions need to address multiple facets of well-being (e.g., meaning, life satisfaction, social skills) is well taken. | Thank you. |
| That said, the discussion could be more concise and better structured to avoid repetition. Several ideas—such as the immersive qualities of IVR, the use of personalization, and the role of mental health professionals, are covered in multiple places, which could be condensed into tighter, more focused paragraphs. | We have revised the parts regarding the unique strength of IVR to be more condensed and tighter (page 20, line 391- 402). |
| Additionally, while the physiological rationale for IVR’s effects (e.g., suppressing sympathetic nervous activity) is interesting, it could be paired with a more critical appraisal of the evidence supporting these mechanisms in older adults. | Thank you for the insights. Based on literature, we have added caution that although proposed physiological mechanisms sound plausible, evidence in older adults is limited as many studies use small samples, heterogeneous protocols and measures, unstandardized dosage, mechanistic claims should be considered provisional [20] (page 18, line 359-362). |
| The section could also acknowledge that many included studies used small samples, limiting generalizability. | The limited sample size and generalizability were acknowledged in the Limitation section (page 22, line 442-443). |
| The implications are practical, particularly the advice to tailor interventions to patient preferences, report participant positioning, and combine IVR with other modalities. However, the discussion might be strengthened by addressing barriers to implementation, such as cost, technical literacy, and infrastructure in care facilities, which are important for real-world adoption. The suggestion to compare IVR to conventional interventions for cost- effectiveness is valuable and could be emphasized earlier as a research priority. | Thank you. We have added one implication: future research may engage stakeholders to address implementation barriers, including cost, technical literacy, and infrastructure within care facilities that are critical to real-world adoption (page 22, line 438-440).  We have also emphasized the suggestion to compare IVR to conventional interventions for cost- effectiveness as the first research implication (page 21, line 425-427). |
| The limitations are appropriate, covering heterogeneity, limited number of studies, and methodological weaknesses in the included RCTs. Still, the authors could make clearer how these limitations might affect the strength of their conclusions, for example, whether the moderate certainty ratings for depression and anxiety could be downgraded due to small-study effects or performance bias. Ending with a more concise, forward-looking statement that ties the findings, implications, and research needs together would leave the reader with a stronger overall takeaway. | We have expanded the limitations section to acknowledge the limited number of studies, the small sample sizes in most trials, and methodological shortcomings that contributed to downgrading the certainty of evidence (page 22, line 442-443). We also strengthened the ending statement to provide a clearer, forward-looking takeaway (page 22, line 450-455):  *Across the included RCTs, inadequate allocation concealment and lack of blinding of participants and personnel increased risk of bias, leading to downgrading the certainty of evidence to low or moderate. Taking together, these constraints temper confidence in the observed effects and their applicability; future work should prioritize adequately powered, methodologically rigorous trials to clarify effectiveness, reduce heterogeneity, and generate decision-ready evidence for practice and policy.* |
| Conclusion  The conclusion provides a clear and positive summary of the main findings, highlighting IVR’s potential in reducing depressive symptoms, anxiety, and stress, and in improving affect. It also appropriately notes the promise of integrating IVR into standard psychological care, and the potential for technological advancements to enhance its effectiveness. The mention of improving interactivity and personalization is forward-looking and aligns well with the earlier discussion points. | Thank you. |
| However, the conclusion is almost entirely optimistic and could benefit from a more balanced tone. While it acknowledges small sample sizes, it does not fully convey how this and other methodological issues (e.g., heterogeneity, lack of blinding) limit the strength of the conclusions. A brief reminder that current evidence is moderate at best for certain outcomes, and inconsistent for overall psychological well-being, would better reflect the nuances of the review’s findings.  The call for rigorous trials with larger samples and longer follow-up is important, but it could be strengthened by also recommending more diverse populations and standardized outcome measures to improve comparability across studies. Finally, the first sentence could be refined to avoid overstating effectiveness, phrasing it as “have shown potential effectiveness” or “are associated with improvements” would make the claim more cautious and in line with the evidence presented. Thank you. | Thank you for suggesting more accurate and balanced conclusion. We have revised the conclusion section (page 23, line 457-465):  *IVR-based psychological interventions have demonstrated effectiveness in alleviating depressive symptoms, anxiety, stress, and improving affect among adults with low- to moderate-quality evidence. The immersiveness, interactivity, and individual tailoring of the included IVR interventions, along with their incorporation of evidence-based psychological therapies (e.g., nature exposure, reminiscence), may have contributed to the effectiveness. However, given study limitations, the lack of standardized intervention protocols and dosing, and inconclusive effects on psychological well-being, further research is warranted. Rigorous, adequately powered trials with evidence-based content, standardized dosage, and longer follow-up are essential to substantiate these promising findings.* |
| **Reviewer #4:**  General Impressions and comments: I am really honored to review this significant and remarkable work. Efforts and collaboration by all authors deserve many thanks and appreciation, as the manuscript demonstrates a beacon modality for all else……. | Thank you for your academic summary and appreciation! The general impressions and comments raised no issues requiring a response. To conserve space, the details are included on the final page of this letter. |
| Minor Revision Feedback: There is a kind of meaningless iteration/repeat of the conjunctive adverb "However" in the last paragraph of the introduction section (However, the current uncertainty..../However, existing reviews...). Please avoid such kind of repeated writing, particularly in introducing or contrasting 2 relevant statements using the same conjunction term. You may consider synonym term instead (E.x: Nonetheless, nevertheless...etc) | Thank you. We have refined the conjunctive adverbs in the Introduction to enhance the overall flow. |
| There is mismatching in data. In the Results section, the content texted under the subsection "search outcome" indicate the removal of 686 duplicates. However, duplicate n was equal to 544 in the figure of PRISMA flow diagram. Kindly modify with the correct matched data. | Thank you for identifying this error. We have corrected the value to 686 for consistency. |
| Overall Comment: I highly recommend the publication of this remarkable piece of evidence and science, as it demonstrates its uniqueness to be the first of its kind. | Thank you for your recommendation regarding its publication. |

Reviewer's Responses to Questions

**Comments to the Author**

1. Does this manuscript meet PLOS Digital Health’s [publication criteria](https://track.editorialmanager.com/CL0/https:%2F%2Fjournals.plos.org%2Fdigitalhealth%2Fs%2Fjournal-information%23loc-criteria-for-publication/1/010f0198ec8360c7-0f066f2d-49eb-4ad2-885a-88561d5734e9-000000/oAyPBouMs4eC-yWYg7NCdSAArd0GzgUcOLGK6DU42PY=226)? Is the manuscript technically sound, and do the data support the conclusions? The manuscript must describe methodologically and ethically rigorous research with conclusions that are appropriately drawn based on the data presented.

Reviewer #1: Yes

Reviewer #2: Yes

Reviewer #3: Yes

Reviewer #4: Yes

2. Has the statistical analysis been performed appropriately and rigorously?

Reviewer #1: Yes

Reviewer #2: N/A

Reviewer #3: Yes

Reviewer #4: Yes

3. Have the authors made all data underlying the findings in their manuscript fully available (please refer to the Data Availability Statement at the start of the manuscript PDF file)?

The [PLOS Data policy](https://track.editorialmanager.com/CL0/https:%2F%2Fjournals.plos.org%2Fdigitalhealth%2Fs%2Fdata-availability/1/010f0198ec8360c7-0f066f2d-49eb-4ad2-885a-88561d5734e9-000000/CP2_Yz6ZdKSGwyibTNHlhiex2Xao1yRODgysBeuqUmM=226) requires authors to make all data underlying the findings described in their manuscript fully available without restriction, with rare exception. The data should be provided as part of the manuscript or its supporting information, or deposited to a public repository. For example, in addition to summary statistics, the data points behind means, medians and variance measures should be available. If there are restrictions on publicly sharing data—e.g. participant privacy or use of data from a third party—those must be specified.

Reviewer #1: Yes

Reviewer #2: Yes

Reviewer #3: Yes

Reviewer #4: Yes

4. Is the manuscript presented in an intelligible fashion and written in standard English?

PLOS Digital Health does not copyedit accepted manuscripts, so the language in submitted articles must be clear, correct, and unambiguous. Any typographical or grammatical errors should be corrected at revision, so please note any specific errors here.

Reviewer #1: Yes

Reviewer #2: Yes

Reviewer #3: Yes

Reviewer #4: Yes

6. PLOS authors have the option to publish the peer review history of their article ([what does this mean?](https://track.editorialmanager.com/CL0/https:%2F%2Fjournals.plos.org%2Fdigitalhealth%2Fs%2Feditorial-and-peer-review-process%23loc-peer-review-history/1/010f0198ec8360c7-0f066f2d-49eb-4ad2-885a-88561d5734e9-000000/kATJsLp8XhWsyMBJuve68RGLwcHwqIx9oKeoUGQ2ykw=226)). If published, this will include your full peer review and any attached files.


**Do you want your identity to be public for this peer review?** If you choose “no”, your identity will remain anonymous but your review may still be made public.


For information about this choice, including consent withdrawal, please see our [Privacy Policy](https://track.editorialmanager.com/CL0/https:%2F%2Fwww.plos.org%2Fprivacy-policy/1/010f0198ec8360c7-0f066f2d-49eb-4ad2-885a-88561d5734e9-000000/KWjwJuoemlxgDLEZFtd_rks9ZvfOnoswq0UsEtHS2qU=226).

Reviewer #1: No

Reviewer #2: **Yes:** Jehad Omar Abualrob

Reviewer #3: **Yes:** Victor Ifechukwude Agboli

Reviewer #4: **Yes:** Omar Abdulkarim Saeed Alhammadi

Reviewer #1: Immersive virtual reality (IVR) is an emerging therapeutic modality that engages older adults in psychological therapeutically orientated activities developed to improve their psychological well-being. This systematic review aims to investigate the effects of IVR psychological intervention on psychological symptoms and well-being. A systematic review and meta-analysis was conducted following the Cochrane Handbook for
Systematic Reviews of Interventions. Six databases were searched, including Embase, PubMed, Web of Science, Scopus, CINAHL, and PsycINFO, covering the period from 2010 to December 2024. RevMan 5.3 was utilized for meta-analysis, and the Cochrane Risk of Bias tool was employed for quality assessment. Ten randomized controlled trials of 746 older adults were included. The IVR interventions employed reminiscence (40%), garden/forest therapy (40%), cognitive stimulation (10%), and sensor motor
rehabilitation (10%). Data pooling suggested that IVR interventions have significantly reduced depressive symptoms [n=5; SMD=-0.83, 95%CI (-1.05, -0.60), I2=21%, p<.001]; anxiety [n=5, SMD=0.07, 95% CI (-0.99, -0.44), I2=70%, p<.001]. Synthesis without meta-analysis showed significant improvement in stress level and affect. IVR based interventions could be an alternative method for alleviating the psychological symptoms of older adults.
Author summary This study investigated the effects of immersive virtual reality (IVR)-based psychological interventions in alleviating psychological symptoms and enhancing wellbeing among older adults. The novelty of this research lies in its pooling of results from updated randomized controlled trials that exclusively examine the immersive format of VR-delivered psychological interventions. Previous studies often combined varying levels of VR immersiveness or mixed different types of IVR-based interventions (e.g.,
exercise, cognitive assessment, psychological), resulting in high heterogeneity that impeded definitive conclusions. The impact of this study is demonstrated by employing advanced immersive technology to create vivid and interactive experiences (e.g.,
garden environments) that achieve therapeutic effects. The meta-analysis results revealed that IVR-based psychological interventions significantly reduced depressive symptoms and anxiety among older adults, indicating the high potential of this modality for improving psychological outcomes, particularly for those with mobility limitations.

can be published in the journal

Reviewer #4: General Impressions and comments:
I am really honored to review this significant and remarkable work. Efforts and collaboration by all authors deserve many thanks and appreciation, as the manuscript demonstrates a beacon modality for all else.
The study implemented a robust methodology to review systematically the effectiveness of a critical concerns entitled "Utilities and Interventions of Virtual Technology". Description by the authors on the research methods, strategies, and implementation are lucid clear. Kudos to that.
The study and its approach represent a significant and timely matter, and demonstrate its eligibility for a broader readerships and scientific community.
One of the strategic implementation I would like to thank authors on is the way of handling disagreement. The authors have ensured a multi-pronged approach and a well-defined implementation strategy, with exemplary impact and effectiveness, ensured a high-quality manuscript, which greatly enhances the robustness and strength of the methodology employed in this systematic review and meta-analysis. For instance, the authors considered discussion among them when it comes to disagreement on study selection. This precise excellent strategy implemented are optimum and highly effective for a key aspect associated with the overall manuscript and its quality, preparation, and development. The collaborative discussions among the authors regarding study selection demonstrate an exemplary commitment to transparency and rigor in the review process, contributing significantly to the overall quality and preparation of the manuscript. In contrast, the authors considered a third author to solve disagreement on interpretation of the extracted data among 2 authors. Such a tailored policy are conclusive and categorical for a particular aspect that doesn't effect the overall quality, preparation and development of the manuscript, particularly when it comes to personal perspectives and preferences to elucidate findings from a similar data extracted. While the involvement of a third author to resolve disagreements on the interpretation of extracted data is commendable and reflects a tailored approach to ensuring comprehensive analysis, it is important to note that this strategy does not detract from the overall quality of the manuscript. Instead, it reinforces the integrity of the findings by addressing personal perspectives and preferences.
Overall, these strategies have positively influenced the manuscript’s development, ensuring that the conclusions drawn are well-founded and reflective of a thorough and balanced assessment of the evidence.
The manuscript comprehensively discuss the entire context of the research title and the overall landscape aspects, including alignment to findings from previous literature and limitations to consider in the future directions of upcoming work.
The authors demonstrated an efficient capacity to deliver the results/findings of this research in well-written statement under the conclusion section.

References

1.	Hodges, L.J., Walker, J., Kleiboer, A. M., Ramirez, A. J., Richardson, A., Velikova, G., & Sharpe, M. , *What is a psychological intervention? A metareview and practical proposal.* Psycho‐oncology, 2011. **20**(5): p. 470-478.

2.	Dermody, G., et al., *The Role of Virtual Reality in Improving Health Outcomes for Community-Dwelling Older Adults: Systematic Review.* J Med Internet Res, 2020. **22**(6): p. e17331.

3.	Li Pira, G., et al., *The Use of Virtual Reality Interventions to Promote Positive Mental Health: Systematic Literature Review.* JMIR Ment Health, 2023. **10**: p. e44998.

4.	Freina, L., & Ott, M., *A literature review on immersive virtual reality in education: state of the art and perspectives*, in *In The international scientific conference elearning and software for education*. 2015. p. 10-1007.

5.	Su, J.J., et al., *Psychological eHealth Interventions for Patients With Cardiovascular Diseases: Systematic Review and Meta-Analysis.* J Med Internet Res, 2025. **27**: p. e57368.

6.	Baker, R., et al., *Tailored interventions to address determinants of practice.* Cochrane Database Syst Rev, 2015. **2015**(4): p. CD005470.

7.	World Health Organization. *Ageing and health*. 2024; Available from: <https://www.who.int/news-room/fact-sheets/detail/ageing-and-health>.

8.	World Health Organization. *Healthy life expectancy (HALE) at age 60 (years)*. 2021; Available from: <https://www.who.int/data/gho/data/indicators/indicator-details/GHO/gho-ghe-hale-healthy-life-expectancy-at-age-60>.

9.	Leung, A.Y.M., et al., *Intrinsic capacity of older people in the community using WHO Integrated Care for Older People (ICOPE) framework: a cross-sectional study.* BMC Geriatr, 2022. **22**(1): p. 304.

10.	World Health Organization. *Mental health of older adults*. 2023; Available from: <https://www.who.int/news-room/fact-sheets/detail/mental-health-of-older-adults>.

11.	Healy, D., et al., *Older Adults' Experiences and Perceptions of Immersive Virtual Reality: Systematic Review and Thematic Synthesis.* JMIR Serious Games, 2022. **10**(4): p. e35802.

12.	Kwan, R.Y.C., et al., *Effects of Virtual Reality Motor-Cognitive Training for Older People With Cognitive Frailty: Multicentered Randomized Controlled Trial.* Journal of Medical Internet Research, 2024. **26**.

13.	Snoswell, A.J., & Snoswell, C. L. , *Immersive virtual reality in health care: systematic review of technology and disease states.* JMIR Biomedical Engineering, 2019. **4**(1): p. e15025.

14.	Granholm, A., W. Alhazzani, and M.H. Moller, *Use of the GRADE approach in systematic reviews and guidelines.* Br J Anaesth, 2019. **123**(5): p. 554-559.
